# Global burden of larynx cancer, 1990-2017: estimates from the global burden of disease 2017 study

**DOI:** 10.18632/aging.102762

**Published:** 2020-02-08

**Authors:** Yujiao Deng, Meng Wang, Linghui Zhou, Yi Zheng, Na Li, Tian Tian, Zhen Zhai, Si Yang, Qian Hao, Ying Wu, Dingli Song, Dai Zhang, Jun Lyu, Zhijun Dai

**Affiliations:** 1 Department of Breast Surgery, The First Affiliated Hospital, College of Medicine, Zhejiang University, Hangzhou 310003, China; 2 Department of Oncology, The Second Affiliated Hospital of Xi’an Jiaotong University, Xi’an 710004, China; 3 Department of Clinical Research, The First Affiliated Hospital of Jinan University, Guangzhou 510632, China

**Keywords:** larynx cancer, global burden of disease, incidence, death, disability adjusted life-years

## Abstract

Larynx cancer is one of the most common cancers in head and neck. This study aimed to investigate the health burden of larynx cancer at global, regional, and national levels. We collected data of larynx cancer between 1990 and 2017 from the Global Burden of Disease study, including incidence, mortality, and disability adjusted life-years (DALYs). Estimated annual percentage changes (EAPCs) were calculated to assess the changes in age-standardized rate (ASR) of larynx cancer. From 1990 to 2017, LC incident cases increased by 58.67%; however, age-standardized incidence rate (ASIR) decreased, with an EAPC of -0.99. Additionally, the incident cases and ASIR of LC were 6-fold higher for male than those for female in 2017. Over the past 28 years, deaths and DALYs of larynx cancer increased by 33.84% and 25%. Contrarily, age-standardized death and DALY rate showed a downward trend. Incidence, death, and DALYs of larynx cancer were always the highest in people aged 50-69 years. Overall, all the ASRs showed downward trends globally. The majority of larynx cancer burden was observed in men, especially among male aged 50-69 years. South and East Asia carried the heaviest burden of larynx cancer worldwide.

## INTRODUCTION

Larynx cancer (LC) is the most common malignancy in otolaryngology. Its major histopathological form is laryngeal squamous cell cancer [1]. In total, LC occurred in 177,422 people and accounted for 94,771 LC-related deaths worldwide (1% of all new cancer cases and deaths) [2]. Incident cases and deaths of LC are approximately 7 times higher in men than that in women. In 2016, the age-standardized incidence rate (ASIR) of LC was 5.0/100,000 people among male and 0.7/100,000 people among female. The age-standardized death rate (ASDR) of LC was 3.0/100,000 people among male and 0.4/100,000 people among female [3]. The burden of head and neck cancer in Central and South America has been assessed previously [4]. The main risk factors of LC are tobacco and alcohol use, which show linear relationships with LC development [5–7]. Some environmental and dietary factors, including exposure to textile dust, polyaromatic hydrocarbons, and asbestos, human papillomavirus (HPV) infection, and red meat consumption, were reported to elevate the LC risk [8–15]. No comprehensive recent study which evaluated the distribution and burden of LC worldwide was published.

The Global Burden of Disease (GBD) study covered the data of 354 diseases and injuries in 195 countries and regions, presenting an opportunity for detailed evaluation of the distribution, burden, and trends of LC in different countries and regions. It including incidence, mortality, disability adjusted life-years (DALYs) and age-standardized rate (ASR) data among gender, age, socio-demographic index (SDI), region and country. Therefore, we conducted this study to evaluate the burden of LC, providing support to policy makers to make rational use of the limited available resources and formulate relevant policies.

## RESULTS

### Analysis of larynx cancer incidence worldwide

From 1990 to 2017, the global incident cases of LC increased from 132,740 to 210,610, with a total increase of 58.67%. Contrarily, the ASIR showed a downtrend with an EAPC of -0.99 (-1.14- -0.83), decreasing from 3.14 per 100,000 persons to 2.59 per 100,000 persons (Table 1). In addition, the incident cases of LC were 178,000 (173,940 – 182,530) in males, which were 6-fold higher than that in females, consistent with the trend of ASIR.

**Table 1:** The incidence of larynx cancer, and its temporal trends from 1990 to 2017.

**Characteristics**	**1990**		**2017**		**1990-2017**	
	**Incident cases No. ×10^3^ (95% UI)**	**ASIR per 100,000 No. (95% UI)**	**Incident cases No. ×10^3^ (95% UI)**	**ASIR per 100,000 No. (95% UI)**	**Change in Incidence Number No. (%)**	**EAPC No. (95% CI)**
Global	132.74 (129.27 - 136.05)	3.14 (3.06 - 3.22)	210.61 (206.42 - 215.54)	2.59 (2.54 - 2.65)	58.67	0.99 (0.83 - 1.14)
**Sex**						
Male	113.36 (109.98 - 116.37)	5.76 (5.60 - 5.92)	178.00 (173.94 - 182.53)	4.63 (4.52 - 4.74)	57.02	1.08 (0.93 - 1.23)
Female	19.38 (18.60 - 20.30)	0.87 (0.83 - 0.91)	32.61 (31.54 - 33.69)	0.77 (0.74 - 0.79)	68.26	0.66 (0.50 - 0.83)
**Socio-demographic index**						
High SDI	48.88 (47.99 - 50.04)	3.91 (3.84 - 4.01)	57.77 (56.12 - 59.52)	2.82 (2.74 - 2.91)	18.17	1.58 (1.46 - 1.71)
High-middle SDI	30.16 (29.47 - 31.31)	3.04 (2.97 - 3.16)	49.55 (48.01 - 51.13)	2.68 (2.59 - 2.76)	64.25	0.87 (0.65 - 1.09)
Middle SDI	20.81 (20.03 - 21.84)	2.10 (2.02 - 2.20)	49.05 (47.04 - 51.67)	2.17 (2.08 - 2.28)	135.76	0.02 (0.18 - 0.22)
Low-middle SDI	18.79 (17.01 - 20.01)	3.11 (2.82 - 3.32)	32.60 (30.62 - 34.79)	2.64 (2.48 - 2.82)	73.46	0.72 (0.62 - 0.82)
Low SDI	13.77 (11.81 - 15.35)	3.88 (3.34 - 4.31)	20.82 (18.95 - 22.48)	2.84 (2.59 - 3.07)	51.20	1.19 (1.00 - 1.38)
**Region**						
Andean Latin America	0.31 (0.29 - 0.34)	1.48 (1.37 - 1.61)	0.48 (0.44 - 0.53)	0.9 (0.81 - 0.99)	54.38	2.21 (2.00 - 2.41)
Australasia	0.71 (0.68 - 0.75)	3.01 (2.86 - 3.17)	0.95 (0.84 - 1.09)	2.08 (1.82 - 2.38)	33.27	1.73 (1.55 - 1.91)
Caribbean	1.05 (1.00 - 1.12)	3.97 (3.78 - 4.20)	2.38 (2.13 - 2.63)	4.64 (4.16 - 5.13)	125.46	0.56 (0.70 - 0.43)
Central Asia	1.52 (1.47 - 1.57)	2.95 (2.86 - 3.04)	1.74 (1.64 - 1.84)	2.20 (2.07 - 2.32)	14.80	1.32 (1.17 - 1.46)
Central Europe	7.17 (6.99 - 7.38)	4.68 (4.56 - 4.82)	8.69 (8.32 - 9.07)	4.39 (4.20 - 5.13)	21.19	0.40 (0.26 - 0.53)
Central Latin America	1.83 (1.79 - 1.89)	2.15 (2.11 - 2.23)	3.43 (3.21 - 3.64)	1.48 (1.39 - 1.57)	88.11	1.88 (1.73 - 2.04)
Central Sub-Saharan Africa	0.47 (0.37 - 0.57)	1.98 (1.62 - 2.36)	0.82 (0.67 - 0.98)	1.55 (1.30 - 1.83)	74.09	1.02 (0.92 - 1.11)
East Asia	14.41 (13.85 - 15.13)	1.53 (1.47 - 1.61)	41.89 (39.97 – 44.00)	1.98 (1.89 - 2.08)	190.73	1.01 (1.40 - 0.62)
Eastern Europe	13.18 (12.66 - 13.81)	4.52 (4.35 - 4.75)	12.27 (11.82 - 12.76)	3.67 (3.54 - 3.81)	6.88	1.48 (1.15 - 1.79)
Eastern Sub-Saharan Africa	1.58 (1.38 - 1.79)	1.92 (1.69 - 2.15)	2.33 (2.09 - 2.60)	1.38 (1.23 1.55)	47.49	1.51 (1.38 - 1.64)
High-income Asia Pacific	4.76 (4.57 - 4.96)	2.28 (2.18 - 2.37)	6.86 (6.47 - 7.26)	1.64 (1.56 - 1.74)	43.99	1.71 (1.53 - 1.90)
High-income North America	12.71 (12.46 - 13.03)	3.71 (3.63 - 3.80)	18.63 (17.95 - 19.28)	3.11 (3.00 - 3.22)	46.52	1.22 (1.01 - 1.44)
North Africa and Middle East	4.93 (4.55 - 5.38)	2.71 (2.51 - 2.99)	10.06 (9.45 - 10.63)	2.33 (2.19 - 2.47)	103.98	0.60 (0.56 - 0.64)
Oceania	0.06 (0.05 - 0.07)	1.91 (1.68 - 2.18)	0.14 (0.12 - 0.17)	2.05 (1.79 - 2.36)	134.75	0.42 (0.50 - 0.34)
South Asia	29.74 (26.62 - 32.02)	4.62 (4.14 - 4.97)	50.56 (47.88 - 53.44)	3.57 (3.38 - 3.77)	69.97	1.11 (0.90 - 1.31)
Southeast Asia	5.29 (4.87 - 5.99)	1.95 (1.80 - 2.20)	11.46 (10.46 - 13.56)	1.89 (1.73 - 2.23)	116.65	0.20 (0.14 - 0.27)
Southern Latin America	1.63 (1.56 - 1.71)	3.42 (3.27 - 3.57)	1.88 (1.69 - 2.10)	2.32 (2.08 - 2.59)	15.17	1.83 (1.65 - 2.02)
Southern Sub-Saharan Africa	0.68 (0.61 - 0.8)	2.3 (2.08 - 2.74)	1.06 (1.00 - 1.13)	1.84 (1.74 - 1.95)	56.32	1.09 (0.55 - 1.63)
Tropical Latin America	3.01 (2.93 - 3.11)	3.08 (3.00 - 3.18)	7.25 (7.02 - 7.49)	3.03 (2.93 - 3.13)	140.92	0.18 (0.10 - 0.27)
Western Europe	26.31 (25.56 - 27.17)	4.78 (4.64 - 4.94)	25.27 (24.06 - 26.64)	3.19 (3.03 - 3.37)	3.93	1.76 (1.63 - 1.90)
Andean Latin America	1.37 (1.14 - 1.65)	1.47 (1.24 - 1.76)	2.44 (2.07 - 2.92)	1.33 (1.14 -1.58)	78.69	0.31 (0.28 - 0.34)

ASIR: age standardized incidence rate; EAPC: estimated annual percentage change; CI: confidence interval; UI: uncertainty interval; SDI: socio-demographic index

**Figure 1:**
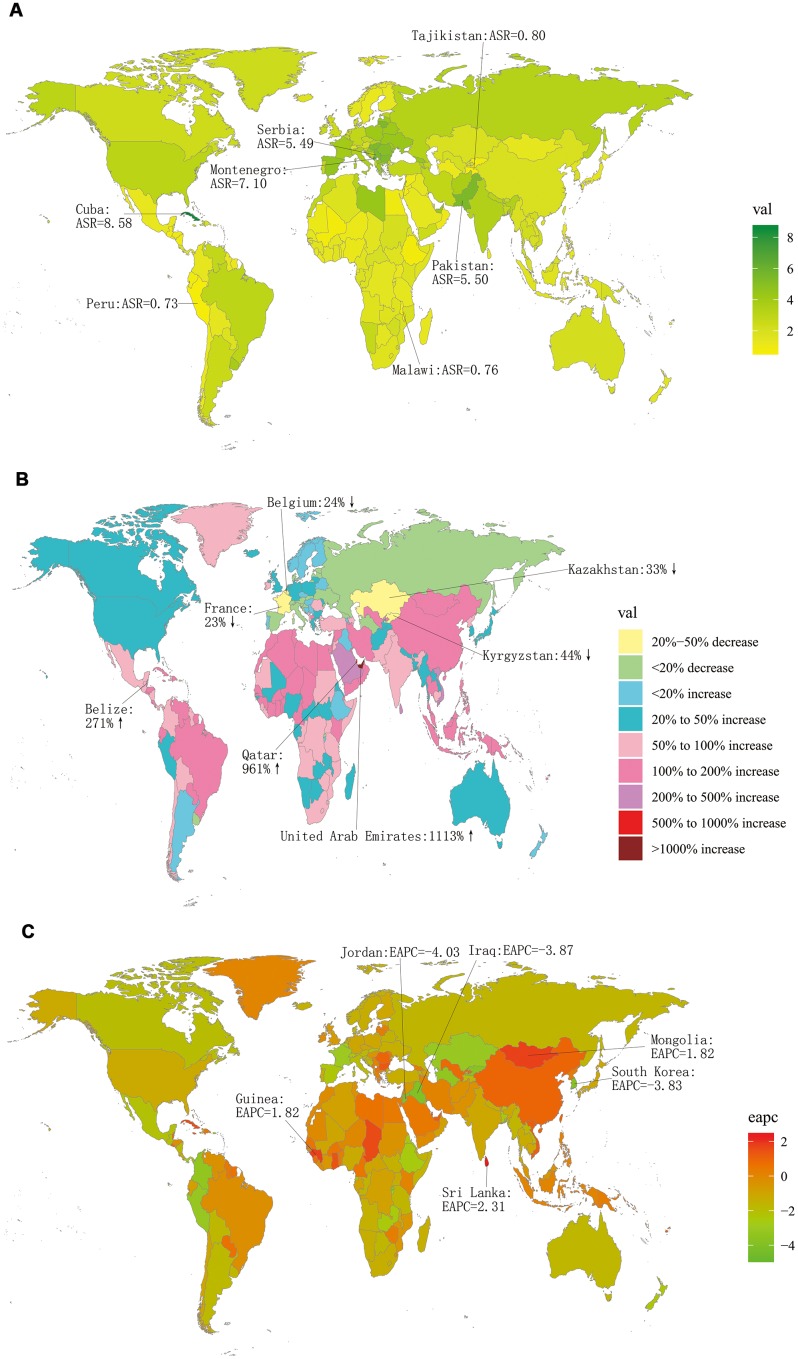
**The global incidence burden of larynx cancer in 195 countries.** (**A**) The ASIR of larynx cancer in 2017; (**B**) The relative change in incident cases of larynx cancer between 1990 and 2017; (**C**) The EAPC of larynx cancer ASIR from 1990 to 2017. Countries with an extreme number of cases/evolution were annotated. ASIR, age-standardized incidence rate; EAPC, estimated annual percentage change.

**Figure 2:**
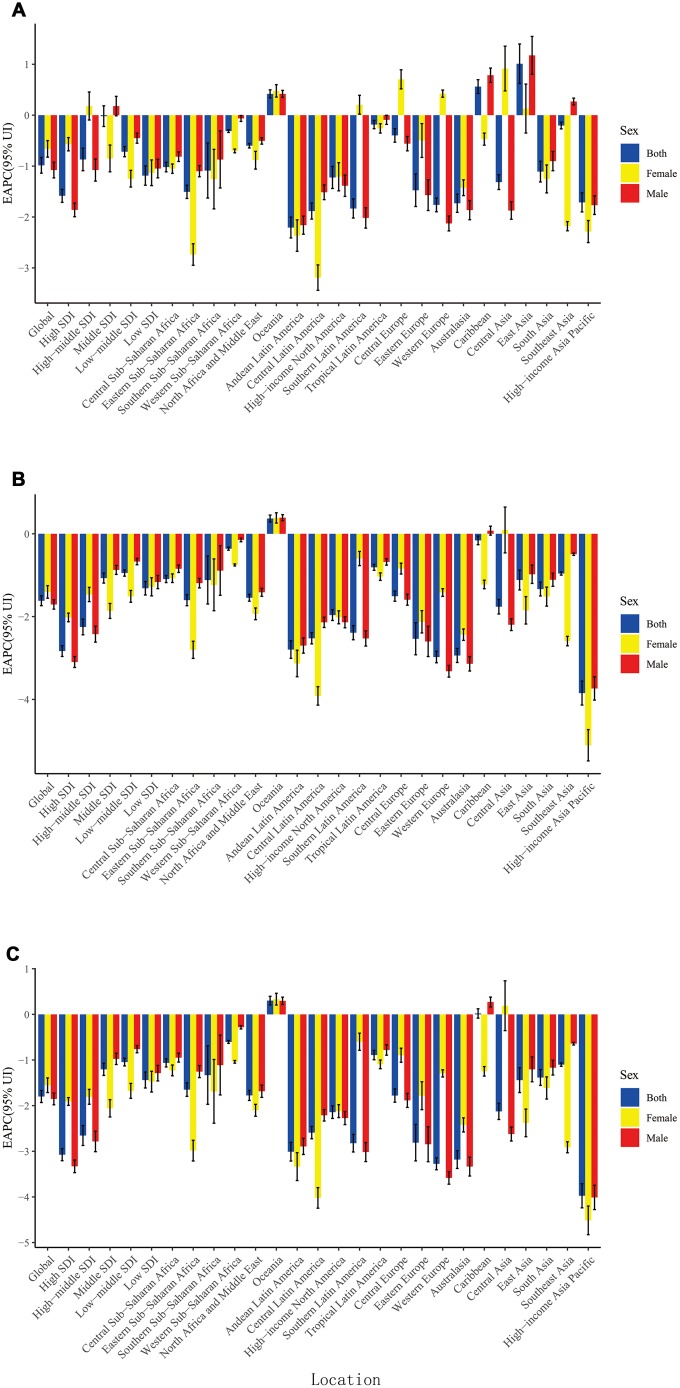
**The EAPC of larynx cancer ASR from 1990 to 2017, by sex and region.** (**A**) The EAPC of ASIR; (**B**) The EAPC of ASDR; (**C**) The EAPC of age-standardized DALY rate. ASR: age-standardized rate; ASDR: age standardized death rate; ASIR: age standardized incidence rate; EAPC, estimated annual percentage change; DALY: disability adjusted life-year.

**Figure 3:**
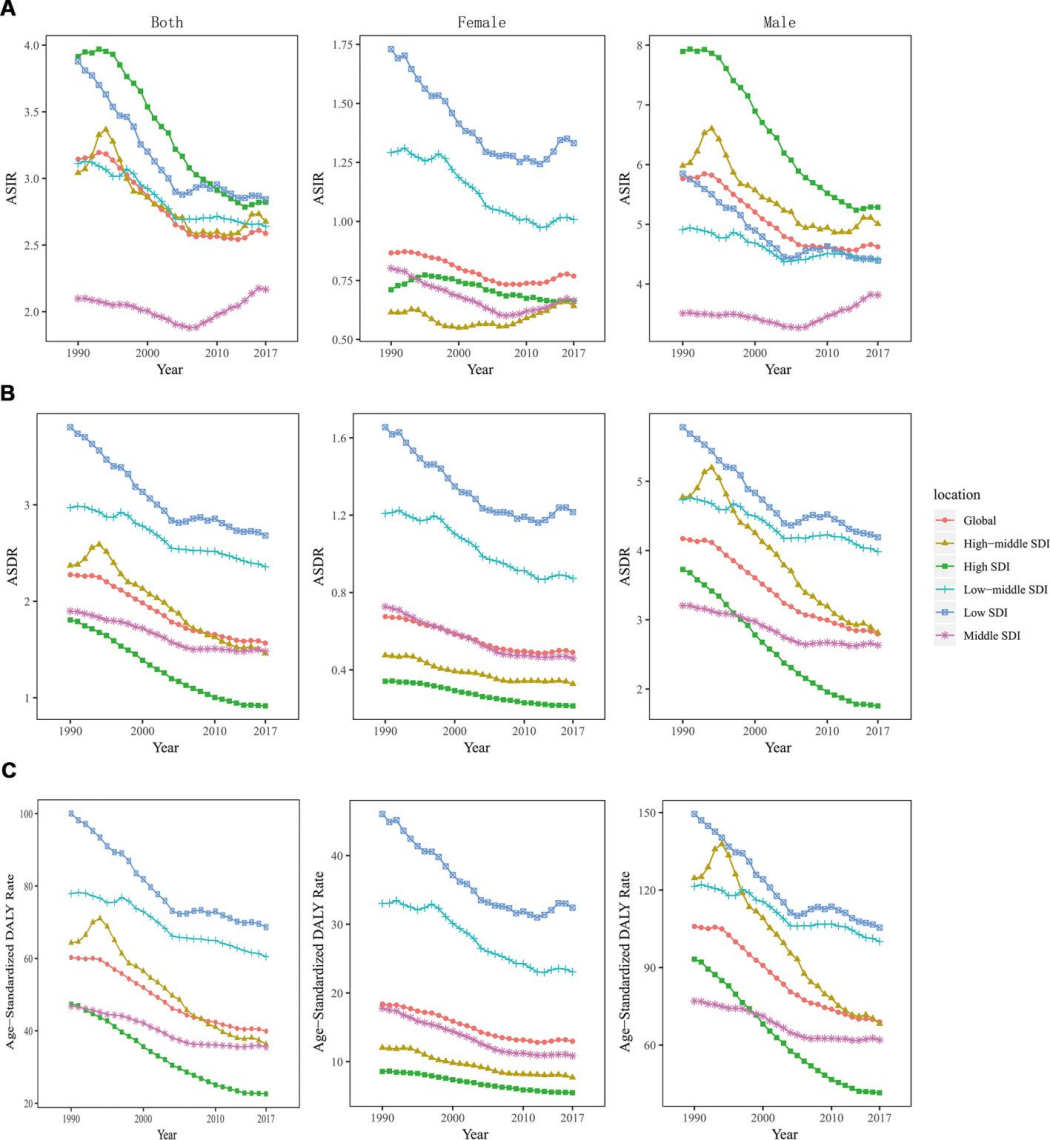
**The change trends of age standardized rate among different SDI quintiles and gender from 1990 to 2017.** (**A**) ASIR: age standardized incidence rate; (**B**) ASDR: age standardized death rate; (**C**) age-standardized DALY rate. DALY, disability adjusted life-year.

**Figure 4:**
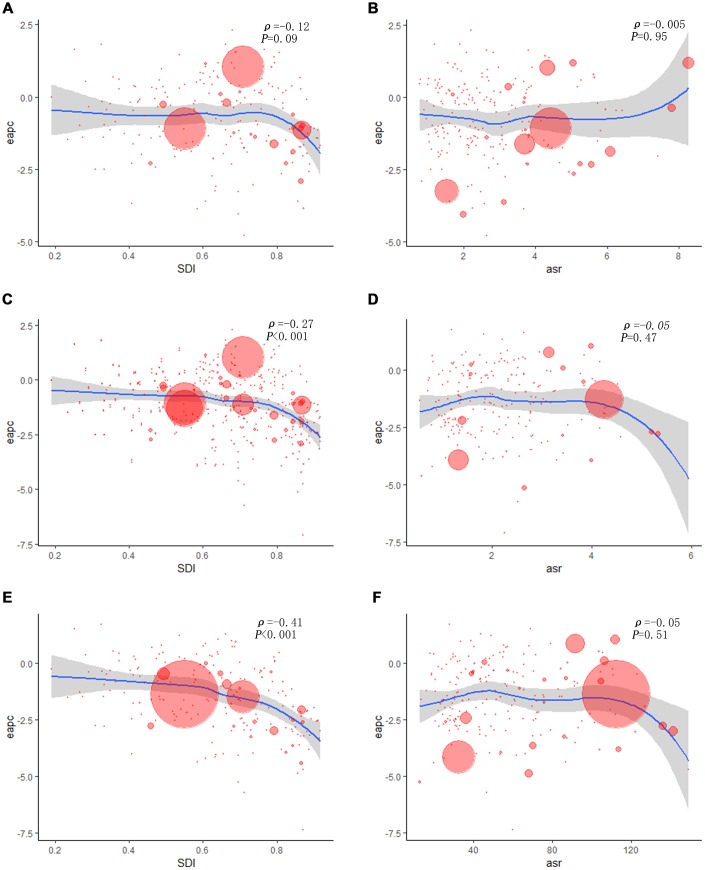
**The correlation between EAPC and larynx cancer ASR in 1990 as well as SDI in 2017.** The circles represent countries that were available on SDI data. The size of circle is increased with the cases of larynx cancer. The *ρ* indices and *P* values presented were derived from Pearson correlation analysis. (**A**) EAPC and SDI in incidence; (**B**) EAPC and ASIR; (**C**) EAPC and SDI in death; (**D**) EAPC and ASDR; (**E**) EAPC and SDI in DALYs; (**F**) EAPC and age-standardized DALY rate. ASIR, age standardized incidence rate; ASDR: age standardized death rate; EAPC, estimated annual percentage change; SDI, socio-demographic index; DALY: disability adjusted life-year.

More LC incident cases were observed in Hungary (39,748.99), China (39,725.40), and USA (17,048.53), whereas fewer LC incident cases were in American Samoa (0.68), Kazakhstan (0.70), and Mali (0.79) (Supplementary Table 1). Cuba had the highest ASIR (8.58/100,000 people), whereas Samoa had the lowest (0.68/100,000 people) (Figure 1). From 1990 to 2017, LC incidence increased in a total of 140 countries. EAPCs were less than zero in 136 countries among female in 129 countries among male (Supplementary Figures 1, 2 and Supplementary Table 3). As shown in Table 1, the greatest increase of ASIR was observed in East Asia (190.73%), whereas the greatest decrease of ASIR was observed in Andean Latin America (-2.21) (Supplementary Table 2). Incident cases of LC showed a rising trend in 19 regions among male, excluding Western and Eastern Europe regions (Supplementary Figure 3A). Only in Eastern Europe, LC incident cases showed a downward trend among female (-2.71%). Meanwhile, the increase of ASIR was highest in East Asia (1.01), while it was lowest in Andean Latin America (-2.21) (Figure 2A). In all the SDI quintiles, LC incident cases increased whereas ASIRs decreased (Supplementary Figures 4–6). The middle SDI quantile showed the highest increase in LC incident cases, whereas the high SDI quintile showed the lowest increase (Table 1). It is worth noting that, in 1995, ASIR showed a sharp increase among male in the high-middle SDI quintile. In 2013, ASIR showed an upward trend in most SDI quintiles, except for the high SDI quintile; then, it decreased since 2016. The specific trends of ASIR over 28 years are presented in Figure 3A. In addition, there was no significant association between EAPC and SDI (ρ = -0.12, P = 0.09, Figure 4A and Supplementary Figure 7A), or between EAPC and ASIR (ρ = -0.005, P = 0.95, Figure 4B).

### Analysis of larynx cancer death worldwide

Over the past 28 years, global deaths due to LC increased by 33.84%, from 94,490 in 1990 to 126,470 in 2017. In contrast, the ASDR decreased worldwide, with an EAPC of -1.62 (-1.74- -1.50). Similar to incidence, most of LC deaths occurred in males (105,610 deaths); which was 5-fold higher than that in females (Table 2). Consistent with deaths, the ASDR in males (2.8/100,000 people) was 5-fold higher than that in females (0.49/100,000 people).

**Table 2:** The death of larynx cancer in 1990 and 2017, and their temporal trends from 1990 to 2017.

**Characteristics**	**1990**		**2017**		**1990-2017**	
	**Deaths No. ×10^3^ (95% UI)**	**ASDR per 100,000 No. (95% UI)**	**Deaths No. ×10^3^ (95% UI)**	**ASDR per 100,000 No. (95% UI)**	**Change in Death Number No. (%)**	**EAPC No. (95% CI)**
Global	94.49 (91.50 - 97.15)	1.75 (1.70 - 1.80)	126.47 (123.38 – 129.85)	1.57 (1.53 - 1.61)	33.84	-1.62 (-1.74 - -1.50)
**Sex**						
Male	79.54 (76.65 - 82.05)	2.93 (2.82 - 3.02)	105.61 (102.69 – 108.75)	2.80 (2.72 - 2.88)	32.78	-1.71 (-1.82 - -1.59)
Female	14.96 (14.23 - 15.81)	0.56 (0.53 - 0.59)	20.86 (19.99 - 21.70)	0.49 (0.47 - 0.51)	39.47	-1.40 (-1.56 - -1.25)
**Socio-demographic index**						
High SDI	22.91 (22.65 - 23.37)	1.81 (1.79 - 1.84)	19.75 (19.28 - 20.28)	0.92 (0.89 - 0.94)	-13.82	-2.83 (-2.96 - -2.70)
High-middle SDI	23.01 (22.54 - 23.89)	2.37 (2.32 - 2.46)	26.57 (25.90 - 27.31)	1.46 (1.42 - 1.50)	15.48	-2.25 (-2.44 - -2.06)
Middle SDI	18.02 (17.33 - 18.95)	1.90 (1.83 – 2.00)	32.55 (31.24 - 34.77)	1.48 (1.42 - 1.58)	80.60	-1.07 (-1.19 - -0.95)
Low-middle SDI	17.30 (15.67 - 18.45)	2.97 (2.70 - 3.17)	28.19 (26.47 - 30.19)	2.36 (2.22 - 2.51)	62.98	-0.95 (-1.02 - -0.87)
Low SDI	13.00 (11.17 - 14.49)	3.80 (3.29 - 4.22)	18.99 (17.32 - 20.51)	2.68 (2.44 - 2.89)	46.12	-1.32 (-1.48 - -1.15)
**Region**						
Andean Latin America	0.29 (0.27 - 0.32)	1.44 (1.34 - 1.59)	0.40 (0.36 - 0.44)	0.75 (0.68 - 0.83)	34.86	-2.80 (-3.01 - -2.59)
Australasia	0.29 (0.28 - 0.30)	1.20 (1.16 - 1.25)	0.29 (0.26 - 0.33)	0.6 (0.54 - 0.68)	1.44	-2.94 (-3.11 - -2.77)
Caribbean	0.82 (0.78 - 0.87)	3.12 (2.98 - 3.33)	1.54 (1.40 - 1.69)	3.02 (2.74 - 3.31)	88.62	-0.16 (-0.26 - -0.05)
Central Asia	1.25 (1.21 - 1.28)	2.47 (2.40 - 2.54)	1.24 (1.17 - 1.31)	1.64 (1.55 - 1.73)	-0.75	-1.76 (-1.94 - -1.58)
Central Europe	5.34 (5.22 - 5.48)	3.48 (3.40 - 3.57)	5.01 (4.81 - 5.22)	2.44 (2.34 - 2.54)	-6.10	-1.51 (-1.63 - -1.38)
Central Latin America	1.60 (1.57 - 1.66)	1.96 (1.92 - 2.02)	2.53 (2.37 - 2.67)	1.11 (1.05 - 1.17)	57.94	-2.52 (-2.66 - -2.39)
Central Sub-Saharan Africa	0.45 (0.36 - 0.54)	2.00 (1.65 - 2.36)	0.77 (0.63 - 0.91)	1.53 (1.29 - 1.81)	70.23	-1.09 (-1.18 - -1.01)
East Asia	11.95 (11.49 - 12.59)	1.33 (1.28 - 1.40)	20.43 (19.56 - 21.36)	1.00 (0.96 - 1.05)	70.91	-1.12 (-1.36 - -0.88)
Eastern Europe	9.37 (9.10 - 9.72)	3.20 (3.11 - 3.32)	6.88 (6.67 - 7.09)	2.02 (1.96 - 2.08)	-26.63	-2.54 (-2.92 - -2.15)
Eastern Sub-Saharan Africa	1.50 (1.32 - 1.70)	1.89 (1.68 - 2.12)	2.16 (1.93 - 2.43)	1.33 (1.18 - 1.5)	43.37	-1.60 (-1.74 - -1.47)
High-income Asia Pacific	1.72 (1.68 - 1.77)	0.84 (0.82 - 0.87)	1.69 (1.62 - 1.77)	0.36 (0.35 - 0.38)	-1.45	-3.85 (-4.14 - -3.56)
High-income North America	4.96 (4.90 - 5.06)	1.41 (1.39 - 1.44)	5.66 (5.48 - 5.83)	0.92 (0.90 - 0.95)	14.04	-1.96 (-2.09 - -1.83)
North Africa and Middle East	4.28 (3.94 - 4.72)	2.46 (2.26 - 2.74)	6.77 (6.34 - 7.14)	1.66 (1.56 - 1.74)	58.18	-1.54 (-1.63 - -1.46)
Oceania	0.05 (0.05 - 0.06)	1.79 (1.58 - 2.02)	0.12 (0.10 - 0.14)	1.88 (1.65 - 2.18)	129.38	0.37 (0.28 - 0.45)
South Asia	27.55 (24.68 - 29.68)	4.45 (3.99 - 4.8)	44.26 (41.78 - 46.79)	3.22 (3.04 - 3.40)	60.63	-1.33 (-1.5 - -1.17)
Southeast Asia	4.56 (4.20 - 5.16)	1.76 (1.63 - 1.99)	8.01 (7.31 - 9.78)	1.38 (1.27 - 1.68)	75.70	-0.96 (-1.00 - -0.92)
Southern Latin America	1.25 (1.20 - 1.29)	2.62 (2.53 - 2.71)	1.21 (1.09 - 1.35)	1.48 (1.33 - 1.64)	-2.82	-2.39 (-2.56 - -2.22)
Southern Sub-Saharan Africa	0.60 (0.54 - 0.71)	2.08 (1.89 - 2.48)	0.91 (0.86 - 0.96)	1.63 (1.54 - 1.71)	52.49	-1.12 (-1.70 - -0.54)
Tropical Latin America	2.47 (2.42 - 2.55)	2.62 (2.56 - 2.70)	5.08 (4.95 - 5.23)	2.16 (2.10 - 2.22)	105.86	-0.81 (-0.88 - -0.74)
Western Europe	12.89 (12.68 - 13.21)	2.27 (2.23 - 2.33)	9.26 (8.89 - 9.66)	1.08 (1.03 - 1.13)	-28.18	-2.98 (-3.12 - -2.84)
Western Sub-Saharan Africa	1.30 (1.09 - 1.56)	1.44 (1.22 - 1.71)	2.26 (1.93 - 2.68)	1.29 (1.11 - 1.52)	73.68	-0.36 (-0.40 - -0.32)

ASDR: age standardized death rate; EAPC: estimated annual percentage change; CI: confidence interval; UI: uncertainty interval; SDI: socio-demographic index

Supplementary Table 1 displays the top three countries with high and low number of death and ASDR. The countries with the highest and lowest ASDR were Panama (5.17/100,000 people) and Kazakhstan (0.32/100,000 people), respectively (Supplementary Figure 8). From 1990 to 2017, LC death showed a decreasing trend in 40-50 countries, and ASDR decreased in most countries (females: 168 countries; males: 151 countries) (Supplementary Figures 9–10 and Supplementary Table 4). The number of LC deaths increased in 15 regions. As for ASDR, only in Oceania, the ASDR increased, with an EAPC of 0.37 (0.28- 0.45). Besides, LC deaths showed an upward trend among males in 16 regions (Supplementary Figure 3B); however, a downward trend was observed in High-income Asia Pacific (-29.86%), Eastern Europe (-29.01%), and Western Europe (-1.91%) (Supplementary Table 2). ASDR increased the fastest in Oceania (0.37), whereas it increased the slowest in High-income Asia Pacific (-3.85) (Figure 2B). Compared to 1990, LC deaths increased in most SDI quintiles in 2017 (Supplementary Figures 11–13), except for the high SDI quintile (decreased by 13.82%). And it increased the fastest in the middle SDI quintile (80.60%). The ASIRs among different SDI quintiles in the past 28 years are shown in Figure 3B. EAPC was positively correlated with SDI (ρ=-0.27, P<0.001, Figure 4C and Supplementary Figure 7B), but was not correlated with ASDR (ρ=-0.05, P=0.47, Figure 4D).

### Analysis of larynx cancer DALYs worldwide

DALYs of LC were 3,279,460 (3,191,430-3,375,120) years in 2017, which is 1.25-fold higher than that in 1990. However, the age-standardized DALY rate showed a downward trend, with an EAPC of -1.8 (-1.93- -1.67) (Table 3). Both DALYs and age-standardized DALY rates were higher in males, which were 5-fold higher than those in females.

**Table 3:** The DALYs of larynx cancer, and their temporal trends from 1990 to 2017.

**Characteristics**	**1990**		**2017**		**1990-2017**	
	**DALY No. ×10^3^ (95% UI)**	**Age Standardized DALY Rate (per 100,000) No. (95% UI)**	**DALY No. ×10^3^ (95% UI)**	**Age Standardized DALY Rate (per 100,000) No. (95% UI)**	**Change in DALYs Number No. (%)**	**EAPC No. (95% CI)**
Global	2618.75 (2527.72 - 2697.72)	60.26 (58.16 - 62.06)	3279.46 (3191.43 - 3375.12)	39.89 (38.82 - 41.05)	25.23	-1.8 (-1.93 - -1.67)
**Sex**						
Male	2200.03 (2110.29 - 2275.19)	105.93 (101.72 - 109.49)	2732.93 (2650.74 - 2817.19)	68.97 (66.89 - 71.10)	24.22	-1.85 (-1.98 - -1.73)
Female	418.72 (396.99 - 442.34)	18.38 (17.42 - 19.42)	546.53 (522.78 - 570.39)	12.97 (12.4 - 13.54)	30.52	-1.55 (-1.71 - -1.39)
**Socio-demographic index**						
High SDI	578.98 (568.21 - 592.16)	47.38 (46.51 - 48.47)	445.77 (430.67 - 461.58)	22.59 (21.84 - 23.41)	-23.01	-3.07 (-3.21 - -2.94)
High-middle SDI	650.39 (636.05 - 674.10)	64.32 (62.93 - 66.71)	677.61 (659.41 - 697.58)	36.32 (35.35 - 37.39)	4.18	-2.65 (-2.87 - -2.44)
Middle SDI	498.16 (478.48 - 523.76)	46.83 (45.02 - 49.23)	827.46 (791.22 - 891.84)	35.51 (33.99 - 38.24)	66.11	-1.20 (-1.34 - -1.07)
Low-middle SDI	502.44 (453.79 - 536.35)	77.90 (70.54 - 83.13)	785.51 (734.75 - 843.69)	60.49 (56.63 - 64.84)	56.34	-1.05 (-1.13 - -0.96)
Low SDI	382.02 (327.71 - 426.66)	100.03 (85.91 - 111.5)	532.99 (485.24 - 577.35)	68.64 (62.57 - 74.19)	39.52	-1.44 (-1.61 - -1.27)
**Region**						
Andean Latin America	7.47 (6.93 - 8.17)	32.88 (30.49 - 36.02)	8.72 (7.89 - 9.67)	15.97 (14.45 - 17.71)	16.80	-3.01 (-3.21 - -2.8)
Australasia	7.00 (6.71 - 7.33)	29.77 (28.50 - 31.19)	6.30 (5.58 - 7.12)	14.02 (12.43 - 15.86)	-9.99	-3.18 (-3.38 - -2.99)
Caribbean	19.25 (18.28 - 20.67)	71.14 (67.59 - 76.50)	36.56 (32.95 - 40.31)	71.27 (64.26 - 78.58)	89.97	0.02 (- 0.08 - 0.12)
Central Asia	36.53 (35.44 - 37.71)	69.44 (67.43 - 71.55)		42.28 (39.97 - 44.60)	-4.60	-2.13 (-2.30 - -1.95)
Central Europe	151.03 (147.61 - 155.10)	99.22 (96.99 - 101.87)	127.34 (121.91 - 133.04)	65.76 (62.96 - 68.67)	-15.68	-1.78 (-1.92 - -1.63)
Central Latin America	38.51 (37.71 - 39.74)	42.64 (41.73 - 44.01)	56.48 (52.93 - 59.85)	23.92 (22.42 - 25.31)	46.69	-2.59 (-2.73 - -2.46)
Central Sub-Saharan Africa	12.86 (9.99 - 15.69)	48.93 (38.81 - 58.93)	34.85 (32.80 - 36.90)	37.97 (30.98 - 45.22)	72.70	-1.06 (-1.15 - -0.97)
East Asia	324.51 (311.19 - 341.08)	32.26 (30.95 - 33.93)	489.64 (465.71 - 513.79)	22.91 (21.82 - 24.02)	50.88	-1.44 (-1.71- -1.17)
Eastern Europe	276.31 (267.33 - 288.23)	95.58 (92.59 - 99.90)	187.82 (181.83 - 194.25)	56.86 (55.05 - 58.84)	-32.03	-2.81 (-3.21- -2.41)
Eastern Sub-Saharan Africa	45.00 (39.00 - 51.40)	50.44 (44.12 - 57.17)	64.32 (57.67 - 71.76)	35.07 (31.37 - 39.34)	42.94	-1.65 (-1.80 - -1.50)
High-income Asia Pacific	41.09 (39.80 - 42.65)	19.44 (18.84 - 20.17)	31.88 (30.14 - 33.84)	7.98 (7.54 - 8.49)	-22.42	-3.97 (-4.24 - -3.71)
High-income North America	120.00 (117.43 - 123.18)	35.99 (35.19 - 36.96)	131.88 (126.52 - 137.04)	22.65 (21.72 - 23.55)	9.90	-2.14 (-2.28 - -2.00)
North Africa and Middle East	119.18 (109.80 - 129.55)	61.28 (56.37 – 67.00)	179.67 (168.03 - 190.51)	39.23 (36.66 - 41.46)	50.76	-1.78 (-1.89 - -1.66)
Oceania	1.65 (1.42 - 1.94)	45.66 (39.71 - 52.86)	3.74 (3.16 - 4.46)	46.86 (40.29 - 54.90)	126.74	0.30 (0.21 - 0.40)
South Asia	812.14 (727.17 - 874.66)	117.24 (104.91 - 126.43)	1236.89 (1170.38 - 1309.34)	83.63 (78.97 - 88.53)	52.30	-1.38 (-1.56 - -1.21)
Southeast Asia	127.24 (117.40 - 144.71)	43.66 (40.37 - 49.54)	209.25 (189.65 - 256.62)	32.94 (29.98 - 40.34)	64.45	-1.10 (-1.14 - -1.07)
Southern Latin America	32.60 (31.33 - 33.82)	68.00 (65.36 - 70.61)	27.82 (24.84 - 31.22)	34.77 (31.10 - 39.01)	-14.64	-2.82 (-3.02 - -2.63)
Southern Sub-Saharan Africa	17.30 (15.70 - 20.14)	55.99 (50.72 - 65.82)	25.06 (23.56 - 26.72)	41.66 (39.23 - 44.26)	44.81	-1.33 (-1.97 - -0.69)
Tropical Latin America	68.67 (66.99 - 70.86)	66.96 (65.39 - 69.16)	132.27 (128.63 - 136.5)	54.49 (53.00 - 56.24)	92.62	-0.89 (-1.00 - -0.79)
Western Europe	324.88 (317.66 - 334.08)	60.82 (59.48 - 62.56)	205.57 (196.09 - 216.03)	26.84 (25.56 - 28.24)	-36.72	-3.27 (-3.41 - -3.14)
Western Sub-Saharan Africa	35.55 (29.40 - 43.21)	36.05 (30.03 - 43.48)	61.19 (51.78 - 73.34)	30.54 (26.04 - 36.28)	72.10	-0.60 (-0.64 - -0.57)

DALY: disability adjusted life-year; EAPC: estimated annual percentage change; CI: confidence interval; UI: uncertainty interval; SDI: socio-demographic index

The top three countries with high DALYs were the same as deaths (Supplementary Table 1). DALYs were low in American Samoa (12.09), Samoa (17.91), and Kiribati (18.88) (Supplementary Figure 14). The values of EAPC were negative among female in 172 countries and among males in 154 countries (Supplementary Figures 15–16 and Supplementary Table 5). DALYs of LC decreased in 7 regions (Supplementary Figure 3C). It is remarkable that the age-standardized DALY rate increased only in Oceania, with an EAPC of 0.3 (0.21- 0.4). In 2017, DALYs of LC were higher in South and East Asia, and were lower in Oceania, Australasia, and Andean Latin America (Supplementary Table 2). Age-standardized DALY rate increased obviously in Oceania (0.30), and it decreased significantly in High-income Asia Pacific (-3.97) (Figure 2C). Only in high SDI quintile, DALYs of LC decreased by 23.01% (Supplementary Figures 17–19). Analogously, the largest increase of LC DALYs was observed in the middle SDI quintile (66.11%). The change trends of ASIR in the past 28 years are presented in Figure 3C. EAPC was positively correlated with SDI (*ρ*= -0.41, *P*< 0.001, Figure 4E and Supplementary Figures 7C), but not correlated with age-standardized DALY rate (*ρ*= -0.0.05, *P*= 0.51, Figure 4F).

### Age distribution of incidence, deaths and DALYs of larynx cancer

**Figure 5:**
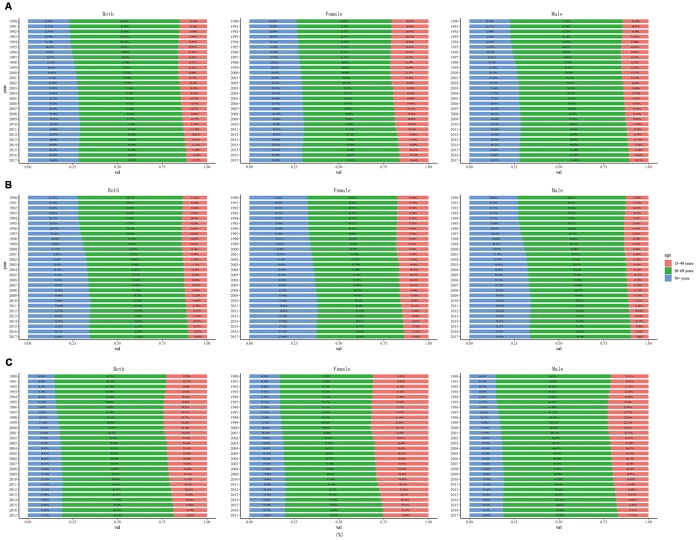
**The proportion of different age groups in larynx cancer by years.** (**A**) incidence, (**B**) death, (**C**) DALY. DALY: disability adjusted life-year.

From 1990 to 2017, in all regions, LC incidence, death and DALYs were mainly concentrated in age group of 50-69 years, followed by those aged over 70 years (Figure 5). In addition, in both female and male, these parameters showed upward trends over time in people aged over 70 years, while significantly downward trends were observed in people aged 15-49 years. A dynamic equilibrium was observed over time in people aged 50-69 years.

**Figure 6:**
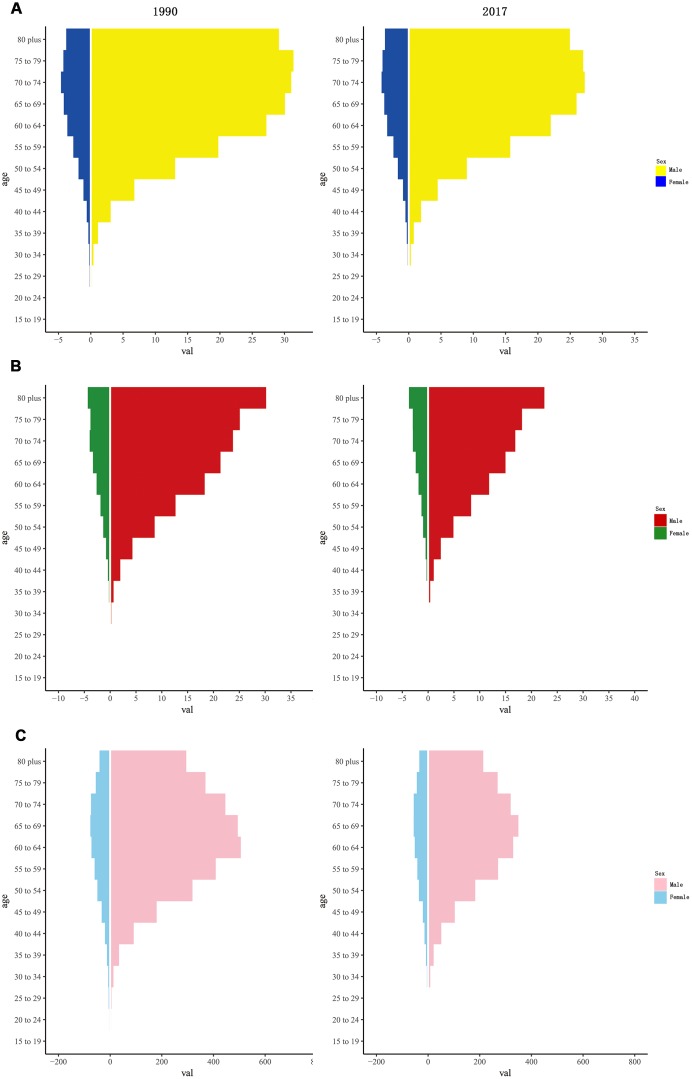
**The rate of larynx cancer among gender and age in 1990 and 2017.** (**A**) incidence rate; (**B**) death rate; (**C**) DALY rate. DALY, disability adjusted life-year.

Both in1990 and 2017, the incidence rate of LC showed unimodal distribution among different age groups, with peak at 60-80 years in all SDI quintiles. The death rate of LC tended to increase gradually with age. DALY rate also showed a unimodal distribution in different gender, with a peak at 65-80 years among females and 60-75 years among males (Figure 6 and Supplementary Figures 20–24).

## DISCUSSION

Our analysis based on the GBD study revealed the latest worldwide patterns and trends of incidence, mortality, and DALYs of LC. Compared to 1990, the incident cases increased by 58.67%, deaths of LC increased by 33.84%, and DALYs increased 1.25-fold globally in 2017. On the contrary, the ASIRs, ASDRs and age-standardized DALY rates of LC all showed downward trends. The LC burden is mainly concentrated in male worldwide [2, 4, 16], which might be attributable to long-term tobacco exposure. From 1990 to 1994, the incident cases, deaths, and DALYs of LC showed a continuous upward trend, after which they held steady between 1995 and 2006. Remarkably, both among female and male, LC burden increased steadily since 2007. These findings will be helpful for rational allocation and planning of resources assigned for health services. The number of patients requiring the care of a laryngeal cancer specialist will continue to grow in the coming decades. As the world’s population grows and ages, a greater burden will be imposed.

The most commonly known and well-established risk factor of LC is tobacco use [17–19]. A study in Algeria by Kariche et al. found that the etiological rate of HPV was low among LC patients, whereas tobacco and alcohol remained the major etiological risks [20]. Hashibe et al. concluded that head and neck cancer has a dose-response relationship with duration, frequency, and number of pack-years of smoking. Furthermore, only high alcohol consumption increased the risk of LC [21, 22]. However, a study on Australia data showed that stopping alcohol consumption could prevent 4% of alcohol-related cancers over 25 years [23]. Another study concluded that eliminating alcohol consumption reduced about 83,000 cancer cases over 30 years and reduced 5.5% of the expected cases of six alcohol-related cancers, including LC, in Nordic countries [24]. Therefore, the most basic way of reducing LC burden is to prevent exposure to risk contributors in different countries, especially tobacco and alcohol [25]. Improving and implementing comprehensive tobacco and alcohol control policies and monitoring these factors are critical for preventing cancer. In addition, the control of second-hand and third-hand smoke is essential [26–28]. Some countries, such as Singapore and the United Kingdom, have taken measures to control tobacco usage by advertising, prohibiting smoking in public spaces, rising tobacco taxation, and adding warning labels on cigarette packaging [29, 30]. The most important groups of tobacco control advocates include medical staff and local government officials. In many rich countries, deaths due to cardiovascular disease and cancer are decreasing, largely owing to the success of anti-smoking campaigns. Although the related medical costs exceed the tobacco tax, some governments cannot give up the tobacco tax to reduce medical costs, because the medical costs are mainly borne by citizens themselves in case of incomplete medical insurance. In addition, reducing the LC burden through early detection and formal treatment is a major challenge. Methods with high sensitivity and specificity are needed to early diagnosis.

The incidences, deaths and DALYs of LC varied among countries. All the ASRs were high in Cuba, which is significant from the perspective of policy makers. Obviously, the burden of LC in Cuba is very high. Consistent with our results, a previous study in Central and South America showed that Cuba has the highest incidence and mortality of LC, and patients among men were four times more than women [4]. In our analysis, Hungary, China, and USA had high LC incident cases of LC. Besides, India, China, and Pakistan showed high deaths and DALYs of LC. It is reported that Chinese men smoke accounted for more than a third of the world’s cigarettes totally [31]. Chen et al. conducted a nationwide prospective cohort study and concluded that tobacco consumption is on the rise among men, accounting for about 20% of adult male deaths in China [32]. The increase of deaths and DALYs among female was the slowest in South Korea. The ASDRs and age-standardized DALY rates increased rapidly in Guinea, Chad, and Mongolia, whereas they decreased rapidly in South Korea and Bahrain. Tobacco control can reduce the burden of chronic diseases and help solve the problem of limited medical resources and economic development. Most countries have implemented the World Health Organization recommendations on public policy control of tobacco and alcohol, with varying effects. In recent years, smoking rates remained high among Chinese men, similar to that observed decades ago in Poland, the Republic of Korea, and the Russian Federation. In contrast to other countries, China has not made significant progress in tobacco control.

As reported in previous studies, regional disparities also exist in LC [33–35]. Our results indicated that South and East Asia had the heaviest burden of LC. The LC increased significantly in East Asia and Tropical Latin America, whereas it decreased largely in Eastern Europe and Western Europe. The ASIR among male in East Asia increased the fastest, suggesting a large burden of LC possibly resulting from tobacco and alcohol exposure [27, 32, 36]. In less-developed countries, most patients were diagnosed in advanced stages due to medical limitations, resulting in poor response to treatment and poor quality of life [37–40]. ASIRs decreased remarkably in Andean, Central, and Southern Latin America. The EAPCs of ASDR and age-standardized DALY rates were low in High-income Asia Pacific, Western Europe, and Australasia; this may be due to healthier lifestyles and superior social welfare and health care services.

The burden of LC varies widely across different SDI quintiles. Incident cases of LC increased, whereas ASIRs decreased in all SDI quintiles, which is consistent with the global cancer statistics [2, 41]. The high SDI quintile had the lowest ASDR, DALYs, and age-standardized DALY rate, which might be attributed to access to their advanced prevention and treatment technologies. We found that the burden of LC increased in low SDI quintile. The low SDI quintile had the highest ASIR, ASDR, and age-standardized DALY rate, reflecting the undeveloped medical conditions. In Africa, there is a shortage of staff, drugs, funding, screening and early detection services, and delivery models for cancer care [42]. Though global medical technology is improving gradually, huge regional imbalances exist worldwide. Regarding deaths, the middle SDI quintile had highest deaths and DALYs of LC. Deaths and DALYs decreased only in high SDI quintile, but increased the most in middle SDI quintile. ASDR and age-standardized DALY rate decreased in all SDI quintiles.

In addition, an upward trend was observed over time in people aged >70 years, whereas a significant downward trend was observed in 15-49 years. These values might improve with an increase in health awareness and improvement of medical services. LC deaths was 35%–38% among patients aged >70 years, which was higher than that in incidence and DALYs; this might be partly attributed to aging of the population. The ASDR of LC tended to increase gradually with age, which is consistent with the rule of natural death. A study also stated that age > 80 years was related to poor survival in LC patients [38].

This study had some unavoidable limitations. The accuracy of results depended on the quality and quantity of GBD data. In terms of quantity, the method used in the GBD study itself had certain limitations regarding epidemiological evaluation of LC, and the study could not cover all districts worldwide. In terms of quality, the possibility of missing information due to limited specialized medical care, treatment resources, and laboratory investigations in less developed countries cannot be ruled out. In addition, information bias is inevitable. Due to the limitations of data, we cannot do the further investigation on the histological, etiological, and risk stratification of LC.

In conclusion, the incident cases, deaths and DALYs of LC increased worldwide. On the contrary, the ASRs showed a downward trend globally. Most of the larynx cancer burden was observed in males, especially among those who aged 50 -69 years. South and East Asia had the heaviest burden of larynx cancer. All the age-standardized rates were high in Cuba, which deserve further investigation. Based on our change analysis of LC patterns according to sex, age, SDI, region, and country, policy makers can allocate the limited available medical resources more reasonably and can formulate more effective public health prevention and control policies to alleviate the LC burden.

## MATERIALS AND METHODS

### Study data sources

Annual data of incidence, death, and DALYs and the corresponding ASRs of larynx cancer from 1990 to 2017 were collected using the Global Health Data Exchange (GHDx) query tool (http://ghdx.healthdata.org/gbd-results-tool). SDI was defined as geometric average of total fertility, per capita income, and average years of education; SDI ranged from 0 to 1 [43, 44]. We calculated the SDI of each country included in the GBD study 2017 and categorized the countries into five SDI quintiles. This study was conducted in accordance to the recommendations of the Guidelines for Accurate and Transparent Health Estimates Reporting. We assessed the epidemiology of multiple sclerosis from 1990 to 2016. DisMod-MR version 2.1, a Bayesian meta-regression framework, was used in GBD epidemiological modelling.

### Attributable burden

To further analyze the global burden of LC, we selected locations according to three different criteria. To assess the relationship between disease burden and development, we classified these countries and regions into five categories according to SDI: low SDI, low-middle SDI, middle SDI, high-middle SDI, and high SDI. The world was geographically divided into 21 regions to observe differences. In addition, we drew world maps including 195 countries to observe the annual ASRs, change in the number of LC cases, and trend of LC ASRs in different countries in the past 28 years.

### Statistical analysis

ASRs and their estimated annual percentage changes (EAPCs) were calculated to quantify the incidence and mortality trends of LC. DALYs were estimated by summing the years lived with disability and years of life lost [45].

Standardization was essential while comparing several groups with different age structures or the same group with the age distribution changing with time. The ASR (per 100,000 population) was estimated using the following formula, which summed the products of age-specific rates (a_i_, where i denotes the i^th^ age class) and the number of persons (or weight) (w_i_) in the same age subgroup i of the designated reference population, divided by the sum of standard population weights.

$$ASR=\frac{\sum_{i=1}^{A}a_{i}w_{i}}{\sum_{i=1}^{A}w_{i}}\times 100,000$$

Moreover, trends in ASR reflect the alterations of LC. While this information is helpful for formulating more effective prevention strategies, the concept of EAPC has been introduced to describe the trends in ASR within a specified time interval. It is assumed that the natural logarithm of ASR has a linear relationship with time, thus Y=α+βX+ε, where Y refers to ln (ASR), X represents calendar year, and ε means error term. Based on this formula, β determines the positive or negative trends in ASR. EAPC is calculated using EAPC=100*(exp (β)-1); the formulas for calculating EAPC and its 95% confidence interval were obtained from the linear model. It has been shown that when the EAPC and lower limit of confidence interval are positive, ASR shows an upward trend. Conversely, when the EAPC and upper limit of the confidence interval are negative, the ASR shows a descending trend. In addition, we evaluated the relation between EAPCs and ASRs in 1990, SDI in 2017 in different countries, aiming to identify the potential factors affecting EAPCs. All calculations were performed using the R software (version 3.5.1).

## Supplementary Material

Supplementary Figures

Supplementary Table 1

Supplementary Table 2

Supplementary Table 3

Supplementary Table 4

Supplementary Table 5
