# Supplementary material for: Global burden of larynx cancer, 1990-2017: estimates from the global burden of disease 2017 study
Source: Aging (Albany NY). 2020 Feb 8;12(3):2545–83. doi: 10.18632/aging.102762 (PMC7041735; doi:10.18632/aging.102762)
Supplement: Supplementary Figures [file aging-12-102762-s006..pdf]

SUPPLEMENTARY FIGURES

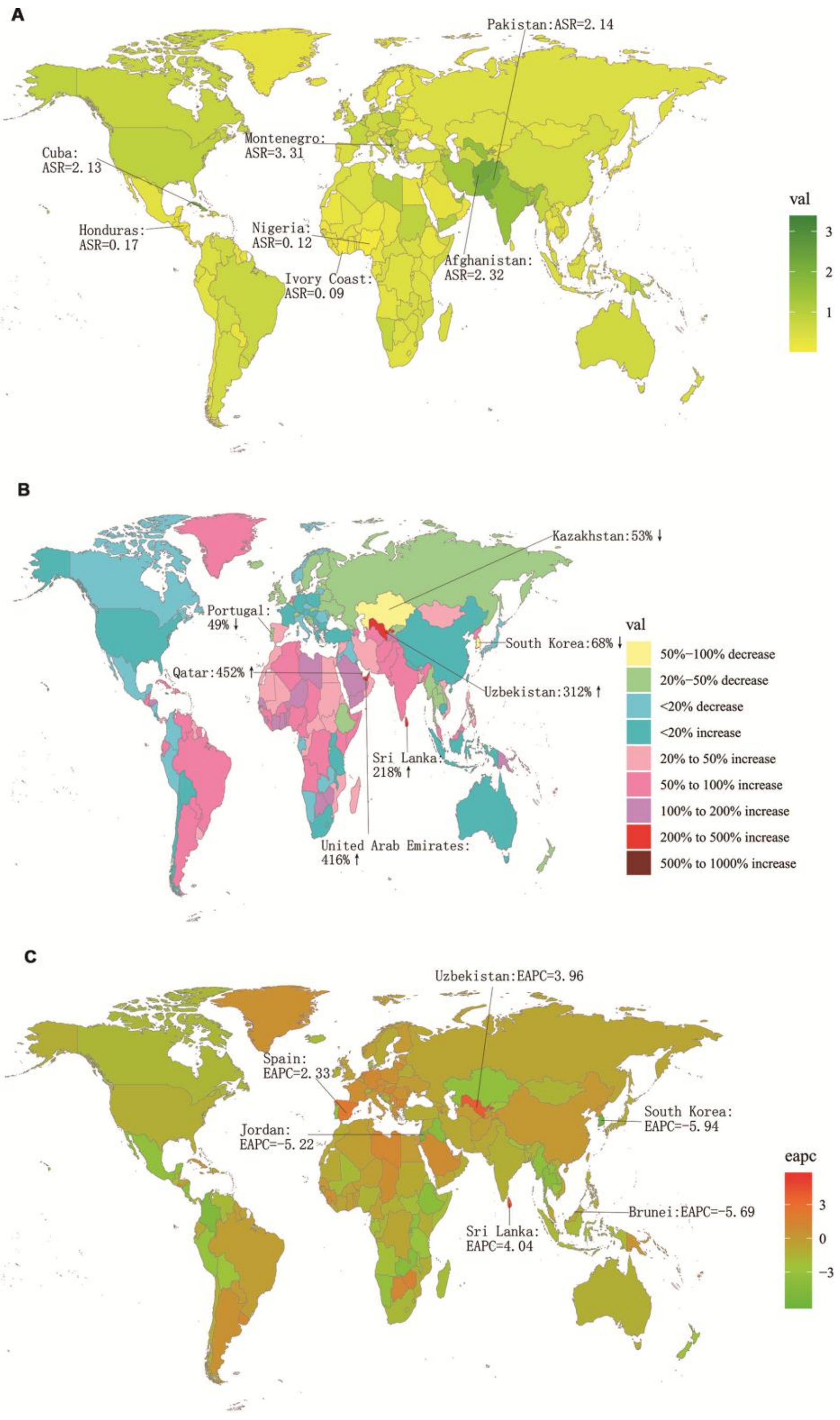

Supplementary Figure 1. The global incidence burden of larynx cancer among female in 195 countries. (**A**) The ASIR of larynx cancer in 2017; (**B**) The relative change in incidences of larynx cancer between 1990 and 2017; (**C**) The EAPC of larynx cancer ASIR from 1990 to 2017. Countries with an extreme number of cases/evolution were annotated. ASIR, age-standardized incidence rate; EAPC, estimated annual percentage change.

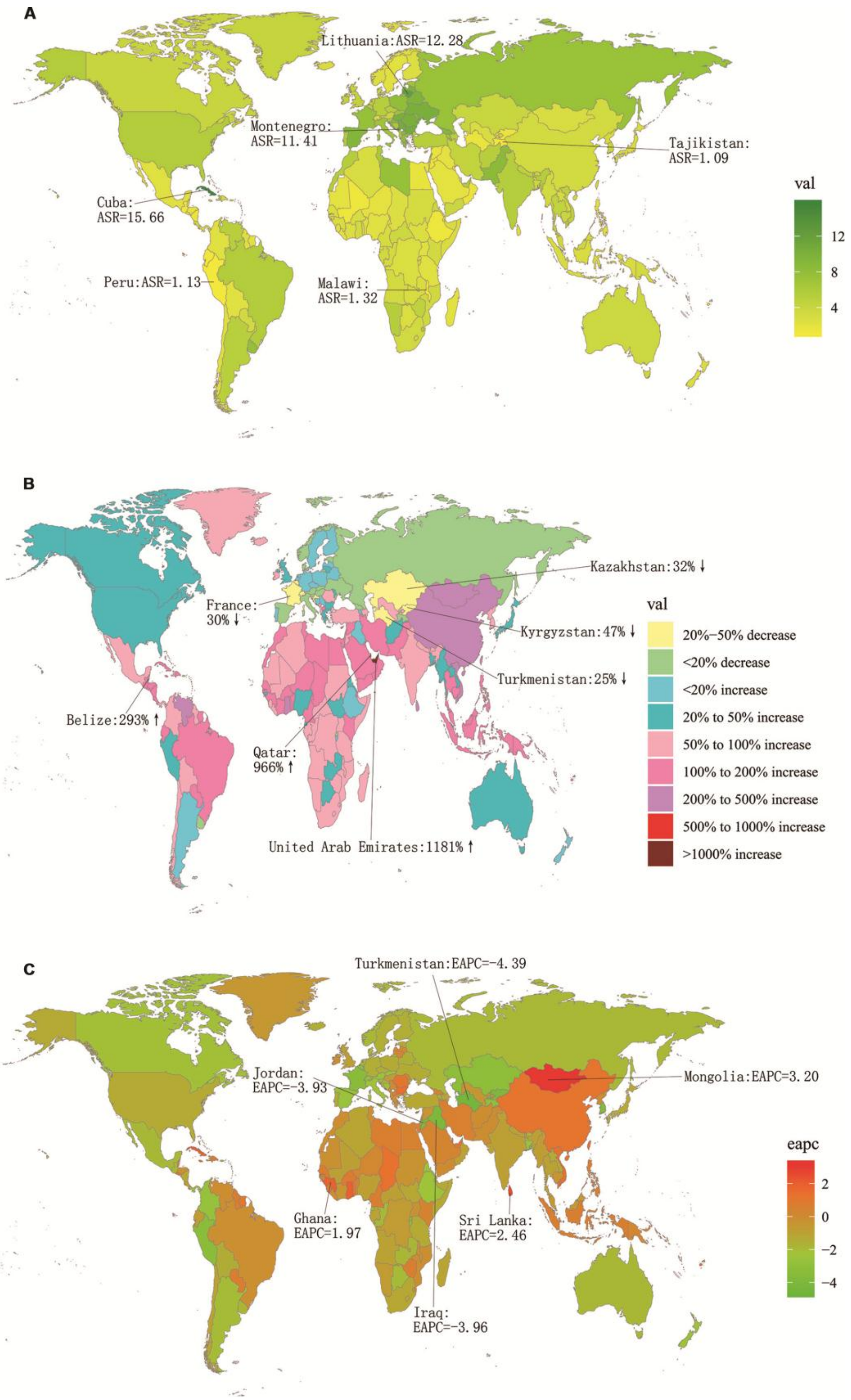

Supplementary Figure 2. The global incidence burden of larynx cancer among male in 195 countries. (**A**) The ASIR of larynx cancer in 2017; (**B**) The relative change in incidences of larynx cancer between 1990 and 2017; (**C**) The EAPC of larynx cancer ASIR from 1990 to 2017. Countries with an extreme number of cases/evolution were annotated. ASIR, age-standardized incidence rate; EAPC, estimated annual percentage change.

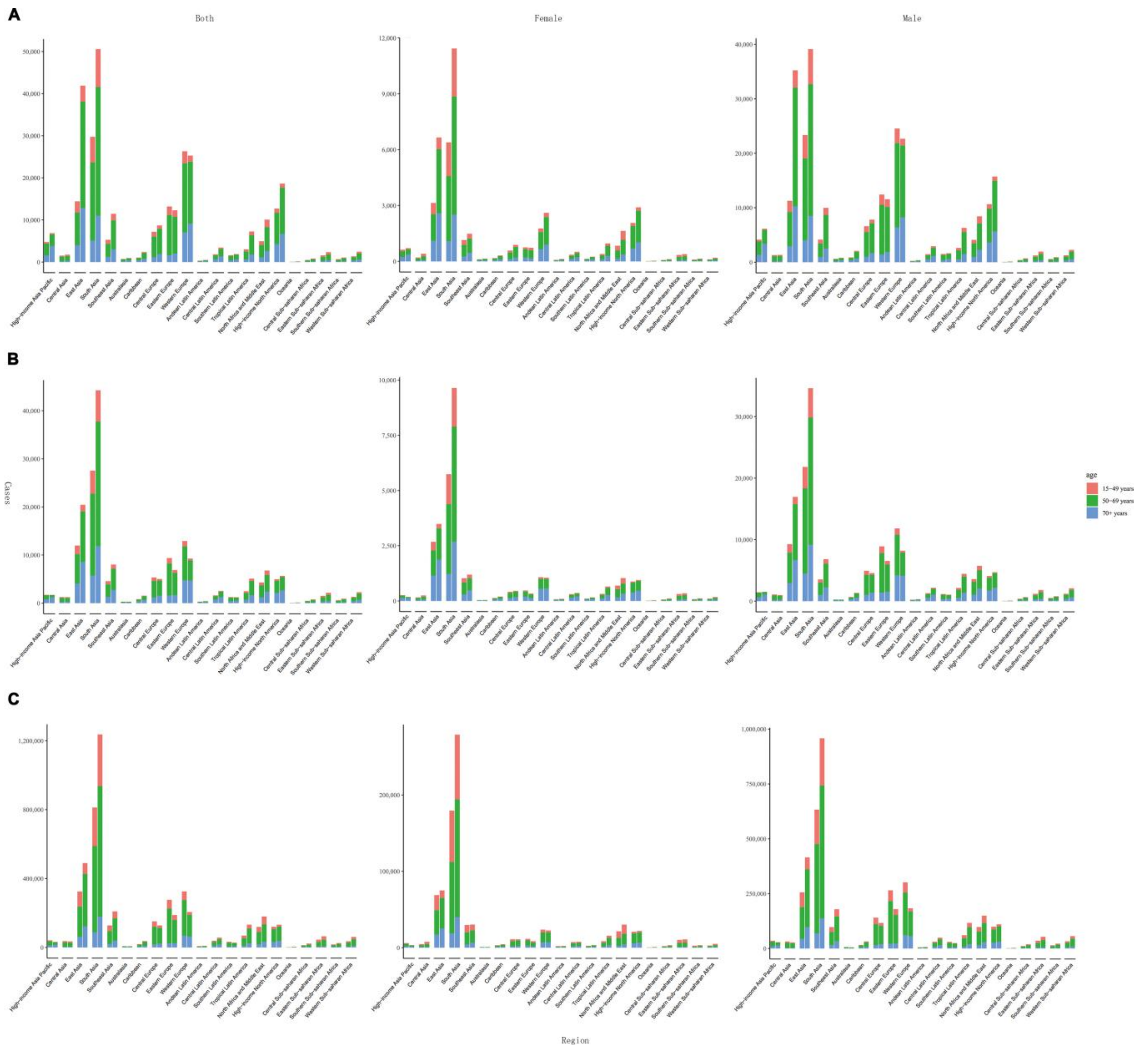

Supplementary Figure 3. The cases of larynx cancer at a regional level by sex and age. The left column in each group is case data in 1990 and the right column in 2017. (**A**) The cases of incidence; (**B**) The cases of death; (**C**) The number of DALYs. DALY: disability adjusted life-year. SDI, socio-demographic index.

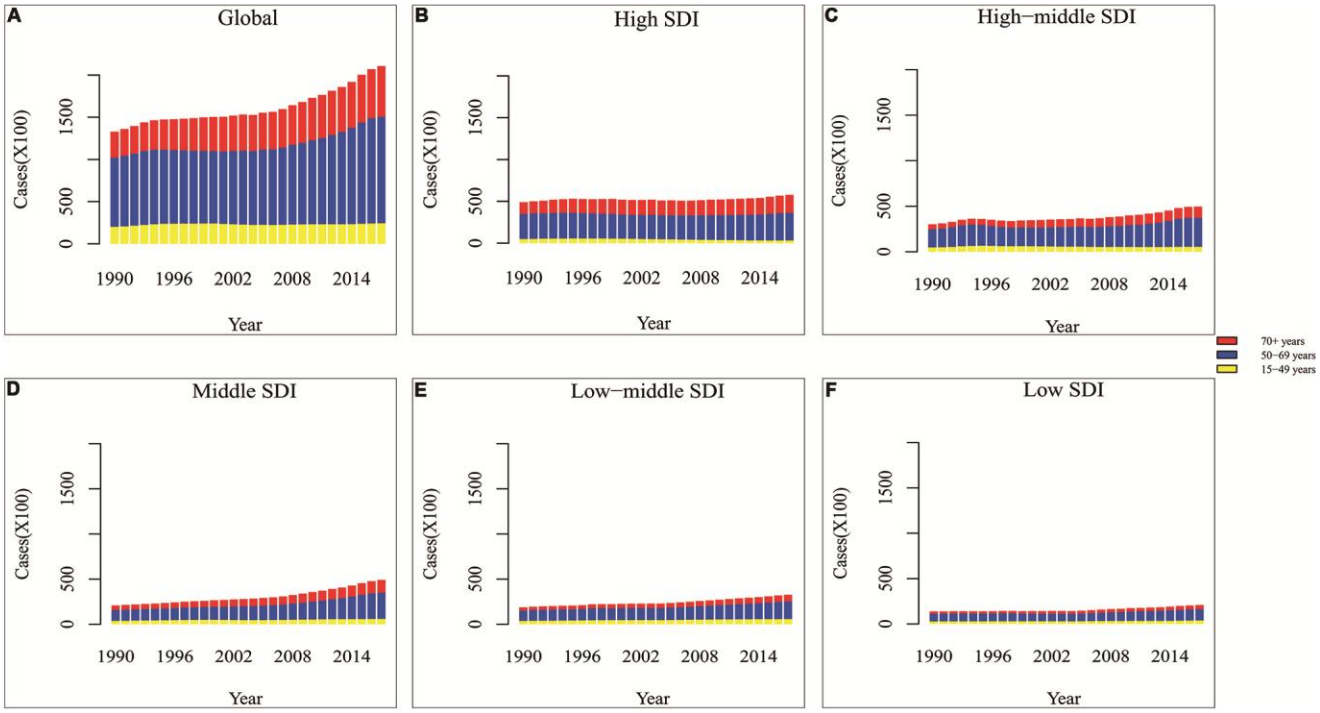

Supplementary Figure 4. Incident cases of larynx cancer among 28 years, three age groups and different SDI quantiles. (**A**)Global; (**B**) High SDI; (**C**) High-middle SDI; (**D**) Middle SDI; (**E**) Low-middle SDI; (**F**) Low SDI. SDI, socio-demographic index.

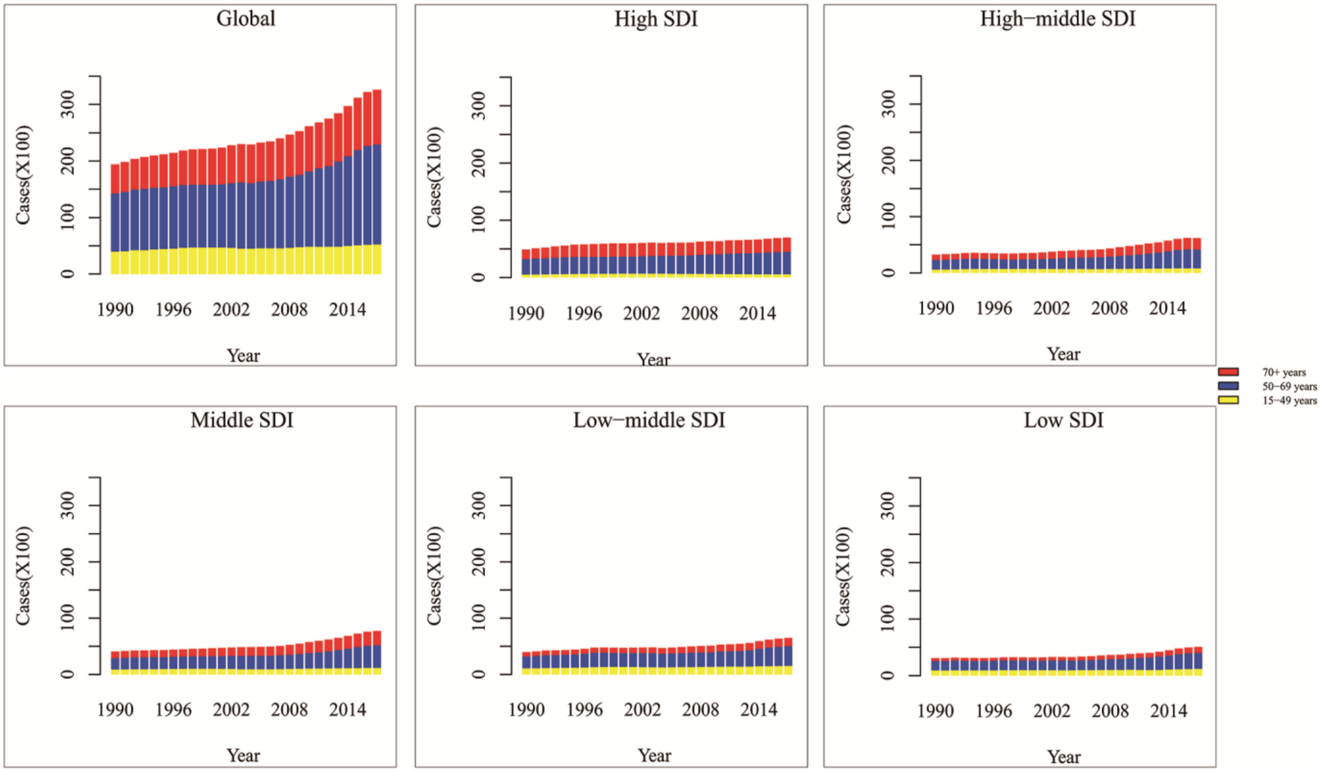

Supplementary Figure 5. Incident cases of larynx cancer among 28 years, three age groups and different SDI quantiles for female. (**A**)Global; (**B**) High SDI; (**C**) High-middle SDI; (**D**) Middle SDI; (**E**) Low-middle SDI; (**F**) Low SDI. SDI, socio-demographic index.

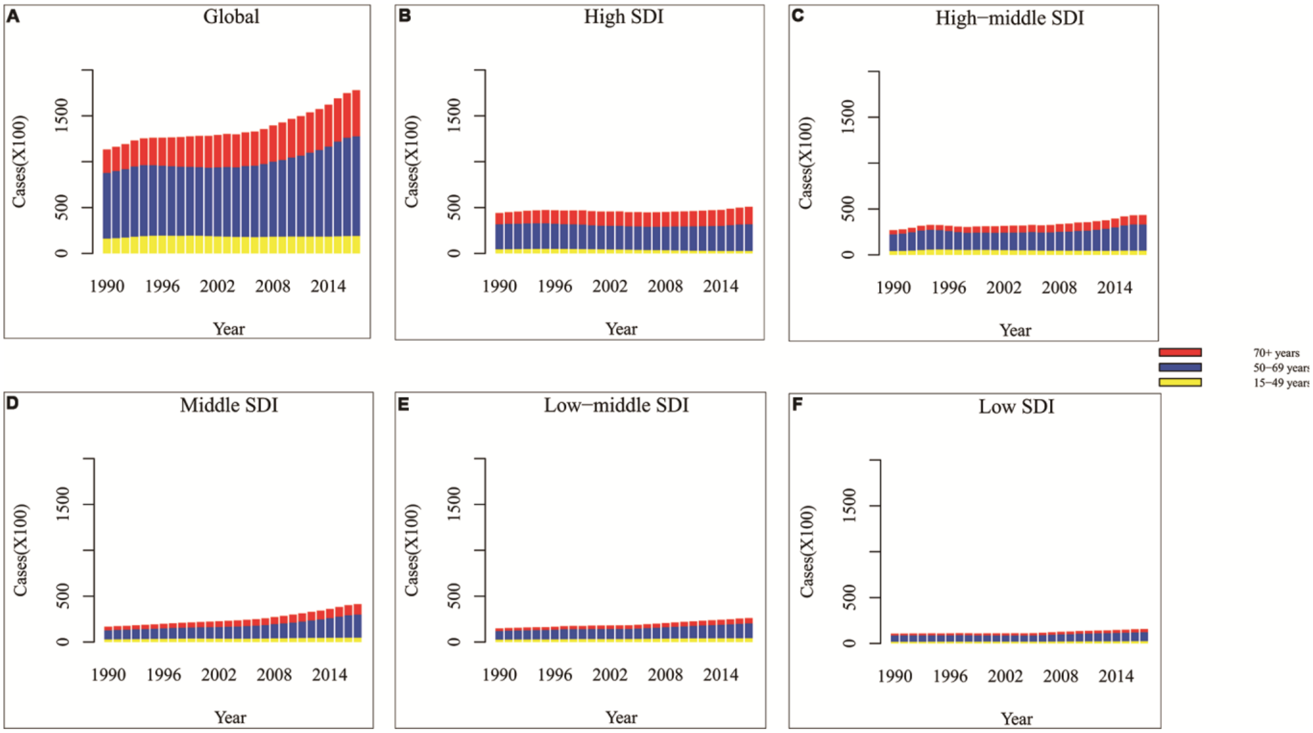

Supplementary Figure 6. Incident cases of larynx cancer among 28 years, three age groups and different SDI quantiles for male. (**A**)Global; (**B**) High SDI; (**C**) High-middle SDI; (**D**) Middle SDI; (**E**) Low-middle SDI; (**F**) Low SDI. SDI, socio-demographic index.

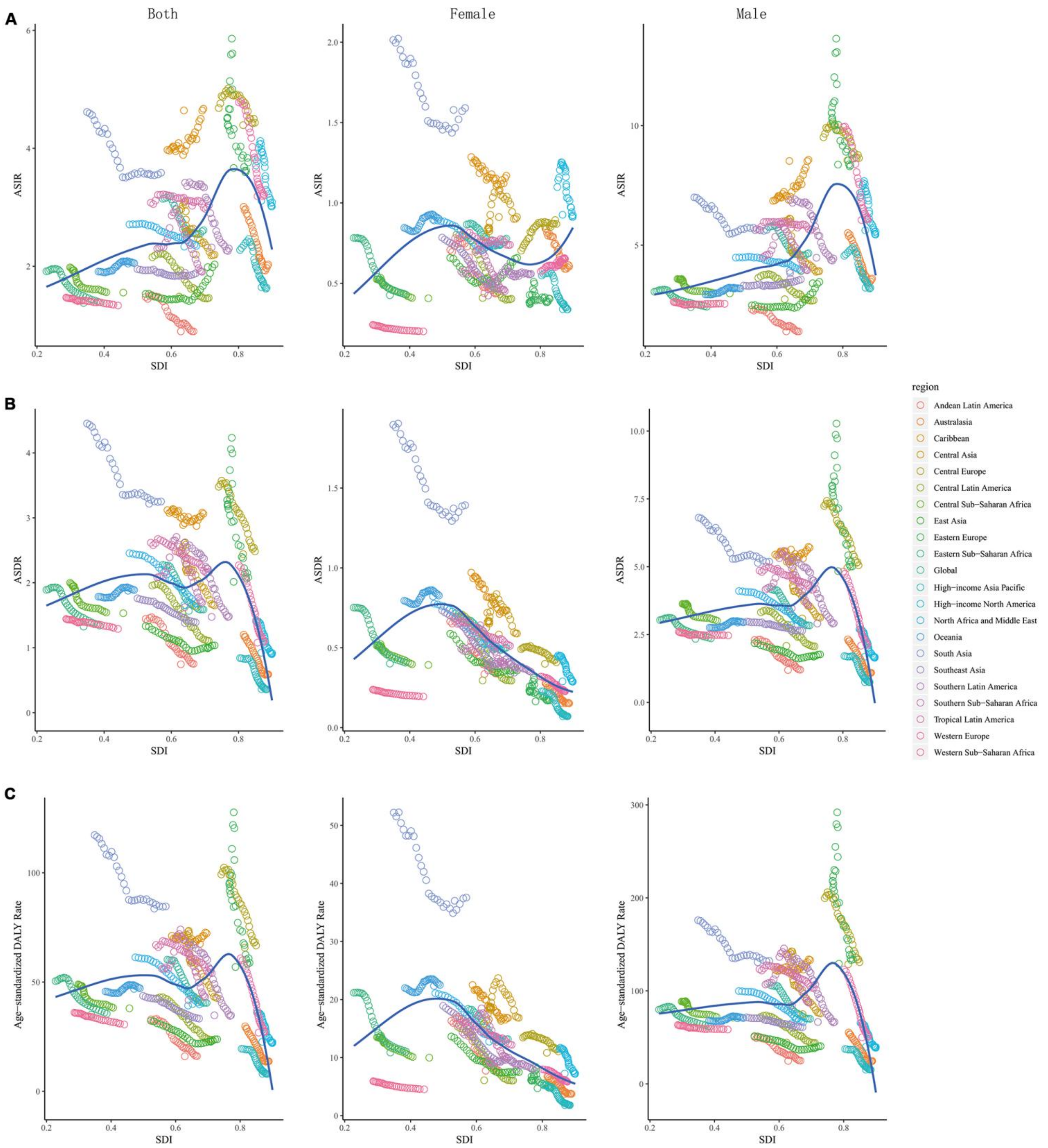

Supplementary Figure 7. The association between ASR of 28 years and SDI among 21 regions. (**A**) ASIR: age standardized incidence rate; (**B**) ASDR: age standardized death rate; (**C**) age-standardized DALY rate. DALY: disability adjusted life-year.

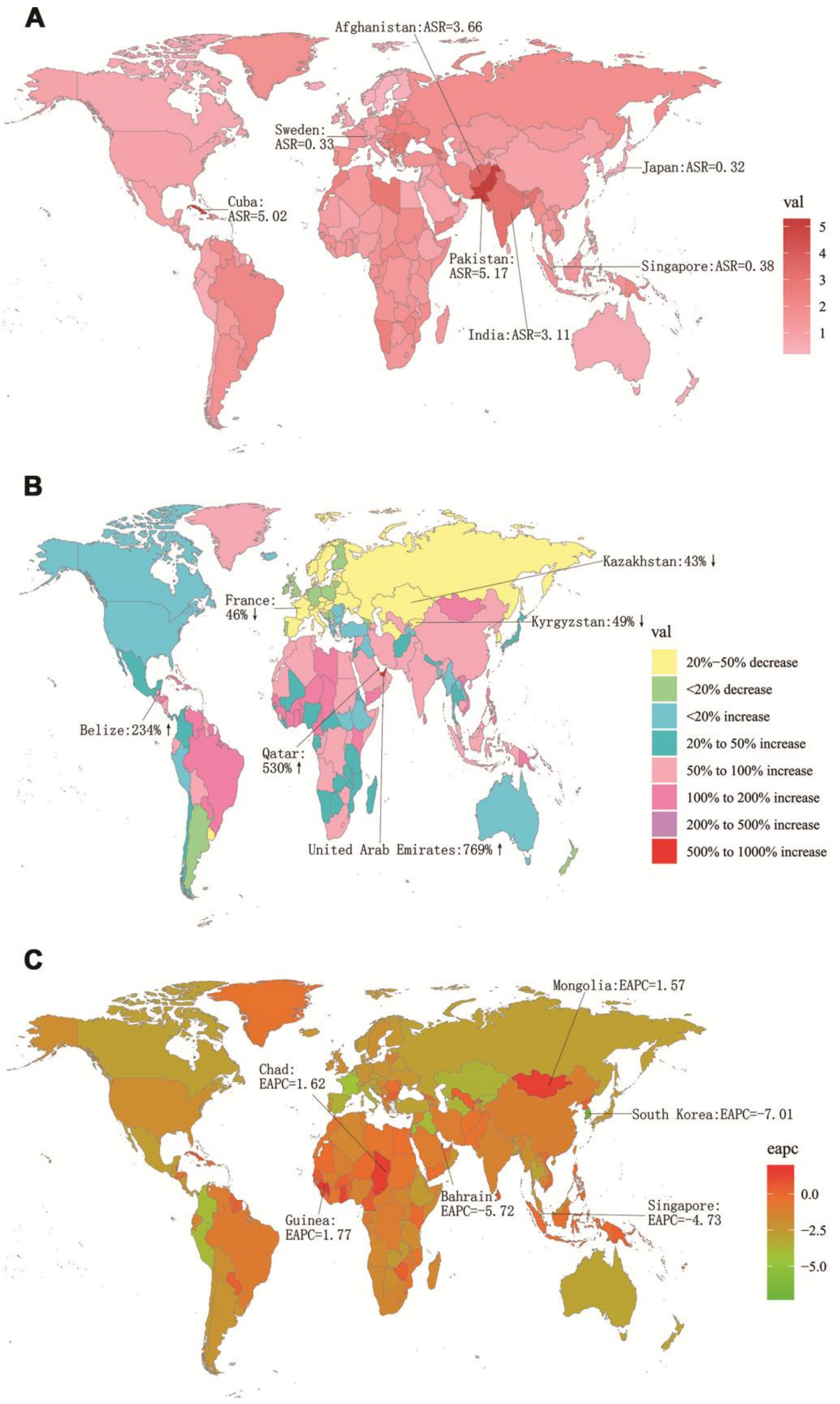

Supplementary Figure 8. The global death burden of larynx cancer in 195 countries. (**A**) The ASDR of larynx cancer in 2017; (**B**) The relative change in deaths of larynx cancer between 1990 and 2017; (**C**) The EAPC of larynx cancer ASDR from 1990 to 2017. Countries with an extreme number of cases/evolution were annotated. ASDR, age-standardized death rate; EAPC, estimated annual percentage change.

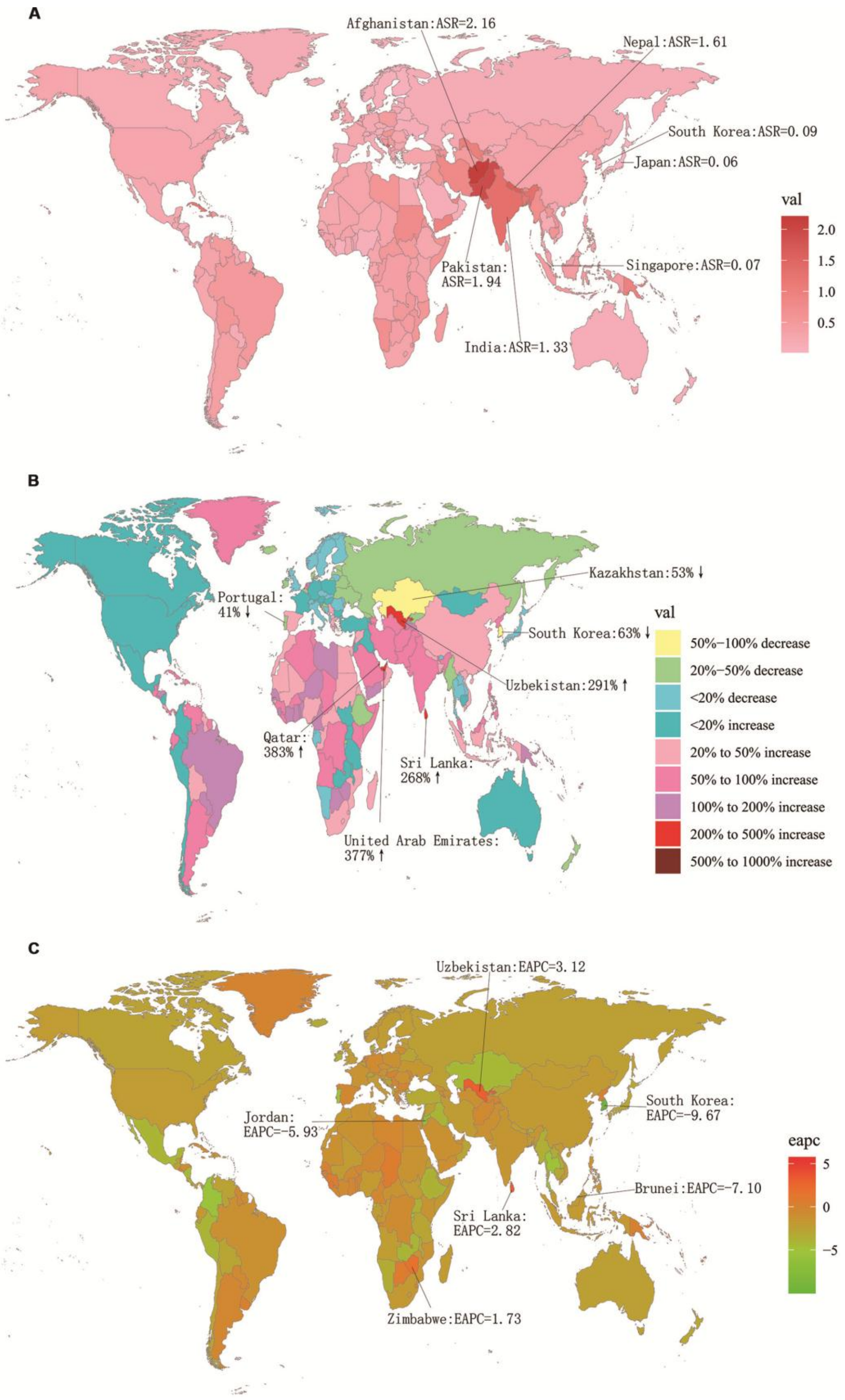

Supplementary Figure 9. The global death burden of larynx cancer among female in 195 countries. (**A**) The ASDR of larynx cancer in 2017; (**B**) The relative change in deaths of larynx cancer between 1990 and 2017; (**C**) The EAPC of larynx cancer ASDR from 1990 to 2017. Countries with an extreme number of cases/evolution were annotated. ASDR, age-standardized death rate; EAPC, estimated annual percentage change.

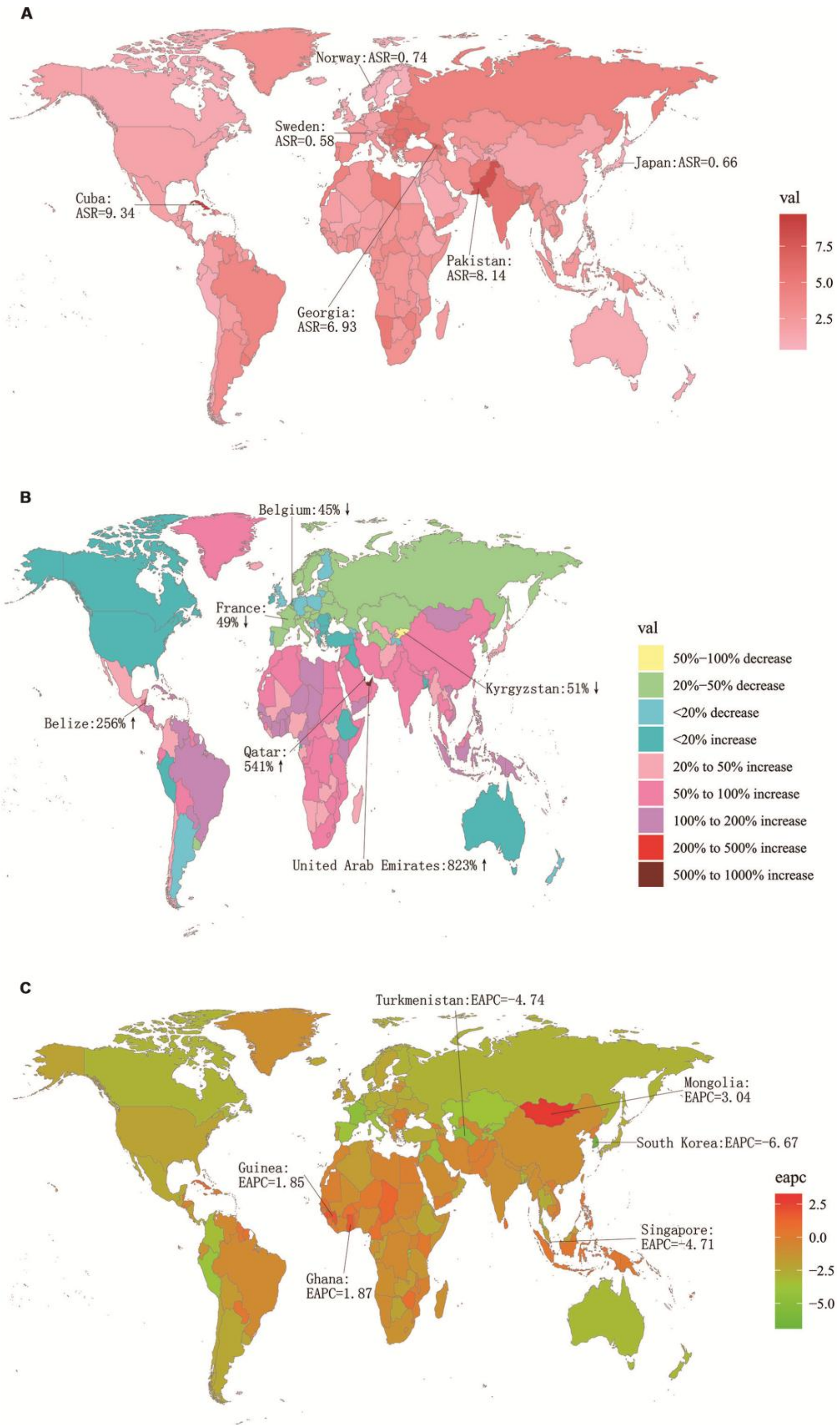

Supplementary Figure 10. The global death burden of larynx cancer among male in 195 countries. (**A**) The ASDR of larynx cancer in 2017; (**B**) The relative change in deaths of larynx cancer between 1990 and 2017; (**C**) The EAPC of larynx cancer ASDR from 1990 to 2017. Countries with an extreme number of cases/evolution were annotated. ASDR, age-standardized death rate; EAPC, estimated annual percentage change.

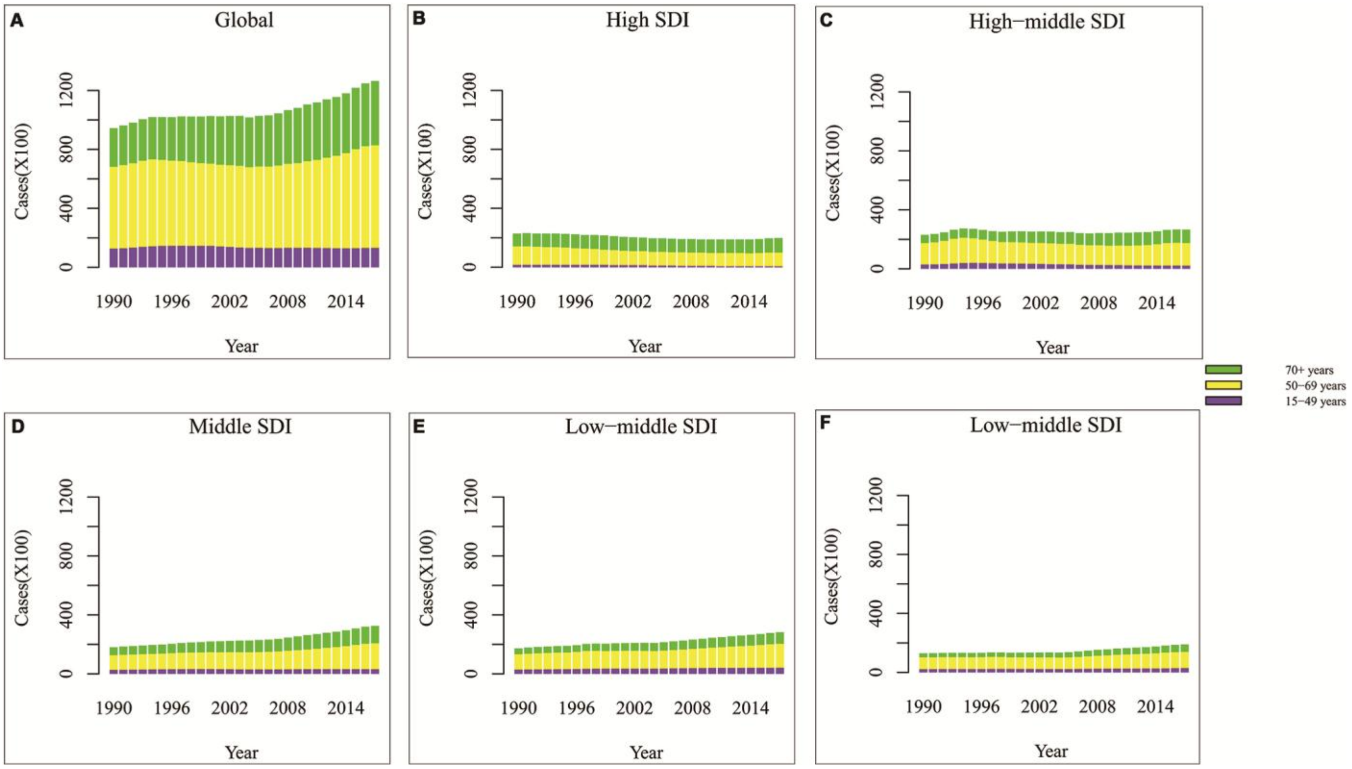

Supplementary Figure 11. Deaths of larynx cancer among 28 years, three age groups and different SDI quantiles. (**A**)Global; (**B**) High SDI; (**C**) High-middle SDI; (**D**) Middle SDI; (**E**) Low-middle SDI; (**F**) Low SDI. SDI, socio-demographic index.

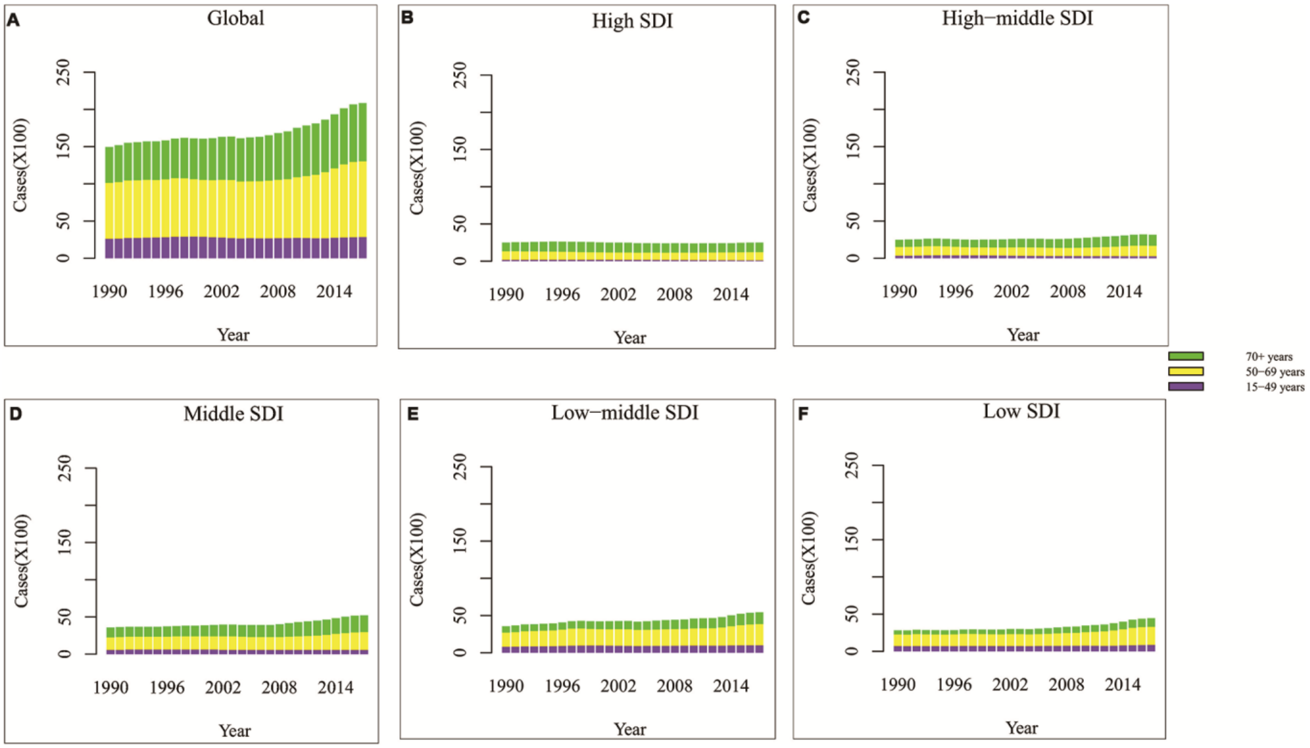

Supplementary Figure 12. Deaths of larynx cancer among 28 years, three age groups and different SDI quantiles for female. (**A**)Global; (**B**) High SDI; (**C**) High-middle SDI; (**D**) Middle SDI; (**E**) Low-middle SDI; (**F**) Low SDI. SDI, socio-demographic index.

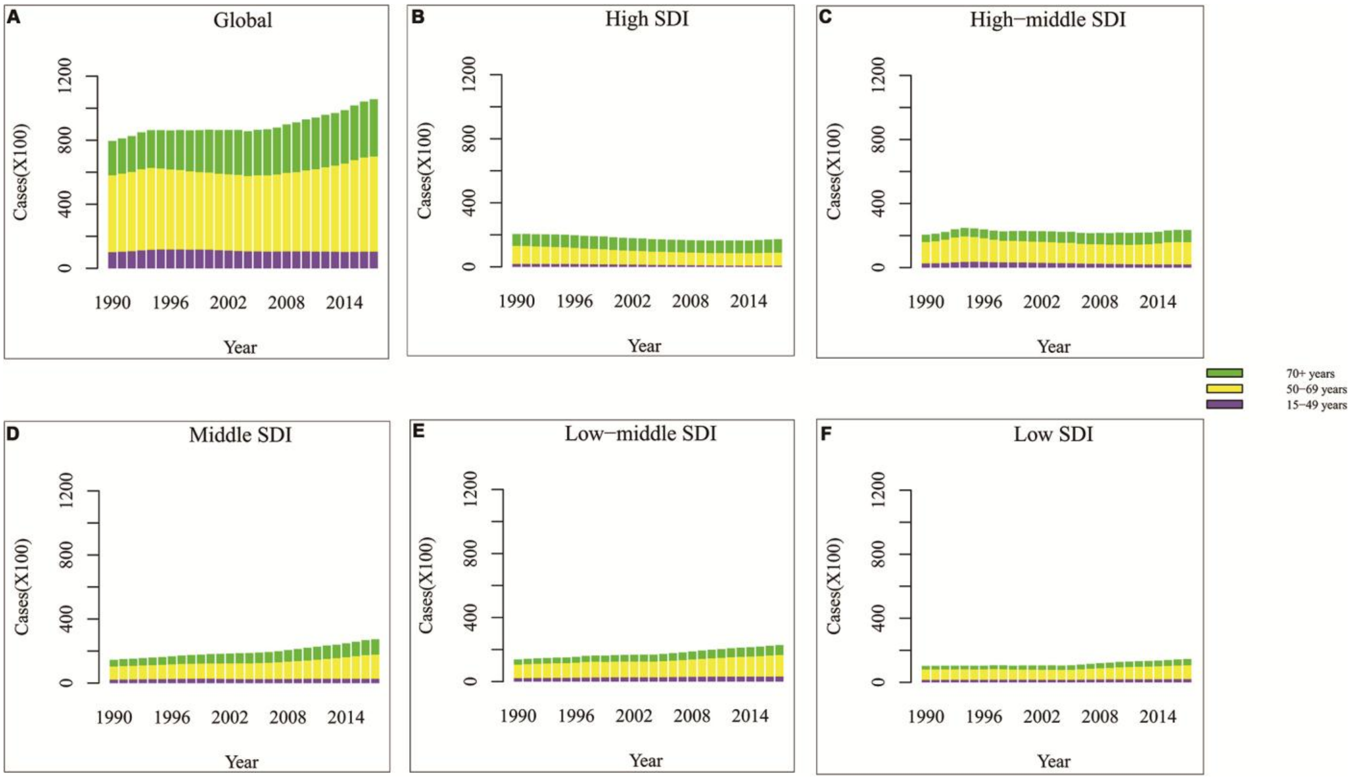

Supplementary Figure 13. Deaths of larynx cancer among 28 years, three age groups and different SDI quantiles for male. (**A**) Global; (**B**) High SDI; (**C**) High-middle SDI; (**D**) Middle SDI; (**E**) Low-middle SDI; (**F**) Low SDI. SDI, socio-demographic index.

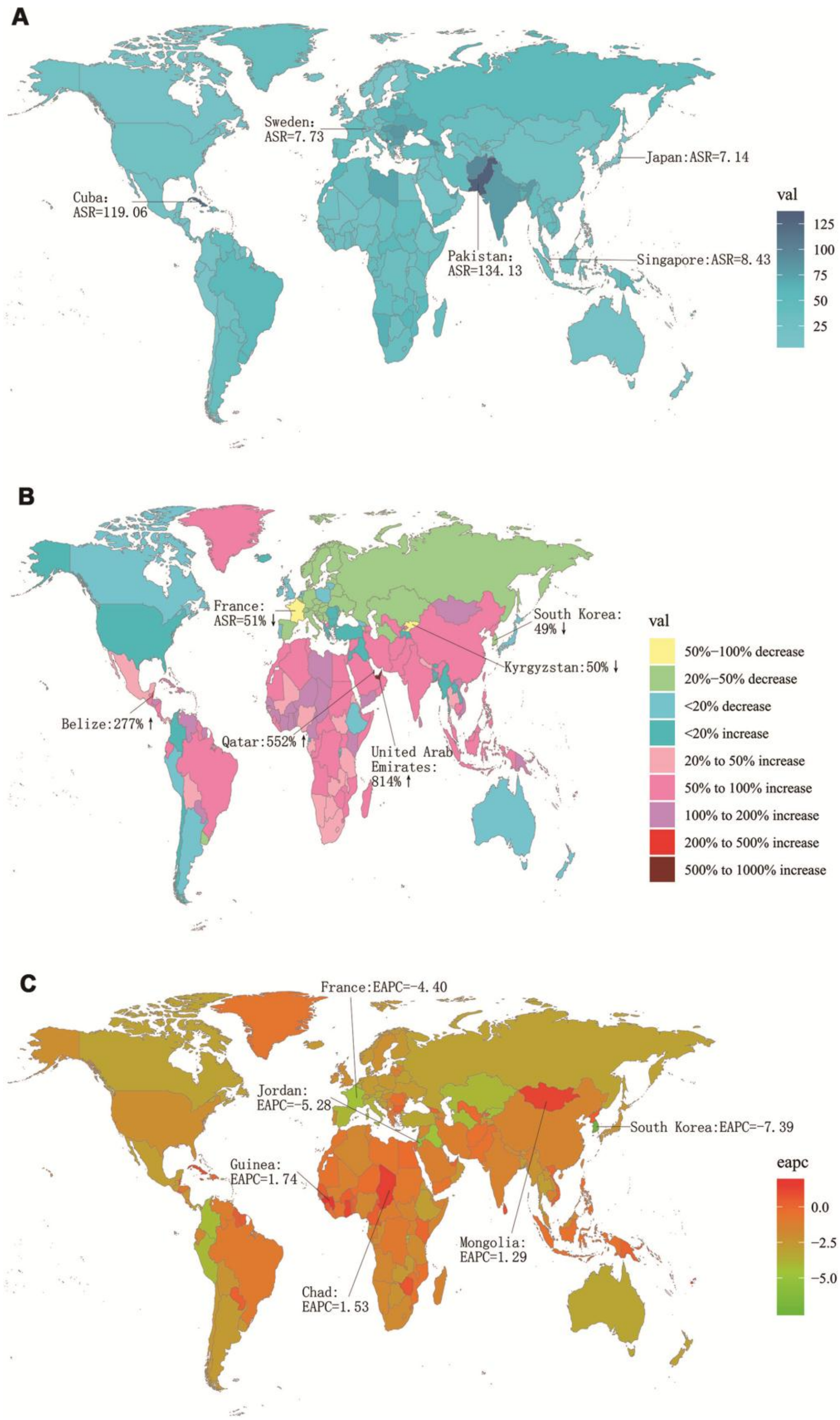

Supplementary Figure 14. The global DALYs burden of larynx cancer for both sexes in 195 countries. (**A**) The age-standardized DALY rate of larynx cancer in 2017; (**B**) The relative change in DALYs of larynx cancer between 1990 and 2017; (**C**) The EAPC of larynx cancer age-standardized DALY rate from 1990 to 2017. Countries with an extreme number of cases/evolution were annotated. DALY, disability adjusted life-year; EAPC, estimated annual percentage change.

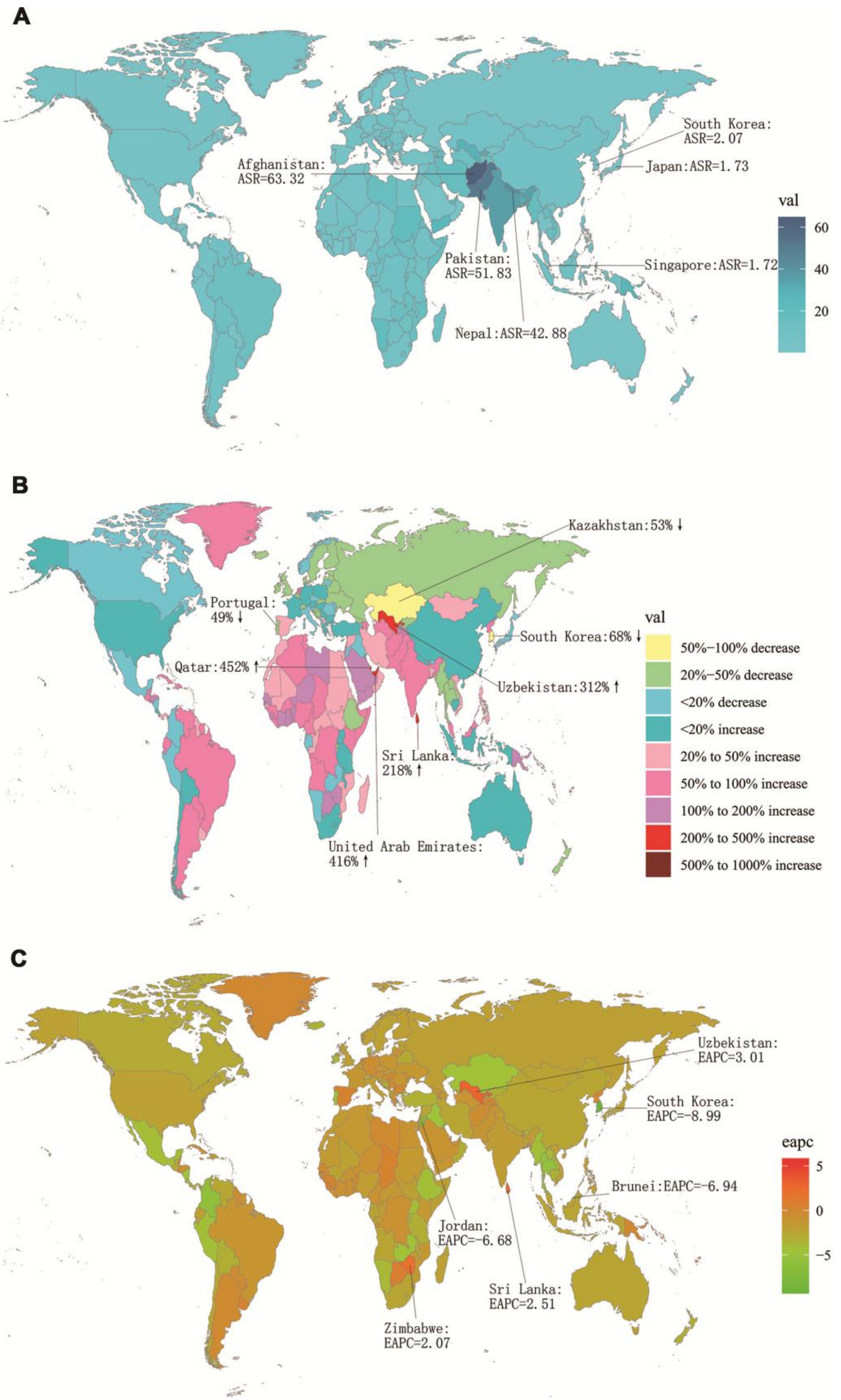

Supplementary Figure 15. The global DALYs burden of larynx cancer for among female in 195 countries. (**A**) The age-standardized DALY rate of larynx cancer in 2017; (**B**) The relative change in DALYs of larynx cancer between 1990 and 2017; (**C**) The EAPC of larynx cancer age-standardized DALY rate from 1990 to 2017. Countries with an extreme number of cases/evolution were annotated. DALY, disability adjusted life-year; EAPC, estimated annual percentage change.

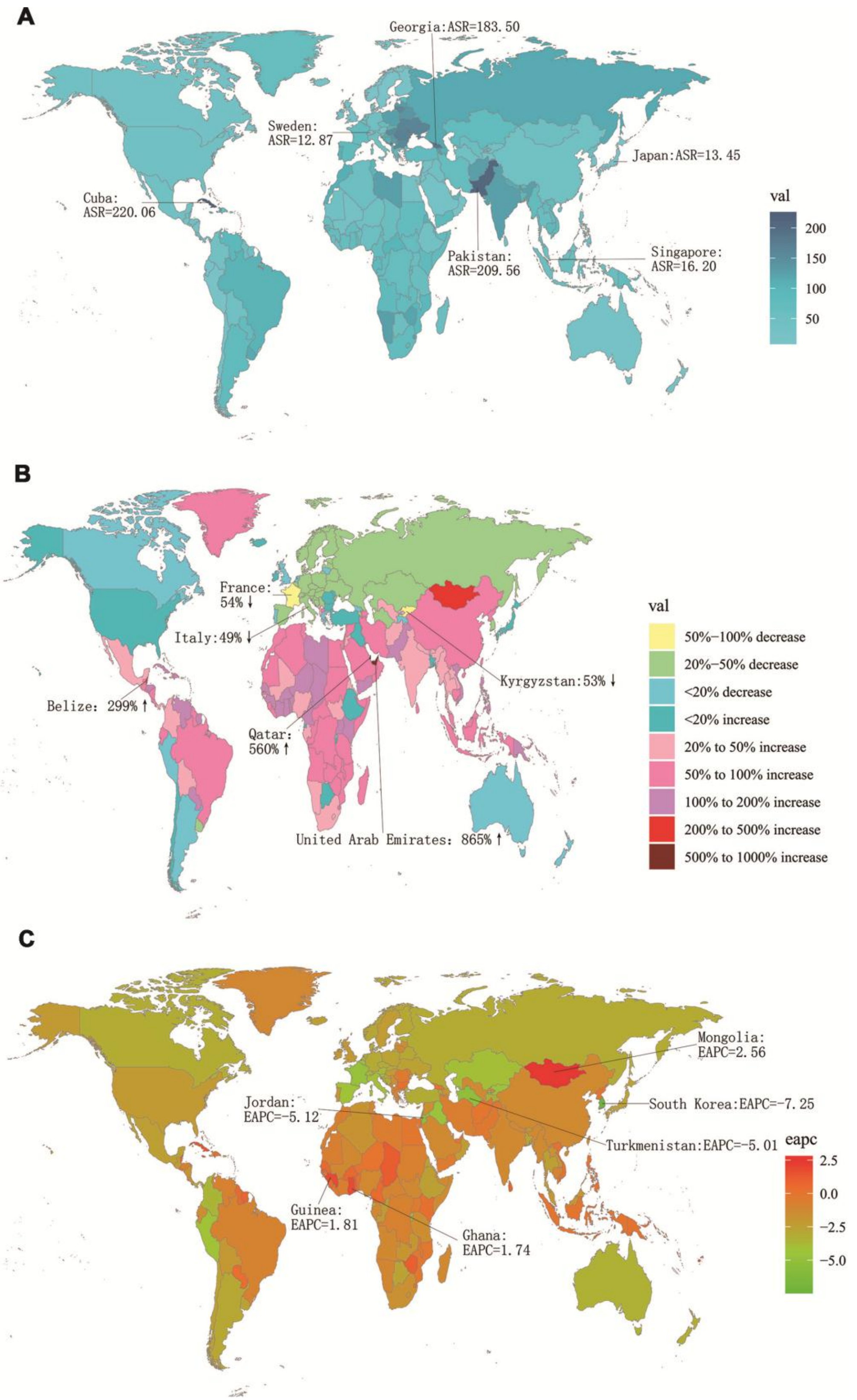

Supplementary Figure 16. The global DALYs burden of larynx cancer for among male in 195 countries. (**A**) The age-standardized DALY rate of larynx cancer in 2017; (**B**) The relative change in DALYs of larynx cancer between 1990 and 2017; (**C**) The EAPC of larynx cancer age-standardized DALY rate from 1990 to 2017. Countries with an extreme number of cases/evolution were annotated. DALY, disability adjusted life-year; EAPC, estimated annual percentage change.

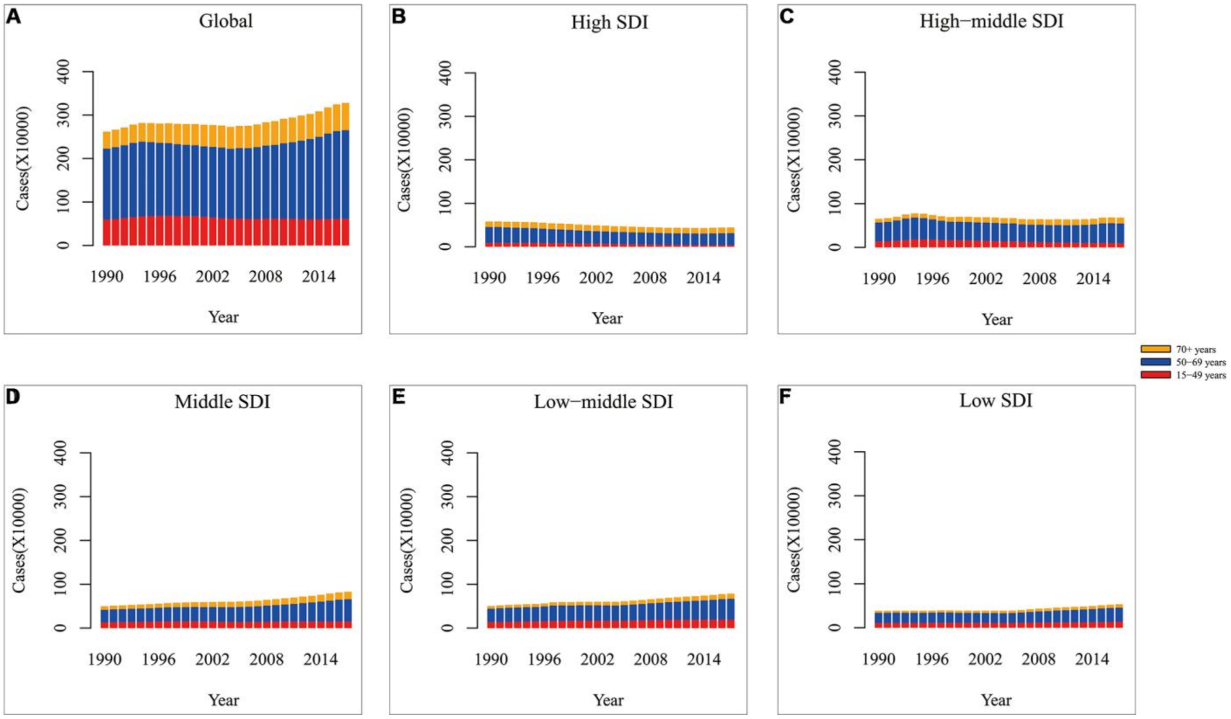

Supplementary Figure 17. DALYs of larynx cancer among 28 years, three age groups and different SDI quantiles. (**A**)Global; (**B**) High SDI; (**C**) High-middle SDI; (**D**) Middle SDI; (**E**) Low-middle SDI; (**F**) Low SDI. DALY, disability adjusted life-year; SDI, socio-demographic index.

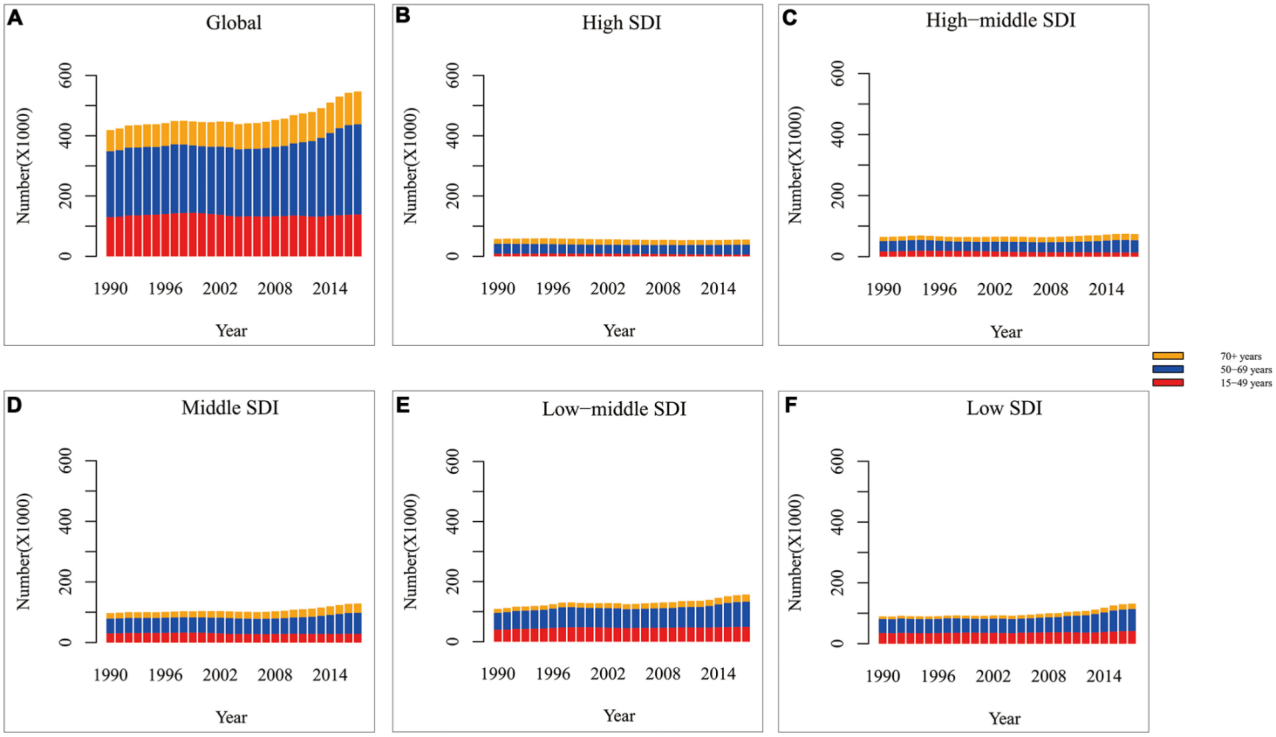

Supplementary Figure 18. DALYs of larynx cancer among 28 years, three age groups and different SDI quantiles for female. (**A**)Global; (**B**) High SDI; (**C**) High-middle SDI; (**D**) Middle SDI; (**E**) Low-middle SDI; (**F**) Low SDI. DALY, disability adjusted life-year; SDI, socio-demographic index

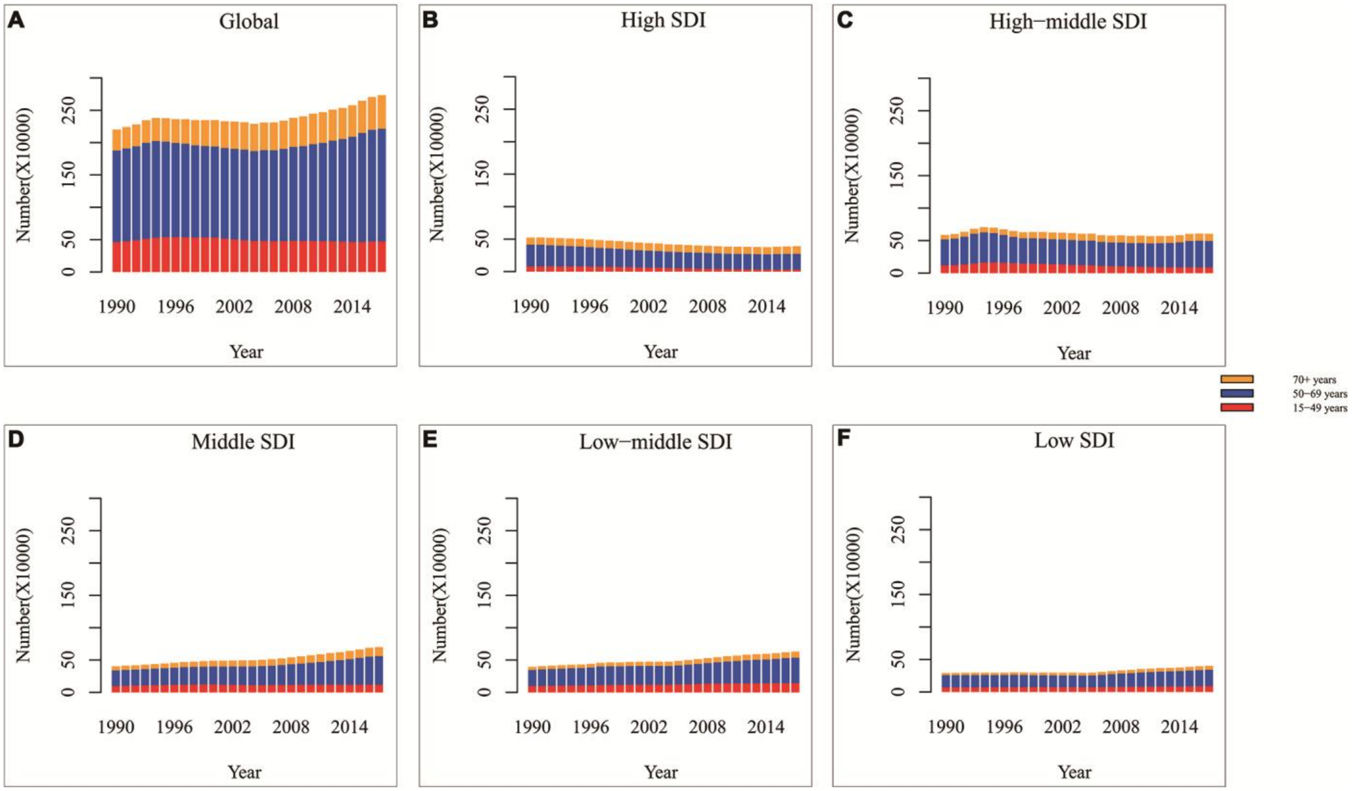

Supplementary Figure 19. DALYs of larynx cancer among 28 years, three age groups and different SDI quantiles for male. (**A**)Global; (**B**) High SDI; (**C**) High-middle SDI; (**D**) Middle SDI; (**E**) Low-middle SDI; (**F**) Low SDI. DALY, disability adjusted life-year; SDI, socio-demographic index

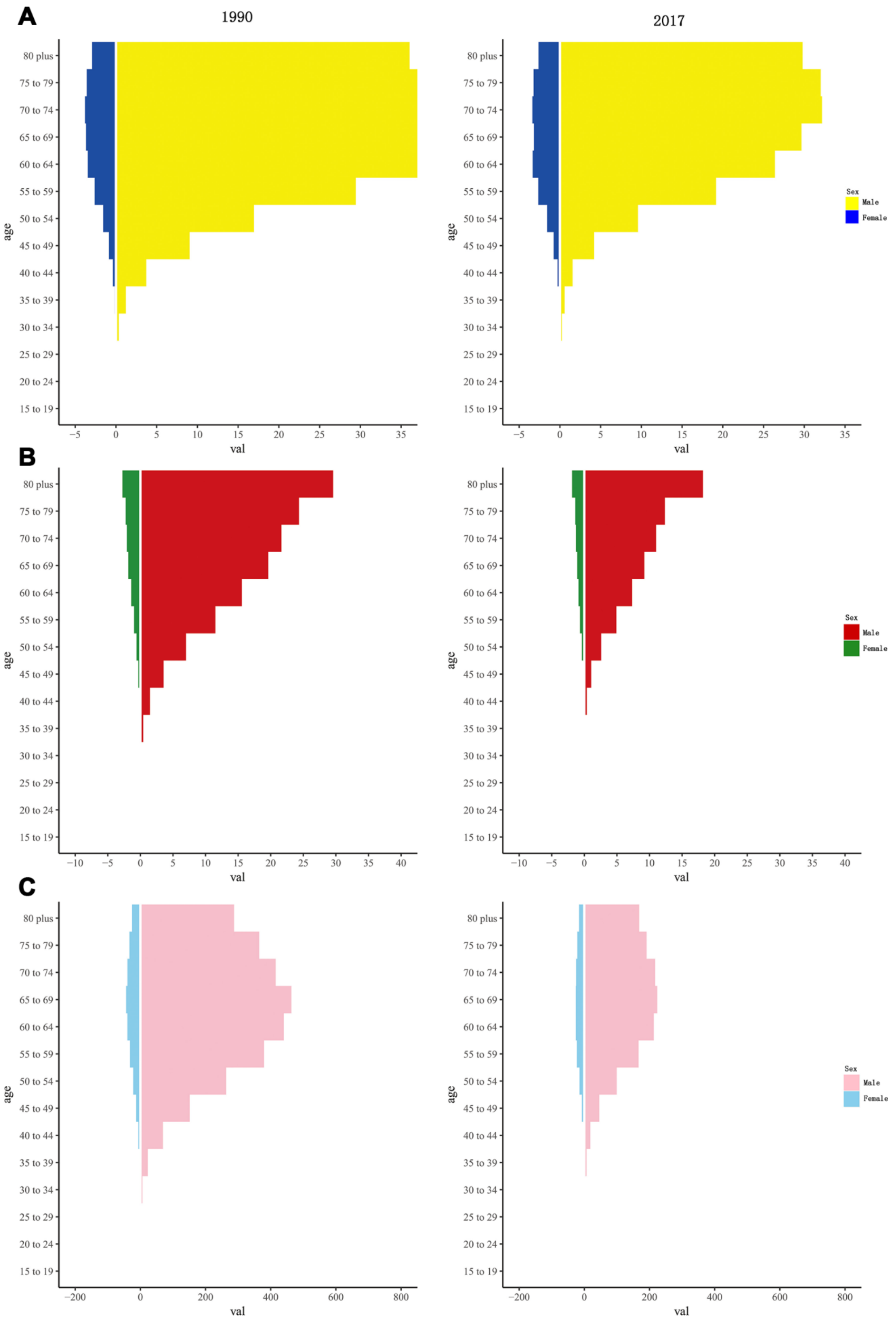

Supplementary Figure 20. The rate of larynx cancer in high SDI countries among gender and age in 1990 and 2017. (**A**) incidence rate; (**B**) death rate; (**C**) DALY rate. DALY, disability adjusted life-year.

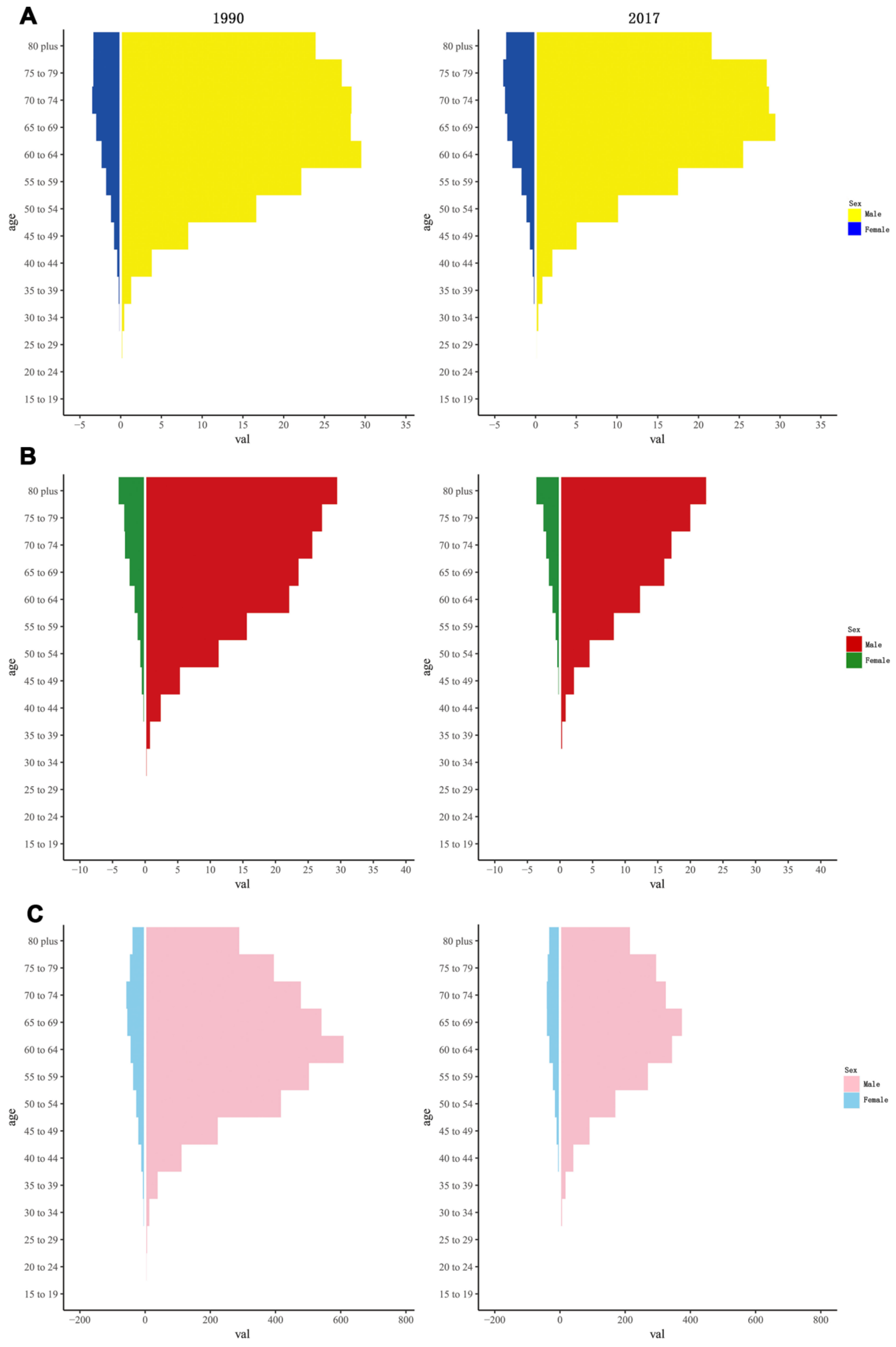

Supplementary Figure 21. The rate of larynx cancer in high-middle SDI countries among gender and age in 1990 and 2017. (**A**) incidence rate; (**B**) death rate; (**C**) DALY rate. DALY, disability adjusted life-year.

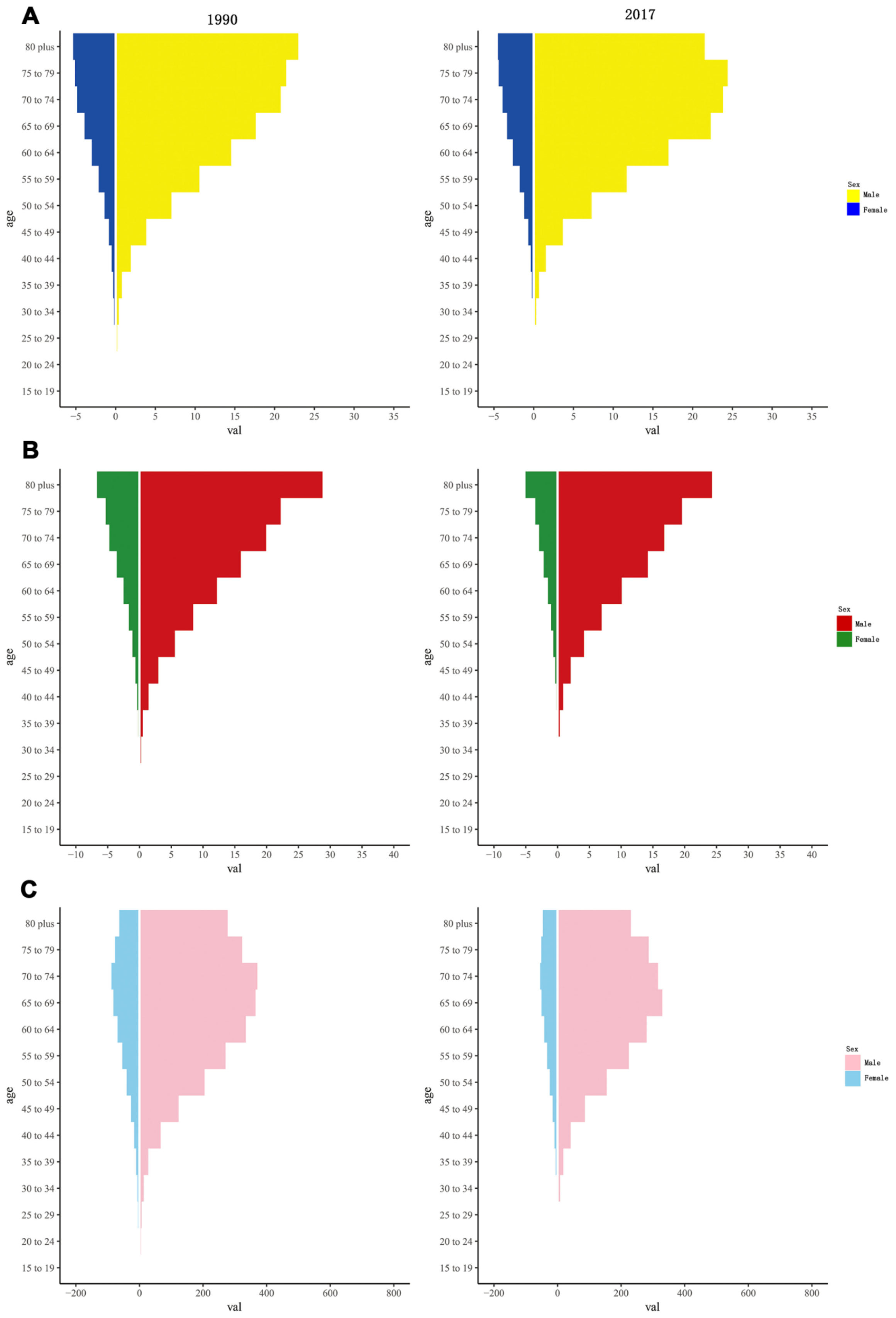

Supplementary Figure 22. The rate of larynx cancer in middle SDI countries among gender and age in 1990 and 2017. (**A**) incidence rate; (**B**) death rate; (**C**) DALY rate. DALY, disability adjusted life-year.

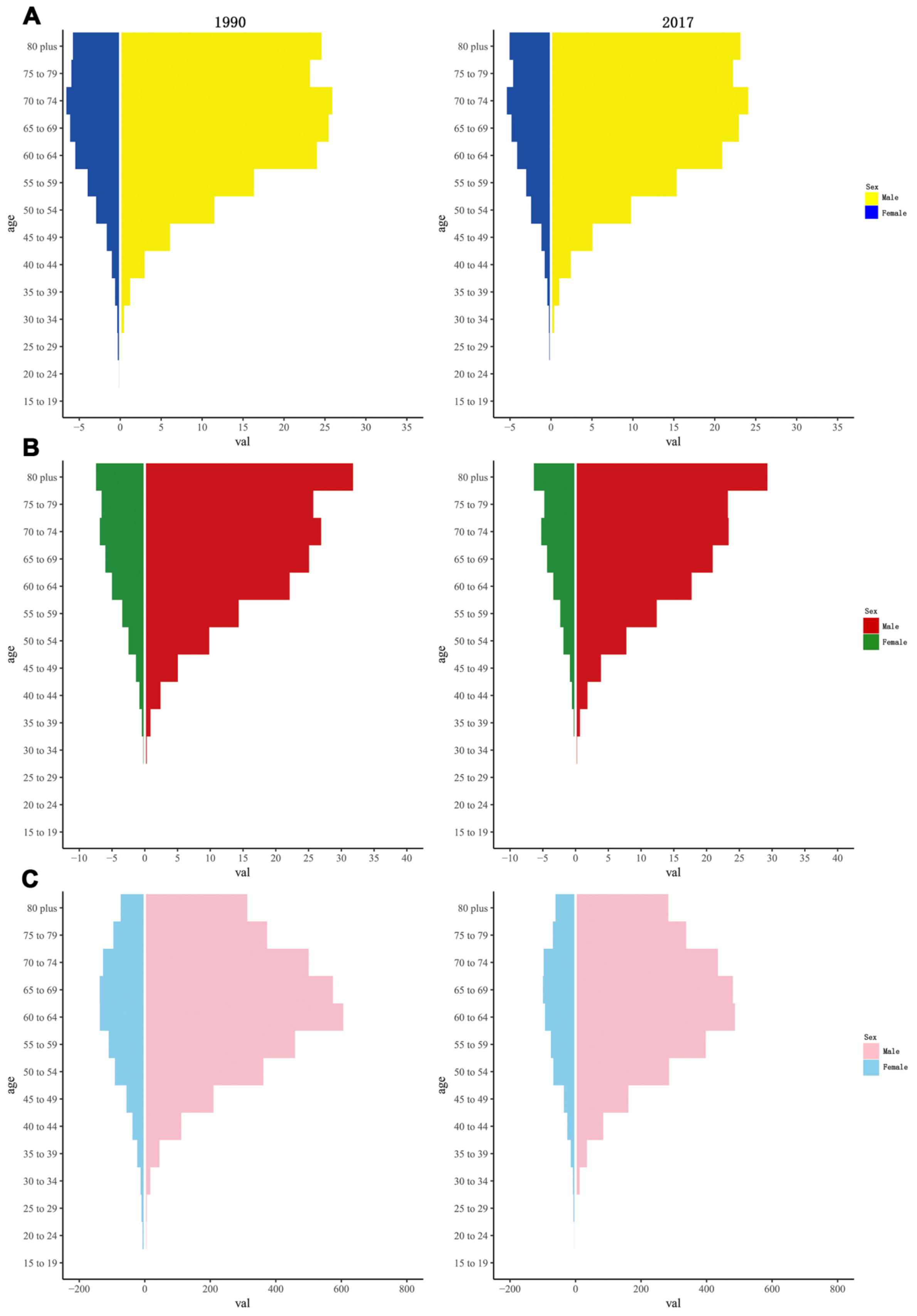

Supplementary Figure 23. The rate of larynx cancer in low-middle SDI countries among gender and age in 1990 and 2017. (**A**) incidence rate; (**B**) death rate; (**C**) DALY rate. DALY, disability adjusted life-year.

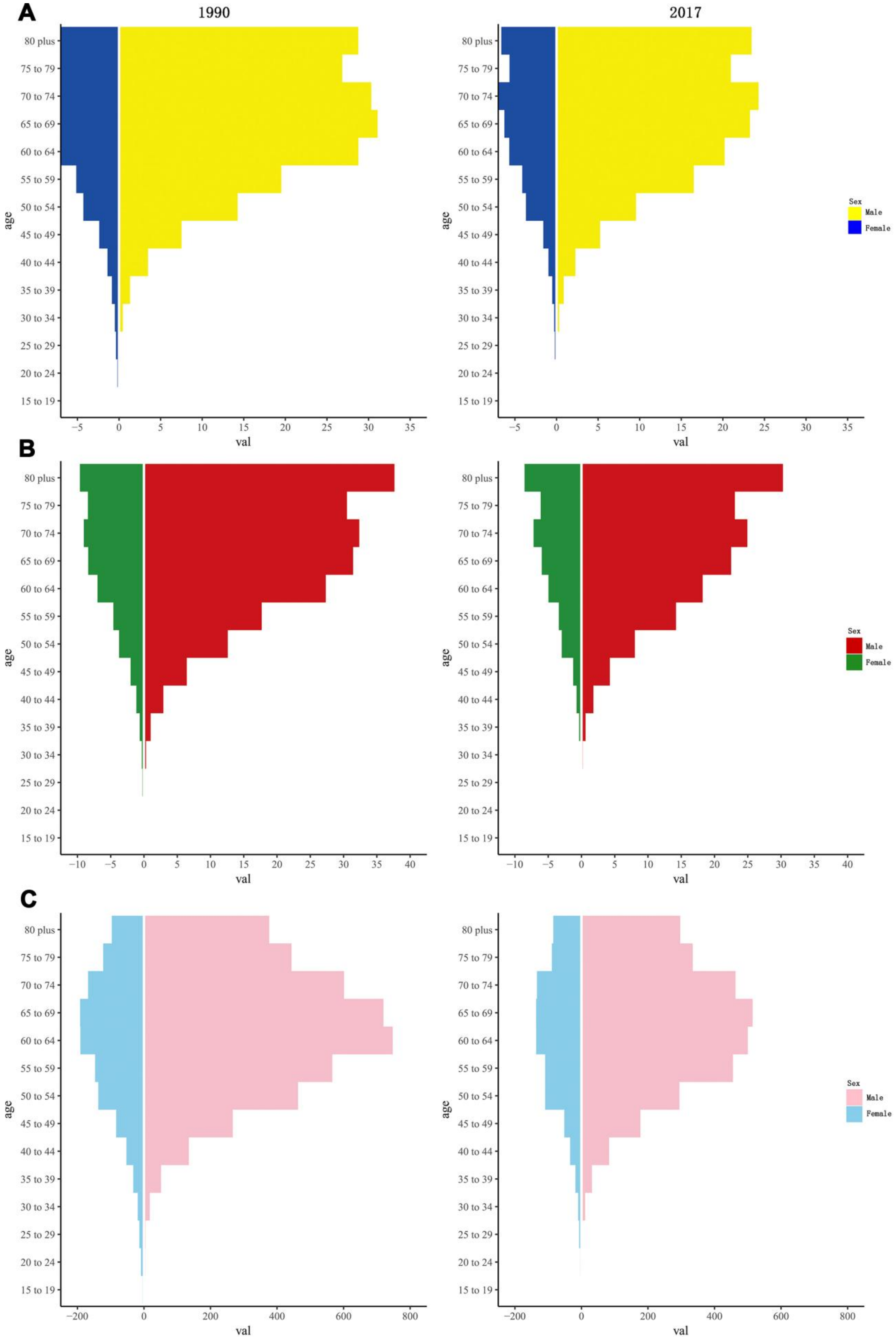

Supplementary Figure 24. The rate of larynx cancer in low SDI countries among gender and age in 1990 and 2017. (**A**) incidence rate; (**B**) death rate; (**C**) DALY rate. DALY, disability adjusted life-year.
