# Supplementary material for: Global burden of larynx cancer, 1990-2017: estimates from the global burden of disease 2017 study
Source: Aging (Albany NY). 2020 Feb 8;12(3):2545–83. doi: 10.18632/aging.102762 (PMC7041735; doi:10.18632/aging.102762)
Supplement: Supplementary Table 1 [file aging-12-102762-s002..docx]

**Supplementary Table 1. The top three and the bottom three countries of larynx cancer incidence, death, or DALY.**

| **Measure** | **Sex** | **Top three countries** | | | **Bottom three countries** | | |
| --- | --- | --- | --- | --- | --- | --- | --- |
| **Cases of 2017** | | | | | | | |
| Incidence | | | | | | | |
|  | Both | Hungary（39748.99） | China（39725.40） | USA（17048.53） | American Samoa(0.68) | Kazakhstan(0.70) | Mali(0.79) |
|  | Female | Hungary(9038.35) | China(6425.80) | USA(2628.19) | Antigua（0.08） | Barbuda（0.08） | Greece(0.10) |
|  | Male | China(33299.59 ) | Hungary(30710.64) | USA(14420.33 ) | Kazakhstan  (0.41) | American Samoa  (0.55) | Mali(0.61) |
| Death |  |  |  |  |  |  |  |
|  | Both | India(34658.69) | China(19460.63) | Pakistan(5911.76) | American Samoa(0.46) | Marshall Islands(0.64) | Kiribati  (0.66) |
|  | Female | India(7644.12） | China(3351.27） | Pakistan(1092.61） | Antigua  (0.05) | Barbuda  (0.05) | Greenland(0.06） |
|  | Male | India(27014.57 ) | China(16109.35) | Pakistan(4819.15) | American Samoa  (0.37) | Kiribati  (0.38) | Marshall Islands  (0.50) |
| DALY | | | | | | | |
|  | Both | India(963096.98) | China(465228.27) | Pakistan(174319.52) | American Samoa(12.09) | Samoa(17.91) | Kiribati(18.88) |
|  | Female | India(218208.58) | China(71781.99) | Pakistan(34596.26) | Antigua(1.24) | Barbuda(1.24) | Greenland(1.59) |
|  | Male | India(744888.40) | China(393446.28) | Pakistan(139723.26) | American Samoa  (9.54) | Kiribati(10.74) | Marshall Islands  (14.51) |
| **ASR of 2017 (per 100,000 people)** | | | | | | | |
| ASIR | | | | | | | |
|  | Both | Cuba（8.58） | Seychelles（7.85） | Montenegro（7.10） | Samoa  (0.68) | Peru (0.73) | Gambia (0.75) |
|  | Female | Montenegro(3.31) | Afghanistan（2.32） | Pakistan（2.15） | Ivory Coast（0.10） | Nigeria（0.12） | Maldives(0.13） |
|  | Male | Cuba(15.67) | Seychelles(15.65) | Lithuania(12.28) | Tajikistan(1.10) | Peru(1.13) | Samoa(1.27) |
| ASDR | | | | | | | |
|  | Both | Panama(5.17) | Cuba(5.02) | Sierra Leone(4.62) | Kazakhstan(0.32) | Switzerland(0.33) | Slovakia(0.38) |
|  | Female | Afghanistan(2.16） | Panama(1.94） | New Zealand(1.61） | Kazakhstan  (0.07) | Slovakia  (0.07) | Tajikistan(0.08） |
|  | Male | Sierra Leone  (9.49) | Cuba(9.34) | Panama(8.14) | Switzerland  (0.58) | Kazakhstan  (0.67) | Pakistan  (0.74) |
| Age Standardized DALY Rate | | | | | | | |
|  | Both | Panama(134.13) | Cuba(119.06) | Sierra Leone(114.52) | Kazakhstan(7.14) | Switzerland(7.73) | Slovakia(8.43) |
|  | Female | Afghanistan(63.32) | Panama(51.84) | New Zealand(42.88) | Slovakia(1.72) | Kazakhstan(1.73) | Tajikistan(1.83) |
|  | Male | Sierra Leone(221.06) | Cuba(220.06) | Panama(209.56) | Switzerland(12.87) | Kazakhstan(13.45) | Slovakia(16.20) |
| **Increase in the number of cases/years from1990 to 2017 (-fold)** | | | | | | | |
| Incidence | | | | | | | |
|  | Both | Qatar(99766.43%) | Iceland (61611.68%) | Sao Tome and Principe  (46178.74%) | Russia(-99.93 %) | Kazakhstan(-99.87%) | Nigeria (-99.77%) |
|  | Female | Qatar  (85568.91%) | Iceland  (62255.94%) | Sao Tome and Principe(41959.49%) | Russia(-99.89 %) | Greece(-99.76 %) | Kazakhstan  (-99.59 %) |
|  | Male | Qatar(100880.82%) | Iceland(61493.92%) | Sao Tome and Principe(46745.42%) | Russia(-99.93 %) | Kazakhstan  (-99.91%) | Nigeria(-99.80%) |
| Death | | | | | | | |
|  | Both | United Arab Emirates  (769.01%) | Qatar(530.50%) | Belize(234.24%) | Kyrgyzstan(-48.86%) | France(-45.90%) | Kazakhstan(-43.29%) |
|  | Female | Guam(611.95%) | Northern Mariana Islands(478.95) | Qatar  (383.5%) | South Korea (-63.30%) | Kazakhstan  (-53.43%) | Portugal(-41.09%) |
|  | Male | United Arab Emirates  (822.76%) | Qatar(541.11%) | Belize(255.84%) | Kyrgyzstan  (-51.01%) | France(-48.98%) | Belgium(-44.99%) |
| DALY | | | | | | | |
|  | Both | United Arab Emirates  (814.13%) | Qatar(552.42%) | Belize(277.01%) | France(-51.08%) | Kyrgyzstan(-50.48%) | South Korea(-48.94) |
|  | Female | Guam(552.36%) | Qatar(451.58) | Northern Mariana Islands(428.23) | South Korea(-68.34%) | Kazakhstan(-53.01%) | Portugal(-49.72%) |
|  | Male | United Arab Emirates  (865.04%) | Qatar(560.34%) | Belize(298.87%) | France(-54.01%) | Kyrgyzstan(-52.87%) | Italy(-49.06%) |
| **EAPC** | | | | | | | |
| Incidence | | | | | | | |
|  | Both | Sri Lanka (2.31) | Mongolia (1.82) | Guinea (1.82) | Bahrain(-4.79) | Jordan (-4.03) | Iraq (-3.87) |
|  | Female | Guam(5.55) | American Samoa  (4.32) | Sri Lanka  (4.04) | South Korea  (-5.94) | Brunei  (-5.69) | Mauritius  (-5.50) |
|  | Male | Mongolia(3.20) | Sri Lanka(2.46) | Ghana(1.97) | Bahrain(-4.69) | Turkmenistan  (-4.39) | Iraq(-3.96) |
| Death | | | | | | | |
|  | Both | Guinea(1.77) | Chad(1.62) | Mongolia(1.57) | South Korea(-7.10) | Bahrain(-5.73) | Brunei(-5.13) |
|  | Female | Guam(5.39) | American Samoa  (3.72) | Cape Verde  (3.32) | South Korea(-9.67) | Brunei(-7.10) | Mauritius(-6.19) |
|  | Male | Mongolia(3.04) | Ghana(1.87) | Guinea(1.85) | South Korea(-6.67) | Bahrain(-5.51) | Turkmenistan(-4.74) |
| DALY | | | | | | | |
|  | Both | Guinea(1.74) | Chad(1.53) | Mongolia(1.29) | South Korea(-7.39) | Bahrain(-5.72) | Jordan(-5.28) |
|  | Female | Guam(5.49) | American Samoa(3.94) | Uzbekistan(3.01) | South Korea(-8.99) | Brunei(-6.94) | Jordan(-6.68) |
|  | Male | Mongolia(2.56) | Guinea(1.81) | Ghana(1.74) | South Korea(-7.25) | Bahrain(-5.67) | Jordan(-5.12) |

**Abbreviations:** ASDR, age standardized death rate; ASIR, age standardized incidence rate; DALY, disability adjusted life-year
