# Supplementary material for: Global burden of larynx cancer, 1990-2017: estimates from the global burden of disease 2017 study
Source: Aging (Albany NY). 2020 Feb 8;12(3):2545–83. doi: 10.18632/aging.102762 (PMC7041735; doi:10.18632/aging.102762)
Supplement: Supplementary Table 2 [file aging-12-102762-s001..docx]

**Supplementary Table 2. The top three and the bottom three regions of larynx cancer incidence, death, or DALY.**

| **Measure** | **Sex** | **Top three regions** | | | **Bottom three regions** | | |
| --- | --- | --- | --- | --- | --- | --- | --- |
| **Cases of 2017** | | | | | | | |
| Incidence | | | | | | | |
|  | Both | South Asia(50557.41） | East Asia(41889.88） | Western Europe(25274.97） | Oceania(141.06) | Andean Latin America(482.58) | Central Sub-Saharan Africa(820.44) |
|  | Female | South Asia(11433.28) | East Asia(6649.06) | High-income North America(2900.87) | Oceania(32.02） | Central Sub-Saharan Africa(118.47） | Andean Latin America  (123.35) |
|  | Male | South Asia(39124.13) | East Asia(35240.82) | Western Europe(  22666.98) | Oceania(109.04) | Andean Latin America(359.24) | Central Sub-Saharan Africa(701.97) |
| Death |  |  |  |  |  |  |  |
|  | Both | South Asia(44258.09) | East Asia(20430.02) | Western Europe(9257.59) | Oceania(119.50) | Australasia(293.03) | Andean Latin America(397.62) |
|  | Female | South Asia(9643.47） | East Asia(3487.65） | Southeast Asia(1190.91） | Oceania(27.41) | Australasia(39.27) | Andean Latin America  (93.44） |
|  | Male | South Asia(34614.63) | East Asia(16942.37) | Western Europe(  8203.244) | Oceania(92.09) | Australasia(253.76) | Andean Latin America  (304.17) |
| DALY | | | | | | | |
|  | Both | South Asia(1236890.09) | East Asia(489640.67) | Southeast Asia(  209250.40) | Oceania(3738.75) | Australasia(6296.69) | Andean Latin America(8723.46) |
|  | Female | South Asia(278933.69) | East Asia(74904.32) | Southeast Asia(  30280.71) | Australasia(853.71) | Oceania(896.55) | Andean Latin America  (2197.35) |
|  | Male | South Asia(957956.40) | East Asia(414736.35) | Western Europe(  183088.29) | Oceania(2842.2) | Australasia(5442.99) | Andean Latin America  (6526.1) |
| **ASR of 2017 (per 100,000 people)** | | | | | | | |
| ASIR | | | | | | | |
|  | Both | Caribbean(4.64） | Central Europe(4.39) | Eastern Europe(3.67） | Andean Latin America  (0.90) | Western Sub-Saharan Africa  (1.33) | Eastern Sub-Saharan Africa(1.38) |
|  | Female | South Asia(1.58) | Caribbean(1.16） | High-income North America(0.93） | Western Sub-Saharan Africa(0.20) | High-income Asia Pacific(0.34） | Eastern Europe  (0.39） |
|  | Male | Central Europe(8.55) | Caribbean(8.53) | Eastern Europe  (8.37) | Andean Latin America(1.39) | Eastern Sub-Saharan Africa(2.41) | Western Sub-Saharan Africa(2.55) |
| ASDR | | | | | | | |
|  | Both | South Asia(3.22) | Caribbean(3.02) | Central Europe(2.44) | High-income Asia Pacific(0.36) | Australasia(0.60) | Andean Latin America  (0.75) |
|  | Female | South Asia(1.37） | Oceania(0.82） | Caribbean(0.72） | High-income Asia Pacific(0.07) | Australasia(0.15) | Eastern Europe(0.16） |
|  | Male | Caribbean(5.63) | South Asia(5.14) | Central Europe(4.96) | High-income Asia Pacific(0.75) | Australasia  (1.12) | Andean Latin America  (1.20) |
| Age Standardized DALY Rate | | | | | | | |
|  | Both | South Asia(83.63) | Caribbean(71.27) | Central Europe(65.76) | High-income Asia Pacific(7.98) | Australasia  (14.02) | Andean Latin America  (15.97) |
|  | Female | South Asia(37.01) | Oceania(22.34) | Central Asia(16.66) | High-income Asia Pacific(1.80) | Australasia(3.75) | Western Sub-Saharan Africa(4.53) |
|  | Male | Caribbean(131.40) | South Asia(130.91) | Eastern Europe  (129.15) | High-income Asia Pacific(15.19) | Andean Latin America(24.73) | Australasia(25.18) |
| **Increase in the number of cases/years from1990 to 2017 (-fold)** | | | | | | | |
| Incidence | | | | | | | |
|  | Both | East Asia(190.73%) | Tropical Latin America (140.92%) | Oceania(134.75%) | Eastern Europe  (-6.88%) | Western Europe  (-3.93 %) | Central Asia (14.80 %) |
|  | Female | Tropical Latin America(142.30%) | Oceania (132.68%) | East Asia (112.13%) | Eastern Europe  (-2.71 %) | Eastern Sub-Saharan Africa(12.87 %) | High-income Asia Pacific(13.31 %) |
|  | Male | East Asia(212.59%) | Southeast Asia(140.75% ) | Tropical Latin America(140.71%) | Western Europe(-7.64%) | Eastern Europe  (-7.13%) | Central Asia(1.89%) |
| Death | | | | | | | |
|  | Both | Oceania(129.38%) | Tropical Latin America  (105.86%) | Caribbean(88.62%) | Western Europe  (-28.18%) | Eastern Europe  (-26.63%) | Central Europe(-6.10%) |
|  | Female | Oceania(125.09%) | Tropical Latin America  (102.42%) | Western Sub-Saharan Africa(68.73%) | High-income Asia Pacific (-29.86%) | Eastern Europe(-29.01%) | Western Europe  (-1.91%) |
|  | Male | Oceania(130.69%) | Tropical Latin America(106.37%) | Caribbean(96.32%) | Western Europe(-30.57%) | Eastern Europe(-26.50%) | Central Asia(-9.00%) |
| DALY | | | | | | | |
|  | Both | Oceania(126.74%) | Tropical Latin America(92.62%) | Caribbean(89.97%) | Western Europe(-36.72%) | Eastern Europe  (-32.03%) | High-income Asia Pacific(-22.42%) |
|  | Female | Oceania(124.81 %) | Tropical Latin America(84.17%) | Western Sub-Saharan Africa(71.99%) | High-income Asia Pacific(-42.42%) | Eastern Europe  (-26.36%) | Western Europe(-4.73%) |
|  | Male | Oceania(127.36%) | Caribbean(99.78%) | Tropical Latin America(93.82%) | Western Europe(-39.23%) | Eastern Europe  (-32.27%) | Southern Latin America(-19.27%) |
| **EAPC** | | | | | | | |
| Incidence | | | | | | | |
|  | Both | East Asia(1.01) | Caribbean (0.56) | Oceania (0.42) | Andean Latin America(-2.21) | Central Latin America(-1.88) | Southern Latin America(-1.83) |
|  | Female | Central Asia(0.92) | Central Europe(0.70) | Oceania(0.48) | Central Latin America(-3.19) | Eastern Sub-Saharan Africa(-2.74) | Andean Latin America  (-2.37) |
|  | Male | East Asia(1.18) | Caribbean(0.79) | Oceania(0.42) | Andean Latin America(-2.16) | Western Europe(-2.13) | Southern Latin America(-2.02) |
| Death | | | | | | | |
|  | Both | Oceania(0.37) | Caribbean(-0.16) | Western Sub-Saharan Africa(-0.36) | High-income Asia Pacific(-3.85) | Western Europe  (-2.98) | Australasia(-2.94) |
|  | Female | Oceania(0.38) | Central Asia(0.09) | Southern Latin America(-0.60) | High-income Asia Pacific(-5.11) | Central Latin America(-3.92) | Andean Latin America  (-3.13) |
|  | Male | Oceania(0.39) | Caribbean(0.08) | Western Sub-Saharan Africa(-0.15) | High-income Asia Pacific(-3.74) | Western Europe(-3.32) | Australasia(-3.14) |
| DALY | | | | | | | |
|  | Both | Oceania(0.30) | Caribbean(0.02) | Western Sub-Saharan Africa(-0.60) | High-income Asia Pacific(-3.97) | Western Europe(-3.27) | Australasia(-3.18) |
|  | Female | Oceania(0.33) | Central Asia(0.19) | Southern Latin America(-0.60) | High-income Asia Pacific(-4.51) | Central Latin America(-4.02) | Andean Latin America(-3.34) |
|  | Male | Oceania(0.30) | Caribbean (0.27) | Western Sub-Saharan Africa(-0.28) | High-income Asia Pacific(-4.01) | Western Europe(-3.58) | Australasia(-3.34) |

**Abbreviations:** ASDR, age standardized death rate; ASIR, age standardized incidence rate; DALY, disability adjusted life-year
