# Supplementary material for: Global burden of larynx cancer, 1990-2017: estimates from the global burden of disease 2017 study
Source: Aging (Albany NY). 2020 Feb 8;12(3):2545–83. doi: 10.18632/aging.102762 (PMC7041735; doi:10.18632/aging.102762)
Supplement: Supplementary Table 3 [file aging-12-102762-s003..docx]

**Supplementary Table 3. The incidence of larynx cancer among 195 countries and territories, and its temporal trends from 1990 to 2017.**

| **Location** | **Sex** | **1990** | | **2017** | | **1990-2017** | |
| --- | --- | --- | --- | --- | --- | --- | --- |
|  |  | **Incident cases No. (95% UI)** | **ASIR per 100,000 No. (95% UI)** | **Incident cases  No. (95% UI)** | **ASIR per 100,000 No. (95% UI)** | **Change in**  **absolute number of**  **Incident cases**  **No. (%)** | **EAPC No. (95% CI)** |
| Afghanistan | Both | 303.04 ( 164.32 - 413.62 ) | 4.1 ( 2.29 - 5.55 ) | 438.31 ( 346.01 - 547.46 ) | 3.78 ( 3.02 - 4.76 ) | 44.64 | -0.33 ( -0.55 - -0.12 ) |
| Albania | Both | 77.25 ( 71.19 - 84.09 ) | 3.44 ( 3.18 - 3.74 ) | 124.58 ( 97.94 - 157.08 ) | 2.98 ( 2.36 - 3.75 ) | 61.27 | -0.38 ( -0.64 - -0.13 ) |
| Algeria | Both | 298.8 ( 260.08 - 338.7 ) | 2.27 ( 1.98 - 2.55 ) | 600.7 ( 528.84 - 687.19 ) | 1.77 ( 1.56 - 2.03 ) | 101.04 | -0.85 ( -0.95 - -0.76 ) |
| American Samoa | Both | 0.41 ( 0.35 - 0.51 ) | 1.78 ( 1.51 - 2.27 ) | 0.68 ( 0.57 - 0.79 ) | 1.59 ( 1.34 - 1.86 ) | 65.62 | -0.21 ( -0.43 - 0.01 ) |
| Andorra | Both | 1.52 ( 1.13 - 2.12 ) | 2.54 ( 1.9 - 3.53 ) | 2.68 ( 2.13 - 3.45 ) | 2.03 ( 1.62 - 2.62 ) | 75.82 | -1.08 ( -1.23 - -0.93 ) |
| Angola | Both | 100.46 ( 66.63 - 131.82 ) | 2.4 ( 1.68 - 3.07 ) | 189.62 ( 153.23 - 228.29 ) | 1.71 ( 1.38 - 2.05 ) | 88.74 | -1.41 ( -1.51 - -1.32 ) |
| Antigua | Both | 1.15 ( 1.04 - 1.27 ) | 2.26 ( 2.03 - 2.5 ) | 2.33 ( 2.04 - 2.67 ) | 2.27 ( 1.99 - 2.59 ) | 102.64 | -0.03 ( -0.12 - 0.06 ) |
| Argentina | Both | 1230.77 ( 1165.12 - 1299.85 ) | 3.69 ( 3.49 - 3.9 ) | 1381.01 ( 1205.62 - 1598.48 ) | 2.64 ( 2.3 - 3.06 ) | 12.21 | -1.75 ( -2.01 - -1.48 ) |
| Armenia | Both | 137.13 ( 126.42 - 149.79 ) | 4.48 ( 4.14 - 4.86 ) | 144.48 ( 130.82 - 158.97 ) | 3.4 ( 3.09 - 3.74 ) | 5.36 | -1.46 ( -1.72 - -1.2 ) |
| Australia | Both | 610 ( 576.82 - 644.63 ) | 3.08 ( 2.91 - 3.26 ) | 837.65 ( 722.31 - 973.25 ) | 2.18 ( 1.87 - 2.54 ) | 37.32 | -1.65 ( -1.84 - -1.47 ) |
| Austria | Both | 345.48 ( 320.86 - 370.46 ) | 3.18 ( 2.94 - 3.43 ) | 344.34 ( 306.37 - 387.32 ) | 2.19 ( 1.94 - 2.47 ) | -0.33 | -1.72 ( -1.87 - -1.57 ) |
| Azerbaijan | Both | 159.92 ( 147.42 - 174.36 ) | 2.82 ( 2.61 - 3.06 ) | 303.69 ( 257.7 - 356.95 ) | 2.93 ( 2.51 - 3.42 ) | 89.9 | -0.84 ( -1.24 - -0.45 ) |
| Bahamas | Both | 5.9 ( 5.33 - 6.52 ) | 3.63 ( 3.29 - 4.01 ) | 14.3 ( 12.32 - 16.36 ) | 3.62 ( 3.15 - 4.13 ) | 142.44 | 0.15 ( 0.04 - 0.27 ) |
| Bahrain | Both | 4.04 ( 3.49 - 4.64 ) | 2.64 ( 2.28 - 3.04 ) | 8.53 ( 7.22 - 10.17 ) | 1.08 ( 0.9 - 1.28 ) | 111.14 | -4.79 ( -5.37 - -4.21 ) |
| Bangladesh | Both | 2631.77 ( 2249.12 - 3092.22 ) | 5.24 ( 4.49 - 6.17 ) | 3457.09 ( 2665.1 - 4158.07 ) | 2.73 ( 2.07 - 3.27 ) | 31.36 | -2.29 ( -2.41 - -2.18 ) |
| Barbados | Both | 5.74 ( 5.27 - 6.22 ) | 2.01 ( 1.84 - 2.18 ) | 10.55 ( 9.2 - 12.05 ) | 2.19 ( 1.92 - 2.5 ) | 83.93 | 0.23 ( 0.07 - 0.38 ) |
| Barbuda | Both | 1.15 ( 1.04 - 1.27 ) | 2.26 ( 2.03 - 2.5 ) | 2.33 ( 2.04 - 2.67 ) | 2.27 ( 1.99 - 2.59 ) | 102.64 | -0.03 ( -0.12 - 0.06 ) |
| Belarus | Both | 657.63 ( 617.96 - 699.43 ) | 4.92 ( 4.64 - 5.24 ) | 658.04 ( 567.02 - 757.26 ) | 4.21 ( 3.63 - 4.81 ) | 0.06 | -1.52 ( -1.94 - -1.1 ) |
| Belgium | Both | 816.45 ( 754.34 - 889.54 ) | 5.62 ( 5.18 - 6.14 ) | 617.66 ( 545.23 - 705.84 ) | 3.11 ( 2.74 - 3.56 ) | -24.35 | -2.57 ( -2.76 - -2.38 ) |
| Belize | Both | 1.5 ( 1.35 - 1.66 ) | 1.59 ( 1.43 - 1.75 ) | 5.56 ( 5.04 - 6.11 ) | 2.07 ( 1.88 - 2.27 ) | 270.63 | 0.8 ( 0.43 - 1.18 ) |
| Benin | Both | 26.88 ( 22.16 - 32.04 ) | 1.33 ( 1.1 - 1.58 ) | 60.52 ( 47 - 78.29 ) | 1.32 ( 1.04 - 1.69 ) | 125.12 | 0.26 ( 0.14 - 0.38 ) |
| Bermuda | Both | 2.47 ( 2.25 - 2.72 ) | 3.84 ( 3.5 - 4.21 ) | 4.75 ( 4.25 - 5.35 ) | 3.84 ( 3.43 - 4.33 ) | 92.03 | 0.31 ( 0.15 - 0.46 ) |
| Bhutan | Both | 11.96 ( 9.55 - 16.2 ) | 4.34 ( 3.44 - 5.9 ) | 16.79 ( 12.65 - 24.34 ) | 2.69 ( 2.04 - 3.93 ) | 40.32 | -1.89 ( -2.02 - -1.76 ) |
| Bolivia | Both | 70.85 ( 59.9 - 81.67 ) | 2.14 ( 1.81 - 2.46 ) | 119.94 ( 94.48 - 152.36 ) | 1.4 ( 1.11 - 1.78 ) | 69.28 | -1.69 ( -1.81 - -1.57 ) |
| Bosnia and Herzegovina | Both | 196.03 ( 182.01 - 210.48 ) | 4.36 ( 4.07 - 4.66 ) | 199.48 ( 175.81 - 225.52 ) | 3.27 ( 2.89 - 3.68 ) | 1.76 | -1.76 ( -2.08 - -1.44 ) |
| Botswana | Both | 14.26 ( 11.74 - 17.43 ) | 2.31 ( 1.93 - 2.8 ) | 20.57 ( 16.83 - 27.8 ) | 1.49 ( 1.23 - 1.97 ) | 44.21 | -1.64 ( -1.73 - -1.56 ) |
| Brazil | Both | 2973.47 ( 2894.89 - 3069.69 ) | 3.12 ( 3.04 - 3.22 ) | 7150.69 ( 6927.02 - 7392.82 ) | 3.06 ( 2.96 - 3.16 ) | 140.48 | -0.2 ( -0.28 - -0.12 ) |
| Brunei | Both | 4.34 ( 3.84 - 4.96 ) | 4.39 ( 3.87 - 5.02 ) | 6.17 ( 5.29 - 7.16 ) | 2.01 ( 1.73 - 2.34 ) | 42.05 | -3.62 ( -3.95 - -3.29 ) |
| Bulgaria | Both | 469.82 ( 441.69 - 500.74 ) | 3.68 ( 3.46 - 3.93 ) | 678.3 ( 601.44 - 762.92 ) | 5.33 ( 4.7 - 6.02 ) | 44.38 | 1.36 ( 1.11 - 1.62 ) |
| Burkina Faso | Both | 62.83 ( 47.55 - 79.84 ) | 1.41 ( 1.07 - 1.78 ) | 134.17 ( 96.7 - 165.41 ) | 1.53 ( 1.11 - 1.86 ) | 113.55 | 0.53 ( 0.42 - 0.65 ) |
| Burundi | Both | 74.35 ( 56.71 - 92.56 ) | 3.06 ( 2.37 - 3.78 ) | 81.43 ( 60.3 - 105.12 ) | 1.77 ( 1.34 - 2.24 ) | 9.51 | -2.49 ( -2.71 - -2.27 ) |
| Cambodia | Both | 129.38 ( 106.83 - 155.36 ) | 2.7 ( 2.24 - 3.26 ) | 238.58 ( 193.46 - 309.68 ) | 2.06 ( 1.69 - 2.72 ) | 84.41 | -1.03 ( -1.12 - -0.94 ) |
| Cameroon | Both | 70.89 ( 57.52 - 86.11 ) | 1.54 ( 1.26 - 1.86 ) | 190.48 ( 142.66 - 249.86 ) | 1.67 ( 1.26 - 2.19 ) | 168.7 | 0.57 ( 0.39 - 0.76 ) |
| Canada | Both | 1213.1 ( 1115.87 - 1318.21 ) | 3.72 ( 3.43 - 4.05 ) | 1574.79 ( 1387.41 - 1788.86 ) | 2.43 ( 2.15 - 2.77 ) | 29.82 | -1.93 ( -2.13 - -1.73 ) |
| Cape Verde | Both | 3.22 ( 2.71 - 3.76 ) | 1.39 ( 1.17 - 1.63 ) | 4.63 ( 4.05 - 5.25 ) | 1.05 ( 0.92 - 1.19 ) | 43.66 | -1.12 ( -1.28 - -0.96 ) |
| Central African Republic | Both | 33.2 ( 20.95 - 42.29 ) | 2.64 ( 1.75 - 3.3 ) | 48.59 ( 32.63 - 64.68 ) | 2.11 ( 1.53 - 2.76 ) | 46.38 | -0.96 ( -1.05 - -0.87 ) |
| Chad | Both | 32.43 ( 22.89 - 40.07 ) | 1.13 ( 0.79 - 1.39 ) | 83.71 ( 61.23 - 104.21 ) | 1.57 ( 1.17 - 1.95 ) | 158.13 | 1.64 ( 1.44 - 1.83 ) |
| Chile | Both | 175 ( 163.99 - 186.36 ) | 1.71 ( 1.6 - 1.82 ) | 306.56 ( 266.09 - 352.85 ) | 1.31 ( 1.14 - 1.51 ) | 75.17 | -0.97 ( -1.18 - -0.75 ) |
| China | Both | 13545.56 ( 13009.64 - 14267.67 ) | 1.52 ( 1.46 - 1.6 ) | 39725.4 ( 37831.08 - 41844.01 ) | 1.98 ( 1.89 - 2.09 ) | 193.27 | 1.03 ( 0.63 - 1.44 ) |
| Colombia | Both | 458.68 ( 438.09 - 486.37 ) | 2.58 ( 2.47 - 2.73 ) | 723.55 ( 633.51 - 825.98 ) | 1.34 ( 1.17 - 1.53 ) | 57.75 | -3.25 ( -3.53 - -2.98 ) |
| Comoros | Both | 4.94 ( 3.85 - 6.39 ) | 2.23 ( 1.76 - 2.85 ) | 6.64 ( 5 - 9.24 ) | 1.4 ( 1.06 - 1.92 ) | 34.48 | -2 ( -2.19 - -1.8 ) |
| Costa Rica | Both | 42.7 ( 39.79 - 45.89 ) | 2.4 ( 2.24 - 2.58 ) | 93.67 ( 81.79 - 105.67 ) | 1.9 ( 1.66 - 2.14 ) | 119.35 | -1.14 ( -1.46 - -0.82 ) |
| Croatia | Both | 421.65 ( 393.69 - 449.39 ) | 6.25 ( 5.85 - 6.66 ) | 341.19 ( 303.14 - 381.2 ) | 4.28 ( 3.8 - 4.82 ) | -19.08 | -1.51 ( -1.82 - -1.2 ) |
| Cuba | Both | 598 ( 565.09 - 630.51 ) | 5.73 ( 5.41 - 6.04 ) | 1589.96 ( 1366.28 - 1824.16 ) | 8.58 ( 7.37 - 9.83 ) | 165.88 | 1.54 ( 1.43 - 1.65 ) |
| Cyprus | Both | 17.76 ( 15.63 - 20.28 ) | 2.09 ( 1.85 - 2.38 ) | 40.41 ( 33.79 - 47.6 ) | 2.14 ( 1.81 - 2.51 ) | 127.49 | 0.27 ( -0.01 - 0.54 ) |
| Czech Republic | Both | 505.35 ( 475.77 - 533.09 ) | 3.75 ( 3.53 - 3.98 ) | 534.8 ( 478.26 - 599.13 ) | 2.83 ( 2.53 - 3.16 ) | 5.83 | -1.03 ( -1.11 - -0.94 ) |
| Democratic Republic of the Congo | Both | 288.68 ( 223.59 - 365.65 ) | 1.77 ( 1.41 - 2.19 ) | 509.64 ( 349.14 - 653.95 ) | 1.44 ( 1.03 - 1.82 ) | 76.54 | -0.86 ( -0.97 - -0.74 ) |
| Denmark | Both | 289.4 ( 271.09 - 309.55 ) | 3.83 ( 3.57 - 4.1 ) | 277.36 ( 247.55 - 311.85 ) | 2.61 ( 2.33 - 2.93 ) | -4.16 | -1.68 ( -1.81 - -1.56 ) |
| Djibouti | Both | 3.72 ( 2.45 - 5.63 ) | 2.14 ( 1.45 - 3.2 ) | 10.08 ( 6.7 - 18.15 ) | 1.63 ( 1.11 - 2.9 ) | 171.07 | -1.37 ( -1.55 - -1.2 ) |
| Dominica | Both | 1.67 ( 1.53 - 1.81 ) | 2.34 ( 2.15 - 2.52 ) | 2.82 ( 2.52 - 3.13 ) | 3.09 ( 2.76 - 3.44 ) | 68.79 | 0.99 ( 0.89 - 1.09 ) |
| Dominican Republic | Both | 71.55 ( 64.35 - 80.14 ) | 1.84 ( 1.65 - 2.06 ) | 182.77 ( 151.6 - 217.13 ) | 1.98 ( 1.64 - 2.35 ) | 155.46 | 0.58 ( 0.17 - 1 ) |
| Ecuador | Both | 66.17 ( 62.6 - 70.05 ) | 1.2 ( 1.13 - 1.27 ) | 138.08 ( 122.71 - 155.49 ) | 0.94 ( 0.84 - 1.06 ) | 108.67 | -0.75 ( -1.06 - -0.44 ) |
| Egypt | Both | 305.23 ( 278.21 - 337.31 ) | 0.97 ( 0.88 - 1.06 ) | 698.52 ( 466.52 - 856.43 ) | 1.05 ( 0.7 - 1.29 ) | 128.85 | 0.44 ( 0.27 - 0.62 ) |
| El Salvador | Both | 30.3 ( 27.49 - 33.5 ) | 0.99 ( 0.9 - 1.1 ) | 66.19 ( 54.18 - 81.01 ) | 1.16 ( 0.95 - 1.43 ) | 118.44 | 0.83 ( 0.55 - 1.1 ) |
| Equatorial Guinea | Both | 5.3 ( 3.03 - 7.23 ) | 2.52 ( 1.49 - 3.35 ) | 6.37 ( 4.42 - 8.83 ) | 1.37 ( 0.97 - 1.86 ) | 20.07 | -2.75 ( -3.18 - -2.31 ) |
| Eritrea | Both | 36.33 ( 25.75 - 47.44 ) | 3.17 ( 2.36 - 4.06 ) | 52.64 ( 40.32 - 78.65 ) | 1.94 ( 1.51 - 2.86 ) | 44.88 | -2.3 ( -2.52 - -2.08 ) |
| Estonia | Both | 78.19 ( 72.71 - 84.77 ) | 3.75 ( 3.49 - 4.06 ) | 72.69 ( 60.48 - 88.3 ) | 3.21 ( 2.67 - 3.93 ) | -7.03 | -0.9 ( -1.2 - -0.6 ) |
| Ethiopia | Both | 358.86 ( 272.83 - 468.44 ) | 1.61 ( 1.24 - 2.08 ) | 371.78 ( 286.07 - 577.43 ) | 0.9 ( 0.69 - 1.41 ) | 3.6 | -2.49 ( -2.61 - -2.38 ) |
| Fiji | Both | 4.01 ( 3.47 - 4.63 ) | 1.09 ( 0.95 - 1.25 ) | 9.67 ( 8.23 - 11.42 ) | 1.34 ( 1.15 - 1.56 ) | 141.24 | 1.17 ( 0.96 - 1.38 ) |
| Finland | Both | 130.67 ( 118.76 - 144.38 ) | 1.88 ( 1.7 - 2.07 ) | 150.96 ( 131.42 - 174.27 ) | 1.4 ( 1.21 - 1.61 ) | 15.53 | -1.04 ( -1.22 - -0.87 ) |
| France | Both | 6268.57 ( 5823.99 - 6808.97 ) | 8.27 ( 7.67 - 9 ) | 4643.05 ( 4107.96 - 5262.98 ) | 4.08 ( 3.6 - 4.64 ) | -25.93 | -2.91 ( -3.35 - -2.47 ) |
| Gabon | Both | 13.85 ( 11.43 - 17 ) | 2.33 ( 1.93 - 2.86 ) | 20.09 ( 16.18 - 29.03 ) | 1.86 ( 1.51 - 2.73 ) | 45.07 | -0.83 ( -0.89 - -0.77 ) |
| Gambia | Both | 3.03 ( 2.36 - 3.85 ) | 0.83 ( 0.65 - 1.04 ) | 251.16 ( 225.13 - 279.08 ) | 0.75 ( 0.6 - 0.97 ) | 8194.42 | -0.19 ( -0.25 - -0.12 ) |
| Georgia | Both | 274.97 ( 254.43 - 296.78 ) | 4.16 ( 3.86 - 4.49 ) | 4655.96 ( 3968.92 - 5359.95 ) | 4.42 ( 3.96 - 4.87 ) | 1593.27 | 0.36 ( -0.2 - 0.93 ) |
| Germany | Both | 3844.5 ( 3587.64 - 4117.56 ) | 3.24 ( 3.02 - 3.48 ) | 250.68 ( 163.09 - 310.12 ) | 2.87 ( 2.45 - 3.31 ) | -93.48 | -0.99 ( -1.21 - -0.77 ) |
| Ghana | Both | 84.05 ( 62.29 - 105.16 ) | 1.29 ( 0.96 - 1.6 ) | 880.94 ( 783.74 - 994.5 ) | 1.62 ( 1.09 - 1.98 ) | 948.15 | 1.45 ( 1.18 - 1.71 ) |
| Greece | Both | 669.38 ( 618.65 - 727.6 ) | 4.26 ( 3.94 - 4.61 ) | 1.74 ( 1.54 - 1.97 ) | 4.19 ( 3.72 - 4.77 ) | -99.74 | 0.15 ( 0 - 0.3 ) |
| Greenland | Both | 0.88 ( 0.79 - 1 ) | 2.45 ( 2.19 - 2.74 ) | 3.45 ( 3.1 - 3.82 ) | 2.48 ( 2.19 - 2.81 ) | 290.62 | 0.08 ( -0.23 - 0.39 ) |
| Grenada | Both | 1.5 ( 1.39 - 1.64 ) | 2.17 ( 2 - 2.35 ) | 4.26 ( 3.68 - 4.87 ) | 2.42 ( 2.16 - 2.68 ) | 183.23 | 0.71 ( 0.37 - 1.04 ) |
| Grenadines | Both | 1.96 ( 1.78 - 2.14 ) | 2.66 ( 2.41 - 2.89 ) | 98.33 ( 87.46 - 110.39 ) | 3.55 ( 3.2 - 3.96 ) | 4908.84 | 1.03 ( 0.87 - 1.2 ) |
| Guam | Both | 1.81 ( 1.53 - 2.34 ) | 2.25 ( 1.93 - 2.89 ) | 87.82 ( 66.86 - 110.17 ) | 2.35 ( 2.05 - 2.68 ) | 4760.44 | -0.09 ( -0.38 - 0.2 ) |
| Guatemala | Both | 49.43 ( 46.31 - 52.73 ) | 1.32 ( 1.24 - 1.41 ) | 12.4 ( 9.59 - 15.48 ) | 0.89 ( 0.8 - 1 ) | -74.91 | -2.06 ( -2.43 - -1.69 ) |
| Guinea | Both | 39.71 ( 33.1 - 47.75 ) | 1.18 ( 0.99 - 1.41 ) | 8.53 ( 7.23 - 9.9 ) | 1.65 ( 1.27 - 2.06 ) | -78.52 | 1.82 ( 1.61 - 2.03 ) |
| Guinea-Bissau | Both | 8.44 ( 6.02 - 10.56 ) | 2.03 ( 1.48 - 2.51 ) | 206.26 ( 152.15 - 307.07 ) | 1.75 ( 1.38 - 2.15 ) | 2344.13 | -0.24 ( -0.38 - -0.1 ) |
| Guyana | Both | 5.11 ( 4.72 - 5.56 ) | 1.28 ( 1.19 - 1.39 ) | 66.66 ( 51.6 - 83.05 ) | 1.32 ( 1.13 - 1.53 ) | 1205.38 | 0.5 ( 0.25 - 0.76 ) |
| Haiti | Both | 127.33 ( 96.78 - 172.88 ) | 3.8 ( 2.9 - 5.22 ) | 905.38 ( 819.05 - 1011.2 ) | 3.09 ( 2.3 - 4.68 ) | 611.03 | -0.66 ( -0.76 - -0.56 ) |
| Honduras | Both | 26.16 ( 22.36 - 30.35 ) | 1.16 ( 1 - 1.34 ) | 8.53 ( 7.55 - 9.67 ) | 1.1 ( 0.86 - 1.37 ) | -67.4 | -0.3 ( -0.4 - -0.2 ) |
| Hungary | Both | 856.9 ( 809.32 - 906.49 ) | 5.94 ( 5.6 - 6.29 ) | 39748.99 ( 37627.69 - 41927.79 ) | 5.4 ( 4.86 - 6.05 ) | 4538.71 | -0.41 ( -0.77 - -0.04 ) |
| Iceland | Both | 5.89 ( 5.27 - 6.56 ) | 2.13 ( 1.9 - 2.37 ) | 3634.29 ( 2961.77 - 5613.74 ) | 1.69 ( 1.5 - 1.91 ) | 61611.68 | -1.43 ( -1.8 - -1.07 ) |
| India | Both | 23306.95 ( 20482.75 - 25481.22 ) | 4.44 ( 3.91 - 4.85 ) | 2080.64 ( 1948.11 - 2222.88 ) | 3.47 ( 3.28 - 3.66 ) | -91.07 | -1.09 ( -1.36 - -0.81 ) |
| Indonesia | Both | 1761.54 ( 1518.17 - 2336.63 ) | 1.69 ( 1.46 - 2.23 ) | 334.73 ( 303.88 - 367.8 ) | 1.7 ( 1.4 - 2.6 ) | -81 | 0.09 ( 0.05 - 0.14 ) |
| Iran | Both | 842.26 ( 782.74 - 957.08 ) | 3.02 ( 2.81 - 3.45 ) | 186.18 ( 162.59 - 212.19 ) | 2.94 ( 2.76 - 3.12 ) | -77.89 | 0.17 ( -0.09 - 0.44 ) |
| Iraq | Both | 303.13 ( 248.75 - 362.79 ) | 3.68 ( 3.04 - 4.4 ) | 174.97 ( 154.5 - 198.67 ) | 1.41 ( 1.28 - 1.54 ) | -42.28 | -3.87 ( -4.42 - -3.33 ) |
| Ireland | Both | 112.99 ( 103.45 - 123.59 ) | 2.77 ( 2.53 - 3.02 ) | 4508.55 ( 4016.53 - 5113.09 ) | 2.64 ( 2.31 - 3.01 ) | 3890.22 | -0.09 ( -0.21 - 0.03 ) |
| Israel | Both | 68.92 ( 62.98 - 75.99 ) | 1.42 ( 1.3 - 1.57 ) | 155.54 ( 118.73 - 203.83 ) | 1.61 ( 1.42 - 1.82 ) | 125.67 | -0.22 ( -0.6 - 0.15 ) |
| Italy | Both | 5367.47 ( 4988.77 - 5833.26 ) | 6.1 ( 5.66 - 6.61 ) | 67.51 ( 52.19 - 82.98 ) | 3.64 ( 3.23 - 4.14 ) | -98.74 | -1.88 ( -1.93 - -1.82 ) |
| Ivory Coast | Both | 70.8 ( 57.72 - 85.65 ) | 1.56 ( 1.28 - 1.86 ) | 4966.52 ( 4679.76 - 5258.74 ) | 1.42 ( 1.1 - 1.86 ) | 6914.37 | -0.47 ( -0.68 - -0.27 ) |
| Jamaica | Both | 25.86 ( 23.62 - 28.29 ) | 1.44 ( 1.32 - 1.58 ) | 56.95 ( 48.62 - 68.87 ) | 2.35 ( 1.83 - 2.89 ) | 120.25 | 1.17 ( 0.63 - 1.71 ) |
| Japan | Both | 3485.17 ( 3328.3 - 3641.63 ) | 1.98 ( 1.9 - 2.07 ) | 357.72 ( 323.65 - 400.37 ) | 1.52 ( 1.44 - 1.61 ) | -89.74 | -1.09 ( -1.17 - -1.01 ) |
| Jordan | Both | 34.37 ( 28.21 - 41.3 ) | 2.23 ( 1.85 - 2.7 ) | 420.06 ( 302.32 - 486.95 ) | 0.95 ( 0.81 - 1.14 ) | 1122.35 | -4.03 ( -4.55 - -3.51 ) |
| Kazakhstan | Both | 530.64 ( 500.73 - 562.62 ) | 3.74 ( 3.55 - 3.96 ) | 0.7 ( 0.6 - 0.81 ) | 1.97 ( 1.79 - 2.19 ) | -99.87 | -3.03 ( -3.3 - -2.77 ) |
| Kenya | Both | 158.39 ( 99.39 - 192.73 ) | 1.77 ( 1.1 - 2.15 ) | 22.98 ( 20.33 - 26.14 ) | 1.81 ( 1.3 - 2.1 ) | -85.49 | 0.13 ( -0.1 - 0.37 ) |
| Kiribati | Both | 0.39 ( 0.35 - 0.43 ) | 1.05 ( 0.95 - 1.16 ) | 42.87 ( 38.62 - 48.19 ) | 1.04 ( 0.9 - 1.19 ) | 10835.3 | -0.14 ( -0.34 - 0.07 ) |
| Kuwait | Both | 11.24 ( 10.17 - 12.32 ) | 1.61 ( 1.46 - 1.76 ) | 79.5 ( 59.13 - 125.5 ) | 0.95 ( 0.84 - 1.06 ) | 607 | -1.56 ( -2.01 - -1.1 ) |
| Kyrgyzstan | Both | 76.26 ( 69.42 - 83.5 ) | 2.35 ( 2.15 - 2.58 ) | 151.35 ( 127.06 - 179.67 ) | 0.92 ( 0.83 - 1.03 ) | 98.47 | -3.6 ( -3.95 - -3.24 ) |
| Laos | Both | 63.52 ( 51.46 - 78.36 ) | 2.86 ( 2.31 - 3.5 ) | 293.89 ( 256.72 - 337.78 ) | 1.87 ( 1.41 - 2.99 ) | 362.69 | -1.68 ( -1.75 - -1.6 ) |
| Latvia | Both | 152.37 ( 141.88 - 163.22 ) | 4.13 ( 3.84 - 4.43 ) | 34.4 ( 27.43 - 42.58 ) | 4.36 ( 3.66 - 5.22 ) | -77.42 | 0.21 ( -0.19 - 0.61 ) |
| Lebanon | Both | 122.22 ( 99.9 - 153.48 ) | 5.17 ( 4.23 - 6.48 ) | 23.22 ( 17.27 - 29.78 ) | 4.75 ( 4.15 - 5.43 ) | -81 | -0.26 ( -0.62 - 0.1 ) |
| Lesotho | Both | 28.99 ( 20.33 - 35.44 ) | 2.75 ( 1.93 - 3.35 ) | 221 ( 155.52 - 275.37 ) | 2.74 ( 2.19 - 3.39 ) | 662.37 | 0.35 ( 0.04 - 0.66 ) |
| Liberia | Both | 15.29 ( 12.29 - 18.97 ) | 1.33 ( 1.07 - 1.65 ) | 273.09 ( 242.43 - 310.62 ) | 1.24 ( 0.93 - 1.59 ) | 1686.65 | -0.15 ( -0.31 - 0 ) |
| Libya | Both | 78.9 ( 63.54 - 99.3 ) | 3.9 ( 3.16 - 4.88 ) | 32.49 ( 27.6 - 37.92 ) | 4.45 ( 3.13 - 5.53 ) | -58.83 | 0.63 ( 0.52 - 0.75 ) |
| Lithuania | Both | 220.83 ( 207.12 - 237.1 ) | 4.75 ( 4.46 - 5.11 ) | 155.6 ( 135.34 - 179.98 ) | 5.44 ( 4.82 - 6.17 ) | -29.54 | 0.37 ( -0.03 - 0.78 ) |
| Luxembourg | Both | 28.45 ( 25.52 - 31.75 ) | 5.28 ( 4.73 - 5.89 ) | 159.34 ( 124.64 - 199.92 ) | 3.57 ( 3.01 - 4.17 ) | 460.04 | -1.75 ( -1.88 - -1.63 ) |
| Macedonia | Both | 76.62 ( 69.22 - 86.07 ) | 3.81 ( 3.45 - 4.27 ) | 59.1 ( 50.02 - 70.2 ) | 4.63 ( 4.04 - 5.35 ) | -22.86 | 0.6 ( 0.39 - 0.81 ) |
| Madagascar | Both | 106.92 ( 90.46 - 125.87 ) | 1.9 ( 1.62 - 2.21 ) | 544.89 ( 430.16 - 656.23 ) | 1.35 ( 1.07 - 1.67 ) | 409.61 | -1.39 ( -1.49 - -1.28 ) |
| Malawi | Both | 39.41 ( 22.88 - 51.17 ) | 0.9 ( 0.56 - 1.15 ) | 4.05 ( 3.49 - 4.7 ) | 0.76 ( 0.65 - 0.9 ) | -89.73 | -1.13 ( -1.48 - -0.78 ) |
| Malaysia | Both | 196.33 ( 174.25 - 224.13 ) | 2.12 ( 1.88 - 2.42 ) | 76.56 ( 61.19 - 94.5 ) | 2.11 ( 1.68 - 2.52 ) | -61 | -0.43 ( -0.79 - -0.08 ) |
| Maldives | Both | 1.95 ( 1.42 - 2.32 ) | 2.19 ( 1.7 - 2.56 ) | 23.31 ( 20.48 - 26.48 ) | 1.45 ( 1.24 - 1.69 ) | 1098.04 | -1.97 ( -2.14 - -1.81 ) |
| Mali | Both | 52.18 ( 44.95 - 60.76 ) | 1.21 ( 1.03 - 1.4 ) | 0.79 ( 0.64 - 0.96 ) | 0.9 ( 0.72 - 1.11 ) | -98.49 | -1 ( -1.2 - -0.79 ) |
| Malta | Both | 14.81 ( 13.37 - 16.41 ) | 3.38 ( 3.06 - 3.74 ) | 22.86 ( 16.75 - 31.28 ) | 2.82 ( 2.48 - 3.18 ) | 54.37 | -1.11 ( -1.3 - -0.91 ) |
| Marshall Islands | Both | 0.37 ( 0.26 - 0.44 ) | 2.14 ( 1.55 - 2.55 ) | 33.26 ( 29.14 - 37.82 ) | 2.43 ( 2 - 2.89 ) | 8964.02 | 0.43 ( 0.28 - 0.58 ) |
| Mauritania | Both | 13.38 ( 10.88 - 16.14 ) | 1.28 ( 1.05 - 1.54 ) | 1479.68 ( 1365.77 - 1532.04 ) | 1.17 ( 0.87 - 1.59 ) | 10959.9 | -0.07 ( -0.29 - 0.16 ) |
| Mauritius | Both | 21.9 ( 20.02 - 23.79 ) | 2.83 ( 2.6 - 3.06 ) | 1.25 ( 1 - 1.51 ) | 1.91 ( 1.68 - 2.15 ) | -94.28 | -1.91 ( -2.12 - -1.71 ) |
| Mexico | Both | 874.94 ( 853.71 - 906.61 ) | 2.02 ( 1.98 - 2.1 ) | 237.42 ( 215.99 - 262.23 ) | 1.31 ( 1.21 - 1.36 ) | -72.86 | -2.11 ( -2.3 - -1.92 ) |
| Micronesia | Both | 0.94 ( 0.77 - 1.2 ) | 1.89 ( 1.56 - 2.47 ) | 25.46 ( 21.88 - 30.22 ) | 1.77 ( 1.46 - 2.11 ) | 2619.75 | -0.25 ( -0.29 - -0.21 ) |
| Moldova | Both | 183.13 ( 171.44 - 195.39 ) | 3.82 ( 3.58 - 4.07 ) | 69.85 ( 60.68 - 80.48 ) | 4.12 ( 3.75 - 4.55 ) | -61.86 | 0.46 ( 0.07 - 0.85 ) |
| Mongolia | Both | 9.79 ( 8.83 - 10.84 ) | 0.97 ( 0.88 - 1.08 ) | 985.89 ( 768.51 - 1261.7 ) | 1.37 ( 1.19 - 1.62 ) | 9970.9 | 1.82 ( 1.34 - 2.31 ) |
| Montenegro | Both | 43.11 ( 37.91 - 49.5 ) | 6.42 ( 5.67 - 7.33 ) | 241.84 ( 161.32 - 307.06 ) | 7.1 ( 6.17 - 8.18 ) | 460.92 | 0.34 ( 0.18 - 0.5 ) |
| Morocco | Both | 452.68 ( 386.57 - 538.17 ) | 3.01 ( 2.57 - 3.6 ) | 914.31 ( 753.02 - 1243.4 ) | 2.97 ( 2.32 - 3.79 ) | 101.98 | -0.14 ( -0.21 - -0.07 ) |
| Mozambique | Both | 155.67 ( 112.68 - 197.64 ) | 2.3 ( 1.67 - 2.87 ) | 40.5 ( 34.21 - 48.08 ) | 1.98 ( 1.36 - 2.47 ) | -73.99 | -0.45 ( -0.55 - -0.35 ) |
| Myanmar | Both | 754.48 ( 601.33 - 921.15 ) | 3.07 ( 2.46 - 3.72 ) | 734.54 ( 589.52 - 886.81 ) | 2.01 ( 1.67 - 2.73 ) | -2.64 | -1.62 ( -1.65 - -1.58 ) |
| Namibia | Both | 27.98 ( 23.31 - 33.32 ) | 3.64 ( 3.05 - 4.32 ) | 791.55 ( 707.76 - 887.77 ) | 2.77 ( 2.36 - 3.27 ) | 2728.63 | -1.35 ( -1.8 - -0.89 ) |
| Nepal | Both | 490.39 ( 390.74 - 602.55 ) | 4.68 ( 3.74 - 5.74 ) | 115.07 ( 101.38 - 130.34 ) | 3.28 ( 2.66 - 3.96 ) | -76.53 | -1.32 ( -1.7 - -0.95 ) |
| Netherlands | Both | 609.85 ( 560.89 - 660.46 ) | 3.11 ( 2.87 - 3.38 ) | 42.35 ( 35.87 - 49.91 ) | 2.49 ( 2.22 - 2.79 ) | -93.06 | -1.15 ( -1.3 - -0.99 ) |
| New Zealand | Both | 104.87 ( 93.94 - 116.98 ) | 2.64 ( 2.37 - 2.95 ) | 85.34 ( 48.92 - 117.5 ) | 1.58 ( 1.39 - 1.79 ) | -18.62 | -2.26 ( -2.43 - -2.09 ) |
| Nicaragua | Both | 23.18 ( 20.72 - 25.93 ) | 1.45 ( 1.3 - 1.63 ) | 1043.05 ( 744.12 - 1479.13 ) | 0.94 ( 0.8 - 1.11 ) | 4400.55 | -1.8 ( -1.99 - -1.61 ) |
| Niger | Both | 35.71 ( 23.54 - 46.79 ) | 1.22 ( 0.81 - 1.57 ) | 503.95 ( 399.76 - 623.25 ) | 1.18 ( 0.68 - 1.59 ) | 1311.1 | -0.01 ( -0.15 - 0.14 ) |
| Nigeria | Both | 758.59 ( 550.04 - 1021.22 ) | 1.61 ( 1.18 - 2.14 ) | 1.72 ( 1.46 - 2.03 ) | 1.21 ( 0.89 - 1.69 ) | -99.77 | -1.2 ( -1.29 - -1.12 ) |
| North Korea | Both | 261.82 ( 201.94 - 327.54 ) | 1.46 ( 1.15 - 1.81 ) | 134.65 ( 122.13 - 147.02 ) | 1.56 ( 1.24 - 1.93 ) | -48.57 | 0.3 ( 0.23 - 0.38 ) |
| Northern Mariana Islands | Both | 0.6 ( 0.47 - 0.83 ) | 3.28 ( 2.65 - 4.52 ) | 25.06 ( 19.19 - 31.82 ) | 3.33 ( 2.85 - 3.89 ) | 4098.49 | 0.23 ( 0.03 - 0.43 ) |
| Norway | Both | 131.65 ( 121.05 - 143.02 ) | 2.07 ( 1.9 - 2.26 ) | 6600 ( 5214.06 - 8124.12 ) | 1.52 ( 1.38 - 1.66 ) | 4913.37 | -1.42 ( -1.55 - -1.28 ) |
| Oman | Both | 10.36 ( 8.02 - 13.19 ) | 1.4 ( 1.09 - 1.76 ) | 22.24 ( 19.53 - 25.43 ) | 1.14 ( 0.88 - 1.42 ) | 114.59 | -0.82 ( -0.96 - -0.69 ) |
| Pakistan | Both | 3303.04 ( 2892.23 - 3743.82 ) | 5.55 ( 4.85 - 6.31 ) | 61.5 ( 54.61 - 68.98 ) | 5.5 ( 4.39 - 6.74 ) | -98.14 | -0.27 ( -0.48 - -0.06 ) |
| Palestine | Both | 12.32 ( 9.21 - 15.33 ) | 1.39 ( 1.03 - 1.72 ) | 103.03 ( 85 - 126.72 ) | 0.9 ( 0.79 - 1.04 ) | 735.98 | -1.86 ( -2.04 - -1.69 ) |
| Panama | Both | 38.51 ( 35.72 - 41.67 ) | 2.52 ( 2.34 - 2.72 ) | 95.94 ( 75.71 - 119.1 ) | 1.55 ( 1.38 - 1.74 ) | 149.16 | -2.31 ( -2.62 - -2 ) |
| Papua New Guinea | Both | 42.16 ( 34.68 - 51.48 ) | 2.12 ( 1.78 - 2.5 ) | 224.56 ( 190.44 - 261.18 ) | 2.22 ( 1.86 - 2.67 ) | 432.69 | 0.35 ( 0.26 - 0.44 ) |
| Paraguay | Both | 34.41 ( 29.78 - 40.69 ) | 1.51 ( 1.31 - 1.78 ) | 974.57 ( 826.23 - 1139.88 ) | 1.78 ( 1.4 - 2.21 ) | 2732.39 | 0.69 ( 0.56 - 0.82 ) |
| Peru | Both | 175.58 ( 157.31 - 201.51 ) | 1.42 ( 1.27 - 1.64 ) | 2780.33 ( 2511.21 - 3077.24 ) | 0.73 ( 0.62 - 0.85 ) | 1483.56 | -3.1 ( -3.43 - -2.76 ) |
| Philippines | Both | 415.21 ( 385.46 - 446.7 ) | 1.29 ( 1.2 - 1.38 ) | 559.25 ( 501.32 - 627.61 ) | 1.33 ( 1.13 - 1.55 ) | 34.69 | 0.13 ( 0.02 - 0.23 ) |
| Poland | Both | 2287.24 ( 2183.49 - 2394.94 ) | 5.07 ( 4.84 - 5.31 ) | 140.82 ( 125.1 - 157.36 ) | 4.21 ( 3.8 - 4.65 ) | -93.84 | -1.06 ( -1.28 - -0.84 ) |
| Portugal | Both | 528.34 ( 502.59 - 556.2 ) | 3.81 ( 3.62 - 4.01 ) | 17.71 ( 13.84 - 22.06 ) | 2.8 ( 2.49 - 3.15 ) | -96.65 | -1.36 ( -1.56 - -1.16 ) |
| Puerto Rico | Both | 144.61 ( 134.84 - 155.69 ) | 3.9 ( 3.63 - 4.2 ) | 46.13 ( 36.25 - 63.65 ) | 2.16 ( 1.92 - 2.42 ) | -68.1 | -2.65 ( -2.85 - -2.45 ) |
| Qatar | Both | 1.67 ( 1.34 - 2.02 ) | 1.61 ( 1.31 - 1.94 ) | 1667.8 ( 1514.31 - 1843.11 ) | 2.05 ( 1.61 - 2.55 ) | 99766.43 | 1.63 ( 0.73 - 2.53 ) |
| Republic of Congo | Both | 29.77 ( 23.94 - 35.72 ) | 2.58 ( 2.11 - 3.05 ) | 7685.13 ( 7374.71 - 8014.09 ) | 1.82 ( 1.43 - 2.57 ) | 25717.46 | -1.56 ( -1.73 - -1.39 ) |
| Romania | Both | 1081.56 ( 1029.24 - 1143.01 ) | 3.68 ( 3.5 - 3.88 ) | 84.45 ( 68.96 - 105 ) | 5.09 ( 4.6 - 5.63 ) | -92.19 | 1.04 ( 0.82 - 1.27 ) |
| Russia | Both | 8263.08 ( 7872.04 - 8848.18 ) | 4.32 ( 4.12 - 4.64 ) | 6.13 ( 5.47 - 6.82 ) | 3.34 ( 3.21 - 3.48 ) | -99.93 | -1.62 ( -1.99 - -1.26 ) |
| Rwanda | Both | 94.46 ( 75.1 - 113.83 ) | 2.97 ( 2.39 - 3.55 ) | 4.87 ( 4.39 - 5.44 ) | 1.37 ( 1.12 - 1.67 ) | -94.84 | -3.68 ( -4.03 - -3.32 ) |
| Saint Lucia | Both | 2.63 ( 2.41 - 2.87 ) | 2.91 ( 2.65 - 3.16 ) | 4.87 ( 4.39 - 5.44 ) | 2.9 ( 2.59 - 3.23 ) | 85.01 | -0.17 ( -0.34 - 0 ) |
| Saint Vincent | Both | 1.96 ( 1.78 - 2.14 ) | 2.66 ( 2.41 - 2.89 ) | 0.9 ( 0.72 - 1.04 ) | 3.55 ( 3.2 - 3.96 ) | -54.1 | 1.03 ( 0.87 - 1.2 ) |
| Samoa | Both | 0.63 ( 0.53 - 0.75 ) | 0.77 ( 0.65 - 0.91 ) | 1.01 ( 0.82 - 1.24 ) | 0.68 ( 0.54 - 0.79 ) | 60.96 | -0.51 ( -0.59 - -0.42 ) |
| Sao Tome and Principe | Both | 0.54 ( 0.47 - 0.63 ) | 0.8 ( 0.69 - 0.93 ) | 249.95 ( 201.71 - 308.86 ) | 1.03 ( 0.83 - 1.25 ) | 46178.74 | 0.97 ( 0.89 - 1.04 ) |
| Saudi Arabia | Both | 83.29 ( 57.91 - 108.23 ) | 1.29 ( 0.9 - 1.67 ) | 105 ( 83.03 - 133.59 ) | 1.37 ( 1.15 - 1.67 ) | 26.05 | 0.46 ( -0.03 - 0.95 ) |
| Senegal | Both | 45.23 ( 37.11 - 54.6 ) | 1.38 ( 1.13 - 1.65 ) | 826.91 ( 728.04 - 942.85 ) | 1.48 ( 1.17 - 1.84 ) | 1728.05 | 0.61 ( 0.39 - 0.84 ) |
| Serbia | Both | 709.23 ( 615.08 - 846.2 ) | 5.62 ( 4.9 - 6.67 ) | 8.71 ( 7.6 - 9.89 ) | 5.5 ( 4.82 - 6.28 ) | -98.77 | 0.14 ( -0.03 - 0.32 ) |
| Seychelles | Both | 4.26 ( 3.68 - 4.8 ) | 7.42 ( 6.4 - 8.37 ) | 50.08 ( 40.69 - 61.51 ) | 7.85 ( 6.88 - 8.92 ) | 1075.52 | -0.15 ( -0.27 - -0.03 ) |
| Sierra Leone | Both | 26.41 ( 19.38 - 32.83 ) | 1.34 ( 0.99 - 1.68 ) | 111.83 ( 97.29 - 127.69 ) | 1.49 ( 1.22 - 1.8 ) | 323.53 | 0.69 ( 0.53 - 0.85 ) |
| Singapore | Both | 64.87 ( 58.52 - 71.68 ) | 2.76 ( 2.49 - 3.04 ) | 301.87 ( 262.73 - 350.42 ) | 1.61 ( 1.41 - 1.84 ) | 365.33 | -2.04 ( -2.22 - -1.85 ) |
| Slovakia | Both | 340.76 ( 315.52 - 363.18 ) | 5.73 ( 5.3 - 6.12 ) | 107.31 ( 93.59 - 121.87 ) | 3.37 ( 2.94 - 3.91 ) | -68.51 | -1.97 ( -2.05 - -1.89 ) |
| Slovenia | Both | 107.62 ( 99.91 - 116.67 ) | 4.23 ( 3.94 - 4.59 ) | 5.61 ( 4.65 - 6.69 ) | 2.81 ( 2.45 - 3.2 ) | -94.79 | -1.72 ( -1.85 - -1.6 ) |
| Solomon Islands | Both | 2.75 ( 2.23 - 3.31 ) | 1.91 ( 1.57 - 2.28 ) | 118.34 ( 87.47 - 155.92 ) | 1.75 ( 1.46 - 2.13 ) | 4209.21 | -0.19 ( -0.26 - -0.13 ) |
| Somalia | Both | 65.36 ( 35.57 - 97.76 ) | 2.24 ( 1.36 - 3.19 ) | 778.19 ( 731.07 - 839.17 ) | 1.72 ( 1.29 - 2.22 ) | 1090.62 | -1.48 ( -1.71 - -1.26 ) |
| South Africa | Both | 492.04 ( 433.3 - 626.28 ) | 2.2 ( 1.93 - 2.82 ) | 1776.5 ( 1538.43 - 2030.41 ) | 1.71 ( 1.61 - 1.84 ) | 261.05 | -1.36 ( -1.94 - -0.78 ) |
| South Korea | Both | 1210.5 ( 1127.53 - 1306.52 ) | 3.78 ( 3.53 - 4.06 ) | 68.79 ( 49.68 - 95.04 ) | 2.06 ( 1.79 - 2.35 ) | -94.32 | -3.83 ( -4.49 - -3.16 ) |
| South Sudan | Both | 56.61 ( 32.69 - 83.72 ) | 2.2 ( 1.32 - 3.15 ) | 3831.18 ( 3408.47 - 4284.51 ) | 1.68 ( 1.24 - 2.29 ) | 6667.76 | -1.41 ( -1.6 - -1.22 ) |
| Spain | Both | 4134.32 ( 3903.02 - 4377.66 ) | 7.78 ( 7.32 - 8.26 ) | 379.87 ( 308.05 - 457.61 ) | 4.66 ( 4.12 - 5.23 ) | -90.81 | -2.31 ( -2.46 - -2.15 ) |
| Sri Lanka | Both | 115.25 ( 103.85 - 128.9 ) | 1.03 ( 0.93 - 1.14 ) | 396.28 ( 299.82 - 515.37 ) | 1.48 ( 1.21 - 1.77 ) | 243.86 | 2.31 ( 2.04 - 2.58 ) |
| Sudan | Both | 234 ( 179.19 - 295.56 ) | 2.39 ( 1.86 - 3.03 ) | 7.18 ( 6.21 - 8.31 ) | 2.15 ( 1.63 - 2.82 ) | -96.93 | -0.35 ( -0.38 - -0.32 ) |
| Suriname | Both | 2.67 ( 2.41 - 2.95 ) | 1.01 ( 0.92 - 1.12 ) | 14.4 ( 10.61 - 17.94 ) | 1.21 ( 1.04 - 1.39 ) | 439.18 | 0.65 ( 0.46 - 0.85 ) |
| Swaziland | Both | 9.38 ( 7.49 - 11.35 ) | 2.95 ( 2.36 - 3.56 ) | 241.66 ( 215.52 - 268.05 ) | 2.4 ( 1.78 - 2.96 ) | 2477.34 | -0.43 ( -0.89 - 0.02 ) |
| Sweden | Both | 238.64 ( 220.89 - 258.66 ) | 1.68 ( 1.56 - 1.83 ) | 276.2 ( 240.18 - 315.03 ) | 1.27 ( 1.13 - 1.4 ) | 15.74 | -1.28 ( -1.37 - -1.18 ) |
| Switzerland | Both | 338.93 ( 308.15 - 370.85 ) | 3.46 ( 3.12 - 3.8 ) | 139.87 ( 113.14 - 173.58 ) | 1.78 ( 1.54 - 2.04 ) | -58.73 | -2.32 ( -2.46 - -2.17 ) |
| Syria | Both | 61.66 ( 53.75 - 70.99 ) | 1.11 ( 0.97 - 1.27 ) | 985.71 ( 865.12 - 1126.51 ) | 1.06 ( 0.86 - 1.31 ) | 1498.5 | -0.54 ( -0.91 - -0.17 ) |
| Tajikistan | Both | 37.6 ( 34.42 - 41.2 ) | 1.24 ( 1.14 - 1.36 ) | 350.9 ( 279.49 - 485.42 ) | 0.8 ( 0.7 - 0.91 ) | 833.34 | -1.81 ( -2.06 - -1.55 ) |
| Tanzania | Both | 231.74 ( 165.52 - 311.71 ) | 1.95 ( 1.43 - 2.59 ) | 2321.07 ( 1966.76 - 2733.39 ) | 1.38 ( 1.11 - 1.9 ) | 901.59 | -1.64 ( -1.85 - -1.43 ) |
| Thailand | Both | 1072.11 ( 964.12 - 1182.87 ) | 2.85 ( 2.57 - 3.15 ) | 6.98 ( 5.56 - 8.93 ) | 2.32 ( 1.96 - 2.72 ) | -99.35 | -1.24 ( -1.51 - -0.98 ) |
| Timor-Leste | Both | 5.49 ( 4.42 - 7.48 ) | 1.82 ( 1.48 - 2.49 ) | 14.3 ( 10.73 - 22.19 ) | 1.78 ( 1.35 - 2.74 ) | 160.3 | -0.01 ( -0.22 - 0.2 ) |
| Tobago | Both | 16.21 ( 14.97 - 17.49 ) | 1.87 ( 1.72 - 2.02 ) | 26.78 ( 20.96 - 33.69 ) | 1.48 ( 1.16 - 1.86 ) | 65.17 | -1.18 ( -1.45 - -0.9 ) |
| Togo | Both | 16.3 ( 13.48 - 18.98 ) | 1.28 ( 1.06 - 1.48 ) | 46.75 ( 35.55 - 58.85 ) | 1.32 ( 1.02 - 1.63 ) | 186.83 | 0.37 ( 0.25 - 0.48 ) |
| Tonga | Both | 0.68 ( 0.59 - 0.88 ) | 1.27 ( 1.09 - 1.63 ) | 1.09 ( 0.9 - 1.37 ) | 1.38 ( 1.14 - 1.73 ) | 59.93 | 0.42 ( 0.37 - 0.48 ) |
| Trinidad | Both | 16.21 ( 14.97 - 17.49 ) | 1.87 ( 1.72 - 2.02 ) | 26.78 ( 20.96 - 33.69 ) | 1.48 ( 1.16 - 1.86 ) | 65.17 | -1.18 ( -1.45 - -0.9 ) |
| Tunisia | Both | 175.67 ( 151.42 - 212.65 ) | 3.35 ( 2.89 - 4.03 ) | 421.19 ( 317.72 - 535.02 ) | 3.38 ( 2.56 - 4.27 ) | 139.76 | -0.29 ( -0.44 - -0.13 ) |
| Turkey | Both | 1433.03 ( 1245.97 - 1707.41 ) | 3.82 ( 3.34 - 4.55 ) | 2471.73 ( 2131.87 - 2827.18 ) | 2.81 ( 2.43 - 3.21 ) | 72.48 | -1.3 ( -1.46 - -1.14 ) |
| Turkmenistan | Both | 52.44 ( 48.75 - 56.26 ) | 2.47 ( 2.3 - 2.64 ) | 49.7 ( 43.84 - 55.09 ) | 1.21 ( 1.08 - 1.33 ) | -5.23 | -3.28 ( -3.83 - -2.72 ) |
| Uganda | Both | 109.14 ( 87.67 - 133.6 ) | 1.57 ( 1.27 - 1.92 ) | 188.82 ( 152.47 - 246.03 ) | 1.3 ( 1.05 - 1.7 ) | 73 | -1.26 ( -1.64 - -0.89 ) |
| UK | Both | 2319.35 ( 2250.65 - 2388.76 ) | 2.64 ( 2.56 - 2.72 ) | 2866.91 ( 2770.62 - 2968.28 ) | 2.48 ( 2.4 - 2.57 ) | 23.61 | -0.59 ( -0.72 - -0.47 ) |
| Ukraine | Both | 3623.09 ( 3397.91 - 3857.94 ) | 5.06 ( 4.73 - 5.39 ) | 3193.82 ( 2899.44 - 3502.13 ) | 4.41 ( 4 - 4.85 ) | -11.85 | -1.39 ( -1.83 - -0.94 ) |
| United Arab Emirates | Both | 18.84 ( 13.85 - 27.26 ) | 3.26 ( 2.51 - 4.19 ) | 228.39 ( 153.42 - 312.25 ) | 4.37 ( 2.76 - 5.96 ) | 1112.5 | 1.17 ( 1.06 - 1.29 ) |
| Uruguay | Both | 227.53 ( 212.83 - 244.16 ) | 5.82 ( 5.44 - 6.23 ) | 193.57 ( 165.86 - 224.82 ) | 3.88 ( 3.31 - 4.51 ) | -14.92 | -1.61 ( -1.77 - -1.45 ) |
| USA | Both | 11497.72 ( 11265.62 - 11769.58 ) | 3.71 ( 3.63 - 3.8 ) | 17048.53 ( 16431.79 - 17639.35 ) | 3.19 ( 3.08 - 3.31 ) | 48.28 | -1.15 ( -1.38 - -0.92 ) |
| Uzbekistan | Both | 240.51 ( 228.09 - 254.06 ) | 1.93 ( 1.83 - 2.03 ) | 522.63 ( 458.13 - 599.19 ) | 2.13 ( 1.88 - 2.43 ) | 117.3 | 0.9 ( 0.34 - 1.45 ) |
| Vanuatu | Both | 1.4 ( 1.04 - 2.16 ) | 2.1 ( 1.6 - 3.25 ) | 3.59 ( 2.53 - 6.78 ) | 2.2 ( 1.57 - 4.14 ) | 157.15 | 0.26 ( 0.22 - 0.3 ) |
| Venezuela | Both | 282.08 ( 265.33 - 298.86 ) | 2.86 ( 2.7 - 3.03 ) | 802.96 ( 671.3 - 960.84 ) | 2.82 ( 2.35 - 3.37 ) | 184.65 | -0.29 ( -0.42 - -0.17 ) |
| Vietnam | Both | 740.21 ( 625.25 - 860.83 ) | 1.75 ( 1.48 - 2.04 ) | 2295.14 ( 1863.23 - 2884.47 ) | 2.36 ( 1.94 - 2.94 ) | 210.06 | 1.15 ( 1.1 - 1.2 ) |
| Virgin Islands | Both | 2.54 ( 2.26 - 2.86 ) | 2.84 ( 2.53 - 3.17 ) | 7.4 ( 5.8 - 8.87 ) | 3.87 ( 3.08 - 4.64 ) | 191.44 | 1.54 ( 1.38 - 1.7 ) |
| Yemen | Both | 141.39 ( 86.31 - 195.31 ) | 2.58 ( 1.61 - 3.52 ) | 334.86 ( 249.94 - 433.85 ) | 2.53 ( 1.9 - 3.24 ) | 136.83 | -0.1 ( -0.16 - -0.04 ) |
| Zambia | Both | 85.51 ( 66.88 - 111.06 ) | 2.71 ( 2.18 - 3.52 ) | 117.91 ( 94.42 - 174.91 ) | 1.71 ( 1.38 - 2.52 ) | 37.89 | -2.43 ( -2.74 - -2.13 ) |
| Zimbabwe | Both | 105.44 ( 89.51 - 123.33 ) | 2.36 ( 2.01 - 2.75 ) | 171.97 ( 137.11 - 210.54 ) | 2.31 ( 1.86 - 2.8 ) | 63.1 | 0.32 ( -0.31 - 0.95 ) |
| Afghanistan | Female | 87.81 ( 52.07 - 116.4 ) | 2.52 ( 1.55 - 3.29 ) | 158.73 ( 116.26 - 216.07 ) | 2.32 ( 1.78 - 3.03 ) | 80.76 | -0.34 ( -0.53 - -0.15 ) |
| Albania | Female | 11.35 ( 10.06 - 12.81 ) | 0.93 ( 0.83 - 1.05 ) | 13.98 ( 10.27 - 18.61 ) | 0.69 ( 0.5 - 0.91 ) | 23.16 | -1.68 ( -2.21 - -1.15 ) |
| Algeria | Female | 38.15 ( 32.38 - 44.81 ) | 0.55 ( 0.47 - 0.64 ) | 81.25 ( 68.77 - 95.39 ) | 0.47 ( 0.39 - 0.54 ) | 113.01 | -0.32 ( -0.46 - -0.18 ) |
| American Samoa | Female | 0.03 ( 0.02 - 0.03 ) | 0.23 ( 0.19 - 0.27 ) | 0.12 ( 0.1 - 0.15 ) | 0.52 ( 0.44 - 0.61 ) | 358.78 | 4.32 ( 3.66 - 4.99 ) |
| Andorra | Female | 0.14 ( 0.1 - 0.19 ) | 0.48 ( 0.35 - 0.67 ) | 0.33 ( 0.24 - 0.43 ) | 0.51 ( 0.38 - 0.67 ) | 134.29 | -0.15 ( -0.4 - 0.1 ) |
| Angola | Female | 12.51 ( 9.08 - 16.46 ) | 0.56 ( 0.42 - 0.73 ) | 21.73 ( 16.48 - 28.58 ) | 0.35 ( 0.27 - 0.46 ) | 73.67 | -2.05 ( -2.24 - -1.86 ) |
| Antigua | Female | 0.04 ( 0.04 - 0.05 ) | 0.15 ( 0.13 - 0.17 ) | 0.08 ( 0.07 - 0.1 ) | 0.16 ( 0.14 - 0.18 ) | 95.58 | 0.02 ( -0.14 - 0.17 ) |
| Argentina | Female | 94.46 ( 86.37 - 103.03 ) | 0.52 ( 0.48 - 0.57 ) | 185.18 ( 155.1 - 218.88 ) | 0.64 ( 0.53 - 0.76 ) | 96.05 | 0.53 ( 0.25 - 0.81 ) |
| Armenia | Female | 12.49 ( 11.08 - 14.12 ) | 0.77 ( 0.69 - 0.87 ) | 17.3 ( 15.26 - 19.77 ) | 0.74 ( 0.65 - 0.85 ) | 38.46 | 0.4 ( -0.48 - 1.28 ) |
| Australia | Female | 85.88 ( 73.39 - 98.96 ) | 0.81 ( 0.69 - 0.93 ) | 125.51 ( 100.76 - 156.5 ) | 0.63 ( 0.5 - 0.78 ) | 46.14 | -1.23 ( -1.38 - -1.08 ) |
| Austria | Female | 28.7 ( 25.48 - 32.44 ) | 0.46 ( 0.4 - 0.52 ) | 47.32 ( 38.97 - 57.05 ) | 0.59 ( 0.48 - 0.72 ) | 64.87 | 0.78 ( 0.51 - 1.04 ) |
| Azerbaijan | Female | 28.75 ( 25.45 - 32.35 ) | 0.91 ( 0.81 - 1.02 ) | 58.41 ( 48.29 - 71.19 ) | 1.05 ( 0.87 - 1.26 ) | 103.18 | -0.25 ( -0.72 - 0.22 ) |
| Bahamas | Female | 0.61 ( 0.54 - 0.68 ) | 0.68 ( 0.61 - 0.77 ) | 1.34 ( 1.11 - 1.61 ) | 0.64 ( 0.54 - 0.77 ) | 121.06 | -0.16 ( -0.36 - 0.04 ) |
| Bahrain | Female | 0.36 ( 0.3 - 0.43 ) | 0.43 ( 0.36 - 0.5 ) | 0.68 ( 0.56 - 0.83 ) | 0.18 ( 0.15 - 0.21 ) | 90.57 | -4.79 ( -5.92 - -3.65 ) |
| Bangladesh | Female | 567.94 ( 462.06 - 706.44 ) | 2.31 ( 1.87 - 2.9 ) | 872.75 ( 671.26 - 1098.08 ) | 1.39 ( 1.07 - 1.75 ) | 53.67 | -1.38 ( -1.6 - -1.16 ) |
| Barbados | Female | 0.88 ( 0.78 - 0.99 ) | 0.53 ( 0.47 - 0.59 ) | 1.35 ( 1.14 - 1.61 ) | 0.51 ( 0.43 - 0.61 ) | 53.17 | -0.26 ( -0.46 - -0.05 ) |
| Barbuda | Female | 0.04 ( 0.04 - 0.05 ) | 0.15 ( 0.13 - 0.17 ) | 0.08 ( 0.07 - 0.1 ) | 0.16 ( 0.14 - 0.18 ) | 95.58 | 0.02 ( -0.14 - 0.17 ) |
| Belarus | Female | 23.34 ( 20.71 - 26.21 ) | 0.3 ( 0.27 - 0.34 ) | 21.44 ( 17.83 - 25.51 ) | 0.25 ( 0.2 - 0.3 ) | -8.12 | -1.08 ( -1.34 - -0.83 ) |
| Belgium | Female | 73.99 ( 65.56 - 83.12 ) | 0.95 ( 0.83 - 1.07 ) | 83.99 ( 69.72 - 100.68 ) | 0.85 ( 0.7 - 1.03 ) | 13.53 | -0.4 ( -0.6 - -0.19 ) |
| Belize | Female | 0.23 ( 0.2 - 0.26 ) | 0.48 ( 0.42 - 0.55 ) | 0.57 ( 0.5 - 0.65 ) | 0.43 ( 0.38 - 0.49 ) | 147.58 | -0.39 ( -0.75 - -0.03 ) |
| Benin | Female | 2.94 ( 2.37 - 3.58 ) | 0.27 ( 0.22 - 0.33 ) | 6.55 ( 4.79 - 8.77 ) | 0.26 ( 0.19 - 0.35 ) | 122.85 | 0.03 ( -0.06 - 0.13 ) |
| Bermuda | Female | 0.26 ( 0.23 - 0.29 ) | 0.71 ( 0.63 - 0.8 ) | 0.31 ( 0.26 - 0.37 ) | 0.45 ( 0.36 - 0.53 ) | 21.3 | -2.11 ( -2.44 - -1.77 ) |
| Bhutan | Female | 3.22 ( 2.53 - 4.21 ) | 2.26 ( 1.76 - 2.96 ) | 3.45 ( 2.54 - 4.52 ) | 1.09 ( 0.82 - 1.43 ) | 7.06 | -3.01 ( -3.22 - -2.8 ) |
| Bolivia | Female | 20.4 ( 16.79 - 24.92 ) | 1.12 ( 0.92 - 1.35 ) | 29.59 ( 22.27 - 38.23 ) | 0.64 ( 0.49 - 0.83 ) | 44.99 | -2.4 ( -2.63 - -2.17 ) |
| Bosnia and Herzegovina | Female | 22.98 ( 20.37 - 25.96 ) | 0.96 ( 0.86 - 1.08 ) | 24.79 ( 20.87 - 29.27 ) | 0.78 ( 0.66 - 0.92 ) | 7.88 | -1.56 ( -2.13 - -0.99 ) |
| Botswana | Female | 1.72 ( 1.23 - 2.24 ) | 0.5 ( 0.36 - 0.65 ) | 3.94 ( 3.16 - 4.85 ) | 0.49 ( 0.39 - 0.59 ) | 129.67 | 1.27 ( 0.34 - 2.22 ) |
| Brazil | Female | 392.3 ( 375.91 - 408.74 ) | 0.79 ( 0.76 - 0.83 ) | 950.92 ( 903.64 - 1002.48 ) | 0.75 ( 0.72 - 0.79 ) | 142.4 | -0.27 ( -0.36 - -0.18 ) |
| Brunei | Female | 1.24 ( 1.04 - 1.47 ) | 2.5 ( 2.1 - 2.96 ) | 1.21 ( 0.99 - 1.46 ) | 0.76 ( 0.62 - 0.91 ) | -2.05 | -5.69 ( -6.23 - -5.15 ) |
| Bulgaria | Female | 26.25 ( 23.56 - 29.16 ) | 0.4 ( 0.36 - 0.45 ) | 36.94 ( 31.47 - 43.14 ) | 0.56 ( 0.47 - 0.67 ) | 40.7 | 1.22 ( 1.03 - 1.42 ) |
| Burkina Faso | Female | 9.94 ( 8.05 - 12.09 ) | 0.41 ( 0.33 - 0.49 ) | 16.52 ( 13.02 - 20.53 ) | 0.35 ( 0.27 - 0.43 ) | 66.22 | -0.56 ( -0.72 - -0.4 ) |
| Burundi | Female | 17.5 ( 13.4 - 23 ) | 1.26 ( 0.97 - 1.64 ) | 14.61 ( 10.42 - 19.13 ) | 0.64 ( 0.46 - 0.83 ) | -16.51 | -3.1 ( -3.38 - -2.82 ) |
| Cambodia | Female | 37.8 ( 30.65 - 45.5 ) | 1.34 ( 1.09 - 1.6 ) | 45.89 ( 35.97 - 58.19 ) | 0.68 ( 0.54 - 0.86 ) | 21.41 | -2.73 ( -2.85 - -2.61 ) |
| Cameroon | Female | 8.53 ( 7.02 - 10.57 ) | 0.34 ( 0.28 - 0.42 ) | 17.46 ( 12.7 - 23.84 ) | 0.29 ( 0.22 - 0.4 ) | 104.59 | -0.42 ( -0.57 - -0.27 ) |
| Canada | Female | 207.73 ( 180.39 - 238.91 ) | 1.18 ( 1.02 - 1.37 ) | 272.53 ( 224.89 - 326.94 ) | 0.81 ( 0.66 - 0.98 ) | 31.2 | -1.68 ( -1.82 - -1.54 ) |
| Cape Verde | Female | 0.15 ( 0.13 - 0.17 ) | 0.11 ( 0.09 - 0.13 ) | 0.73 ( 0.63 - 0.83 ) | 0.29 ( 0.25 - 0.33 ) | 397.4 | 3.6 ( 2.95 - 4.25 ) |
| Central African Republic | Female | 5.34 ( 3.99 - 6.72 ) | 0.78 ( 0.59 - 0.97 ) | 6.58 ( 4.4 - 8.96 ) | 0.55 ( 0.38 - 0.74 ) | 23.02 | -1.3 ( -1.42 - -1.17 ) |
| Chad | Female | 4.22 ( 3.45 - 5.12 ) | 0.28 ( 0.22 - 0.33 ) | 7.99 ( 6.05 - 10.47 ) | 0.32 ( 0.24 - 0.41 ) | 89.37 | 0.75 ( 0.66 - 0.85 ) |
| Chile | Female | 26.45 ( 23.73 - 29.31 ) | 0.47 ( 0.42 - 0.52 ) | 44.85 ( 36.46 - 54.32 ) | 0.35 ( 0.29 - 0.43 ) | 69.57 | -0.97 ( -1.32 - -0.62 ) |
| China | Female | 3017.1 ( 2856.18 - 3254.36 ) | 0.67 ( 0.63 - 0.73 ) | 6425.8 ( 6032.46 - 6828.23 ) | 0.65 ( 0.61 - 0.69 ) | 112.98 | 0.14 ( -0.34 - 0.63 ) |
| Colombia | Female | 119.57 ( 111.65 - 128.52 ) | 1.3 ( 1.22 - 1.4 ) | 142.38 ( 118.54 - 169.04 ) | 0.48 ( 0.4 - 0.57 ) | 19.07 | -4.63 ( -4.95 - -4.3 ) |
| Comoros | Female | 1.06 ( 0.72 - 1.39 ) | 0.91 ( 0.62 - 1.19 ) | 1.37 ( 0.94 - 1.84 ) | 0.53 ( 0.36 - 0.71 ) | 28.92 | -2.27 ( -2.39 - -2.14 ) |
| Costa Rica | Female | 5.52 ( 5 - 6.09 ) | 0.6 ( 0.54 - 0.66 ) | 8.2 ( 7.07 - 9.56 ) | 0.31 ( 0.26 - 0.36 ) | 48.68 | -2.8 ( -3.25 - -2.34 ) |
| Croatia | Female | 23.88 ( 21.07 - 26.93 ) | 0.65 ( 0.58 - 0.74 ) | 23.32 ( 19.84 - 27.44 ) | 0.55 ( 0.46 - 0.66 ) | -2.33 | -0.53 ( -1.01 - -0.04 ) |
| Cuba | Female | 104.47 ( 94.66 - 115.87 ) | 1.98 ( 1.79 - 2.19 ) | 206.23 ( 169.44 - 251.11 ) | 2.13 ( 1.75 - 2.6 ) | 97.4 | 0.17 ( 0.05 - 0.29 ) |
| Cyprus | Female | 1.58 ( 1.36 - 1.83 ) | 0.35 ( 0.3 - 0.41 ) | 2.49 ( 2.03 - 3.08 ) | 0.27 ( 0.22 - 0.33 ) | 57.85 | -1.14 ( -1.36 - -0.92 ) |
| Czech Republic | Female | 38.46 ( 34.2 - 43.02 ) | 0.51 ( 0.45 - 0.58 ) | 54.66 ( 45.8 - 64.86 ) | 0.58 ( 0.49 - 0.7 ) | 42.13 | 0.74 ( 0.6 - 0.89 ) |
| Democratic Republic of the Congo | Female | 45.19 ( 33.85 - 57.71 ) | 0.48 ( 0.37 - 0.61 ) | 80.26 ( 57.9 - 107.6 ) | 0.41 ( 0.3 - 0.55 ) | 77.63 | -0.65 ( -0.72 - -0.58 ) |
| Denmark | Female | 50.63 ( 44.93 - 57.49 ) | 1.28 ( 1.13 - 1.47 ) | 43.9 ( 36.39 - 52.48 ) | 0.83 ( 0.68 - 1 ) | -13.29 | -1.86 ( -2.02 - -1.7 ) |
| Djibouti | Female | 0.7 ( 0.48 - 0.96 ) | 0.8 ( 0.56 - 1.1 ) | 1.42 ( 0.93 - 2.09 ) | 0.48 ( 0.32 - 0.7 ) | 102.06 | -2.29 ( -2.49 - -2.1 ) |
| Dominica | Female | 0.33 ( 0.29 - 0.37 ) | 0.78 ( 0.7 - 0.88 ) | 0.36 ( 0.31 - 0.42 ) | 0.78 ( 0.66 - 0.91 ) | 11.36 | -0.11 ( -0.26 - 0.03 ) |
| Dominican Republic | Female | 16.65 ( 14.47 - 18.92 ) | 0.83 ( 0.72 - 0.94 ) | 34.75 ( 28.07 - 42.88 ) | 0.72 ( 0.58 - 0.88 ) | 108.7 | -0.51 ( -0.9 - -0.11 ) |
| Ecuador | Female | 16.38 ( 15.12 - 17.7 ) | 0.56 ( 0.52 - 0.61 ) | 36.01 ( 30.29 - 42.28 ) | 0.47 ( 0.39 - 0.55 ) | 119.84 | -0.9 ( -1.32 - -0.48 ) |
| Egypt | Female | 59.86 ( 51.22 - 70.12 ) | 0.33 ( 0.28 - 0.37 ) | 114.26 ( 89.64 - 142.86 ) | 0.31 ( 0.25 - 0.38 ) | 90.89 | -0.06 ( -0.2 - 0.09 ) |
| El Salvador | Female | 3.24 ( 2.89 - 3.62 ) | 0.2 ( 0.18 - 0.22 ) | 9.12 ( 7.11 - 11.55 ) | 0.28 ( 0.21 - 0.35 ) | 181.86 | 1.5 ( 1.22 - 1.78 ) |
| Equatorial Guinea | Female | 0.82 ( 0.6 - 1.08 ) | 0.7 ( 0.52 - 0.91 ) | 0.92 ( 0.55 - 1.44 ) | 0.32 ( 0.2 - 0.5 ) | 11.97 | -3.27 ( -3.63 - -2.9 ) |
| Eritrea | Female | 6.76 ( 4.97 - 8.78 ) | 1.07 ( 0.81 - 1.36 ) | 10.19 ( 7.2 - 13.61 ) | 0.68 ( 0.49 - 0.88 ) | 50.65 | -1.93 ( -2.04 - -1.81 ) |
| Estonia | Female | 5.54 ( 4.88 - 6.32 ) | 0.44 ( 0.39 - 0.5 ) | 6.33 ( 4.98 - 8.02 ) | 0.5 ( 0.39 - 0.64 ) | 14.28 | 0.3 ( 0.04 - 0.56 ) |
| Ethiopia | Female | 80.16 ( 62.76 - 98.28 ) | 0.68 ( 0.54 - 0.84 ) | 61.46 ( 52.8 - 71.21 ) | 0.28 ( 0.24 - 0.33 ) | -23.33 | -3.78 ( -3.99 - -3.57 ) |
| Fiji | Female | 0.94 ( 0.76 - 1.16 ) | 0.47 ( 0.38 - 0.57 ) | 2.04 ( 1.61 - 2.55 ) | 0.51 ( 0.4 - 0.62 ) | 117.32 | 0.61 ( 0.42 - 0.8 ) |
| Finland | Female | 12.9 ( 11.43 - 14.52 ) | 0.33 ( 0.29 - 0.37 ) | 16.94 ( 14.09 - 20.48 ) | 0.32 ( 0.26 - 0.39 ) | 31.31 | 0.09 ( -0.05 - 0.24 ) |
| France | Female | 301.25 ( 274.68 - 330.59 ) | 0.72 ( 0.64 - 0.79 ) | 490.39 ( 409.88 - 589.91 ) | 0.85 ( 0.7 - 1.04 ) | 62.78 | 0.73 ( 0.55 - 0.92 ) |
| Gabon | Female | 1.64 ( 1.29 - 2.07 ) | 0.5 ( 0.4 - 0.64 ) | 1.71 ( 1.25 - 2.28 ) | 0.3 ( 0.22 - 0.4 ) | 4.3 | -1.94 ( -2.2 - -1.67 ) |
| Gambia | Female | 0.36 ( 0.28 - 0.47 ) | 0.2 ( 0.16 - 0.26 ) | 21.73 ( 18.92 - 24.86 ) | 0.2 ( 0.16 - 0.25 ) | 5863.46 | 0.2 ( 0.12 - 0.28 ) |
| Georgia | Female | 27.51 ( 24.6 - 30.94 ) | 0.76 ( 0.68 - 0.86 ) | 564.45 ( 453.13 - 680.81 ) | 0.68 ( 0.59 - 0.79 ) | 1951.53 | -0.82 ( -1.43 - -0.21 ) |
| Germany | Female | 311.22 ( 285.56 - 341.97 ) | 0.46 ( 0.42 - 0.51 ) | 22.92 ( 18.05 - 29.38 ) | 0.69 ( 0.54 - 0.84 ) | -92.63 | 1.18 ( 0.99 - 1.38 ) |
| Ghana | Female | 10.08 ( 7.72 - 12.97 ) | 0.28 ( 0.22 - 0.36 ) | 62.94 ( 53.41 - 73.15 ) | 0.26 ( 0.21 - 0.33 ) | 524.39 | -0.09 ( -0.17 - -0.01 ) |
| Greece | Female | 41 ( 36.44 - 45.94 ) | 0.5 ( 0.45 - 0.56 ) | 0.1 ( 0.08 - 0.12 ) | 0.59 ( 0.49 - 0.69 ) | -99.76 | 0.89 ( 0.65 - 1.14 ) |
| Greenland | Female | 0.05 ( 0.04 - 0.06 ) | 0.29 ( 0.24 - 0.34 ) | 0.33 ( 0.29 - 0.38 ) | 0.3 ( 0.25 - 0.36 ) | 529.25 | 0.66 ( 0.14 - 1.18 ) |
| Grenada | Female | 0.3 ( 0.27 - 0.34 ) | 0.73 ( 0.65 - 0.82 ) | 0.69 ( 0.58 - 0.83 ) | 0.44 ( 0.38 - 0.51 ) | 131.55 | -1.41 ( -1.72 - -1.1 ) |
| Grenadines | Female | 0.22 ( 0.19 - 0.24 ) | 0.53 ( 0.47 - 0.59 ) | 20.93 ( 17.46 - 24.73 ) | 0.43 ( 0.38 - 0.5 ) | 9512.01 | -0.87 ( -1.15 - -0.58 ) |
| Guam | Female | 0.1 ( 0.08 - 0.11 ) | 0.23 ( 0.19 - 0.27 ) | 8.88 ( 6.9 - 11.36 ) | 0.73 ( 0.61 - 0.87 ) | 9211.01 | 5.55 ( 4.79 - 6.33 ) |
| Guatemala | Female | 11.01 ( 10.02 - 12.09 ) | 0.57 ( 0.52 - 0.62 ) | 1.37 ( 1.04 - 1.77 ) | 0.35 ( 0.29 - 0.41 ) | -87.52 | -1.99 ( -2.65 - -1.32 ) |
| Guinea | Female | 5.27 ( 4.29 - 6.34 ) | 0.3 ( 0.25 - 0.36 ) | 1.12 ( 0.92 - 1.34 ) | 0.33 ( 0.26 - 0.42 ) | -78.83 | 0.62 ( 0.47 - 0.77 ) |
| Guinea-Bissau | Female | 0.93 ( 0.7 - 1.2 ) | 0.41 ( 0.31 - 0.53 ) | 30.32 ( 22 - 40.18 ) | 0.36 ( 0.27 - 0.45 ) | 3144.12 | -0.42 ( -0.55 - -0.3 ) |
| Guyana | Female | 0.7 ( 0.63 - 0.78 ) | 0.34 ( 0.3 - 0.38 ) | 5.54 ( 4.06 - 7.13 ) | 0.34 ( 0.28 - 0.4 ) | 689.84 | -0.18 ( -0.56 - 0.19 ) |
| Haiti | Female | 21.88 ( 17.45 - 26.86 ) | 1.27 ( 1.02 - 1.55 ) | 132.25 ( 111.15 - 158.85 ) | 0.83 ( 0.62 - 1.09 ) | 504.38 | -1.65 ( -1.75 - -1.56 ) |
| Honduras | Female | 1.98 ( 1.66 - 2.37 ) | 0.19 ( 0.16 - 0.22 ) | 0.96 ( 0.8 - 1.13 ) | 0.18 ( 0.13 - 0.23 ) | -51.71 | -0.4 ( -0.58 - -0.21 ) |
| Hungary | Female | 82.82 ( 73.73 - 92.55 ) | 1.07 ( 0.94 - 1.21 ) | 9038.35 ( 8317.16 - 9716.49 ) | 1.48 ( 1.23 - 1.8 ) | 10812.88 | 1.5 ( 1 - 2 ) |
| Iceland | Female | 0.91 ( 0.79 - 1.06 ) | 0.64 ( 0.55 - 0.74 ) | 567.48 ( 515.65 - 631.27 ) | 0.38 ( 0.32 - 0.46 ) | 62255.94 | -1.85 ( -2.04 - -1.65 ) |
| India | Female | 4987.32 ( 4472.75 - 5509.48 ) | 1.92 ( 1.72 - 2.13 ) | 486.48 ( 443.49 - 536.53 ) | 1.54 ( 1.41 - 1.65 ) | -90.25 | -1.26 ( -1.62 - -0.9 ) |
| Indonesia | Female | 447.85 ( 401.37 - 499.31 ) | 0.8 ( 0.71 - 0.89 ) | 88.81 ( 76.5 - 103.08 ) | 0.51 ( 0.46 - 0.56 ) | -80.17 | -1.7 ( -1.72 - -1.68 ) |
| Iran | Female | 213.27 ( 196.31 - 232.35 ) | 1.56 ( 1.45 - 1.69 ) | 23.82 ( 19.62 - 28.63 ) | 1.36 ( 1.25 - 1.49 ) | -88.83 | -0.82 ( -1.32 - -0.32 ) |
| Iraq | Female | 81.36 ( 64.98 - 102.27 ) | 1.87 ( 1.5 - 2.32 ) | 19.58 ( 16.71 - 22.78 ) | 0.72 ( 0.62 - 0.82 ) | -75.94 | -3.9 ( -4.5 - -3.29 ) |
| Ireland | Female | 18.89 ( 16.7 - 21.14 ) | 0.87 ( 0.77 - 0.98 ) | 389.92 ( 330.05 - 460.42 ) | 0.67 ( 0.54 - 0.8 ) | 1964.18 | -1.27 ( -1.43 - -1.1 ) |
| Israel | Female | 8.56 ( 7.67 - 9.58 ) | 0.33 ( 0.3 - 0.37 ) | 4.66 ( 3.57 - 6.03 ) | 0.34 ( 0.29 - 0.39 ) | -45.56 | -0.37 ( -0.72 - -0.02 ) |
| Italy | Female | 302.08 ( 273.94 - 335.14 ) | 0.63 ( 0.57 - 0.7 ) | 4.61 ( 3.62 - 5.81 ) | 0.6 ( 0.5 - 0.71 ) | -98.47 | 0.1 ( -0.08 - 0.28 ) |
| Ivory Coast | Female | 2.17 ( 1.78 - 2.66 ) | 0.11 ( 0.09 - 0.14 ) | 530.92 ( 477.16 - 581.66 ) | 0.1 ( 0.07 - 0.12 ) | 24311.02 | -0.57 ( -0.73 - -0.4 ) |
| Jamaica | Female | 3.56 ( 3.17 - 4.01 ) | 0.37 ( 0.33 - 0.42 ) | 6.17 ( 4.92 - 7.65 ) | 0.31 ( 0.24 - 0.38 ) | 73.45 | -0.77 ( -1.18 - -0.35 ) |
| Japan | Female | 424.67 ( 390.03 - 461.06 ) | 0.45 ( 0.41 - 0.49 ) | 44.17 ( 37.49 - 51.6 ) | 0.34 ( 0.3 - 0.37 ) | -89.6 | -1.07 ( -1.15 - -0.99 ) |
| Jordan | Female | 4.65 ( 3.82 - 5.66 ) | 0.6 ( 0.49 - 0.73 ) | 47.34 ( 40.79 - 54.2 ) | 0.21 ( 0.17 - 0.26 ) | 917.11 | -5.22 ( -6.21 - -4.21 ) |
| Kazakhstan | Female | 70.74 ( 64.46 - 78.27 ) | 0.87 ( 0.79 - 0.96 ) | 0.29 ( 0.23 - 0.37 ) | 0.43 ( 0.36 - 0.5 ) | -99.59 | -3.17 ( -3.7 - -2.65 ) |
| Kenya | Female | 23.68 ( 20.12 - 27.47 ) | 0.5 ( 0.43 - 0.58 ) | 2.76 ( 2.24 - 3.4 ) | 0.38 ( 0.33 - 0.43 ) | -88.33 | -1.18 ( -1.36 - -1 ) |
| Kiribati | Female | 0.18 ( 0.15 - 0.21 ) | 0.84 ( 0.71 - 0.98 ) | 6.3 ( 5.52 - 7.19 ) | 0.73 ( 0.57 - 0.91 ) | 3342.33 | -0.58 ( -0.72 - -0.44 ) |
| Kuwait | Female | 1.84 ( 1.55 - 2.15 ) | 0.63 ( 0.54 - 0.73 ) | 14.75 ( 11.42 - 18.83 ) | 0.22 ( 0.18 - 0.26 ) | 701.39 | -3.76 ( -4.68 - -2.82 ) |
| Kyrgyzstan | Female | 7.33 ( 6.49 - 8.25 ) | 0.39 ( 0.35 - 0.44 ) | 9.13 ( 7.31 - 11.39 ) | 0.24 ( 0.21 - 0.27 ) | 24.67 | -2.39 ( -2.89 - -1.88 ) |
| Laos | Female | 17.38 ( 13.24 - 22.64 ) | 1.47 ( 1.12 - 1.89 ) | 57.54 ( 45.59 - 71.53 ) | 0.65 ( 0.51 - 0.83 ) | 231.11 | -3.24 ( -3.34 - -3.15 ) |
| Latvia | Female | 10.72 ( 9.43 - 12.27 ) | 0.48 ( 0.42 - 0.54 ) | 4.86 ( 3.21 - 6.78 ) | 0.44 ( 0.34 - 0.56 ) | -54.66 | 0.2 ( -0.22 - 0.62 ) |
| Lebanon | Female | 20.18 ( 15.85 - 25.04 ) | 1.53 ( 1.21 - 1.88 ) | 2.74 ( 2.06 - 3.58 ) | 1.7 ( 1.36 - 2.11 ) | -86.41 | 0.28 ( -0.05 - 0.62 ) |
| Lesotho | Female | 3.74 ( 2.67 - 4.86 ) | 0.66 ( 0.47 - 0.85 ) | 27.6 ( 20.54 - 35.4 ) | 0.67 ( 0.44 - 0.93 ) | 638.6 | 0.96 ( 0.58 - 1.35 ) |
| Liberia | Female | 1.72 ( 1.35 - 2.14 ) | 0.32 ( 0.25 - 0.4 ) | 16.26 ( 13.4 - 19.82 ) | 0.28 ( 0.22 - 0.37 ) | 842.69 | -0.45 ( -0.61 - -0.3 ) |
| Libya | Female | 7.89 ( 6.23 - 9.84 ) | 0.81 ( 0.64 - 1.01 ) | 4.49 ( 3.58 - 5.57 ) | 1.08 ( 0.81 - 1.36 ) | -43.14 | 1.31 ( 1.13 - 1.49 ) |
| Lithuania | Female | 12.49 ( 10.83 - 14.3 ) | 0.46 ( 0.4 - 0.52 ) | 13.11 ( 10.71 - 15.96 ) | 0.57 ( 0.47 - 0.7 ) | 4.96 | 0.94 ( 0.49 - 1.39 ) |
| Luxembourg | Female | 2.48 ( 2.16 - 2.85 ) | 0.85 ( 0.73 - 0.98 ) | 32.23 ( 22.43 - 43.65 ) | 0.97 ( 0.77 - 1.21 ) | 1201.57 | 0.56 ( 0.4 - 0.71 ) |
| Macedonia | Female | 7.61 ( 6.57 - 8.74 ) | 0.72 ( 0.62 - 0.82 ) | 14.51 ( 11.17 - 18.35 ) | 0.82 ( 0.67 - 0.99 ) | 90.85 | 0.36 ( 0 - 0.71 ) |
| Madagascar | Female | 23.1 ( 17.99 - 28.13 ) | 0.79 ( 0.62 - 0.95 ) | 70.32 ( 58.38 - 85.33 ) | 0.51 ( 0.36 - 0.68 ) | 204.44 | -1.88 ( -2.03 - -1.74 ) |
| Malawi | Female | 13.28 ( 8.33 - 17.01 ) | 0.56 ( 0.37 - 0.71 ) | 0.18 ( 0.15 - 0.21 ) | 0.33 ( 0.25 - 0.42 ) | -98.67 | -2.87 ( -3.31 - -2.43 ) |
| Malaysia | Female | 30.07 ( 26.32 - 34.37 ) | 0.61 ( 0.54 - 0.7 ) | 15.96 ( 12.41 - 20.18 ) | 0.54 ( 0.45 - 0.65 ) | -46.94 | -0.8 ( -1.11 - -0.5 ) |
| Maldives | Female | 0.14 ( 0.11 - 0.17 ) | 0.33 ( 0.28 - 0.4 ) | 2.48 ( 2.1 - 2.95 ) | 0.13 ( 0.11 - 0.16 ) | 1732.61 | -4.15 ( -4.38 - -3.91 ) |
| Mali | Female | 12.42 ( 10.29 - 14.7 ) | 0.54 ( 0.45 - 0.64 ) | 0.17 ( 0.13 - 0.23 ) | 0.36 ( 0.28 - 0.45 ) | -98.6 | -1.8 ( -1.95 - -1.64 ) |
| Malta | Female | 1.21 ( 1.07 - 1.38 ) | 0.51 ( 0.45 - 0.58 ) | 2.6 ( 1.95 - 3.44 ) | 0.6 ( 0.51 - 0.72 ) | 114.22 | 0.16 ( -0.05 - 0.38 ) |
| Marshall Islands | Female | 0.07 ( 0.06 - 0.09 ) | 0.81 ( 0.67 - 1 ) | 2.65 ( 2.23 - 3.1 ) | 0.97 ( 0.76 - 1.24 ) | 3637.62 | 0.68 ( 0.42 - 0.94 ) |
| Mauritania | Female | 1.86 ( 1.48 - 2.29 ) | 0.34 ( 0.27 - 0.42 ) | 205.99 ( 196.65 - 215.73 ) | 0.26 ( 0.19 - 0.33 ) | 10965.64 | -0.94 ( -1.09 - -0.79 ) |
| Mauritius | Female | 3.27 ( 2.93 - 3.64 ) | 0.79 ( 0.71 - 0.87 ) | 0.33 ( 0.26 - 0.44 ) | 0.29 ( 0.25 - 0.34 ) | -89.77 | -5.5 ( -6.24 - -4.75 ) |
| Mexico | Female | 154.42 ( 149.72 - 159.24 ) | 0.67 ( 0.65 - 0.7 ) | 9.74 ( 8.34 - 11.26 ) | 0.34 ( 0.33 - 0.36 ) | -93.69 | -3.29 ( -3.59 - -2.98 ) |
| Micronesia | Female | 0.23 ( 0.18 - 0.28 ) | 0.88 ( 0.7 - 1.07 ) | 3.97 ( 3.25 - 4.79 ) | 0.84 ( 0.66 - 1.07 ) | 1649.45 | -0.21 ( -0.29 - -0.12 ) |
| Moldova | Female | 8.42 ( 7.46 - 9.59 ) | 0.31 ( 0.28 - 0.35 ) | 16.52 ( 13.46 - 20.26 ) | 0.31 ( 0.27 - 0.36 ) | 96.28 | 0.55 ( 0 - 1.1 ) |
| Mongolia | Female | 2.95 ( 2.49 - 3.49 ) | 0.5 ( 0.42 - 0.59 ) | 78.77 ( 60.54 - 100.53 ) | 0.36 ( 0.29 - 0.44 ) | 2567.87 | -1.52 ( -1.97 - -1.08 ) |
| Montenegro | Female | 9.73 ( 8.19 - 11.45 ) | 2.73 ( 2.3 - 3.2 ) | 41.46 ( 30.3 - 54.67 ) | 3.31 ( 2.68 - 4.06 ) | 326.29 | 0.79 ( 0.47 - 1.12 ) |
| Morocco | Female | 46.29 ( 39.44 - 54.69 ) | 0.58 ( 0.5 - 0.69 ) | 200.75 ( 152.38 - 256.09 ) | 0.46 ( 0.36 - 0.59 ) | 333.69 | -0.92 ( -1 - -0.84 ) |
| Mozambique | Female | 30.36 ( 21.57 - 39.48 ) | 0.81 ( 0.58 - 1.05 ) | 7.04 ( 5.18 - 9.58 ) | 0.61 ( 0.45 - 0.8 ) | -76.81 | -1.22 ( -1.38 - -1.05 ) |
| Myanmar | Female | 248.53 ( 181.98 - 332.18 ) | 1.9 ( 1.4 - 2.52 ) | 213.28 ( 144.75 - 278.59 ) | 0.79 ( 0.6 - 1.01 ) | -14.18 | -3.49 ( -3.72 - -3.26 ) |
| Namibia | Female | 6.59 ( 5.2 - 8.2 ) | 1.58 ( 1.25 - 1.96 ) | 135.21 ( 111.93 - 160.61 ) | 0.84 ( 0.62 - 1.13 ) | 1950.84 | -3.14 ( -3.92 - -2.36 ) |
| Nepal | Female | 155.21 ( 106.4 - 207.22 ) | 2.96 ( 1.94 - 3.93 ) | 18.95 ( 15.65 - 22.44 ) | 1.78 ( 1.21 - 2.33 ) | -87.79 | -1.96 ( -2.35 - -1.57 ) |
| Netherlands | Female | 56.36 ( 49.63 - 63.89 ) | 0.55 ( 0.48 - 0.62 ) | 6.6 ( 5.38 - 7.97 ) | 0.86 ( 0.71 - 1.03 ) | -88.29 | 1.53 ( 1.14 - 1.92 ) |
| New Zealand | Female | 21.44 ( 17.63 - 25.36 ) | 1.02 ( 0.84 - 1.2 ) | 9.78 ( 7.05 - 13.1 ) | 0.51 ( 0.42 - 0.6 ) | -54.4 | -2.48 ( -2.79 - -2.18 ) |
| Nicaragua | Female | 5.51 ( 4.8 - 6.3 ) | 0.63 ( 0.55 - 0.72 ) | 53.01 ( 35.19 - 76.45 ) | 0.27 ( 0.22 - 0.32 ) | 862.49 | -3.54 ( -4 - -3.06 ) |
| Niger | Female | 4.09 ( 3.25 - 5.06 ) | 0.28 ( 0.22 - 0.34 ) | 77.95 ( 59.41 - 99.21 ) | 0.26 ( 0.19 - 0.33 ) | 1806.34 | -0.36 ( -0.47 - -0.25 ) |
| Nigeria | Female | 35.03 ( 26.29 - 45.74 ) | 0.16 ( 0.12 - 0.21 ) | 0.3 ( 0.24 - 0.37 ) | 0.12 ( 0.08 - 0.17 ) | -99.15 | -1.49 ( -1.61 - -1.37 ) |
| North Korea | Female | 43.02 ( 31.83 - 54.58 ) | 0.42 ( 0.31 - 0.53 ) | 19.63 ( 17.29 - 22.48 ) | 0.43 ( 0.33 - 0.54 ) | -54.36 | 0.24 ( 0.2 - 0.29 ) |
| Northern Mariana Islands | Female | 0.04 ( 0.03 - 0.05 ) | 0.53 ( 0.43 - 0.65 ) | 2.59 ( 1.93 - 3.36 ) | 1.03 ( 0.85 - 1.26 ) | 6197.71 | 3.59 ( 2.83 - 4.35 ) |
| Norway | Female | 14.74 ( 13.43 - 16.26 ) | 0.44 ( 0.4 - 0.49 ) | 1305.45 ( 979.69 - 1680.94 ) | 0.45 ( 0.39 - 0.51 ) | 8757.37 | -0.27 ( -0.56 - 0.01 ) |
| Oman | Female | 1.25 ( 0.93 - 1.63 ) | 0.37 ( 0.28 - 0.48 ) | 3.39 ( 2.88 - 4.02 ) | 0.29 ( 0.22 - 0.38 ) | 171.03 | -1.19 ( -1.53 - -0.85 ) |
| Pakistan | Female | 678.18 ( 578.2 - 789.3 ) | 2.39 ( 2.04 - 2.78 ) | 6.32 ( 5.48 - 7.29 ) | 2.15 ( 1.62 - 2.74 ) | -99.07 | -0.88 ( -1.16 - -0.6 ) |
| Palestine | Female | 1.99 ( 1.54 - 2.55 ) | 0.38 ( 0.3 - 0.49 ) | 24.03 ( 18.56 - 30.14 ) | 0.25 ( 0.21 - 0.29 ) | 1107.23 | -1.76 ( -2.01 - -1.51 ) |
| Panama | Female | 3.58 ( 3.25 - 3.94 ) | 0.46 ( 0.42 - 0.51 ) | 6.6 ( 5.07 - 8.31 ) | 0.31 ( 0.27 - 0.36 ) | 84.53 | -1.87 ( -2.26 - -1.49 ) |
| Papua New Guinea | Female | 10.24 ( 8.03 - 12.73 ) | 1.02 ( 0.82 - 1.26 ) | 57.75 ( 45.71 - 72.44 ) | 1.04 ( 0.82 - 1.28 ) | 464.1 | 0.32 ( 0.18 - 0.46 ) |
| Paraguay | Female | 2.89 ( 2.51 - 3.32 ) | 0.25 ( 0.21 - 0.28 ) | 171.29 ( 138.22 - 210.97 ) | 0.24 ( 0.18 - 0.3 ) | 5827.1 | -0.02 ( -0.48 - 0.43 ) |
| Peru | Female | 43.74 ( 37.79 - 50.38 ) | 0.66 ( 0.57 - 0.76 ) | 365.57 ( 315.32 - 426.35 ) | 0.36 ( 0.29 - 0.46 ) | 735.82 | -3 ( -3.4 - -2.59 ) |
| Philippines | Female | 97.32 ( 87 - 107.92 ) | 0.61 ( 0.55 - 0.67 ) | 33.68 ( 28.82 - 39.14 ) | 0.45 ( 0.37 - 0.55 ) | -65.4 | -1.26 ( -1.52 - -1 ) |
| Poland | Female | 209.69 ( 194.43 - 226.02 ) | 0.84 ( 0.77 - 0.9 ) | 16.62 ( 14.13 - 19.55 ) | 1.05 ( 0.9 - 1.22 ) | -92.07 | 0.67 ( 0.4 - 0.93 ) |
| Portugal | Female | 41.59 ( 37.97 - 45.69 ) | 0.54 ( 0.5 - 0.6 ) | 1.2 ( 0.89 - 1.61 ) | 0.29 ( 0.24 - 0.34 ) | -97.11 | -3.03 ( -3.55 - -2.51 ) |
| Puerto Rico | Female | 17.26 ( 15.39 - 19.44 ) | 0.86 ( 0.77 - 0.98 ) | 7.27 ( 5.26 - 9.41 ) | 0.46 ( 0.39 - 0.54 ) | -57.88 | -2.52 ( -2.91 - -2.12 ) |
| Qatar | Female | 0.12 ( 0.1 - 0.15 ) | 0.3 ( 0.24 - 0.37 ) | 104.13 ( 89.54 - 121.8 ) | 0.35 ( 0.26 - 0.45 ) | 85568.91 | 1.58 ( 0.72 - 2.45 ) |
| Republic of Congo | Female | 4.76 ( 3.76 - 5.86 ) | 0.73 ( 0.58 - 0.89 ) | 528.87 ( 480.25 - 580.29 ) | 0.52 ( 0.39 - 0.67 ) | 11008.68 | -1.45 ( -1.65 - -1.25 ) |
| Romania | Female | 80.02 ( 72.74 - 88.42 ) | 0.53 ( 0.48 - 0.58 ) | 15.66 ( 11.74 - 20.09 ) | 0.61 ( 0.52 - 0.72 ) | -80.43 | 0.51 ( 0.3 - 0.73 ) |
| Russia | Female | 535.57 ( 487.07 - 592.63 ) | 0.47 ( 0.42 - 0.52 ) | 0.61 ( 0.52 - 0.7 ) | 0.41 ( 0.37 - 0.46 ) | -99.89 | -0.7 ( -1.06 - -0.33 ) |
| Rwanda | Female | 21.53 ( 17.61 - 26.14 ) | 1.17 ( 0.96 - 1.42 ) | 0.29 ( 0.25 - 0.33 ) | 0.43 ( 0.33 - 0.55 ) | -98.65 | -4.61 ( -5.05 - -4.17 ) |
| Saint Lucia | Female | 0.33 ( 0.3 - 0.37 ) | 0.66 ( 0.59 - 0.73 ) | 0.29 ( 0.25 - 0.33 ) | 0.56 ( 0.48 - 0.64 ) | -12.28 | -1 ( -1.29 - -0.71 ) |
| Saint Vincent | Female | 0.22 ( 0.19 - 0.24 ) | 0.53 ( 0.47 - 0.59 ) | 0.14 ( 0.11 - 0.17 ) | 0.43 ( 0.38 - 0.5 ) | -36.44 | -0.87 ( -1.15 - -0.58 ) |
| Samoa | Female | 0.08 ( 0.06 - 0.1 ) | 0.18 ( 0.14 - 0.23 ) | 0.12 ( 0.09 - 0.16 ) | 0.19 ( 0.15 - 0.24 ) | 53.91 | 0.32 ( 0.2 - 0.44 ) |
| Sao Tome and Principe | Female | 0.06 ( 0.05 - 0.08 ) | 0.17 ( 0.15 - 0.21 ) | 26.9 ( 20.71 - 35.1 ) | 0.21 ( 0.16 - 0.29 ) | 41959.49 | 0.58 ( 0.37 - 0.78 ) |
| Saudi Arabia | Female | 6.97 ( 5.33 - 8.94 ) | 0.24 ( 0.19 - 0.31 ) | 9.52 ( 7.17 - 12.22 ) | 0.3 ( 0.24 - 0.37 ) | 36.74 | 0.96 ( 0.74 - 1.18 ) |
| Senegal | Female | 4.25 ( 3.43 - 5.15 ) | 0.25 ( 0.2 - 0.3 ) | 66.17 ( 56.24 - 77.08 ) | 0.25 ( 0.19 - 0.32 ) | 1457.93 | 0.15 ( 0.03 - 0.28 ) |
| Serbia | Female | 43.3 ( 36.73 - 51.45 ) | 0.68 ( 0.58 - 0.8 ) | 0.5 ( 0.44 - 0.58 ) | 0.84 ( 0.71 - 0.99 ) | -98.84 | 1.06 ( 0.83 - 1.28 ) |
| Seychelles | Female | 0.39 ( 0.35 - 0.45 ) | 1.22 ( 1.07 - 1.4 ) | 6.33 ( 4.88 - 8.15 ) | 0.89 ( 0.77 - 1.03 ) | 1516.34 | -1.08 ( -1.21 - -0.96 ) |
| Sierra Leone | Female | 2.93 ( 2.3 - 3.67 ) | 0.3 ( 0.24 - 0.38 ) | 11.04 ( 9.21 - 13.2 ) | 0.37 ( 0.29 - 0.47 ) | 276.53 | 1.2 ( 0.99 - 1.41 ) |
| Singapore | Female | 6.38 ( 5.6 - 7.36 ) | 0.52 ( 0.46 - 0.6 ) | 18.88 ( 15.41 - 22.93 ) | 0.3 ( 0.25 - 0.36 ) | 195.73 | -1.77 ( -2.04 - -1.49 ) |
| Slovakia | Female | 16.07 ( 14.14 - 18.36 ) | 0.48 ( 0.43 - 0.55 ) | 11.73 ( 9.66 - 14.25 ) | 0.42 ( 0.33 - 0.51 ) | -26.99 | -0.51 ( -0.61 - -0.41 ) |
| Slovenia | Female | 7.54 ( 6.6 - 8.57 ) | 0.53 ( 0.46 - 0.6 ) | 1.35 ( 1.07 - 1.67 ) | 0.61 ( 0.5 - 0.75 ) | -82.13 | 0.06 ( -0.2 - 0.31 ) |
| Solomon Islands | Female | 0.6 ( 0.46 - 0.76 ) | 0.83 ( 0.66 - 1.02 ) | 22.94 ( 15.43 - 31.88 ) | 0.8 ( 0.65 - 0.97 ) | 3738.23 | 0.04 ( -0.07 - 0.15 ) |
| Somalia | Female | 14.3 ( 8.47 - 20.67 ) | 0.92 ( 0.59 - 1.3 ) | 102.46 ( 95.83 - 109.98 ) | 0.63 ( 0.43 - 0.87 ) | 616.23 | -1.92 ( -2.13 - -1.7 ) |
| South Africa | Female | 73.88 ( 67.79 - 80.87 ) | 0.56 ( 0.51 - 0.62 ) | 165.65 ( 138.75 - 195.43 ) | 0.39 ( 0.36 - 0.42 ) | 124.23 | -1.72 ( -2.37 - -1.05 ) |
| South Korea | Female | 193.29 ( 174.07 - 212.47 ) | 1.12 ( 1.01 - 1.22 ) | 10.5 ( 7.18 - 14.97 ) | 0.37 ( 0.31 - 0.43 ) | -94.57 | -5.94 ( -6.68 - -5.2 ) |
| South Sudan | Female | 9.01 ( 5.71 - 12.99 ) | 0.77 ( 0.51 - 1.07 ) | 205.85 ( 172.56 - 243.46 ) | 0.52 ( 0.36 - 0.73 ) | 2183.51 | -1.9 ( -2.09 - -1.72 ) |
| Spain | Female | 96.34 ( 87.82 - 105.54 ) | 0.32 ( 0.29 - 0.36 ) | 60.87 ( 44.35 - 79.77 ) | 0.5 ( 0.42 - 0.6 ) | -36.82 | 2.33 ( 2.08 - 2.57 ) |
| Sri Lanka | Female | 12.19 ( 10.62 - 14.03 ) | 0.22 ( 0.19 - 0.25 ) | 87.12 ( 60.94 - 118.54 ) | 0.44 ( 0.32 - 0.58 ) | 614.5 | 4.04 ( 3.55 - 4.53 ) |
| Sudan | Female | 58.58 ( 44.3 - 73.8 ) | 1.16 ( 0.88 - 1.45 ) | 0.99 ( 0.84 - 1.16 ) | 0.91 ( 0.65 - 1.22 ) | -98.31 | -0.94 ( -0.95 - -0.92 ) |
| Suriname | Female | 0.49 ( 0.43 - 0.55 ) | 0.36 ( 0.32 - 0.4 ) | 1.73 ( 1.17 - 2.42 ) | 0.32 ( 0.27 - 0.37 ) | 255.6 | -0.55 ( -0.78 - -0.31 ) |
| Swaziland | Female | 1.25 ( 0.97 - 1.56 ) | 0.7 ( 0.55 - 0.87 ) | 33.14 ( 28.08 - 38.38 ) | 0.51 ( 0.35 - 0.7 ) | 2549.72 | -0.52 ( -1.05 - 0 ) |
| Sweden | Female | 30.33 ( 26.73 - 34.88 ) | 0.43 ( 0.37 - 0.5 ) | 38.25 ( 31.91 - 45.52 ) | 0.37 ( 0.31 - 0.43 ) | 26.11 | -0.89 ( -1.09 - -0.69 ) |
| Switzerland | Female | 36.26 ( 31.88 - 40.86 ) | 0.68 ( 0.6 - 0.78 ) | 20.45 ( 15.94 - 25.72 ) | 0.5 ( 0.41 - 0.6 ) | -43.59 | -1.32 ( -1.47 - -1.18 ) |
| Syria | Female | 10.58 ( 8.78 - 12.62 ) | 0.38 ( 0.32 - 0.44 ) | 38.19 ( 32.3 - 44.92 ) | 0.31 ( 0.24 - 0.38 ) | 260.98 | -1.15 ( -1.59 - -0.7 ) |
| Tajikistan | Female | 7.46 ( 6.64 - 8.33 ) | 0.45 ( 0.4 - 0.5 ) | 60.19 ( 46.99 - 75.41 ) | 0.55 ( 0.45 - 0.67 ) | 707.09 | 0.42 ( 0.11 - 0.73 ) |
| Tanzania | Female | 50.83 ( 38.12 - 62.58 ) | 0.8 ( 0.61 - 0.98 ) | 171.94 ( 143.5 - 204.53 ) | 0.44 ( 0.35 - 0.55 ) | 238.28 | -2.73 ( -3.05 - -2.41 ) |
| Thailand | Female | 152.93 ( 135.06 - 172.6 ) | 0.77 ( 0.68 - 0.87 ) | 1.01 ( 0.79 - 1.29 ) | 0.32 ( 0.27 - 0.39 ) | -99.34 | -3.93 ( -4.21 - -3.64 ) |
| Timor-Leste | Female | 1.55 ( 1.21 - 2 ) | 0.99 ( 0.79 - 1.26 ) | 2.38 ( 1.86 - 3.03 ) | 0.58 ( 0.46 - 0.73 ) | 53.15 | -2.15 ( -2.34 - -1.96 ) |
| Tobago | Female | 2.15 ( 1.92 - 2.39 ) | 0.48 ( 0.43 - 0.53 ) | 1.83 ( 1.36 - 2.4 ) | 0.19 ( 0.15 - 0.26 ) | -14.71 | -4.7 ( -5.4 - -3.99 ) |
| Togo | Female | 2.15 ( 1.72 - 2.65 ) | 0.3 ( 0.24 - 0.36 ) | 4.9 ( 3.64 - 6.47 ) | 0.25 ( 0.19 - 0.32 ) | 128.13 | -0.65 ( -0.78 - -0.52 ) |
| Tonga | Female | 0.14 ( 0.12 - 0.17 ) | 0.49 ( 0.42 - 0.57 ) | 0.22 ( 0.17 - 0.27 ) | 0.5 ( 0.39 - 0.62 ) | 51.52 | 0.12 ( 0.05 - 0.19 ) |
| Trinidad | Female | 2.15 ( 1.92 - 2.39 ) | 0.48 ( 0.43 - 0.53 ) | 1.83 ( 1.36 - 2.4 ) | 0.19 ( 0.15 - 0.26 ) | -14.71 | -4.7 ( -5.4 - -3.99 ) |
| Tunisia | Female | 12.76 ( 10.97 - 14.74 ) | 0.49 ( 0.42 - 0.56 ) | 31.37 ( 22.85 - 42.41 ) | 0.49 ( 0.36 - 0.67 ) | 145.89 | -0.23 ( -0.38 - -0.09 ) |
| Turkey | Female | 143.78 ( 121.97 - 168.65 ) | 0.72 ( 0.62 - 0.84 ) | 262.98 ( 220.28 - 314.97 ) | 0.56 ( 0.47 - 0.67 ) | 82.91 | -1.03 ( -1.46 - -0.6 ) |
| Turkmenistan | Female | 7.4 ( 6.67 - 8.18 ) | 0.62 ( 0.56 - 0.69 ) | 15.77 ( 13.24 - 18.75 ) | 0.7 ( 0.59 - 0.82 ) | 113.04 | -0.21 ( -1.88 - 1.49 ) |
| Uganda | Female | 31.08 ( 24.84 - 38.03 ) | 0.84 ( 0.68 - 1.03 ) | 33.26 ( 26.33 - 41.77 ) | 0.4 ( 0.32 - 0.51 ) | 7.01 | -3.54 ( -3.92 - -3.15 ) |
| UK | Female | 333.4 ( 321.83 - 344.89 ) | 0.68 ( 0.66 - 0.71 ) | 385.53 ( 367.14 - 403.72 ) | 0.65 ( 0.62 - 0.68 ) | 15.64 | -0.45 ( -0.59 - -0.32 ) |
| Ukraine | Female | 153.18 ( 138.03 - 170.34 ) | 0.35 ( 0.32 - 0.4 ) | 137.2 ( 116.51 - 162.57 ) | 0.35 ( 0.29 - 0.43 ) | -10.43 | -0.11 ( -0.47 - 0.26 ) |
| United Arab Emirates | Female | 2.33 ( 1.61 - 3.51 ) | 1.32 ( 0.93 - 1.92 ) | 16.9 ( 11.71 - 23.68 ) | 1.47 ( 1.08 - 2.02 ) | 625.99 | 0.47 ( 0.17 - 0.76 ) |
| Uruguay | Female | 11.18 ( 9.88 - 12.48 ) | 0.51 ( 0.45 - 0.57 ) | 20.08 ( 16.41 - 24.25 ) | 0.69 ( 0.56 - 0.84 ) | 79.56 | 1.18 ( 1 - 1.36 ) |
| USA | Female | 1851.8 ( 1791.24 - 1921.79 ) | 1.09 ( 1.05 - 1.13 ) | 2628.19 ( 2487.29 - 2771.8 ) | 0.94 ( 0.89 - 0.99 ) | 41.93 | -1.16 ( -1.45 - -0.86 ) |
| Uzbekistan | Female | 44 ( 39.31 - 48.49 ) | 0.63 ( 0.56 - 0.7 ) | 222.28 ( 177.38 - 273.96 ) | 1.58 ( 1.29 - 1.92 ) | 405.23 | 3.96 ( 2.85 - 5.08 ) |
| Vanuatu | Female | 0.21 ( 0.16 - 0.28 ) | 0.66 ( 0.52 - 0.86 ) | 0.56 ( 0.42 - 0.73 ) | 0.68 ( 0.51 - 0.87 ) | 162.35 | 0.18 ( 0.09 - 0.26 ) |
| Venezuela | Female | 52.42 ( 47.6 - 57.35 ) | 1.02 ( 0.92 - 1.11 ) | 104.2 ( 81.03 - 130.01 ) | 0.69 ( 0.54 - 0.86 ) | 98.79 | -1.74 ( -1.96 - -1.52 ) |
| Vietnam | Female | 94.15 ( 75.22 - 112.83 ) | 0.39 ( 0.31 - 0.47 ) | 170.94 ( 130.51 - 218.38 ) | 0.32 ( 0.25 - 0.41 ) | 81.56 | -0.93 ( -1.07 - -0.79 ) |
| Virgin Islands | Female | 0.27 ( 0.23 - 0.32 ) | 0.58 ( 0.49 - 0.68 ) | 0.6 ( 0.49 - 0.74 ) | 0.58 ( 0.48 - 0.72 ) | 121.54 | 0.1 ( 0.02 - 0.17 ) |
| Yemen | Female | 35.94 ( 22.73 - 49.07 ) | 1.25 ( 0.83 - 1.7 ) | 79.92 ( 57.06 - 108.44 ) | 1.09 ( 0.79 - 1.47 ) | 122.38 | -0.63 ( -0.7 - -0.57 ) |
| Zambia | Female | 19.92 ( 15.3 - 25.04 ) | 1.22 ( 0.96 - 1.51 ) | 20.27 ( 15 - 26.36 ) | 0.55 ( 0.41 - 0.72 ) | 1.76 | -3.98 ( -4.58 - -3.38 ) |
| Zimbabwe | Female | 10.13 ( 8.33 - 12.42 ) | 0.45 ( 0.37 - 0.54 ) | 20.71 ( 15.61 - 26.52 ) | 0.49 ( 0.38 - 0.62 ) | 104.43 | 1.56 ( 0.77 - 2.37 ) |
| Afghanistan | Male | 215.22 ( 103.07 - 314.7 ) | 5.29 ( 2.6 - 7.69 ) | 279.58 ( 200.95 - 371.75 ) | 5.3 ( 3.89 - 7.25 ) | 29.9 | -0.01 ( -0.24 - 0.22 ) |
| Albania | Male | 65.9 ( 60 - 72.99 ) | 6.25 ( 5.73 - 6.83 ) | 110.6 ( 83.68 - 143.22 ) | 5.49 ( 4.16 - 7.07 ) | 67.83 | -0.25 ( -0.52 - 0.02 ) |
| Algeria | Male | 260.66 ( 223.09 - 299.66 ) | 4.05 ( 3.48 - 4.63 ) | 519.45 ( 447.82 - 606.4 ) | 3.03 ( 2.6 - 3.55 ) | 99.29 | -1.06 ( -1.15 - -0.98 ) |
| American Samoa | Male | 0.38 ( 0.32 - 0.48 ) | 3.36 ( 2.82 - 4.34 ) | 0.55 ( 0.45 - 0.67 ) | 2.78 ( 2.27 - 3.33 ) | 44.84 | -0.64 ( -0.82 - -0.46 ) |
| Andorra | Male | 1.39 ( 0.99 - 1.97 ) | 4.49 ( 3.23 - 6.36 ) | 2.35 ( 1.81 - 3.13 ) | 3.52 ( 2.71 - 4.68 ) | 69.96 | -1.09 ( -1.24 - -0.94 ) |
| Angola | Male | 87.95 ( 55.75 - 117.37 ) | 4.19 ( 2.8 - 5.46 ) | 167.89 ( 132 - 204.82 ) | 3.39 ( 2.66 - 4.16 ) | 90.89 | -0.96 ( -1.05 - -0.86 ) |
| Antigua | Male | 1.11 ( 0.99 - 1.23 ) | 4.87 ( 4.39 - 5.42 ) | 2.24 ( 1.96 - 2.58 ) | 4.66 ( 4.06 - 5.34 ) | 102.92 | -0.19 ( -0.31 - -0.08 ) |
| Argentina | Male | 1136.31 ( 1071.78 - 1204.59 ) | 7.45 ( 7.03 - 7.88 ) | 1195.83 ( 1019.53 - 1408.43 ) | 5.08 ( 4.34 - 5.99 ) | 5.24 | -1.94 ( -2.22 - -1.66 ) |
| Armenia | Male | 124.64 ( 113.89 - 137.07 ) | 9.32 ( 8.58 - 10.2 ) | 127.18 ( 113.47 - 141.8 ) | 6.86 ( 6.13 - 7.62 ) | 2.04 | -1.67 ( -1.89 - -1.46 ) |
| Australia | Male | 524.12 ( 492.95 - 558.13 ) | 5.68 ( 5.35 - 6.05 ) | 712.14 ( 596.28 - 843.68 ) | 3.87 ( 3.24 - 4.63 ) | 35.87 | -1.8 ( -2 - -1.61 ) |
| Austria | Male | 316.78 ( 292.21 - 342.12 ) | 6.81 ( 6.29 - 7.37 ) | 297.02 ( 260.86 - 338.66 ) | 4 ( 3.51 - 4.56 ) | -6.24 | -2.32 ( -2.49 - -2.14 ) |
| Azerbaijan | Male | 131.18 ( 118.96 - 144.94 ) | 5.38 ( 4.91 - 5.91 ) | 245.28 ( 201.55 - 298.24 ) | 5.34 ( 4.41 - 6.43 ) | 86.99 | -1.09 ( -1.53 - -0.65 ) |
| Bahamas | Male | 5.29 ( 4.73 - 5.92 ) | 7.34 ( 6.59 - 8.17 ) | 12.96 ( 11.01 - 14.95 ) | 7.17 ( 6.13 - 8.23 ) | 144.9 | 0.06 ( -0.06 - 0.19 ) |
| Bahrain | Male | 3.68 ( 3.16 - 4.29 ) | 4.52 ( 3.86 - 5.26 ) | 7.84 ( 6.54 - 9.49 ) | 1.85 ( 1.53 - 2.21 ) | 113.15 | -4.69 ( -5.26 - -4.13 ) |
| Bangladesh | Male | 2063.84 ( 1708.43 - 2477.81 ) | 7.63 ( 6.35 - 9.15 ) | 2584.34 ( 1848.8 - 3273.26 ) | 3.95 ( 2.81 - 4.95 ) | 25.22 | -2.41 ( -2.52 - -2.3 ) |
| Barbados | Male | 4.86 ( 4.41 - 5.35 ) | 4.01 ( 3.64 - 4.41 ) | 9.2 ( 7.89 - 10.66 ) | 4.22 ( 3.62 - 4.87 ) | 89.51 | 0.1 ( -0.08 - 0.28 ) |
| Barbuda | Male | 1.11 ( 0.99 - 1.23 ) | 4.87 ( 4.39 - 5.42 ) | 2.24 ( 1.96 - 2.58 ) | 4.66 ( 4.06 - 5.34 ) | 102.92 | -0.19 ( -0.31 - -0.08 ) |
| Belarus | Male | 634.29 ( 595.03 - 676.91 ) | 11.63 ( 10.97 - 12.37 ) | 636.6 ( 546.53 - 736.18 ) | 9.75 ( 8.41 - 11.17 ) | 0.36 | -1.54 ( -1.94 - -1.13 ) |
| Belgium | Male | 742.47 ( 680.32 - 813.44 ) | 11.31 ( 10.38 - 12.39 ) | 533.67 ( 464.02 - 616.68 ) | 5.62 ( 4.9 - 6.5 ) | -28.12 | -2.99 ( -3.18 - -2.81 ) |
| Belize | Male | 1.27 ( 1.13 - 1.43 ) | 2.69 ( 2.38 - 3.02 ) | 5 ( 4.47 - 5.54 ) | 3.64 ( 3.27 - 4.03 ) | 292.81 | 0.9 ( 0.52 - 1.29 ) |
| Benin | Male | 23.94 ( 19.36 - 29.05 ) | 2.45 ( 1.98 - 2.97 ) | 53.97 ( 40.86 - 71.25 ) | 2.53 ( 1.96 - 3.32 ) | 125.4 | 0.45 ( 0.31 - 0.59 ) |
| Bermuda | Male | 2.22 ( 2 - 2.45 ) | 7.88 ( 7.11 - 8.69 ) | 4.44 ( 3.92 - 5.04 ) | 8.08 ( 7.16 - 9.14 ) | 100.2 | 0.42 ( 0.24 - 0.61 ) |
| Bhutan | Male | 8.74 ( 6.52 - 12.87 ) | 6.38 ( 4.75 - 9.4 ) | 13.34 ( 9.42 - 21.1 ) | 4.2 ( 2.98 - 6.64 ) | 52.59 | -1.56 ( -1.7 - -1.43 ) |
| Bolivia | Male | 50.45 ( 41.07 - 60.3 ) | 3.31 ( 2.72 - 3.93 ) | 90.35 ( 66.76 - 122.25 ) | 2.28 ( 1.69 - 3.04 ) | 79.1 | -1.41 ( -1.5 - -1.31 ) |
| Bosnia and Herzegovina | Male | 173.05 ( 159.24 - 187.04 ) | 8.81 ( 8.16 - 9.51 ) | 174.69 ( 151.2 - 200.93 ) | 6.28 ( 5.47 - 7.19 ) | 0.95 | -1.92 ( -2.21 - -1.63 ) |
| Botswana | Male | 12.55 ( 10.13 - 15.73 ) | 4.61 ( 3.76 - 5.66 ) | 16.63 ( 12.89 - 24.17 ) | 2.86 ( 2.25 - 3.98 ) | 32.51 | -1.93 ( -2.09 - -1.77 ) |
| Brazil | Male | 2581.18 ( 2509.42 - 2679.26 ) | 5.75 ( 5.59 - 5.97 ) | 6199.76 ( 5987.5 - 6434.73 ) | 5.79 ( 5.6 - 6 ) | 140.19 | -0.1 ( -0.19 - -0.01 ) |
| Brunei | Male | 3.11 ( 2.64 - 3.67 ) | 6.18 ( 5.21 - 7.27 ) | 4.96 ( 4.17 - 5.93 ) | 3.39 ( 2.86 - 4.05 ) | 59.57 | -2.76 ( -3.02 - -2.5 ) |
| Bulgaria | Male | 443.57 ( 415.48 - 473.97 ) | 7.29 ( 6.84 - 7.81 ) | 641.37 ( 565.68 - 725.23 ) | 10.79 ( 9.46 - 12.26 ) | 44.59 | 1.47 ( 1.18 - 1.76 ) |
| Burkina Faso | Male | 52.89 ( 37.96 - 69.68 ) | 2.49 ( 1.83 - 3.26 ) | 117.65 ( 80.8 - 148.06 ) | 2.93 ( 2.04 - 3.65 ) | 122.44 | 0.87 ( 0.73 - 1.01 ) |
| Burundi | Male | 56.85 ( 39.84 - 75.11 ) | 5.36 ( 3.88 - 6.99 ) | 66.82 ( 47.18 - 89.55 ) | 2.86 ( 2.07 - 3.76 ) | 17.53 | -2.76 ( -2.98 - -2.54 ) |
| Cambodia | Male | 91.58 ( 70.57 - 115.84 ) | 4.56 ( 3.55 - 5.82 ) | 192.69 ( 148.16 - 265.13 ) | 4.09 ( 3.17 - 5.82 ) | 110.41 | -0.34 ( -0.44 - -0.25 ) |
| Cameroon | Male | 62.36 ( 49.47 - 77.24 ) | 2.78 ( 2.24 - 3.42 ) | 173.02 ( 124.87 - 230.2 ) | 3.15 ( 2.34 - 4.21 ) | 177.47 | 0.73 ( 0.54 - 0.91 ) |
| Canada | Male | 1005.38 ( 914.04 - 1100.53 ) | 6.74 ( 6.13 - 7.37 ) | 1302.26 ( 1123.35 - 1512.66 ) | 4.24 ( 3.66 - 4.9 ) | 29.53 | -2.07 ( -2.28 - -1.86 ) |
| Cape Verde | Male | 3.07 ( 2.57 - 3.61 ) | 3.27 ( 2.71 - 3.84 ) | 3.9 ( 3.35 - 4.55 ) | 2.08 ( 1.79 - 2.4 ) | 26.83 | -1.77 ( -1.96 - -1.58 ) |
| Central African Republic | Male | 27.85 ( 15.93 - 36.34 ) | 4.72 ( 2.92 - 6.01 ) | 42.02 ( 26.27 - 57.42 ) | 3.89 ( 2.72 - 5.13 ) | 50.86 | -0.87 ( -0.98 - -0.77 ) |
| Chad | Male | 28.21 ( 18.57 - 35.77 ) | 2.05 ( 1.36 - 2.58 ) | 75.72 ( 54.08 - 97.37 ) | 2.62 ( 1.89 - 3.33 ) | 168.42 | 1.3 ( 1.09 - 1.51 ) |
| Chile | Male | 148.56 ( 137.97 - 159.79 ) | 3.23 ( 3 - 3.46 ) | 261.71 ( 221.67 - 307.22 ) | 2.48 ( 2.1 - 2.91 ) | 76.17 | -0.99 ( -1.2 - -0.78 ) |
| China | Male | 10528.46 ( 10064.7 - 11164.66 ) | 2.44 ( 2.34 - 2.59 ) | 33299.59 ( 31440.92 - 35421.62 ) | 3.37 ( 3.19 - 3.58 ) | 216.28 | 1.2 ( 0.81 - 1.59 ) |
| Colombia | Male | 339.11 ( 320.05 - 365.22 ) | 3.93 ( 3.72 - 4.22 ) | 581.17 ( 493.47 - 677.96 ) | 2.37 ( 2.01 - 2.76 ) | 71.38 | -2.68 ( -2.95 - -2.4 ) |
| Comoros | Male | 3.88 ( 2.89 - 5.24 ) | 3.58 ( 2.72 - 4.8 ) | 5.27 ( 3.76 - 7.97 ) | 2.45 ( 1.78 - 3.77 ) | 36 | -1.7 ( -1.91 - -1.49 ) |
| Costa Rica | Male | 37.19 ( 34.46 - 40.19 ) | 4.33 ( 4.01 - 4.69 ) | 85.47 ( 73.28 - 97.12 ) | 3.76 ( 3.23 - 4.27 ) | 129.83 | -0.8 ( -1.12 - -0.48 ) |
| Croatia | Male | 397.77 ( 369.63 - 426.13 ) | 13.58 ( 12.7 - 14.49 ) | 317.86 ( 280.31 - 357.69 ) | 8.72 ( 7.71 - 9.82 ) | -20.09 | -1.75 ( -2.05 - -1.44 ) |
| Cuba | Male | 493.53 ( 463.54 - 524.07 ) | 9.6 ( 9.02 - 10.19 ) | 1383.73 ( 1161.51 - 1620.91 ) | 15.67 ( 13.13 - 18.3 ) | 180.37 | 1.86 ( 1.75 - 1.98 ) |
| Cyprus | Male | 16.18 ( 14.07 - 18.68 ) | 4.1 ( 3.58 - 4.71 ) | 37.91 ( 31.34 - 44.71 ) | 4.23 ( 3.52 - 4.96 ) | 134.29 | 0.33 ( 0.03 - 0.64 ) |
| Czech Republic | Male | 466.89 ( 439.06 - 496.24 ) | 7.85 ( 7.39 - 8.33 ) | 480.14 ( 424.12 - 541.99 ) | 5.37 ( 4.75 - 6.06 ) | 2.84 | -1.4 ( -1.49 - -1.3 ) |
| Democratic Republic of the Congo | Male | 243.5 ( 180.32 - 317.44 ) | 3.2 ( 2.45 - 4.05 ) | 429.38 ( 276.24 - 566.55 ) | 2.76 ( 1.86 - 3.59 ) | 76.34 | -0.66 ( -0.76 - -0.56 ) |
| Denmark | Male | 238.77 ( 221.68 - 257.24 ) | 6.87 ( 6.38 - 7.4 ) | 233.46 ( 205.55 - 267.97 ) | 4.57 ( 4.03 - 5.21 ) | -2.22 | -1.79 ( -1.92 - -1.66 ) |
| Djibouti | Male | 3.02 ( 1.9 - 4.87 ) | 3.42 ( 2.23 - 5.39 ) | 8.67 ( 5.61 - 17.04 ) | 2.6 ( 1.73 - 4.97 ) | 187.12 | -1.37 ( -1.53 - -1.2 ) |
| Dominica | Male | 1.34 ( 1.21 - 1.48 ) | 4.52 ( 4.07 - 4.95 ) | 2.45 ( 2.16 - 2.77 ) | 5.43 ( 4.79 - 6.14 ) | 82.82 | 0.58 ( 0.44 - 0.71 ) |
| Dominican Republic | Male | 54.89 ( 47.76 - 63.29 ) | 2.87 ( 2.51 - 3.33 ) | 148.02 ( 115.97 - 180.91 ) | 3.33 ( 2.61 - 4.08 ) | 169.64 | 0.9 ( 0.48 - 1.33 ) |
| Ecuador | Male | 49.79 ( 46.52 - 53.52 ) | 1.87 ( 1.75 - 2.01 ) | 102.07 ( 88.02 - 117.88 ) | 1.46 ( 1.26 - 1.68 ) | 104.99 | -0.65 ( -1 - -0.3 ) |
| Egypt | Male | 245.37 ( 218.95 - 276.27 ) | 1.57 ( 1.41 - 1.75 ) | 584.26 ( 348.61 - 737.41 ) | 1.74 ( 1.05 - 2.19 ) | 138.11 | 0.49 ( 0.29 - 0.7 ) |
| El Salvador | Male | 27.06 ( 24.18 - 30.23 ) | 1.91 ( 1.71 - 2.13 ) | 57.06 ( 45.1 - 71.71 ) | 2.36 ( 1.87 - 2.98 ) | 110.86 | 1.02 ( 0.73 - 1.31 ) |
| Equatorial Guinea | Male | 4.48 ( 2.19 - 6.28 ) | 4.75 ( 2.52 - 6.47 ) | 5.44 ( 3.81 - 7.51 ) | 2.8 ( 2.01 - 3.79 ) | 21.56 | -2.47 ( -2.9 - -2.04 ) |
| Eritrea | Male | 29.57 ( 19.55 - 40.17 ) | 6.06 ( 4.3 - 7.96 ) | 42.45 ( 29.88 - 67.85 ) | 3.86 ( 2.84 - 6.2 ) | 43.55 | -2.15 ( -2.44 - -1.86 ) |
| Estonia | Male | 72.64 ( 67.11 - 79.18 ) | 8.82 ( 8.17 - 9.58 ) | 66.36 ( 54.65 - 81.81 ) | 6.9 ( 5.67 - 8.51 ) | -8.65 | -1.17 ( -1.47 - -0.87 ) |
| Ethiopia | Male | 278.7 ( 199.94 - 383.67 ) | 2.52 ( 1.86 - 3.43 ) | 310.32 ( 223.47 - 519.22 ) | 1.48 ( 1.06 - 2.48 ) | 11.35 | -2.25 ( -2.38 - -2.11 ) |
| Fiji | Male | 3.07 ( 2.56 - 3.66 ) | 1.76 ( 1.49 - 2.09 ) | 7.63 ( 6.3 - 9.25 ) | 2.37 ( 1.98 - 2.87 ) | 148.57 | 1.57 ( 1.33 - 1.8 ) |
| Finland | Male | 117.77 ( 105.96 - 130.92 ) | 4 ( 3.61 - 4.43 ) | 134.03 ( 115.34 - 157.64 ) | 2.62 ( 2.26 - 3.07 ) | 13.81 | -1.53 ( -1.73 - -1.32 ) |
| France | Male | 5967.32 ( 5521.69 - 6497.55 ) | 17.24 ( 15.99 - 18.77 ) | 4152.66 ( 3622.05 - 4773.98 ) | 7.74 ( 6.71 - 8.85 ) | -30.41 | -3.28 ( -3.73 - -2.83 ) |
| Gabon | Male | 12.21 ( 9.79 - 15.27 ) | 4.66 ( 3.8 - 5.87 ) | 18.38 ( 14.5 - 27.27 ) | 3.61 ( 2.87 - 5.48 ) | 50.55 | -0.98 ( -1.03 - -0.93 ) |
| Gambia | Male | 2.66 ( 1.99 - 3.46 ) | 1.4 ( 1.05 - 1.8 ) | 229.44 ( 204.07 - 256.32 ) | 1.36 ( 1.07 - 1.78 ) | 8513.23 | 0.11 ( 0.02 - 0.2 ) |
| Georgia | Male | 247.46 ( 226.26 - 268.66 ) | 8.93 ( 8.2 - 9.65 ) | 4091.51 ( 3449.9 - 4767.23 ) | 9.32 ( 8.31 - 10.33 ) | 1553.43 | 0.41 ( -0.24 - 1.06 ) |
| Germany | Male | 3533.28 ( 3277.83 - 3802.83 ) | 6.84 ( 6.36 - 7.35 ) | 227.76 ( 140.97 - 286.11 ) | 5.32 ( 4.47 - 6.21 ) | -93.55 | -1.5 ( -1.75 - -1.26 ) |
| Ghana | Male | 73.97 ( 51.51 - 95.26 ) | 2.35 ( 1.66 - 2.99 ) | 818 ( 721.99 - 933.28 ) | 3.35 ( 2.15 - 4.13 ) | 1005.9 | 1.97 ( 1.67 - 2.28 ) |
| Greece | Male | 628.38 ( 576.67 - 685.03 ) | 8.71 ( 8.03 - 9.48 ) | 1.64 ( 1.44 - 1.86 ) | 8.27 ( 7.27 - 9.47 ) | -99.74 | 0.01 ( -0.13 - 0.15 ) |
| Greenland | Male | 0.83 ( 0.73 - 0.95 ) | 5.02 ( 4.46 - 5.63 ) | 3.13 ( 2.77 - 3.49 ) | 4.46 ( 3.92 - 5.04 ) | 275.68 | -0.47 ( -0.81 - -0.14 ) |
| Grenada | Male | 1.2 ( 1.09 - 1.33 ) | 4.12 ( 3.73 - 4.53 ) | 3.57 ( 3.04 - 4.15 ) | 4.34 ( 3.84 - 4.85 ) | 196.09 | 0.44 ( 0.12 - 0.76 ) |
| Grenadines | Male | 1.75 ( 1.56 - 1.92 ) | 5.32 ( 4.78 - 5.84 ) | 77.4 ( 66.6 - 89.1 ) | 6.5 ( 5.83 - 7.3 ) | 4334.51 | 0.68 ( 0.51 - 0.84 ) |
| Guam | Male | 1.71 ( 1.44 - 2.24 ) | 4.55 ( 3.89 - 5.81 ) | 78.94 ( 58.18 - 100.74 ) | 4.1 ( 3.51 - 4.72 ) | 4512.43 | -0.72 ( -1.05 - -0.39 ) |
| Guatemala | Male | 38.42 ( 35.57 - 41.52 ) | 2.1 ( 1.94 - 2.27 ) | 11.03 ( 8.24 - 14.11 ) | 1.56 ( 1.35 - 1.8 ) | -71.29 | -1.8 ( -2.12 - -1.49 ) |
| Guinea | Male | 34.44 ( 27.91 - 42.38 ) | 2.04 ( 1.66 - 2.51 ) | 7.42 ( 6.16 - 8.81 ) | 2.93 ( 2.2 - 3.71 ) | -78.47 | 1.89 ( 1.7 - 2.08 ) |
| Guinea-Bissau | Male | 7.5 ( 5.08 - 9.63 ) | 3.68 ( 2.58 - 4.64 ) | 175.94 ( 122.99 - 277.16 ) | 3.41 ( 2.63 - 4.26 ) | 2244.51 | 0.03 ( -0.11 - 0.16 ) |
| Guyana | Male | 4.4 ( 4.02 - 4.82 ) | 2.3 ( 2.11 - 2.52 ) | 61.12 ( 46.22 - 77.71 ) | 2.43 ( 2.02 - 2.87 ) | 1287.52 | 0.66 ( 0.41 - 0.92 ) |
| Haiti | Male | 105.45 ( 77.13 - 150.39 ) | 6.41 ( 4.7 - 9.3 ) | 773.14 ( 692.19 - 875.17 ) | 5.65 ( 3.99 - 8.97 ) | 633.16 | -0.32 ( -0.45 - -0.18 ) |
| Honduras | Male | 24.18 ( 20.44 - 28.41 ) | 2.17 ( 1.83 - 2.53 ) | 7.57 ( 6.65 - 8.76 ) | 2.1 ( 1.59 - 2.64 ) | -68.69 | -0.22 ( -0.32 - -0.11 ) |
| Hungary | Male | 774.08 ( 727.76 - 822.96 ) | 12.1 ( 11.39 - 12.84 ) | 30710.64 ( 28769.9 - 32772.79 ) | 10.24 ( 9.15 - 11.6 ) | 3867.4 | -0.7 ( -1.07 - -0.34 ) |
| Iceland | Male | 4.98 ( 4.36 - 5.65 ) | 3.78 ( 3.31 - 4.26 ) | 3066.82 ( 2389.75 - 5041.57 ) | 3.06 ( 2.7 - 3.53 ) | 61493.92 | -1.45 ( -1.91 - -0.98 ) |
| India | Male | 18319.63 ( 15527.36 - 20438.64 ) | 6.8 ( 5.79 - 7.58 ) | 1594.16 ( 1476.89 - 1723.29 ) | 5.52 ( 5.15 - 5.88 ) | -91.3 | -0.87 ( -1.12 - -0.61 ) |
| Indonesia | Male | 1313.68 ( 1088.53 - 1863.78 ) | 2.67 ( 2.22 - 3.75 ) | 245.92 ( 218.67 - 274.56 ) | 3.09 ( 2.44 - 5.01 ) | -81.28 | 0.64 ( 0.58 - 0.7 ) |
| Iran | Male | 628.99 ( 567.97 - 749.17 ) | 4.44 ( 4.02 - 5.27 ) | 162.36 ( 138.95 - 187.66 ) | 4.53 ( 4.2 - 4.87 ) | -74.19 | 0.57 ( 0.25 - 0.89 ) |
| Iraq | Male | 221.77 ( 166.76 - 274.8 ) | 5.55 ( 4.22 - 6.86 ) | 155.4 ( 135.04 - 178.52 ) | 2.07 ( 1.85 - 2.31 ) | -29.93 | -3.96 ( -4.47 - -3.44 ) |
| Ireland | Male | 94.1 ( 84.75 - 104.27 ) | 4.98 ( 4.5 - 5.51 ) | 4118.63 ( 3630.8 - 4698.65 ) | 4.77 ( 4.1 - 5.5 ) | 4276.86 | -0.02 ( -0.16 - 0.12 ) |
| Israel | Male | 60.36 ( 54.57 - 67.19 ) | 2.72 ( 2.46 - 3.02 ) | 150.88 ( 113.97 - 199.02 ) | 3.09 ( 2.69 - 3.54 ) | 149.96 | -0.25 ( -0.67 - 0.17 ) |
| Italy | Male | 5065.4 ( 4688.39 - 5523.86 ) | 12.85 ( 11.91 - 13.92 ) | 62.89 ( 47.65 - 78.49 ) | 7.16 ( 6.32 - 8.19 ) | -98.76 | -2.16 ( -2.22 - -2.11 ) |
| Ivory Coast | Male | 68.63 ( 55.54 - 83.6 ) | 2.79 ( 2.28 - 3.35 ) | 4435.6 ( 4146.29 - 4728.26 ) | 2.62 ( 2.01 - 3.45 ) | 6363.07 | -0.38 ( -0.62 - -0.14 ) |
| Jamaica | Male | 22.3 ( 20.03 - 24.76 ) | 2.68 ( 2.41 - 2.96 ) | 50.78 ( 42.73 - 62.07 ) | 4.54 ( 3.44 - 5.65 ) | 127.72 | 1.22 ( 0.62 - 1.83 ) |
| Japan | Male | 3060.5 ( 2909.53 - 3217.69 ) | 4.01 ( 3.82 - 4.21 ) | 313.55 ( 281.17 - 354.95 ) | 2.96 ( 2.77 - 3.15 ) | -89.75 | -1.25 ( -1.34 - -1.16 ) |
| Jordan | Male | 29.71 ( 23.35 - 36.66 ) | 3.82 ( 3.05 - 4.72 ) | 372.73 ( 255.86 - 437.49 ) | 1.63 ( 1.36 - 1.99 ) | 1154.5 | -3.93 ( -4.38 - -3.48 ) |
| Kazakhstan | Male | 459.9 ( 430.07 - 492.7 ) | 8.09 ( 7.63 - 8.61 ) | 0.41 ( 0.33 - 0.5 ) | 4.16 ( 3.76 - 4.66 ) | -99.91 | -3.05 ( -3.3 - -2.8 ) |
| Kenya | Male | 134.71 ( 76.81 - 168.89 ) | 3.06 ( 1.73 - 3.83 ) | 20.22 ( 17.66 - 23.37 ) | 3.41 ( 2.33 - 3.99 ) | -84.99 | 0.46 ( 0.19 - 0.73 ) |
| Kiribati | Male | 0.21 ( 0.18 - 0.24 ) | 1.37 ( 1.18 - 1.56 ) | 36.57 ( 32.22 - 42.06 ) | 1.59 ( 1.3 - 1.89 ) | 17403.31 | 0.4 ( 0.1 - 0.7 ) |
| Kuwait | Male | 9.4 ( 8.41 - 10.47 ) | 2.25 ( 2.02 - 2.49 ) | 64.75 ( 45.48 - 111.24 ) | 1.41 ( 1.23 - 1.61 ) | 588.53 | -1.27 ( -1.81 - -0.73 ) |
| Kyrgyzstan | Male | 68.93 ( 62 - 76.31 ) | 5.13 ( 4.63 - 5.67 ) | 142.22 ( 118.86 - 171.05 ) | 1.81 ( 1.62 - 2.05 ) | 106.32 | -3.84 ( -4.24 - -3.44 ) |
| Laos | Male | 46.14 ( 34.96 - 59.98 ) | 4.4 ( 3.36 - 5.75 ) | 236.35 ( 199.4 - 277.21 ) | 3.21 ( 2.29 - 5.54 ) | 412.25 | -1.24 ( -1.32 - -1.16 ) |
| Latvia | Male | 141.65 ( 131.11 - 152.58 ) | 9.75 ( 9.08 - 10.47 ) | 29.54 ( 22.68 - 37.58 ) | 9.92 ( 8.28 - 11.94 ) | -79.15 | 0.1 ( -0.29 - 0.5 ) |
| Lebanon | Male | 102.04 ( 80.89 - 131.82 ) | 9.52 ( 7.59 - 12.33 ) | 20.48 ( 14.45 - 27.18 ) | 8.46 ( 7.2 - 9.89 ) | -79.93 | -0.33 ( -0.68 - 0.03 ) |
| Lesotho | Male | 25.25 ( 17.21 - 31.75 ) | 5.46 ( 3.69 - 6.8 ) | 193.41 ( 127.5 - 247.15 ) | 5.9 ( 4.61 - 7.42 ) | 665.89 | 0.61 ( 0.32 - 0.9 ) |
| Liberia | Male | 13.56 ( 10.62 - 17.41 ) | 2.17 ( 1.7 - 2.75 ) | 256.83 ( 225.58 - 292.29 ) | 2.15 ( 1.56 - 2.85 ) | 1794 | 0.13 ( -0.02 - 0.28 ) |
| Libya | Male | 71 ( 55.95 - 91.24 ) | 6.63 ( 5.22 - 8.41 ) | 28 ( 23.43 - 33.22 ) | 7.73 ( 5.15 - 9.88 ) | -60.57 | 0.72 ( 0.61 - 0.83 ) |
| Lithuania | Male | 208.34 ( 195.26 - 224.72 ) | 10.87 ( 10.18 - 11.68 ) | 142.5 ( 122.74 - 168.19 ) | 12.28 ( 10.83 - 13.97 ) | -31.6 | 0.34 ( -0.05 - 0.74 ) |
| Luxembourg | Male | 25.98 ( 23 - 29.3 ) | 10.82 ( 9.6 - 12.19 ) | 127.11 ( 93.91 - 165.48 ) | 6.31 ( 5.28 - 7.49 ) | 389.35 | -2.35 ( -2.49 - -2.21 ) |
| Macedonia | Male | 69.02 ( 61.51 - 78.71 ) | 7.18 ( 6.43 - 8.17 ) | 44.59 ( 36.17 - 54.9 ) | 8.78 ( 7.6 - 10.34 ) | -35.39 | 0.62 ( 0.39 - 0.84 ) |
| Madagascar | Male | 83.82 ( 68.25 - 102.52 ) | 2.95 ( 2.41 - 3.59 ) | 474.57 ( 358.25 - 584.73 ) | 2.25 ( 1.7 - 2.84 ) | 466.15 | -1.07 ( -1.17 - -0.98 ) |
| Malawi | Male | 26.13 ( 14.37 - 36.31 ) | 1.29 ( 0.75 - 1.78 ) | 3.87 ( 3.31 - 4.52 ) | 1.32 ( 1.08 - 1.62 ) | -85.18 | -0.22 ( -0.53 - 0.09 ) |
| Malaysia | Male | 166.26 ( 144.5 - 193.82 ) | 3.77 ( 3.27 - 4.34 ) | 60.61 ( 46.2 - 78.15 ) | 3.7 ( 2.83 - 4.53 ) | -63.55 | -0.53 ( -0.89 - -0.17 ) |
| Maldives | Male | 1.81 ( 1.27 - 2.17 ) | 3.39 ( 2.54 - 4.02 ) | 20.83 ( 17.97 - 24.03 ) | 2.61 ( 2.21 - 3.05 ) | 1050.64 | -1.35 ( -1.51 - -1.19 ) |
| Mali | Male | 39.76 ( 32.57 - 47.54 ) | 1.86 ( 1.53 - 2.22 ) | 0.61 ( 0.45 - 0.78 ) | 1.4 ( 1.08 - 1.79 ) | -98.46 | -0.87 ( -1.12 - -0.62 ) |
| Malta | Male | 13.59 ( 12.19 - 15.14 ) | 6.92 ( 6.22 - 7.71 ) | 20.26 ( 14.26 - 28.75 ) | 5.24 ( 4.54 - 6 ) | 49.02 | -1.48 ( -1.68 - -1.28 ) |
| Marshall Islands | Male | 0.3 ( 0.18 - 0.37 ) | 3.6 ( 2.26 - 4.41 ) | 30.61 ( 26.53 - 35.13 ) | 3.93 ( 3.02 - 4.84 ) | 10239.64 | 0.25 ( 0.15 - 0.36 ) |
| Mauritania | Male | 11.52 ( 9.15 - 14.21 ) | 2.34 ( 1.88 - 2.88 ) | 1273.69 ( 1159.94 - 1325.22 ) | 2.08 ( 1.49 - 2.94 ) | 10958.97 | -0.15 ( -0.4 - 0.11 ) |
| Mauritius | Male | 18.63 ( 16.86 - 20.46 ) | 5.39 ( 4.9 - 5.89 ) | 0.92 ( 0.7 - 1.16 ) | 3.86 ( 3.37 - 4.37 ) | -95.08 | -1.42 ( -1.62 - -1.21 ) |
| Mexico | Male | 720.52 ( 700.65 - 752.23 ) | 3.52 ( 3.43 - 3.67 ) | 227.68 ( 205.95 - 252.6 ) | 2.43 ( 2.21 - 2.52 ) | -68.4 | -1.83 ( -2.01 - -1.65 ) |
| Micronesia | Male | 0.71 ( 0.55 - 0.97 ) | 3.05 ( 2.37 - 4.27 ) | 21.49 ( 18 - 26.28 ) | 2.99 ( 2.42 - 3.76 ) | 2930.21 | -0.03 ( -0.05 - 0 ) |
| Moldova | Male | 174.72 ( 163.23 - 187.24 ) | 8.46 ( 7.92 - 9.04 ) | 53.33 ( 44.48 - 63.7 ) | 9.08 ( 8.24 - 10.04 ) | -69.47 | 0.43 ( 0.03 - 0.82 ) |
| Mongolia | Male | 6.84 ( 5.99 - 7.71 ) | 1.64 ( 1.45 - 1.85 ) | 907.11 ( 691.51 - 1182.82 ) | 3.06 ( 2.61 - 3.64 ) | 13168.16 | 3.2 ( 2.61 - 3.78 ) |
| Montenegro | Male | 33.39 ( 28.53 - 40.3 ) | 10.91 ( 9.4 - 13.12 ) | 200.38 ( 120.99 - 265.9 ) | 11.41 ( 9.56 - 13.6 ) | 500.13 | 0.09 ( -0.03 - 0.21 ) |
| Morocco | Male | 406.39 ( 340.26 - 491 ) | 5.45 ( 4.57 - 6.62 ) | 713.56 ( 565.15 - 1044.53 ) | 5.48 ( 4.19 - 7.09 ) | 75.58 | -0.05 ( -0.16 - 0.05 ) |
| Mozambique | Male | 125.31 ( 87.47 - 162.82 ) | 3.91 ( 2.77 - 4.98 ) | 33.45 ( 27.55 - 40.8 ) | 3.68 ( 2.36 - 4.73 ) | -73.3 | -0.07 ( -0.18 - 0.03 ) |
| Myanmar | Male | 505.95 ( 379.78 - 656.36 ) | 4.41 ( 3.35 - 5.64 ) | 521.26 ( 392.53 - 700.53 ) | 3.61 ( 2.89 - 5.34 ) | 3.03 | -0.68 ( -0.75 - -0.61 ) |
| Namibia | Male | 21.39 ( 16.97 - 26.38 ) | 6.07 ( 4.9 - 7.4 ) | 656.34 ( 576.92 - 750.29 ) | 5.4 ( 4.53 - 6.5 ) | 2968.35 | -0.65 ( -1.01 - -0.29 ) |
| Nepal | Male | 335.18 ( 255.16 - 435.7 ) | 6.3 ( 4.81 - 8.11 ) | 96.13 ( 83.21 - 110.93 ) | 4.91 ( 3.74 - 6.52 ) | -71.32 | -0.91 ( -1.3 - -0.52 ) |
| Netherlands | Male | 553.49 ( 506.49 - 602.05 ) | 6.34 ( 5.83 - 6.89 ) | 35.74 ( 29.74 - 42.74 ) | 4.3 ( 3.79 - 4.9 ) | -93.54 | -1.8 ( -1.96 - -1.64 ) |
| New Zealand | Male | 83.43 ( 72.75 - 95.11 ) | 4.6 ( 4.04 - 5.23 ) | 75.56 ( 40.09 - 106.88 ) | 2.8 ( 2.43 - 3.22 ) | -9.43 | -2.31 ( -2.48 - -2.14 ) |
| Nicaragua | Male | 17.67 ( 15.41 - 20.22 ) | 2.37 ( 2.07 - 2.71 ) | 990.04 ( 707.96 - 1408.94 ) | 1.73 ( 1.44 - 2.06 ) | 5503.35 | -1.32 ( -1.48 - -1.17 ) |
| Niger | Male | 31.62 ( 19.79 - 42.58 ) | 2.08 ( 1.33 - 2.74 ) | 426 ( 324.92 - 546.75 ) | 2.13 ( 1.16 - 2.96 ) | 1247.07 | 0.27 ( 0.1 - 0.43 ) |
| Nigeria | Male | 723.56 ( 522.43 - 978.81 ) | 2.87 ( 2.12 - 3.81 ) | 1.42 ( 1.18 - 1.72 ) | 2.4 ( 1.75 - 3.39 ) | -99.8 | -0.82 ( -0.92 - -0.71 ) |
| North Korea | Male | 218.81 ( 157.84 - 285.36 ) | 3.19 ( 2.39 - 4.03 ) | 115.02 ( 102.61 - 128.22 ) | 3.16 ( 2.47 - 3.96 ) | -47.43 | 0.01 ( -0.03 - 0.04 ) |
| Northern Mariana Islands | Male | 0.56 ( 0.43 - 0.79 ) | 5.55 ( 4.41 - 7.7 ) | 22.47 ( 16.55 - 29.37 ) | 5.79 ( 4.88 - 6.87 ) | 3943.32 | 0.17 ( 0 - 0.35 ) |
| Norway | Male | 116.91 ( 106.39 - 128.44 ) | 4.02 ( 3.67 - 4.4 ) | 5294.56 ( 3983.51 - 6837.79 ) | 2.67 ( 2.39 - 2.97 ) | 4428.77 | -1.78 ( -1.92 - -1.64 ) |
| Oman | Male | 9.11 ( 6.77 - 11.91 ) | 2.29 ( 1.73 - 2.94 ) | 18.85 ( 16.15 - 22.23 ) | 1.77 ( 1.33 - 2.24 ) | 106.84 | -0.9 ( -1.02 - -0.78 ) |
| Pakistan | Male | 2624.87 ( 2219.32 - 3055.36 ) | 8.17 ( 6.92 - 9.49 ) | 55.18 ( 48.5 - 62.64 ) | 8.57 ( 6.54 - 10.83 ) | -97.9 | -0.01 ( -0.21 - 0.19 ) |
| Palestine | Male | 10.33 ( 7.25 - 13.19 ) | 2.55 ( 1.79 - 3.24 ) | 78.99 ( 61.05 - 102.34 ) | 1.6 ( 1.37 - 1.9 ) | 664.46 | -2 ( -2.21 - -1.78 ) |
| Panama | Male | 34.93 ( 32.15 - 38.01 ) | 4.54 ( 4.19 - 4.95 ) | 89.34 ( 69.69 - 113.06 ) | 2.86 ( 2.52 - 3.25 ) | 155.78 | -2.24 ( -2.55 - -1.93 ) |
| Papua New Guinea | Male | 31.92 ( 24.76 - 40.44 ) | 3.14 ( 2.51 - 3.85 ) | 166.81 ( 134.85 - 202.48 ) | 3.3 ( 2.63 - 4.15 ) | 422.62 | 0.32 ( 0.24 - 0.39 ) |
| Paraguay | Male | 31.52 ( 26.84 - 37.82 ) | 2.91 ( 2.49 - 3.44 ) | 803.28 ( 654.3 - 964.58 ) | 3.42 ( 2.68 - 4.32 ) | 2448.63 | 0.72 ( 0.57 - 0.86 ) |
| Peru | Male | 131.84 ( 114.85 - 156.76 ) | 2.25 ( 1.95 - 2.68 ) | 2414.76 ( 2156.33 - 2703.24 ) | 1.13 ( 0.91 - 1.37 ) | 1731.63 | -3.16 ( -3.48 - -2.85 ) |
| Philippines | Male | 317.88 ( 289.89 - 349.07 ) | 2.01 ( 1.84 - 2.21 ) | 525.57 ( 467.32 - 593.68 ) | 2.37 ( 1.95 - 2.84 ) | 65.34 | 0.7 ( 0.63 - 0.78 ) |
| Poland | Male | 2077.55 ( 1975.57 - 2178.71 ) | 10.45 ( 9.96 - 10.96 ) | 124.19 ( 108.79 - 141.36 ) | 8.06 ( 7.23 - 9.02 ) | -94.02 | -1.35 ( -1.58 - -1.11 ) |
| Portugal | Male | 486.75 ( 460.38 - 513.8 ) | 7.86 ( 7.44 - 8.3 ) | 16.51 ( 12.6 - 20.87 ) | 5.76 ( 5.11 - 6.52 ) | -96.61 | -1.36 ( -1.57 - -1.14 ) |
| Puerto Rico | Male | 127.35 ( 117.86 - 138.23 ) | 7.44 ( 6.87 - 8.08 ) | 38.86 ( 29.32 - 56.3 ) | 4.23 ( 3.71 - 4.81 ) | -69.49 | -2.6 ( -2.8 - -2.4 ) |
| Qatar | Male | 1.55 ( 1.23 - 1.9 ) | 2.57 ( 2.08 - 3.13 ) | 1563.67 ( 1407.18 - 1743.25 ) | 2.81 ( 2.17 - 3.51 ) | 100880.8 | 1.06 ( 0.2 - 1.93 ) |
| Republic of Congo | Male | 25.01 ( 19.27 - 30.47 ) | 4.94 ( 3.96 - 5.94 ) | 7156.27 ( 6844.2 - 7480.14 ) | 3.25 ( 2.49 - 4.87 ) | 28517.8 | -1.84 ( -2.02 - -1.66 ) |
| Romania | Male | 1001.55 ( 949.55 - 1062.98 ) | 7.31 ( 6.94 - 7.74 ) | 68.8 ( 53.31 - 89.37 ) | 10.23 ( 9.2 - 11.39 ) | -93.13 | 1.1 ( 0.88 - 1.33 ) |
| Russia | Male | 7727.51 ( 7344.33 - 8320.21 ) | 10.44 ( 9.95 - 11.17 ) | 5.53 ( 4.86 - 6.22 ) | 7.58 ( 7.26 - 7.9 ) | -99.93 | -1.78 ( -2.12 - -1.43 ) |
| Rwanda | Male | 72.93 ( 52.22 - 91.95 ) | 5.14 ( 3.82 - 6.42 ) | 4.58 ( 4.1 - 5.15 ) | 2.69 ( 2.1 - 3.41 ) | -93.72 | -3.16 ( -3.5 - -2.82 ) |
| Saint Lucia | Male | 2.3 ( 2.08 - 2.54 ) | 5.74 ( 5.19 - 6.3 ) | 4.58 ( 4.1 - 5.15 ) | 5.41 ( 4.77 - 6.08 ) | 98.98 | -0.37 ( -0.55 - -0.19 ) |
| Saint Vincent | Male | 1.75 ( 1.56 - 1.92 ) | 5.32 ( 4.78 - 5.84 ) | 0.76 ( 0.59 - 0.9 ) | 6.5 ( 5.83 - 7.3 ) | -56.3 | 0.68 ( 0.51 - 0.84 ) |
| Samoa | Male | 0.55 ( 0.45 - 0.68 ) | 1.4 ( 1.15 - 1.69 ) | 0.89 ( 0.69 - 1.11 ) | 1.27 ( 0.93 - 1.5 ) | 61.95 | -0.42 ( -0.49 - -0.35 ) |
| Sao Tome and Principe | Male | 0.48 ( 0.4 - 0.57 ) | 1.57 ( 1.34 - 1.86 ) | 223.06 ( 176.27 - 282.36 ) | 2.01 ( 1.58 - 2.47 ) | 46745.42 | 0.99 ( 0.93 - 1.04 ) |
| Saudi Arabia | Male | 76.33 ( 51.04 - 101.41 ) | 2.06 ( 1.38 - 2.71 ) | 95.47 ( 74.05 - 123.27 ) | 2.13 ( 1.76 - 2.62 ) | 25.08 | 0.38 ( -0.08 - 0.84 ) |
| Senegal | Male | 40.99 ( 33.19 - 49.93 ) | 2.48 ( 2.01 - 3 ) | 760.74 ( 661.98 - 873.42 ) | 2.81 ( 2.18 - 3.55 ) | 1756.04 | 0.82 ( 0.58 - 1.06 ) |
| Serbia | Male | 665.92 ( 570.92 - 802.23 ) | 11.26 ( 9.8 - 13.46 ) | 8.21 ( 7.09 - 9.4 ) | 10.66 ( 9.21 - 12.25 ) | -98.77 | 0.02 ( -0.15 - 0.2 ) |
| Seychelles | Male | 3.87 ( 3.31 - 4.39 ) | 15.23 ( 13.08 - 17.29 ) | 43.75 ( 34.56 - 55.31 ) | 15.65 ( 13.61 - 17.89 ) | 1030.88 | -0.33 ( -0.48 - -0.17 ) |
| Sierra Leone | Male | 23.47 ( 16.48 - 29.84 ) | 2.33 ( 1.65 - 2.93 ) | 100.8 ( 86.45 - 116.67 ) | 2.6 ( 2.07 - 3.23 ) | 329.4 | 0.73 ( 0.55 - 0.91 ) |
| Singapore | Male | 58.49 ( 52.14 - 65.3 ) | 5.32 ( 4.78 - 5.92 ) | 283 ( 243.81 - 331.13 ) | 3.14 ( 2.72 - 3.63 ) | 383.84 | -2.04 ( -2.27 - -1.81 ) |
| Slovakia | Male | 324.69 ( 299.56 - 347.3 ) | 12.19 ( 11.26 - 13.05 ) | 95.58 ( 81.68 - 110.15 ) | 6.95 ( 6.03 - 8.1 ) | -70.56 | -2.09 ( -2.16 - -2.01 ) |
| Slovenia | Male | 100.09 ( 92.4 - 108.94 ) | 9.27 ( 8.59 - 10.03 ) | 4.26 ( 3.38 - 5.3 ) | 5.28 ( 4.55 - 6.06 ) | -95.74 | -2.27 ( -2.4 - -2.15 ) |
| Solomon Islands | Male | 2.15 ( 1.65 - 2.68 ) | 2.72 ( 2.13 - 3.33 ) | 95.4 ( 69.84 - 127.4 ) | 2.67 ( 2.1 - 3.39 ) | 4340.24 | 0.05 ( -0.01 - 0.11 ) |
| Somalia | Male | 51.05 ( 27.35 - 78.85 ) | 3.56 ( 2.1 - 5.17 ) | 675.73 ( 629.56 - 737.14 ) | 2.91 ( 2.17 - 3.82 ) | 1223.54 | -1.25 ( -1.48 - -1.02 ) |
| South Africa | Male | 418.17 ( 359.87 - 553.75 ) | 4.37 ( 3.76 - 5.86 ) | 1610.85 ( 1380.74 - 1857.39 ) | 3.58 ( 3.35 - 3.88 ) | 285.22 | -1.18 ( -1.78 - -0.57 ) |
| South Korea | Male | 1017.21 ( 934.84 - 1108.51 ) | 7.54 ( 7 - 8.15 ) | 58.29 ( 41.98 - 81.12 ) | 4.22 ( 3.64 - 4.85 ) | -94.27 | -3.66 ( -4.34 - -2.98 ) |
| South Sudan | Male | 47.59 ( 26.14 - 71.97 ) | 3.28 ( 1.88 - 4.82 ) | 3625.32 ( 3205.22 - 4081.59 ) | 2.67 ( 1.97 - 3.69 ) | 7517.12 | -1.16 ( -1.34 - -0.97 ) |
| Spain | Male | 4037.98 ( 3806.83 - 4281.05 ) | 16.52 ( 15.55 - 17.5 ) | 319 ( 250.51 - 394.47 ) | 9.35 ( 8.25 - 10.54 ) | -92.1 | -2.55 ( -2.71 - -2.38 ) |
| Sri Lanka | Male | 103.05 ( 92.05 - 116.62 ) | 1.82 ( 1.63 - 2.05 ) | 309.16 ( 222.1 - 424.3 ) | 2.76 ( 2.19 - 3.4 ) | 200 | 2.46 ( 2.14 - 2.77 ) |
| Sudan | Male | 175.42 ( 124.26 - 236.97 ) | 3.51 ( 2.53 - 4.72 ) | 6.19 ( 5.21 - 7.31 ) | 3.19 ( 2.31 - 4.43 ) | -96.47 | -0.31 ( -0.33 - -0.28 ) |
| Suriname | Male | 2.18 ( 1.94 - 2.45 ) | 1.73 ( 1.54 - 1.93 ) | 12.66 ( 9.01 - 16.09 ) | 2.24 ( 1.9 - 2.63 ) | 480.21 | 1 ( 0.8 - 1.19 ) |
| Swaziland | Male | 8.13 ( 6.31 - 10.09 ) | 5.8 ( 4.54 - 7.15 ) | 208.52 ( 183.05 - 234.32 ) | 5.14 ( 3.65 - 6.4 ) | 2466.2 | -0.14 ( -0.59 - 0.31 ) |
| Sweden | Male | 208.31 ( 190.63 - 227.88 ) | 3.2 ( 2.93 - 3.52 ) | 237.95 ( 202.52 - 276.46 ) | 2.25 ( 1.98 - 2.53 ) | 14.23 | -1.52 ( -1.62 - -1.42 ) |
| Switzerland | Male | 302.68 ( 272.28 - 335.32 ) | 6.85 ( 6.16 - 7.6 ) | 119.42 ( 93.39 - 152.55 ) | 3.21 ( 2.72 - 3.73 ) | -60.55 | -2.62 ( -2.77 - -2.47 ) |
| Syria | Male | 51.08 ( 43.24 - 60.23 ) | 1.78 ( 1.52 - 2.09 ) | 947.51 ( 823.71 - 1089.19 ) | 1.78 ( 1.4 - 2.25 ) | 1754.79 | -0.37 ( -0.73 - 0 ) |
| Tajikistan | Male | 30.14 ( 27.06 - 33.75 ) | 2.21 ( 2 - 2.45 ) | 290.71 ( 219.77 - 428.89 ) | 1.1 ( 0.92 - 1.29 ) | 864.58 | -2.78 ( -3.12 - -2.44 ) |
| Tanzania | Male | 180.91 ( 121.4 - 258.22 ) | 3.13 ( 2.14 - 4.4 ) | 2149.12 ( 1799.11 - 2570.54 ) | 2.4 ( 1.84 - 3.49 ) | 1087.95 | -1.31 ( -1.5 - -1.13 ) |
| Thailand | Male | 919.19 ( 808.52 - 1027.17 ) | 5.31 ( 4.69 - 5.93 ) | 5.97 ( 4.61 - 7.92 ) | 4.65 ( 3.9 - 5.54 ) | -99.35 | -0.93 ( -1.21 - -0.66 ) |
| Timor-Leste | Male | 3.94 ( 2.96 - 5.92 ) | 2.62 ( 2 - 3.87 ) | 11.92 ( 8.41 - 19.95 ) | 3 ( 2.14 - 4.96 ) | 202.51 | 0.7 ( 0.46 - 0.95 ) |
| Tobago | Male | 14.06 ( 12.86 - 15.32 ) | 3.46 ( 3.16 - 3.75 ) | 24.94 ( 19.22 - 31.79 ) | 2.91 ( 2.25 - 3.68 ) | 77.4 | -0.82 ( -1.09 - -0.55 ) |
| Togo | Male | 14.15 ( 11.3 - 16.72 ) | 2.35 ( 1.89 - 2.75 ) | 41.84 ( 31.13 - 53.7 ) | 2.8 ( 2.14 - 3.51 ) | 195.74 | 0.99 ( 0.83 - 1.15 ) |
| Tonga | Male | 0.54 ( 0.45 - 0.73 ) | 2.1 ( 1.75 - 2.81 ) | 0.88 ( 0.7 - 1.16 ) | 2.43 ( 1.94 - 3.17 ) | 62.14 | 0.66 ( 0.61 - 0.72 ) |
| Trinidad | Male | 14.06 ( 12.86 - 15.32 ) | 3.46 ( 3.16 - 3.75 ) | 24.94 ( 19.22 - 31.79 ) | 2.91 ( 2.25 - 3.68 ) | 77.4 | -0.82 ( -1.09 - -0.55 ) |
| Tunisia | Male | 162.91 ( 138.44 - 200.39 ) | 6 ( 5.13 - 7.34 ) | 389.81 ( 287.48 - 504.18 ) | 6.4 ( 4.77 - 8.24 ) | 139.28 | -0.1 ( -0.26 - 0.07 ) |
| Turkey | Male | 1289.25 ( 1103.11 - 1553.02 ) | 7.24 ( 6.21 - 8.81 ) | 2208.74 ( 1879.31 - 2574.32 ) | 5.46 ( 4.66 - 6.36 ) | 71.32 | -1.22 ( -1.41 - -1.02 ) |
| Turkmenistan | Male | 45.04 ( 41.55 - 48.79 ) | 4.95 ( 4.58 - 5.35 ) | 33.93 ( 28.65 - 38.78 ) | 1.85 ( 1.58 - 2.1 ) | -24.67 | -4.39 ( -4.78 - -3.99 ) |
| Uganda | Male | 78.07 ( 58.52 - 101.64 ) | 2.26 ( 1.71 - 2.94 ) | 155.56 ( 119.55 - 216.57 ) | 2.42 ( 1.87 - 3.36 ) | 99.27 | -0.25 ( -0.65 - 0.16 ) |
| UK | Male | 1985.95 ( 1917.21 - 2056.42 ) | 5.1 ( 4.93 - 5.29 ) | 2481.38 ( 2391.03 - 2583.53 ) | 4.52 ( 4.35 - 4.7 ) | 24.95 | -0.84 ( -0.98 - -0.71 ) |
| Ukraine | Male | 3469.91 ( 3248.19 - 3703.09 ) | 11.84 ( 11.1 - 12.59 ) | 3056.61 ( 2759.98 - 3359.92 ) | 10.2 ( 9.23 - 11.2 ) | -11.91 | -1.38 ( -1.81 - -0.96 ) |
| United Arab Emirates | Male | 16.51 ( 11.68 - 24.52 ) | 4.47 ( 3.34 - 5.79 ) | 211.49 ( 136.63 - 295.45 ) | 5.67 ( 3.28 - 7.92 ) | 1181.13 | 1.06 ( 0.95 - 1.17 ) |
| Uruguay | Male | 216.35 ( 201.45 - 232.76 ) | 12.22 ( 11.4 - 13.1 ) | 173.49 ( 146.65 - 204.82 ) | 7.88 ( 6.65 - 9.3 ) | -19.81 | -1.73 ( -1.88 - -1.58 ) |
| USA | Male | 9645.92 ( 9422.3 - 9935.09 ) | 7.02 ( 6.86 - 7.23 ) | 14420.33 ( 13796.43 - 15004.25 ) | 5.79 ( 5.54 - 6.02 ) | 49.5 | -1.31 ( -1.53 - -1.09 ) |
| Uzbekistan | Male | 196.51 ( 184.6 - 209.16 ) | 3.63 ( 3.42 - 3.84 ) | 300.34 ( 255.01 - 355.57 ) | 2.93 ( 2.51 - 3.43 ) | 52.84 | -0.42 ( -0.79 - -0.06 ) |
| Vanuatu | Male | 1.18 ( 0.86 - 1.96 ) | 3.25 ( 2.39 - 5.39 ) | 3.03 ( 2.05 - 6.24 ) | 3.6 ( 2.48 - 7.31 ) | 156.2 | 0.48 ( 0.43 - 0.53 ) |
| Venezuela | Male | 229.66 ( 214.55 - 246.18 ) | 4.95 ( 4.63 - 5.29 ) | 698.76 ( 564.64 - 858.7 ) | 5.25 ( 4.27 - 6.39 ) | 204.25 | 0 ( -0.13 - 0.12 ) |
| Vietnam | Male | 646.07 ( 530.38 - 767.18 ) | 3.63 ( 2.98 - 4.28 ) | 2124.21 ( 1692.72 - 2703.97 ) | 4.98 ( 4.02 - 6.22 ) | 228.79 | 1.23 ( 1.17 - 1.29 ) |
| Virgin Islands | Male | 2.27 ( 2 - 2.58 ) | 5.52 ( 4.89 - 6.21 ) | 6.81 ( 5.21 - 8.23 ) | 7.85 ( 6.1 - 9.46 ) | 199.74 | 1.7 ( 1.54 - 1.86 ) |
| Yemen | Male | 105.45 ( 57.27 - 147.62 ) | 4 ( 2.27 - 5.59 ) | 254.94 ( 187.19 - 330.37 ) | 4.01 ( 2.97 - 5.16 ) | 141.76 | 0.03 ( -0.01 - 0.07 ) |
| Zambia | Male | 65.59 ( 49.36 - 91.99 ) | 4.05 ( 3.14 - 5.65 ) | 97.64 ( 74.52 - 154.16 ) | 2.96 ( 2.27 - 4.7 ) | 48.86 | -1.76 ( -2.06 - -1.47 ) |
| Zimbabwe | Male | 95.31 ( 79.56 - 112.89 ) | 4.38 ( 3.67 - 5.14 ) | 151.26 ( 116.31 - 188.25 ) | 4.77 ( 3.74 - 5.91 ) | 58.71 | 0.68 ( 0.04 - 1.32 ) |

ASDR, age standardized death rate; CI, confidence interval; EAPC, estimated annual percentage change; UI, uncertainty interval.
