# Supplementary material for: Global burden of larynx cancer, 1990-2017: estimates from the global burden of disease 2017 study
Source: Aging (Albany NY). 2020 Feb 8;12(3):2545–83. doi: 10.18632/aging.102762 (PMC7041735; doi:10.18632/aging.102762)
Supplement: Supplementary Table 4 [file aging-12-102762-s005..docx]

**Supplementary Table** **4.**  **The deaths of larynx cancer in 1990 and 2017 among 195vcountries and territories, and its temporal trends from 1990 to 2017.**

| **Location** | **Sex** | **1990** | | **2017** | | **1990-2017** | |
| --- | --- | --- | --- | --- | --- | --- | --- |
|  |  | **Deaths No. (95% UI)** | **ASDR per 100,000 No. (95% UI)** | **Deaths No. (95% UI)** | **ASDR per 100,000 No. (95% UI)** | **Change in**  **absolute number of deaths**  **No. (%)** | **EAPC No. (95% CI)** |
| Afghanistan | Both | 290.1 ( 163.16 - 394.79 ) | 4.01 ( 2.3 - 5.41 ) | 397.89 ( 315.63 - 499.46 ) | 3.66 ( 2.93 - 4.65 ) | 37.16 | -0.39 ( -0.62 - -0.16 ) |
| Albania | Both | 67.15 ( 62.21 - 72.72 ) | 3.09 ( 2.86 - 3.32 ) | 88.14 ( 69.39 - 111.02 ) | 2.08 ( 1.64 - 2.62 ) | 31.26 | -1.38 ( -1.63 - -1.12 ) |
| Algeria | Both | 260.74 ( 226.95 - 297.2 ) | 2.05 ( 1.79 - 2.32 ) | 436.13 ( 377.21 - 493.74 ) | 1.33 ( 1.16 - 1.51 ) | 67.26 | -1.53 ( -1.6 - -1.45 ) |
| American Samoa | Both | 0.32 ( 0.27 - 0.4 ) | 1.51 ( 1.28 - 1.95 ) | 0.46 ( 0.39 - 0.53 ) | 1.19 ( 1 - 1.39 ) | 44.2 | -0.74 ( -1.02 - -0.47 ) |
| Andorra | Both | 0.63 ( 0.47 - 0.86 ) | 1.07 ( 0.81 - 1.47 ) | 0.9 ( 0.73 - 1.13 ) | 0.66 ( 0.54 - 0.83 ) | 43.14 | -1.95 ( -2.03 - -1.88 ) |
| Angola | Both | 95.71 ( 64.78 - 124.68 ) | 2.4 ( 1.71 - 3.06 ) | 175.71 ( 141.78 - 211.73 ) | 1.68 ( 1.36 - 2.02 ) | 83.59 | -1.47 ( -1.57 - -1.38 ) |
| Antigua | Both | 0.93 ( 0.84 - 1.03 ) | 1.79 ( 1.63 - 1.98 ) | 1.57 ( 1.39 - 1.78 ) | 1.56 ( 1.39 - 1.77 ) | 68.74 | -0.56 ( -0.69 - -0.43 ) |
| Argentina | Both | 941.54 ( 902.6 - 983.23 ) | 2.83 ( 2.72 - 2.96 ) | 918.71 ( 804.67 - 1048.38 ) | 1.73 ( 1.52 - 1.98 ) | -2.43 | -2.17 ( -2.41 - -1.93 ) |
| Armenia | Both | 113.03 ( 104.9 - 122.32 ) | 3.8 ( 3.54 - 4.11 ) | 102.13 ( 93.22 - 111.59 ) | 2.41 ( 2.2 - 2.63 ) | -9.64 | -2.16 ( -2.37 - -1.94 ) |
| Australia | Both | 247.52 ( 236.8 - 258.28 ) | 1.24 ( 1.18 - 1.29 ) | 255.13 ( 225.18 - 290.92 ) | 0.62 ( 0.55 - 0.72 ) | 3.08 | -2.95 ( -3.12 - -2.77 ) |
| Austria | Both | 189.98 ( 180.78 - 198.9 ) | 1.68 ( 1.6 - 1.76 ) | 136.79 ( 124.1 - 150.28 ) | 0.83 ( 0.75 - 0.91 ) | -28 | -2.95 ( -3.1 - -2.8 ) |
| Azerbaijan | Both | 133.71 ( 123.41 - 145.31 ) | 2.42 ( 2.24 - 2.63 ) | 211.24 ( 180.08 - 247.48 ) | 2.16 ( 1.84 - 2.5 ) | 57.98 | -1.41 ( -1.82 - -1.01 ) |
| Bahamas | Both | 4.48 ( 4.09 - 4.9 ) | 2.84 ( 2.59 - 3.1 ) | 9.86 ( 8.55 - 11.19 ) | 2.6 ( 2.28 - 2.94 ) | 119.86 | -0.23 ( -0.38 - -0.08 ) |
| Bahrain | Both | 3.58 ( 3.1 - 4.14 ) | 2.53 ( 2.17 - 2.91 ) | 5.36 ( 4.54 - 6.28 ) | 0.8 ( 0.67 - 0.94 ) | 49.48 | -5.73 ( -6.32 - -5.12 ) |
| Bangladesh | Both | 2530.49 ( 2172.31 - 2957.62 ) | 5.2 ( 4.44 - 6.1 ) | 2997.1 ( 2270.58 - 3604.8 ) | 2.42 ( 1.81 - 2.9 ) | 18.44 | -2.7 ( -2.82 - -2.59 ) |
| Barbados | Both | 4.64 ( 4.27 - 5.01 ) | 1.55 ( 1.44 - 1.67 ) | 7.23 ( 6.42 - 8.19 ) | 1.48 ( 1.31 - 1.68 ) | 55.86 | -0.26 ( -0.41 - -0.1 ) |
| Barbuda | Both | 0.93 ( 0.84 - 1.03 ) | 1.79 ( 1.63 - 1.98 ) | 1.57 ( 1.39 - 1.78 ) | 1.56 ( 1.39 - 1.77 ) | 68.74 | -0.56 ( -0.69 - -0.43 ) |
| Belarus | Both | 461.47 ( 440.38 - 483.23 ) | 3.43 ( 3.28 - 3.59 ) | 342.94 ( 305.92 - 385.73 ) | 2.16 ( 1.93 - 2.44 ) | -25.69 | -2.66 ( -3.21 - -2.11 ) |
| Belgium | Both | 381.23 ( 364.09 - 399.81 ) | 2.54 ( 2.43 - 2.66 ) | 217.96 ( 199.44 - 239.34 ) | 1.03 ( 0.94 - 1.13 ) | -42.83 | -3.62 ( -3.75 - -3.5 ) |
| Belize | Both | 1.35 ( 1.22 - 1.48 ) | 1.44 ( 1.3 - 1.59 ) | 4.5 ( 4.09 - 4.9 ) | 1.74 ( 1.59 - 1.89 ) | 234.24 | 0.49 ( 0.08 - 0.91 ) |
| Benin | Both | 26.55 ( 21.87 - 31.46 ) | 1.34 ( 1.11 - 1.57 ) | 57.57 ( 45.05 - 74.45 ) | 1.3 ( 1.03 - 1.66 ) | 116.84 | 0.19 ( 0.07 - 0.3 ) |
| Bermuda | Both | 1.76 ( 1.62 - 1.92 ) | 2.77 ( 2.55 - 3.01 ) | 2.44 ( 2.21 - 2.68 ) | 1.93 ( 1.75 - 2.11 ) | 38.12 | -1.11 ( -1.26 - -0.97 ) |
| Bhutan | Both | 11.28 ( 8.94 - 15.38 ) | 4.26 ( 3.37 - 5.75 ) | 14.6 ( 11.03 - 21.16 ) | 2.42 ( 1.84 - 3.51 ) | 29.35 | -2.19 ( -2.27 - -2.11 ) |
| Bolivia | Both | 68.7 ( 57.96 - 79.21 ) | 2.15 ( 1.82 - 2.47 ) | 110.31 ( 86.93 - 140.43 ) | 1.33 ( 1.05 - 1.68 ) | 60.58 | -1.9 ( -2 - -1.8 ) |
| Bosnia and Herzegovina | Both | 171.31 ( 159.57 - 183.46 ) | 3.96 ( 3.7 - 4.23 ) | 149.06 ( 132.12 - 166.36 ) | 2.42 ( 2.15 - 2.7 ) | -12.99 | -2.59 ( -2.95 - -2.23 ) |
| Botswana | Both | 13.17 ( 10.9 - 16.04 ) | 2.2 ( 1.84 - 2.65 ) | 17.47 ( 14.34 - 23.24 ) | 1.33 ( 1.1 - 1.73 ) | 32.67 | -1.85 ( -1.94 - -1.76 ) |
| Brazil | Both | 2438.17 ( 2383.64 - 2519.42 ) | 2.65 ( 2.59 - 2.74 ) | 5005.31 ( 4879.29 - 5148.35 ) | 2.17 ( 2.12 - 2.23 ) | 105.29 | -0.83 ( -0.9 - -0.76 ) |
| Brunei | Both | 2.4 ( 2.13 - 2.69 ) | 2.67 ( 2.36 - 2.98 ) | 2.28 ( 2.01 - 2.58 ) | 0.84 ( 0.73 - 0.95 ) | -5.19 | -5.13 ( -5.53 - -4.73 ) |
| Bulgaria | Both | 317.91 ( 302.53 - 333.28 ) | 2.45 ( 2.34 - 2.58 ) | 369.67 ( 333.73 - 407.98 ) | 2.74 ( 2.48 - 3.02 ) | 16.28 | 0.48 ( 0.25 - 0.72 ) |
| Burkina Faso | Both | 60.59 ( 46.21 - 77.13 ) | 1.41 ( 1.09 - 1.78 ) | 124.94 ( 90.36 - 153.71 ) | 1.49 ( 1.08 - 1.8 ) | 106.19 | 0.44 ( 0.32 - 0.55 ) |
| Burundi | Both | 71.16 ( 54.07 - 89 ) | 3.04 ( 2.37 - 3.76 ) | 76.8 ( 57.09 - 99.14 ) | 1.75 ( 1.32 - 2.21 ) | 7.92 | -2.51 ( -2.73 - -2.29 ) |
| Cambodia | Both | 121.18 ( 99.87 - 145.37 ) | 2.64 ( 2.19 - 3.18 ) | 208.3 ( 169.21 - 270.14 ) | 1.88 ( 1.54 - 2.51 ) | 71.89 | -1.27 ( -1.31 - -1.23 ) |
| Cameroon | Both | 67.73 ( 55.03 - 82.2 ) | 1.53 ( 1.26 - 1.84 ) | 180.71 ( 135.48 - 237.27 ) | 1.65 ( 1.26 - 2.19 ) | 166.8 | 0.56 ( 0.37 - 0.75 ) |
| Canada | Both | 452.19 ( 436.59 - 468.73 ) | 1.37 ( 1.32 - 1.42 ) | 480.91 ( 437.29 - 526.71 ) | 0.71 ( 0.65 - 0.78 ) | 6.35 | -2.75 ( -2.95 - -2.55 ) |
| Cape Verde | Both | 3.12 ( 2.62 - 3.63 ) | 1.34 ( 1.13 - 1.56 ) | 4.1 ( 3.6 - 4.62 ) | 0.93 ( 0.81 - 1.04 ) | 31.49 | -1.45 ( -1.56 - -1.34 ) |
| Central African Republic | Both | 31.78 ( 20.37 - 40.25 ) | 2.64 ( 1.78 - 3.29 ) | 46.6 ( 31.5 - 62.03 ) | 2.12 ( 1.55 - 2.76 ) | 46.6 | -0.95 ( -1.03 - -0.86 ) |
| Chad | Both | 32.27 ( 22.95 - 39.77 ) | 1.14 ( 0.81 - 1.4 ) | 81.37 ( 60.17 - 101.47 ) | 1.59 ( 1.18 - 1.96 ) | 152.14 | 1.62 ( 1.42 - 1.82 ) |
| Chile | Both | 136.08 ( 128.31 - 144.33 ) | 1.35 ( 1.28 - 1.44 ) | 169.56 ( 147.6 - 192.86 ) | 0.73 ( 0.63 - 0.83 ) | 24.6 | -2.29 ( -2.48 - -2.11 ) |
| China | Both | 11379.43 ( 10934.59 - 11998.94 ) | 1.34 ( 1.29 - 1.42 ) | 19460.63 ( 18610.15 - 20371.47 ) | 1 ( 0.96 - 1.05 ) | 71.02 | -1.12 ( -1.37 - -0.87 ) |
| Colombia | Both | 397.99 ( 383.2 - 416.8 ) | 2.33 ( 2.25 - 2.44 ) | 537.45 ( 472.46 - 612.08 ) | 1 ( 0.88 - 1.14 ) | 35.04 | -3.93 ( -4.21 - -3.65 ) |
| Comoros | Both | 4.73 ( 3.72 - 6.1 ) | 2.21 ( 1.75 - 2.8 ) | 6.26 ( 4.73 - 8.62 ) | 1.36 ( 1.04 - 1.89 ) | 32.44 | -2.08 ( -2.27 - -1.89 ) |
| Costa Rica | Both | 32.33 ( 30.4 - 34.42 ) | 1.86 ( 1.75 - 1.99 ) | 61.5 ( 54.09 - 68.15 ) | 1.27 ( 1.12 - 1.41 ) | 90.21 | -1.7 ( -1.96 - -1.44 ) |
| Croatia | Both | 273.57 ( 259.85 - 285.99 ) | 4.08 ( 3.88 - 4.26 ) | 185.48 ( 168.05 - 204.1 ) | 2.18 ( 1.98 - 2.4 ) | -32.2 | -2.53 ( -2.81 - -2.25 ) |
| Cuba | Both | 434.96 ( 416.62 - 452.69 ) | 4.16 ( 3.99 - 4.33 ) | 942.33 ( 817.77 - 1069.95 ) | 5.02 ( 4.35 - 5.7 ) | 116.64 | 0.67 ( 0.56 - 0.77 ) |
| Cyprus | Both | 11.98 ( 10.6 - 13.53 ) | 1.4 ( 1.25 - 1.58 ) | 16.18 ( 13.96 - 18.59 ) | 0.85 ( 0.73 - 0.97 ) | 35.08 | -2.04 ( -2.35 - -1.73 ) |
| Czech Republic | Both | 348.78 ( 334.73 - 363.74 ) | 2.56 ( 2.45 - 2.67 ) | 253.16 ( 229.24 - 278.93 ) | 1.28 ( 1.17 - 1.41 ) | -27.42 | -2.51 ( -2.59 - -2.43 ) |
| Democratic Republic of the Congo | Both | 277.84 ( 216.92 - 348.92 ) | 1.8 ( 1.44 - 2.2 ) | 480.66 ( 333.22 - 614.32 ) | 1.43 ( 1.03 - 1.8 ) | 73 | -0.94 ( -1.05 - -0.84 ) |
| Denmark | Both | 132.24 ( 126.66 - 138.28 ) | 1.68 ( 1.61 - 1.77 ) | 96.09 ( 87.36 - 104.89 ) | 0.87 ( 0.8 - 0.95 ) | -27.34 | -2.8 ( -2.96 - -2.64 ) |
| Djibouti | Both | 3.45 ( 2.29 - 5.26 ) | 2.1 ( 1.44 - 3.14 ) | 9.21 ( 6.14 - 16.39 ) | 1.57 ( 1.08 - 2.77 ) | 166.74 | -1.44 ( -1.63 - -1.26 ) |
| Dominica | Both | 1.45 ( 1.33 - 1.58 ) | 1.98 ( 1.83 - 2.14 ) | 2.24 ( 2.01 - 2.49 ) | 2.42 ( 2.17 - 2.68 ) | 54.59 | 0.66 ( 0.54 - 0.78 ) |
| Dominican Republic | Both | 66.12 ( 59.24 - 73.9 ) | 1.77 ( 1.58 - 1.98 ) | 142.79 ( 118.3 - 169.08 ) | 1.57 ( 1.3 - 1.86 ) | 115.97 | -0.06 ( -0.48 - 0.36 ) |
| Ecuador | Both | 62.21 ( 58.96 - 65.82 ) | 1.16 ( 1.1 - 1.23 ) | 111.15 ( 99.02 - 124.39 ) | 0.77 ( 0.69 - 0.86 ) | 78.65 | -1.38 ( -1.69 - -1.07 ) |
| Egypt | Both | 265.43 ( 241.82 - 294.43 ) | 0.89 ( 0.82 - 0.99 ) | 503.74 ( 330.65 - 619.29 ) | 0.82 ( 0.54 - 1 ) | 89.78 | -0.2 ( -0.4 - -0.01 ) |
| El Salvador | Both | 28.21 ( 25.71 - 31.02 ) | 0.95 ( 0.86 - 1.04 ) | 51.82 ( 42.25 - 62.73 ) | 0.91 ( 0.74 - 1.1 ) | 83.72 | 0.09 ( -0.18 - 0.36 ) |
| Equatorial Guinea | Both | 5.13 ( 2.96 - 6.93 ) | 2.53 ( 1.53 - 3.33 ) | 5.46 ( 3.84 - 7.48 ) | 1.24 ( 0.89 - 1.68 ) | 6.42 | -3.12 ( -3.5 - -2.73 ) |
| Eritrea | Both | 34.3 ( 24.69 - 44.77 ) | 3.13 ( 2.36 - 3.97 ) | 48.49 ( 36.91 - 74.56 ) | 1.87 ( 1.46 - 2.81 ) | 41.37 | -2.35 ( -2.56 - -2.13 ) |
| Estonia | Both | 53.19 ( 49.78 - 56.81 ) | 2.53 ( 2.37 - 2.7 ) | 33.67 ( 28.77 - 40.07 ) | 1.38 ( 1.18 - 1.64 ) | -36.71 | -2.83 ( -3.16 - -2.5 ) |
| Ethiopia | Both | 338.94 ( 259.24 - 441.7 ) | 1.59 ( 1.24 - 2.06 ) | 350.49 ( 267.99 - 549.62 ) | 0.88 ( 0.67 - 1.4 ) | 3.41 | -2.5 ( -2.6 - -2.39 ) |
| Fiji | Both | 3.33 ( 2.89 - 3.8 ) | 0.99 ( 0.86 - 1.13 ) | 7.81 ( 6.71 - 9.15 ) | 1.17 ( 1.01 - 1.36 ) | 134.99 | 1.05 ( 0.84 - 1.25 ) |
| Finland | Both | 52.89 ( 49.86 - 55.93 ) | 0.74 ( 0.7 - 0.78 ) | 44.97 ( 40.81 - 49.79 ) | 0.38 ( 0.35 - 0.43 ) | -14.99 | -2.38 ( -2.52 - -2.25 ) |
| France | Both | 3114.92 ( 2997.79 - 3248.39 ) | 3.99 ( 3.83 - 4.16 ) | 1685.1 ( 1546.7 - 1844.61 ) | 1.38 ( 1.26 - 1.51 ) | -45.9 | -4.2 ( -4.61 - -3.79 ) |
| Gabon | Both | 13.29 ( 10.99 - 16.4 ) | 2.28 ( 1.91 - 2.82 ) | 18.45 ( 14.9 - 26.94 ) | 1.77 ( 1.43 - 2.61 ) | 38.84 | -0.94 ( -1.03 - -0.84 ) |
| Gambia | Both | 2.86 ( 2.23 - 3.64 ) | 2.4 ( 2.17 - 2.63 ) | 6.66 ( 5.33 - 8.54 ) | 1.25 ( 1.09 - 1.45 ) | 133.09 | -0.19 ( -0.26 - -0.13 ) |
| Georgia | Both | 211.81 ( 197.15 - 226.73 ) | 0.81 ( 0.64 - 1.02 ) | 185.54 ( 168.55 - 203.99 ) | 0.74 ( 0.59 - 0.94 ) | -12.4 | 0.22 ( -0.28 - 0.73 ) |
| Germany | Both | 1932.18 ( 1859.66 - 2007.26 ) | 3.23 ( 3.01 - 3.46 ) | 1686.38 ( 1475.11 - 1911.24 ) | 3.19 ( 2.9 - 3.5 ) | -12.72 | -2.1 ( -2.3 - -1.91 ) |
| Ghana | Both | 79.05 ( 58.03 - 98.48 ) | 1.59 ( 1.52 - 1.65 ) | 227.73 ( 149.37 - 281.78 ) | 0.98 ( 0.85 - 1.12 ) | 188.09 | 1.33 ( 1.06 - 1.6 ) |
| Greece | Both | 351.49 ( 334.51 - 369.8 ) | 1.27 ( 0.94 - 1.56 ) | 381.04 ( 347.15 - 418.05 ) | 1.54 ( 1.06 - 1.87 ) | 8.4 | -1.05 ( -1.16 - -0.93 ) |
| Greenland | Both | 0.67 ( 0.6 - 0.75 ) | 2.22 ( 2.11 - 2.33 ) | 1.16 ( 1.02 - 1.3 ) | 1.63 ( 1.48 - 1.8 ) | 73.43 | -0.37 ( -0.61 - -0.12 ) |
| Grenada | Both | 1.35 ( 1.25 - 1.47 ) | 1.98 ( 1.78 - 2.2 ) | 2.87 ( 2.58 - 3.16 ) | 1.73 ( 1.53 - 1.94 ) | 111.7 | 0.32 ( -0.01 - 0.65 ) |
| Grenadines | Both | 1.71 ( 1.56 - 1.87 ) | 2.95 ( 2.39 - 3.52 ) | 4 ( 3.63 - 4.44 ) | 1.32 ( 1.09 - 1.61 ) | 133.48 | 0.78 ( 0.59 - 0.96 ) |
| Guam | Both | 1.03 ( 0.89 - 1.33 ) | 1.92 ( 1.77 - 2.08 ) | 2.4 ( 2.13 - 2.71 ) | 1.94 ( 1.74 - 2.14 ) | 133.62 | -0.26 ( -0.42 - -0.1 ) |
| Guatemala | Both | 46.95 ( 43.99 - 50.01 ) | 2.31 ( 2.1 - 2.52 ) | 86.67 ( 77.07 - 97.29 ) | 2.91 ( 2.63 - 3.22 ) | 84.6 | -2.46 ( -2.81 - -2.1 ) |
| Guinea | Both | 39.54 ( 33.1 - 47.74 ) | 1.44 ( 1.25 - 1.82 ) | 86.03 ( 65.96 - 107.19 ) | 1.39 ( 1.23 - 1.55 ) | 117.56 | 1.77 ( 1.56 - 1.98 ) |
| Guinea-Bissau | Both | 8.15 ( 5.85 - 10.19 ) | 1.33 ( 1.24 - 1.42 ) | 11.73 ( 9.1 - 14.53 ) | 0.82 ( 0.73 - 0.91 ) | 43.99 | -0.27 ( -0.41 - -0.13 ) |
| Guyana | Both | 4.64 ( 4.29 - 5.05 ) | 1.2 ( 1.01 - 1.45 ) | 7.23 ( 6.15 - 8.36 ) | 1.66 ( 1.3 - 2.06 ) | 55.91 | 0.29 ( 0.04 - 0.55 ) |
| Haiti | Both | 123.88 ( 94.71 - 170.4 ) | 2.03 ( 1.5 - 2.51 ) | 198.04 ( 146.47 - 301.5 ) | 1.74 ( 1.39 - 2.11 ) | 59.86 | -0.68 ( -0.77 - -0.6 ) |
| Honduras | Both | 24.16 ( 20.61 - 28.11 ) | 1.2 ( 1.11 - 1.3 ) | 59.15 ( 46.22 - 73.16 ) | 1.16 ( 1 - 1.34 ) | 144.82 | -0.51 ( -0.62 - -0.39 ) |
| Hungary | Both | 626.56 ( 600.44 - 653.91 ) | 3.86 ( 2.97 - 5.38 ) | 494.33 ( 456.14 - 538.75 ) | 3.11 ( 2.32 - 4.81 ) | -21.1 | -1.65 ( -1.94 - -1.37 ) |
| Iceland | Both | 2.27 ( 2.11 - 2.46 ) | 1.13 ( 0.97 - 1.31 ) | 2.58 ( 2.37 - 2.83 ) | 1.01 ( 0.79 - 1.25 ) | 13.45 | -2.4 ( -2.81 - -2 ) |
| India | Both | 21448.87 ( 18786.38 - 23494.21 ) | 4.27 ( 4.09 - 4.46 ) | 34658.69 ( 32725.15 - 36614.69 ) | 2.81 ( 2.6 - 3.07 ) | 61.59 | -1.3 ( -1.54 - -1.07 ) |
| Indonesia | Both | 1556.89 ( 1347.04 - 2060.21 ) | 0.8 ( 0.74 - 0.87 ) | 2992.35 ( 2455.21 - 4580.25 ) | 0.49 ( 0.45 - 0.54 ) | 92.2 | -0.14 ( -0.23 - -0.05 ) |
| Iran | Both | 693.65 ( 643.78 - 795.83 ) | 4.26 ( 3.74 - 4.66 ) | 1293.95 ( 1242.83 - 1356.45 ) | 3.11 ( 2.94 - 3.29 ) | 86.54 | -0.94 ( -1.21 - -0.67 ) |
| Iraq | Both | 263.6 ( 215.44 - 313.93 ) | 1.58 ( 1.37 - 2.07 ) | 280.04 ( 255.24 - 306.32 ) | 1.48 ( 1.22 - 2.22 ) | 6.24 | -3.93 ( -4.49 - -3.37 ) |
| Ireland | Both | 63.29 ( 59.47 - 67.72 ) | 2.68 ( 2.49 - 3.08 ) | 62.3 ( 55.92 - 69.43 ) | 1.93 ( 1.85 - 2.02 ) | -1.56 | -2.15 ( -2.27 - -2.03 ) |
| Israel | Both | 45.82 ( 42.63 - 49.64 ) | 3.3 ( 2.69 - 3.92 ) | 88.99 ( 80.74 - 98.83 ) | 1.25 ( 1.14 - 1.36 ) | 94.21 | -1.33 ( -1.69 - -0.98 ) |
| Italy | Both | 2520.23 ( 2428.14 - 2617.67 ) | 1.52 ( 1.43 - 1.63 ) | 1594.7 ( 1459.2 - 1740.79 ) | 0.86 ( 0.77 - 0.96 ) | -36.72 | -3.37 ( -3.49 - -3.25 ) |
| Ivory Coast | Both | 64.33 ( 52.55 - 78.07 ) | 0.95 ( 0.88 - 1.02 ) | 142.19 ( 108.58 - 188.23 ) | 0.79 ( 0.72 - 0.87 ) | 121.04 | -0.48 ( -0.7 - -0.25 ) |
| Jamaica | Both | 22.37 ( 20.46 - 24.44 ) | 2.78 ( 2.68 - 2.89 ) | 50.74 ( 39.59 - 61.69 ) | 1.12 ( 1.03 - 1.23 ) | 126.85 | 0.79 ( 0.3 - 1.27 ) |
| Japan | Both | 1008.09 ( 988.4 - 1033.67 ) | 1.49 ( 1.23 - 1.77 ) | 1232.83 ( 1182.44 - 1281.25 ) | 1.36 ( 1.06 - 1.79 ) | 22.29 | -2.46 ( -2.56 - -2.36 ) |
| Jordan | Both | 27.35 ( 22.35 - 33.09 ) | 1.23 ( 1.12 - 1.35 ) | 36.2 ( 30.93 - 43.36 ) | 1.77 ( 1.38 - 2.16 ) | 32.36 | -4.61 ( -5.13 - -4.08 ) |
| Kazakhstan | Both | 431.37 ( 407.85 - 454.73 ) | 0.59 ( 0.57 - 0.6 ) | 244.64 ( 223.66 - 270.44 ) | 0.32 ( 0.31 - 0.34 ) | -43.29 | -3.63 ( -3.93 - -3.32 ) |
| Kenya | Both | 144.81 ( 89.14 - 178.11 ) | 1.88 ( 1.55 - 2.27 ) | 366.16 ( 264.94 - 425.16 ) | 0.67 ( 0.57 - 0.8 ) | 152.86 | -0.02 ( -0.27 - 0.24 ) |
| Kiribati | Both | 0.37 ( 0.34 - 0.41 ) | 3.11 ( 2.95 - 3.27 ) | 0.66 ( 0.56 - 0.75 ) | 1.39 ( 1.28 - 1.53 ) | 76.06 | -0.16 ( -0.36 - 0.04 ) |
| Kuwait | Both | 6.69 ( 6.16 - 7.25 ) | 1.68 ( 1.02 - 2.05 ) | 11.71 ( 10.54 - 13.07 ) | 1.65 ( 1.19 - 1.92 ) | 75 | -2.18 ( -2.56 - -1.8 ) |
| Kyrgyzstan | Both | 65 ( 59.42 - 71.23 ) | 1.06 ( 0.95 - 1.17 ) | 33.24 ( 29.9 - 37.24 ) | 1.05 ( 0.91 - 1.19 ) | -48.86 | -3.84 ( -4.16 - -3.51 ) |
| Laos | Both | 59.86 ( 48.13 - 73.63 ) | 1.08 ( 0.99 - 1.17 ) | 70.74 ( 52.96 - 111.9 ) | 0.54 ( 0.49 - 0.6 ) | 18.18 | -1.83 ( -1.87 - -1.79 ) |
| Latvia | Both | 103.71 ( 98.28 - 109.63 ) | 2.03 ( 1.87 - 2.22 ) | 79.1 ( 67.92 - 91.44 ) | 0.74 ( 0.67 - 0.82 ) | -23.73 | -1.17 ( -1.54 - -0.81 ) |
| Lebanon | Both | 94.75 ( 77.73 - 118.95 ) | 2.79 ( 2.26 - 3.45 ) | 117.3 ( 104.4 - 131.49 ) | 1.74 ( 1.32 - 2.76 ) | 23.8 | -2.89 ( -3.12 - -2.66 ) |
| Lesotho | Both | 27.29 ( 19.36 - 33.17 ) | 2.8 ( 2.65 - 2.96 ) | 32.22 ( 25.61 - 39.77 ) | 2.15 ( 1.85 - 2.49 ) | 18.04 | 0.37 ( 0.05 - 0.69 ) |
| Liberia | Both | 15.07 ( 12.08 - 18.79 ) | 4.17 ( 3.43 - 5.25 ) | 22.25 ( 16.67 - 28.89 ) | 1.98 ( 1.76 - 2.22 ) | 47.61 | -0.18 ( -0.32 - -0.04 ) |
| Libya | Both | 63.54 ( 50.88 - 79.97 ) | 2.64 ( 1.87 - 3.19 ) | 133.66 ( 95.57 - 165.63 ) | 2.62 ( 2.11 - 3.22 ) | 110.36 | -0.53 ( -0.62 - -0.45 ) |
| Lithuania | Both | 140.49 ( 133.58 - 147.53 ) | 1.34 ( 1.08 - 1.66 ) | 134.06 ( 121.46 - 148.08 ) | 1.24 ( 0.94 - 1.58 ) | -4.57 | -0.9 ( -1.26 - -0.53 ) |
| Luxembourg | Both | 12.75 ( 11.76 - 13.86 ) | 3.27 ( 2.64 - 4.1 ) | 9.96 ( 8.69 - 11.42 ) | 2.89 ( 2.08 - 3.58 ) | -21.89 | -3.16 ( -3.32 - -3.01 ) |
| Macedonia | Both | 62.47 ( 56.63 - 69.91 ) | 3.01 ( 2.86 - 3.15 ) | 103.88 ( 91.07 - 119.64 ) | 2.56 ( 2.32 - 2.82 ) | 66.3 | -0.39 ( -0.62 - -0.16 ) |
| Madagascar | Both | 101.76 ( 86.05 - 119.43 ) | 2.33 ( 2.15 - 2.52 ) | 148.1 ( 115.96 - 185.65 ) | 1.06 ( 0.92 - 1.21 ) | 45.55 | -1.42 ( -1.53 - -1.32 ) |
| Malawi | Both | 37.99 ( 22.68 - 49.07 ) | 3.19 ( 2.9 - 3.56 ) | 55.51 ( 47.14 - 65.62 ) | 3.06 ( 2.69 - 3.53 ) | 46.11 | -1.25 ( -1.6 - -0.9 ) |
| Malaysia | Both | 161.31 ( 143.46 - 182.45 ) | 1.87 ( 1.59 - 2.18 ) | 304.63 ( 245.65 - 361.58 ) | 1.32 ( 1.04 - 1.62 ) | 88.85 | -1.75 ( -2 - -1.49 ) |
| Maldives | Both | 1.66 ( 1.23 - 1.96 ) | 0.9 ( 0.58 - 1.15 ) | 2.14 ( 1.89 - 2.41 ) | 0.73 ( 0.63 - 0.87 ) | 29.02 | -3.88 ( -4.12 - -3.64 ) |
| Mali | Both | 49.65 ( 42.73 - 57.74 ) | 1.83 ( 1.62 - 2.07 ) | 70.86 ( 56.95 - 87.83 ) | 1.26 ( 1.03 - 1.49 ) | 42.71 | -1.08 ( -1.27 - -0.89 ) |
| Malta | Both | 8.27 ( 7.65 - 8.96 ) | 2.02 ( 1.61 - 2.35 ) | 9.25 ( 8.38 - 10.16 ) | 0.81 ( 0.71 - 0.92 ) | 11.87 | -2.62 ( -2.79 - -2.44 ) |
| Marshall Islands | Both | 0.33 ( 0.23 - 0.39 ) | 1.2 ( 1.02 - 1.38 ) | 0.64 ( 0.52 - 0.78 ) | 0.87 ( 0.7 - 1.08 ) | 98.28 | 0.31 ( 0.12 - 0.51 ) |
| Mauritania | Both | 12.75 ( 10.45 - 15.3 ) | 1.89 ( 1.76 - 2.05 ) | 21.33 ( 15.78 - 29.12 ) | 1.07 ( 0.97 - 1.17 ) | 67.33 | -0.07 ( -0.28 - 0.14 ) |
| Mauritius | Both | 15.13 ( 14.01 - 16.26 ) | 2 ( 1.46 - 2.38 ) | 17.73 ( 15.78 - 19.78 ) | 2.19 ( 1.81 - 2.59 ) | 17.24 | -2.78 ( -2.95 - -2.61 ) |
| Mexico | Both | 782.38 ( 764.72 - 811.87 ) | 1.25 ( 1.03 - 1.49 ) | 1098.32 ( 1014.76 - 1134.85 ) | 1.14 ( 0.85 - 1.54 ) | 40.38 | -2.7 ( -2.84 - -2.56 ) |
| Micronesia | Both | 0.87 ( 0.72 - 1.13 ) | 2.04 ( 1.9 - 2.2 ) | 1.06 ( 0.87 - 1.27 ) | 1.06 ( 0.95 - 1.18 ) | 21.85 | -0.45 ( -0.48 - -0.43 ) |
| Moldova | Both | 142.07 ( 133.6 - 150.82 ) | 1.87 ( 1.83 - 1.94 ) | 156.41 ( 143.41 - 170.47 ) | 1 ( 0.92 - 1.03 ) | 10.1 | -0.23 ( -0.59 - 0.14 ) |
| Mongolia | Both | 9.94 ( 8.95 - 10.99 ) | 1.84 ( 1.52 - 2.43 ) | 23.42 ( 20.06 - 28.01 ) | 1.63 ( 1.37 - 1.93 ) | 135.48 | 1.57 ( 1.08 - 2.06 ) |
| Montenegro | Both | 29.83 ( 26.48 - 34.15 ) | 2.97 ( 2.8 - 3.15 ) | 39.81 ( 35.02 - 45.48 ) | 2.7 ( 2.48 - 2.93 ) | 33.45 | -0.66 ( -0.92 - -0.41 ) |
| Morocco | Both | 409.72 ( 349.62 - 491.8 ) | 1.02 ( 0.92 - 1.13 ) | 770.26 ( 603.31 - 980.92 ) | 1.35 ( 1.17 - 1.6 ) | 88 | -0.64 ( -0.76 - -0.51 ) |
| Mozambique | Both | 150.28 ( 109.37 - 189.11 ) | 4.53 ( 4.04 - 5.2 ) | 223.29 ( 150.06 - 281.99 ) | 3.98 ( 3.51 - 4.54 ) | 48.58 | -0.61 ( -0.7 - -0.52 ) |
| Myanmar | Both | 707.06 ( 562.17 - 859.02 ) | 2.78 ( 2.37 - 3.35 ) | 792.3 ( 658.25 - 1085.66 ) | 2.38 ( 1.87 - 3.01 ) | 12.06 | -1.87 ( -1.96 - -1.78 ) |
| Namibia | Both | 26.66 ( 22.34 - 31.6 ) | 2.31 ( 1.7 - 2.85 ) | 36.17 ( 30.79 - 42.7 ) | 1.91 ( 1.32 - 2.37 ) | 35.69 | -1.56 ( -2.03 - -1.08 ) |
| Nepal | Both | 461.32 ( 369.19 - 565.98 ) | 2.98 ( 2.4 - 3.6 ) | 675.94 ( 547.61 - 818.32 ) | 1.8 ( 1.51 - 2.44 ) | 46.52 | -1.44 ( -1.79 - -1.1 ) |
| Netherlands | Both | 261.68 ( 249.25 - 273.43 ) | 3.52 ( 2.99 - 4.16 ) | 254.23 ( 233.78 - 276.58 ) | 2.53 ( 2.17 - 2.96 ) | -2.85 | -2.4 ( -2.62 - -2.18 ) |
| New Zealand | Both | 41.36 ( 38.61 - 44.24 ) | 4.57 ( 3.68 - 5.61 ) | 37.9 ( 34.48 - 41.82 ) | 3.09 ( 2.55 - 3.71 ) | -8.37 | -2.93 ( -3.08 - -2.79 ) |
| Nicaragua | Both | 21.12 ( 18.87 - 23.62 ) | 1.3 ( 1.24 - 1.36 ) | 33.69 ( 28.79 - 39.17 ) | 0.76 ( 0.7 - 0.83 ) | 59.54 | -2.39 ( -2.56 - -2.22 ) |
| Niger | Both | 34.51 ( 22.8 - 44.99 ) | 1.03 ( 0.96 - 1.1 ) | 81.35 ( 46.42 - 111.21 ) | 0.5 ( 0.45 - 0.55 ) | 135.75 | -0.02 ( -0.16 - 0.11 ) |
| Nigeria | Both | 718.58 ( 526.75 - 959.13 ) | 1.38 ( 1.23 - 1.54 ) | 946.36 ( 683.87 - 1333.18 ) | 0.77 ( 0.66 - 0.89 ) | 31.7 | -1.28 ( -1.39 - -1.18 ) |
| North Korea | Both | 182.13 ( 143.56 - 225.36 ) | 1.24 ( 0.83 - 1.58 ) | 356.57 ( 287.11 - 431.22 ) | 1.19 ( 0.68 - 1.58 ) | 95.78 | 0.26 ( 0.16 - 0.36 ) |
| Northern Mariana Islands | Both | 0.33 ( 0.27 - 0.46 ) | 1.56 ( 1.16 - 2.07 ) | 0.81 ( 0.7 - 0.94 ) | 1.16 ( 0.85 - 1.6 ) | 145.96 | -0.34 ( -0.51 - -0.18 ) |
| Norway | Both | 50.43 ( 49.16 - 51.72 ) | 1.07 ( 0.86 - 1.31 ) | 38.67 ( 37.01 - 40.82 ) | 1.12 ( 0.91 - 1.34 ) | -23.32 | -2.43 ( -2.54 - -2.32 ) |
| Oman | Both | 8.06 ( 6.26 - 10.27 ) | 2.16 ( 1.76 - 2.92 ) | 12.74 ( 9.9 - 16.07 ) | 1.82 ( 1.57 - 2.1 ) | 58.18 | -2.04 ( -2.21 - -1.88 ) |
| Pakistan | Both | 3101.11 ( 2719.47 - 3512.54 ) | 0.75 ( 0.73 - 0.77 ) | 5911.76 ( 4693.77 - 7220.3 ) | 0.42 ( 0.4 - 0.44 ) | 90.63 | -0.35 ( -0.57 - -0.12 ) |
| Palestine | Both | 10.81 ( 8.1 - 13.37 ) | 1.15 ( 0.89 - 1.45 ) | 17.56 ( 15.39 - 20.11 ) | 0.65 ( 0.51 - 0.81 ) | 62.44 | -2.1 ( -2.26 - -1.94 ) |
| Panama | Both | 32.2 ( 29.98 - 34.71 ) | 5.33 ( 4.69 - 6.04 ) | 44.3 ( 39.69 - 49.02 ) | 5.17 ( 4.14 - 6.3 ) | 37.61 | -2.77 ( -3.06 - -2.48 ) |
| Papua New Guinea | Both | 37.15 ( 30.78 - 44.72 ) | 1.25 ( 0.93 - 1.54 ) | 88.89 ( 73.45 - 108.83 ) | 0.75 ( 0.66 - 0.86 ) | 139.25 | 0.33 ( 0.24 - 0.43 ) |
| Paraguay | Both | 31.18 ( 27.11 - 36.66 ) | 2.15 ( 2.01 - 2.32 ) | 78 ( 62.31 - 96.25 ) | 1.12 ( 1 - 1.24 ) | 150.18 | 0.36 ( 0.23 - 0.49 ) |
| Peru | Both | 163.92 ( 146.74 - 188.45 ) | 2.03 ( 1.71 - 2.37 ) | 176.16 ( 149.34 - 204.56 ) | 2.1 ( 1.76 - 2.53 ) | 7.46 | -3.87 ( -4.21 - -3.52 ) |
| Philippines | Both | 342.47 ( 318.86 - 369.31 ) | 1.4 ( 1.22 - 1.63 ) | 744.28 ( 638.74 - 869.11 ) | 1.48 ( 1.19 - 1.82 ) | 117.33 | -0.16 ( -0.31 - 0 ) |
| Poland | Both | 1807.9 ( 1743.21 - 1877.72 ) | 1.38 ( 1.23 - 1.59 ) | 1668.7 ( 1533.54 - 1817.87 ) | 0.58 ( 0.49 - 0.67 ) | -7.7 | -2.11 ( -2.28 - -1.95 ) |
| Portugal | Both | 444.79 ( 425.4 - 465.68 ) | 1.13 ( 1.05 - 1.21 ) | 393.25 ( 356.19 - 433.14 ) | 1.07 ( 0.92 - 1.25 ) | -11.59 | -2.13 ( -2.31 - -1.96 ) |
| Puerto Rico | Both | 98.77 ( 93.23 - 104.63 ) | 3.98 ( 3.84 - 4.14 ) | 74.11 ( 67.5 - 81.76 ) | 2.46 ( 2.26 - 2.68 ) | -24.96 | -3.93 ( -4.17 - -3.69 ) |
| Qatar | Both | 1.21 ( 0.97 - 1.45 ) | 3.19 ( 3.05 - 3.33 ) | 7.6 ( 6.02 - 9.25 ) | 1.89 ( 1.69 - 2.1 ) | 530.5 | -0.07 ( -1.02 - 0.88 ) |
| Republic of Congo | Both | 28.53 ( 23.18 - 34.1 ) | 2.65 ( 2.51 - 2.81 ) | 43.05 ( 33.93 - 60.46 ) | 1.06 ( 0.97 - 1.17 ) | 50.88 | -1.59 ( -1.75 - -1.42 ) |
| Romania | Both | 860.68 ( 825.13 - 902.41 ) | 1.35 ( 1.11 - 1.63 ) | 1013.71 ( 933.58 - 1107.39 ) | 1.16 ( 0.91 - 1.45 ) | 17.78 | -0.16 ( -0.38 - 0.07 ) |
| Russia | Both | 5983.06 ( 5791.87 - 6330.22 ) | 2.56 ( 2.11 - 3.03 ) | 4315.2 ( 4205.96 - 4431.28 ) | 1.79 ( 1.42 - 2.57 ) | -27.88 | -2.75 ( -3.19 - -2.3 ) |
| Rwanda | Both | 90.64 ( 72.47 - 108.82 ) | 2.93 ( 2.81 - 3.07 ) | 78.2 ( 63.75 - 96.82 ) | 2.97 ( 2.73 - 3.25 ) | -13.73 | -3.77 ( -4.12 - -3.42 ) |
| Saint Lucia | Both | 2.31 ( 2.13 - 2.51 ) | 3.13 ( 3.03 - 3.32 ) | 4.61 ( 4.13 - 5.14 ) | 1.85 ( 1.8 - 1.9 ) | 99.46 | -0.71 ( -0.89 - -0.53 ) |
| Saint Vincent | Both | 1.71 ( 1.56 - 1.87 ) | 2.56 ( 2.36 - 2.78 ) | 4 ( 3.63 - 4.44 ) | 2.2 ( 1.97 - 2.45 ) | 133.48 | 0.78 ( 0.59 - 0.96 ) |
| Samoa | Both | 0.56 ( 0.47 - 0.66 ) | 2.31 ( 2.1 - 2.52 ) | 0.77 ( 0.61 - 0.89 ) | 2.91 ( 2.63 - 3.22 ) | 39.1 | -0.66 ( -0.76 - -0.56 ) |
| Sao Tome and Principe | Both | 0.54 ( 0.47 - 0.63 ) | 0.72 ( 0.61 - 0.85 ) | 0.96 ( 0.77 - 1.17 ) | 0.61 ( 0.47 - 0.7 ) | 77.45 | 0.85 ( 0.75 - 0.94 ) |
| Saudi Arabia | Both | 68.88 ( 47.76 - 89.31 ) | 0.81 ( 0.7 - 0.94 ) | 122.65 ( 102.31 - 149.11 ) | 1.02 ( 0.82 - 1.23 ) | 78.08 | -0.92 ( -1.24 - -0.6 ) |
| Senegal | Both | 43.96 ( 36.4 - 52.78 ) | 1.12 ( 0.78 - 1.45 ) | 101.72 ( 80.83 - 127.7 ) | 0.8 ( 0.69 - 0.96 ) | 131.42 | 0.62 ( 0.41 - 0.84 ) |
| Serbia | Both | 475.93 ( 419.9 - 560.53 ) | 1.38 ( 1.14 - 1.64 ) | 425 ( 382.04 - 471.02 ) | 1.47 ( 1.18 - 1.83 ) | -10.7 | -1.28 ( -1.49 - -1.06 ) |
| Seychelles | Both | 3.4 ( 2.99 - 3.8 ) | 3.86 ( 3.42 - 4.53 ) | 4.92 ( 4.39 - 5.52 ) | 2.7 ( 2.42 - 3.01 ) | 44.74 | -1.17 ( -1.26 - -1.09 ) |
| Sierra Leone | Both | 26.17 ( 19.31 - 32.56 ) | 5.94 ( 5.22 - 6.62 ) | 48.16 ( 39.3 - 58.86 ) | 4.62 ( 4.13 - 5.16 ) | 84.01 | 0.65 ( 0.49 - 0.82 ) |
| Singapore | Both | 28.95 ( 26.87 - 31.21 ) | 1.36 ( 1.01 - 1.69 ) | 25.61 ( 22.94 - 28.38 ) | 1.48 ( 1.23 - 1.78 ) | -11.54 | -4.73 ( -4.92 - -4.54 ) |
| Slovakia | Both | 226.28 ( 211.11 - 240.31 ) | 1.32 ( 1.23 - 1.42 ) | 171.21 ( 150.35 - 197.24 ) | 0.38 ( 0.34 - 0.43 ) | -24.34 | -2.63 ( -2.73 - -2.52 ) |
| Slovenia | Both | 70.29 ( 66.26 - 74.78 ) | 3.78 ( 3.54 - 4.01 ) | 51 ( 45.53 - 56.94 ) | 1.89 ( 1.66 - 2.17 ) | -27.45 | -3.29 ( -3.43 - -3.14 ) |
| Solomon Islands | Both | 2.47 ( 2.03 - 2.96 ) | 2.77 ( 2.61 - 2.95 ) | 5.08 ( 4.24 - 6.13 ) | 1.25 ( 1.12 - 1.4 ) | 105.25 | -0.14 ( -0.23 - -0.05 ) |
| Somalia | Both | 62.17 ( 34.74 - 92.05 ) | 1.85 ( 1.53 - 2.2 ) | 113.69 ( 83.72 - 148.89 ) | 1.7 ( 1.41 - 2.1 ) | 82.86 | -1.48 ( -1.7 - -1.25 ) |
| South Africa | Both | 426.73 ( 375.48 - 543.98 ) | 2.25 ( 1.41 - 3.16 ) | 653.99 ( 615.99 - 704.87 ) | 1.73 ( 1.3 - 2.21 ) | 53.25 | -1.42 ( -2.04 - -0.79 ) |
| South Korea | Both | 679.86 ( 651.23 - 713.01 ) | 1.96 ( 1.72 - 2.52 ) | 433.66 ( 390.36 - 482.47 ) | 1.47 ( 1.39 - 1.58 ) | -36.21 | -7.1 ( -7.81 - -6.4 ) |
| South Sudan | Both | 54.31 ( 31.5 - 79.29 ) | 2.28 ( 2.19 - 2.39 ) | 64.69 ( 46.89 - 88.75 ) | 0.51 ( 0.46 - 0.57 ) | 19.1 | -1.43 ( -1.62 - -1.23 ) |
| Spain | Both | 2101.63 ( 2023.31 - 2190.45 ) | 2.18 ( 1.32 - 3.11 ) | 1478.31 ( 1356.93 - 1620.21 ) | 1.66 ( 1.23 - 2.24 ) | -29.66 | -3.53 ( -3.69 - -3.37 ) |
| Sri Lanka | Both | 88.62 ( 80.25 - 98.35 ) | 3.84 ( 3.7 - 4 ) | 194.12 ( 160.94 - 231.25 ) | 1.64 ( 1.5 - 1.79 ) | 119.06 | 0.73 ( 0.39 - 1.06 ) |
| Sudan | Both | 220.82 ( 171.61 - 282.86 ) | 0.83 ( 0.75 - 0.91 ) | 349.5 ( 264.8 - 460.21 ) | 0.79 ( 0.66 - 0.94 ) | 58.27 | -0.52 ( -0.57 - -0.46 ) |
| Suriname | Both | 2.41 ( 2.18 - 2.66 ) | 2.34 ( 1.83 - 2.99 ) | 6.02 ( 5.23 - 6.97 ) | 1.99 ( 1.52 - 2.64 ) | 149.61 | 0.37 ( 0.14 - 0.6 ) |
| Swaziland | Both | 8.64 ( 6.92 - 10.51 ) | 0.94 ( 0.85 - 1.04 ) | 13.02 ( 9.62 - 16.25 ) | 1.03 ( 0.9 - 1.19 ) | 50.7 | -0.47 ( -0.96 - 0.02 ) |
| Sweden | Both | 85.42 ( 81.16 - 90.05 ) | 2.82 ( 2.26 - 3.41 ) | 68.33 ( 63.43 - 73.91 ) | 2.23 ( 1.68 - 2.76 ) | -20 | -1.97 ( -2.04 - -1.91 ) |
| Switzerland | Both | 130.9 ( 124.64 - 137.46 ) | 0.56 ( 0.53 - 0.59 ) | 92.34 ( 83.31 - 102.87 ) | 0.33 ( 0.31 - 0.36 ) | -29.46 | -2.86 ( -3.01 - -2.72 ) |
| Syria | Both | 52.88 ( 46.23 - 60.66 ) | 1.28 ( 1.21 - 1.34 ) | 95.59 ( 77.96 - 116.51 ) | 0.56 ( 0.5 - 0.62 ) | 80.78 | -1.34 ( -1.68 - -1 ) |
| Tajikistan | Both | 33.74 ( 31 - 36.94 ) | 1.19 ( 1.14 - 1.24 ) | 37.27 ( 32.54 - 42.14 ) | 0.74 ( 0.67 - 0.81 ) | 10.48 | -2 ( -2.23 - -1.77 ) |
| Tanzania | Both | 220.99 ( 160.22 - 295.29 ) | 1.14 ( 1.05 - 1.24 ) | 325.7 ( 259.96 - 450.71 ) | 0.7 ( 0.61 - 0.79 ) | 47.38 | -1.73 ( -1.93 - -1.52 ) |
| Thailand | Both | 852.66 ( 769.47 - 937.83 ) | 1.92 ( 1.43 - 2.54 ) | 1230.21 ( 1067.53 - 1423.05 ) | 1.33 ( 1.07 - 1.81 ) | 44.28 | -2.92 ( -3.14 - -2.69 ) |
| Timor-Leste | Both | 5 ( 4.05 - 6.87 ) | 1.79 ( 1.46 - 2.45 ) | 12.56 ( 9.47 - 19.46 ) | 1.62 ( 1.24 - 2.48 ) | 150.96 | -0.34 ( -0.48 - -0.19 ) |
| Tobago | Both | 13.86 ( 12.85 - 14.92 ) | 1.63 ( 1.51 - 1.75 ) | 20.31 ( 16.2 - 25.25 ) | 1.14 ( 0.91 - 1.42 ) | 46.57 | -1.7 ( -1.96 - -1.44 ) |
| Togo | Both | 15.65 ( 12.88 - 18.24 ) | 1.29 ( 1.07 - 1.49 ) | 43.74 ( 33.32 - 54.78 ) | 1.3 ( 1.01 - 1.6 ) | 179.44 | 0.3 ( 0.17 - 0.42 ) |
| Tonga | Both | 0.61 ( 0.52 - 0.78 ) | 1.19 ( 1.02 - 1.52 ) | 0.93 ( 0.77 - 1.16 ) | 1.19 ( 0.99 - 1.49 ) | 52.28 | 0.1 ( 0.02 - 0.18 ) |
| Trinidad | Both | 13.86 ( 12.85 - 14.92 ) | 1.63 ( 1.51 - 1.75 ) | 20.31 ( 16.2 - 25.25 ) | 1.14 ( 0.91 - 1.42 ) | 46.57 | -1.7 ( -1.96 - -1.44 ) |
| Tunisia | Both | 144.7 ( 124.8 - 173.95 ) | 2.88 ( 2.51 - 3.46 ) | 270.65 ( 207.38 - 340.66 ) | 2.24 ( 1.72 - 2.81 ) | 87.04 | -1.2 ( -1.34 - -1.06 ) |
| Turkey | Both | 1246.18 ( 1085.52 - 1496.5 ) | 3.43 ( 2.98 - 4.11 ) | 1482.53 ( 1294.34 - 1681.48 ) | 1.71 ( 1.49 - 1.94 ) | 18.97 | -2.86 ( -3.09 - -2.64 ) |
| Turkmenistan | Both | 46.02 ( 42.83 - 49.47 ) | 2.23 ( 2.08 - 2.39 ) | 36.48 ( 32.41 - 40.21 ) | 0.94 ( 0.84 - 1.03 ) | -20.73 | -3.78 ( -4.39 - -3.17 ) |
| Uganda | Both | 105.65 ( 84.81 - 129.26 ) | 1.57 ( 1.28 - 1.93 ) | 177.28 ( 143.59 - 233.19 ) | 1.27 ( 1.03 - 1.66 ) | 67.8 | -1.35 ( -1.72 - -0.98 ) |
| UK | Both | 982.9 ( 966.26 - 1000.54 ) | 1.07 ( 1.05 - 1.09 ) | 889.69 ( 868.6 - 912.91 ) | 0.71 ( 0.7 - 0.73 ) | -9.48 | -1.82 ( -1.94 - -1.69 ) |
| Ukraine | Both | 2487.83 ( 2369.16 - 2604.34 ) | 3.42 ( 3.26 - 3.57 ) | 1814.91 ( 1671.39 - 1962.82 ) | 2.43 ( 2.24 - 2.63 ) | -27.05 | -2.26 ( -2.69 - -1.83 ) |
| United Arab Emirates | Both | 14.15 ( 10.5 - 19.86 ) | 2.79 ( 2.17 - 3.56 ) | 122.92 ( 83.86 - 168.96 ) | 2.84 ( 1.77 - 3.85 ) | 769.01 | 0.11 ( 0.01 - 0.22 ) |
| Uruguay | Both | 167.81 ( 158.6 - 177.55 ) | 4.23 ( 4 - 4.48 ) | 122.06 ( 105.51 - 139.45 ) | 2.35 ( 2.01 - 2.7 ) | -27.26 | -2.19 ( -2.34 - -2.05 ) |
| USA | Both | 4506.31 ( 4443.34 - 4605.19 ) | 1.41 ( 1.39 - 1.44 ) | 5173.26 ( 5007.16 - 5328.88 ) | 0.95 ( 0.92 - 0.98 ) | 14.8 | -1.88 ( -2.01 - -1.75 ) |
| Uzbekistan | Both | 201.03 ( 190.9 - 211.37 ) | 1.65 ( 1.57 - 1.73 ) | 362.35 ( 320.6 - 410.53 ) | 1.61 ( 1.43 - 1.81 ) | 80.24 | 0.37 ( -0.22 - 0.97 ) |
| Vanuatu | Both | 1.3 ( 0.97 - 2.07 ) | 2.06 ( 1.58 - 3.25 ) | 3.39 ( 2.38 - 6.5 ) | 2.19 ( 1.56 - 4.18 ) | 162.09 | 0.3 ( 0.24 - 0.36 ) |
| Venezuela | Both | 236.69 ( 224.56 - 249.78 ) | 2.49 ( 2.36 - 2.62 ) | 557.34 ( 468.8 - 659.41 ) | 2.02 ( 1.71 - 2.38 ) | 135.47 | -1 ( -1.13 - -0.87 ) |
| Vietnam | Both | 636.25 ( 536.58 - 738.86 ) | 1.53 ( 1.3 - 1.77 ) | 1422.9 ( 1175.98 - 1748.76 ) | 1.51 ( 1.25 - 1.84 ) | 123.64 | -0.03 ( -0.16 - 0.1 ) |
| Virgin Islands | Both | 1.94 ( 1.74 - 2.16 ) | 2.25 ( 2.03 - 2.5 ) | 4.56 ( 3.61 - 5.37 ) | 2.37 ( 1.89 - 2.78 ) | 134.67 | 0.41 ( 0.29 - 0.52 ) |
| Yemen | Both | 130.89 ( 80.76 - 180.44 ) | 2.5 ( 1.61 - 3.38 ) | 296.4 ( 222.68 - 379.57 ) | 2.37 ( 1.8 - 3 ) | 126.45 | -0.23 ( -0.31 - -0.14 ) |
| Zambia | Both | 81.36 ( 64.11 - 105.75 ) | 2.69 ( 2.18 - 3.48 ) | 110.1 ( 88.38 - 163.44 ) | 1.67 ( 1.35 - 2.46 ) | 35.33 | -2.48 ( -2.79 - -2.16 ) |
| Zimbabwe | Both | 95.01 ( 80.4 - 110.98 ) | 2.2 ( 1.87 - 2.56 ) | 158.26 ( 125.84 - 192.31 ) | 2.21 ( 1.79 - 2.67 ) | 66.57 | 0.48 ( -0.18 - 1.15 ) |
| Afghanistan | Female | 80.2 ( 49.74 - 104.89 ) | 2.35 ( 1.5 - 3.03 ) | 137.43 ( 101.76 - 185.19 ) | 2.16 ( 1.65 - 2.77 ) | 71.36 | -0.38 ( -0.61 - -0.16 ) |
| Albania | Female | 9.14 ( 8.24 - 10.18 ) | 0.77 ( 0.69 - 0.86 ) | 8.95 ( 6.71 - 11.95 ) | 0.41 ( 0.31 - 0.55 ) | -1.98 | -2.9 ( -3.49 - -2.3 ) |
| Algeria | Female | 31.49 ( 26.98 - 37 ) | 0.47 ( 0.4 - 0.55 ) | 52.44 ( 45.47 - 60.74 ) | 0.31 ( 0.27 - 0.36 ) | 66.51 | -1.17 ( -1.3 - -1.04 ) |
| American Samoa | Female | 0.02 ( 0.02 - 0.03 ) | 0.2 ( 0.17 - 0.23 ) | 0.09 ( 0.07 - 0.1 ) | 0.4 ( 0.34 - 0.47 ) | 294.06 | 3.72 ( 3.08 - 4.36 ) |
| Andorra | Female | 0.07 ( 0.05 - 0.09 ) | 0.23 ( 0.17 - 0.32 ) | 0.12 ( 0.09 - 0.15 ) | 0.17 ( 0.13 - 0.22 ) | 77.11 | -1.54 ( -1.98 - -1.11 ) |
| Angola | Female | 11.47 ( 8.31 - 15.07 ) | 0.55 ( 0.41 - 0.71 ) | 19.33 ( 14.72 - 25.39 ) | 0.33 ( 0.25 - 0.43 ) | 68.59 | -2.11 ( -2.32 - -1.9 ) |
| Antigua | Female | 0.03 ( 0.03 - 0.04 ) | 0.11 ( 0.1 - 0.13 ) | 0.05 ( 0.05 - 0.06 ) | 0.1 ( 0.09 - 0.11 ) | 58.66 | -0.62 ( -0.77 - -0.47 ) |
| Argentina | Female | 74.39 ( 68.92 - 80.27 ) | 0.41 ( 0.38 - 0.44 ) | 123.16 ( 104.98 - 145.81 ) | 0.41 ( 0.35 - 0.48 ) | 65.56 | -0.15 ( -0.41 - 0.11 ) |
| Armenia | Female | 9.73 ( 8.74 - 10.92 ) | 0.62 ( 0.56 - 0.7 ) | 11.2 ( 10.03 - 12.5 ) | 0.47 ( 0.42 - 0.52 ) | 15.02 | -0.6 ( -1.36 - 0.18 ) |
| Australia | Female | 30.28 ( 27.92 - 32.76 ) | 0.27 ( 0.25 - 0.29 ) | 33.88 ( 28.57 - 40.09 ) | 0.16 ( 0.13 - 0.18 ) | 11.9 | -2.34 ( -2.48 - -2.2 ) |
| Austria | Female | 16.37 ( 15.01 - 17.95 ) | 0.23 ( 0.22 - 0.25 ) | 16.74 ( 14.77 - 19.02 ) | 0.18 ( 0.16 - 0.21 ) | 2.24 | -1.14 ( -1.34 - -0.94 ) |
| Azerbaijan | Female | 22.29 ( 19.88 - 24.85 ) | 0.73 ( 0.65 - 0.81 ) | 34.11 ( 28.8 - 40.08 ) | 0.65 ( 0.55 - 0.76 ) | 53 | -1.15 ( -1.79 - -0.52 ) |
| Bahamas | Female | 0.45 ( 0.41 - 0.5 ) | 0.52 ( 0.47 - 0.58 ) | 0.87 ( 0.73 - 1.02 ) | 0.44 ( 0.37 - 0.51 ) | 93.72 | -0.68 ( -0.87 - -0.5 ) |
| Bahrain | Female | 0.28 ( 0.24 - 0.33 ) | 0.36 ( 0.31 - 0.42 ) | 0.36 ( 0.3 - 0.42 ) | 0.11 ( 0.09 - 0.13 ) | 27.01 | -6.09 ( -7.29 - -4.87 ) |
| Bangladesh | Female | 522.15 ( 423.83 - 650.33 ) | 2.23 ( 1.79 - 2.79 ) | 716.68 ( 550.44 - 896.36 ) | 1.18 ( 0.91 - 1.48 ) | 37.26 | -1.82 ( -2.02 - -1.61 ) |
| Barbados | Female | 0.71 ( 0.63 - 0.79 ) | 0.4 ( 0.36 - 0.44 ) | 0.89 ( 0.76 - 1.02 ) | 0.33 ( 0.28 - 0.37 ) | 26.38 | -0.84 ( -1.05 - -0.63 ) |
| Barbuda | Female | 0.03 ( 0.03 - 0.04 ) | 0.11 ( 0.1 - 0.13 ) | 0.05 ( 0.05 - 0.06 ) | 0.1 ( 0.09 - 0.11 ) | 58.66 | -0.62 ( -0.77 - -0.47 ) |
| Belarus | Female | 14.68 ( 13.45 - 16.03 ) | 0.18 ( 0.16 - 0.2 ) | 9.62 ( 8.38 - 10.84 ) | 0.1 ( 0.09 - 0.11 ) | -34.49 | -2.67 ( -2.98 - -2.36 ) |
| Belgium | Female | 41.54 ( 38.26 - 45.03 ) | 0.48 ( 0.45 - 0.52 ) | 31.1 ( 27.47 - 35.28 ) | 0.27 ( 0.24 - 0.31 ) | -25.12 | -2.12 ( -2.3 - -1.94 ) |
| Belize | Female | 0.2 ( 0.18 - 0.23 ) | 0.43 ( 0.38 - 0.49 ) | 0.44 ( 0.39 - 0.49 ) | 0.35 ( 0.31 - 0.39 ) | 113.55 | -0.79 ( -1.18 - -0.39 ) |
| Benin | Female | 2.81 ( 2.3 - 3.42 ) | 0.27 ( 0.22 - 0.32 ) | 6.19 ( 4.55 - 8.24 ) | 0.26 ( 0.19 - 0.34 ) | 120.24 | 0.07 ( -0.03 - 0.17 ) |
| Bermuda | Female | 0.18 ( 0.16 - 0.2 ) | 0.5 ( 0.45 - 0.56 ) | 0.16 ( 0.14 - 0.19 ) | 0.21 ( 0.18 - 0.25 ) | -10.73 | -3.53 ( -3.86 - -3.19 ) |
| Bhutan | Female | 2.94 ( 2.29 - 3.86 ) | 2.16 ( 1.68 - 2.81 ) | 2.8 ( 2.08 - 3.64 ) | 0.92 ( 0.69 - 1.19 ) | -4.76 | -3.44 ( -3.61 - -3.28 ) |
| Bolivia | Female | 19.03 ( 15.6 - 23.15 ) | 1.09 ( 0.89 - 1.32 ) | 25.49 ( 19.3 - 32.91 ) | 0.57 ( 0.43 - 0.73 ) | 33.95 | -2.75 ( -2.97 - -2.52 ) |
| Bosnia and Herzegovina | Female | 19.82 ( 17.62 - 22.2 ) | 0.86 ( 0.77 - 0.96 ) | 17.61 ( 15.16 - 20.58 ) | 0.53 ( 0.46 - 0.61 ) | -11.12 | -2.71 ( -3.32 - -2.1 ) |
| Botswana | Female | 1.53 ( 1.09 - 1.98 ) | 0.46 ( 0.33 - 0.59 ) | 3.12 ( 2.54 - 3.81 ) | 0.41 ( 0.33 - 0.49 ) | 104.86 | 0.9 ( 0.02 - 1.78 ) |
| Brazil | Female | 319.51 ( 307.86 - 331.46 ) | 0.67 ( 0.65 - 0.7 ) | 646.7 ( 619.35 - 676.1 ) | 0.52 ( 0.49 - 0.54 ) | 102.41 | -1.05 ( -1.14 - -0.96 ) |
| Brunei | Female | 0.68 ( 0.59 - 0.79 ) | 1.52 ( 1.3 - 1.75 ) | 0.46 ( 0.4 - 0.53 ) | 0.32 ( 0.28 - 0.37 ) | -32.5 | -7.1 ( -7.7 - -6.5 ) |
| Bulgaria | Female | 18.81 ( 17.13 - 20.58 ) | 0.28 ( 0.25 - 0.3 ) | 21.81 ( 19.02 - 24.64 ) | 0.29 ( 0.25 - 0.32 ) | 15.98 | 0.05 ( -0.13 - 0.22 ) |
| Burkina Faso | Female | 9.36 ( 7.6 - 11.41 ) | 0.4 ( 0.32 - 0.48 ) | 15.43 ( 12.12 - 19.16 ) | 0.34 ( 0.27 - 0.42 ) | 64.96 | -0.52 ( -0.67 - -0.36 ) |
| Burundi | Female | 16.27 ( 12.47 - 21.45 ) | 1.21 ( 0.94 - 1.59 ) | 13.37 ( 9.52 - 17.47 ) | 0.61 ( 0.44 - 0.8 ) | -17.83 | -3.1 ( -3.37 - -2.82 ) |
| Cambodia | Female | 35.33 ( 28.61 - 42.63 ) | 1.3 ( 1.06 - 1.55 ) | 41.43 ( 32.6 - 52.34 ) | 0.64 ( 0.51 - 0.8 ) | 17.24 | -2.86 ( -2.96 - -2.76 ) |
| Cameroon | Female | 7.91 ( 6.51 - 9.77 ) | 0.33 ( 0.28 - 0.41 ) | 16.24 ( 11.82 - 22.11 ) | 0.29 ( 0.21 - 0.39 ) | 105.33 | -0.35 ( -0.51 - -0.19 ) |
| Canada | Female | 71.37 ( 66.81 - 76.04 ) | 0.39 ( 0.36 - 0.42 ) | 76.99 ( 67.89 - 87 ) | 0.21 ( 0.19 - 0.24 ) | 7.88 | -2.52 ( -2.67 - -2.37 ) |
| Cape Verde | Female | 0.14 ( 0.12 - 0.16 ) | 0.1 ( 0.09 - 0.12 ) | 0.66 ( 0.56 - 0.76 ) | 0.25 ( 0.22 - 0.29 ) | 374.77 | 3.32 ( 2.58 - 4.07 ) |
| Central African Republic | Female | 4.96 ( 3.69 - 6.24 ) | 0.76 ( 0.58 - 0.94 ) | 6.26 ( 4.23 - 8.48 ) | 0.55 ( 0.38 - 0.73 ) | 26.26 | -1.22 ( -1.34 - -1.11 ) |
| Chad | Female | 4.09 ( 3.31 - 4.93 ) | 0.27 ( 0.22 - 0.33 ) | 7.63 ( 5.78 - 9.95 ) | 0.32 ( 0.24 - 0.41 ) | 86.81 | 0.83 ( 0.72 - 0.94 ) |
| Chile | Female | 20.51 ( 18.56 - 22.46 ) | 0.37 ( 0.34 - 0.41 ) | 24.12 ( 20.27 - 28.55 ) | 0.19 ( 0.16 - 0.22 ) | 17.63 | -2.46 ( -2.78 - -2.15 ) |
| China | Female | 2586.73 ( 2444.32 - 2805.89 ) | 0.6 ( 0.57 - 0.65 ) | 3351.27 ( 3148.36 - 3552.87 ) | 0.35 ( 0.32 - 0.37 ) | 29.56 | -1.86 ( -2.2 - -1.52 ) |
| Colombia | Female | 100.84 ( 94.93 - 107.65 ) | 1.15 ( 1.08 - 1.23 ) | 101.15 ( 84.85 - 119.32 ) | 0.34 ( 0.28 - 0.4 ) | 0.3 | -5.38 ( -5.68 - -5.08 ) |
| Comoros | Female | 0.98 ( 0.67 - 1.3 ) | 0.88 ( 0.6 - 1.15 ) | 1.25 ( 0.85 - 1.67 ) | 0.5 ( 0.34 - 0.66 ) | 27.07 | -2.39 ( -2.53 - -2.25 ) |
| Costa Rica | Female | 3.96 ( 3.62 - 4.32 ) | 0.44 ( 0.4 - 0.48 ) | 5.03 ( 4.4 - 5.73 ) | 0.19 ( 0.17 - 0.22 ) | 27.11 | -3.46 ( -3.86 - -3.06 ) |
| Croatia | Female | 15.34 ( 13.95 - 16.91 ) | 0.4 ( 0.37 - 0.44 ) | 12.08 ( 10.5 - 13.8 ) | 0.24 ( 0.21 - 0.27 ) | -21.26 | -1.97 ( -2.45 - -1.48 ) |
| Cuba | Female | 71.07 ( 65.82 - 76.41 ) | 1.34 ( 1.25 - 1.44 ) | 115.75 ( 98.7 - 136.29 ) | 1.16 ( 0.98 - 1.37 ) | 62.86 | -0.7 ( -0.89 - -0.52 ) |
| Cyprus | Female | 1.14 ( 0.99 - 1.31 ) | 0.25 ( 0.22 - 0.29 ) | 1 ( 0.84 - 1.19 ) | 0.1 ( 0.09 - 0.12 ) | -12.82 | -3.88 ( -4.22 - -3.54 ) |
| Czech Republic | Female | 25.32 ( 23.27 - 27.53 ) | 0.32 ( 0.29 - 0.34 ) | 23.7 ( 20.92 - 26.66 ) | 0.22 ( 0.19 - 0.24 ) | -6.37 | -1.12 ( -1.32 - -0.91 ) |
| Democratic Republic of the Congo | Female | 41.73 ( 31.29 - 53.15 ) | 0.47 ( 0.36 - 0.6 ) | 74.34 ( 53.67 - 99.45 ) | 0.4 ( 0.29 - 0.53 ) | 78.15 | -0.65 ( -0.73 - -0.58 ) |
| Denmark | Female | 27.48 ( 25.43 - 30.06 ) | 0.64 ( 0.59 - 0.7 ) | 17.45 ( 15.24 - 19.74 ) | 0.3 ( 0.26 - 0.34 ) | -36.5 | -3.2 ( -3.38 - -3.02 ) |
| Djibouti | Female | 0.63 ( 0.43 - 0.86 ) | 0.76 ( 0.54 - 1.06 ) | 1.24 ( 0.81 - 1.82 ) | 0.45 ( 0.3 - 0.65 ) | 96.95 | -2.4 ( -2.61 - -2.19 ) |
| Dominica | Female | 0.29 ( 0.26 - 0.32 ) | 0.66 ( 0.59 - 0.73 ) | 0.29 ( 0.25 - 0.33 ) | 0.59 ( 0.5 - 0.68 ) | 2.56 | -0.5 ( -0.65 - -0.35 ) |
| Dominican Republic | Female | 15 ( 13.15 - 17.04 ) | 0.79 ( 0.69 - 0.9 ) | 25.82 ( 20.98 - 31.04 ) | 0.54 ( 0.43 - 0.65 ) | 72.16 | -1.3 ( -1.7 - -0.9 ) |
| Ecuador | Female | 14.86 ( 13.72 - 16.07 ) | 0.53 ( 0.49 - 0.57 ) | 26.98 ( 23.08 - 31.2 ) | 0.35 ( 0.3 - 0.41 ) | 81.57 | -1.7 ( -2.13 - -1.27 ) |
| Egypt | Female | 46.5 ( 40.35 - 53.97 ) | 0.27 ( 0.24 - 0.31 ) | 69.57 ( 56.03 - 85.49 ) | 0.21 ( 0.17 - 0.25 ) | 49.62 | -0.87 ( -0.99 - -0.75 ) |
| El Salvador | Female | 2.97 ( 2.66 - 3.32 ) | 0.19 ( 0.17 - 0.21 ) | 6.84 ( 5.38 - 8.62 ) | 0.2 ( 0.16 - 0.26 ) | 130.36 | 0.59 ( 0.33 - 0.85 ) |
| Equatorial Guinea | Female | 0.78 ( 0.57 - 1.02 ) | 0.69 ( 0.51 - 0.88 ) | 0.74 ( 0.45 - 1.13 ) | 0.28 ( 0.17 - 0.43 ) | -5.73 | -3.8 ( -4.12 - -3.48 ) |
| Eritrea | Female | 6.34 ( 4.68 - 8.19 ) | 1.05 ( 0.8 - 1.33 ) | 9.28 ( 6.58 - 12.19 ) | 0.65 ( 0.47 - 0.84 ) | 46.37 | -1.91 ( -2.01 - -1.8 ) |
| Estonia | Female | 3.22 ( 2.94 - 3.53 ) | 0.25 ( 0.22 - 0.27 ) | 2.29 ( 1.85 - 2.78 ) | 0.15 ( 0.12 - 0.18 ) | -28.95 | -2.35 ( -2.67 - -2.04 ) |
| Ethiopia | Female | 73.33 ( 57.45 - 90.35 ) | 0.66 ( 0.52 - 0.81 ) | 54.75 ( 46.86 - 63.6 ) | 0.27 ( 0.23 - 0.31 ) | -25.33 | -3.82 ( -4.04 - -3.6 ) |
| Fiji | Female | 0.79 ( 0.64 - 0.97 ) | 0.42 ( 0.34 - 0.52 ) | 1.69 ( 1.34 - 2.09 ) | 0.44 ( 0.35 - 0.54 ) | 113.16 | 0.48 ( 0.29 - 0.67 ) |
| Finland | Female | 7.31 ( 6.65 - 7.98 ) | 0.17 ( 0.16 - 0.19 ) | 6.1 ( 5.32 - 6.93 ) | 0.1 ( 0.08 - 0.11 ) | -16.55 | -2.1 ( -2.23 - -1.98 ) |
| France | Female | 188.3 ( 178.32 - 198.57 ) | 0.4 ( 0.38 - 0.42 ) | 192.01 ( 171.58 - 215.74 ) | 0.28 ( 0.25 - 0.32 ) | 1.97 | -1.36 ( -1.57 - -1.14 ) |
| Gabon | Female | 1.55 ( 1.22 - 1.95 ) | 0.48 ( 0.39 - 0.61 ) | 1.52 ( 1.12 - 2.03 ) | 0.28 ( 0.21 - 0.38 ) | -1.41 | -2.07 ( -2.37 - -1.77 ) |
| Gambia | Female | 0.33 ( 0.26 - 0.42 ) | 0.69 ( 0.61 - 0.78 ) | 0.92 ( 0.72 - 1.16 ) | 0.21 ( 0.18 - 0.24 ) | 177.73 | 0.11 ( 0.03 - 0.2 ) |
| Georgia | Female | 20.23 ( 18.26 - 22.43 ) | 0.19 ( 0.15 - 0.24 ) | 15.32 ( 13.58 - 17.18 ) | 0.19 ( 0.15 - 0.24 ) | -24.26 | -1.08 ( -1.72 - -0.44 ) |
| Germany | Female | 185.79 ( 176.26 - 196.39 ) | 0.55 ( 0.5 - 0.6 ) | 219.93 ( 185.16 - 257.14 ) | 0.44 ( 0.39 - 0.49 ) | 18.38 | -0.46 ( -0.63 - -0.28 ) |
| Ghana | Female | 9.19 ( 7.03 - 11.91 ) | 0.25 ( 0.24 - 0.26 ) | 20.31 ( 16.01 - 25.89 ) | 0.24 ( 0.2 - 0.28 ) | 120.99 | -0.12 ( -0.22 - -0.02 ) |
| Greece | Female | 25.72 ( 23.49 - 28.03 ) | 0.27 ( 0.21 - 0.35 ) | 31.11 ( 27.16 - 35.29 ) | 0.25 ( 0.19 - 0.31 ) | 20.97 | -0.66 ( -0.87 - -0.44 ) |
| Greenland | Female | 0.04 ( 0.03 - 0.04 ) | 0.3 ( 0.28 - 0.33 ) | 0.06 ( 0.05 - 0.07 ) | 0.24 ( 0.21 - 0.27 ) | 61.18 | 0.06 ( -0.48 - 0.6 ) |
| Grenada | Female | 0.27 ( 0.24 - 0.3 ) | 0.22 ( 0.19 - 0.25 ) | 0.28 ( 0.24 - 0.32 ) | 0.19 ( 0.16 - 0.23 ) | 3.3 | -1.76 ( -2.07 - -1.45 ) |
| Grenadines | Female | 0.19 ( 0.17 - 0.21 ) | 1.12 ( 0.92 - 1.37 ) | 0.23 ( 0.2 - 0.26 ) | 0.41 ( 0.31 - 0.52 ) | 21.7 | -1.19 ( -1.49 - -0.89 ) |
| Guam | Female | 0.06 ( 0.05 - 0.07 ) | 0.64 ( 0.57 - 0.72 ) | 0.43 ( 0.36 - 0.51 ) | 0.35 ( 0.31 - 0.4 ) | 611.95 | 5.39 ( 4.69 - 6.09 ) |
| Guatemala | Female | 10.1 ( 9.2 - 11.08 ) | 0.45 ( 0.41 - 0.5 ) | 17.5 ( 14.67 - 20.54 ) | 0.34 ( 0.3 - 0.39 ) | 73.37 | -2.48 ( -3.13 - -1.83 ) |
| Guinea | Female | 5.06 ( 4.14 - 6.16 ) | 0.16 ( 0.13 - 0.18 ) | 8.55 ( 6.69 - 10.89 ) | 0.47 ( 0.39 - 0.55 ) | 68.84 | 0.68 ( 0.54 - 0.83 ) |
| Guinea-Bissau | Female | 0.87 ( 0.65 - 1.12 ) | 0.56 ( 0.51 - 0.61 ) | 1.28 ( 0.97 - 1.65 ) | 0.3 ( 0.25 - 0.36 ) | 47.53 | -0.35 ( -0.47 - -0.23 ) |
| Guyana | Female | 0.63 ( 0.57 - 0.7 ) | 0.29 ( 0.24 - 0.35 ) | 0.92 ( 0.77 - 1.09 ) | 0.33 ( 0.26 - 0.42 ) | 46.57 | -0.44 ( -0.81 - -0.08 ) |
| Haiti | Female | 20.89 ( 16.67 - 25.69 ) | 0.41 ( 0.31 - 0.52 ) | 28.2 ( 20.66 - 37 ) | 0.35 ( 0.27 - 0.45 ) | 35.02 | -1.71 ( -1.79 - -1.62 ) |
| Honduras | Female | 1.9 ( 1.6 - 2.28 ) | 0.32 ( 0.28 - 0.35 ) | 4.71 ( 3.43 - 6.05 ) | 0.29 ( 0.24 - 0.34 ) | 147.45 | -0.82 ( -1.03 - -0.62 ) |
| Hungary | Female | 53.99 ( 49.47 - 58.88 ) | 1.27 ( 1.03 - 1.54 ) | 62.1 ( 55.26 - 70.51 ) | 0.82 ( 0.61 - 1.06 ) | 15.02 | 0.07 ( -0.35 - 0.49 ) |
| Iceland | Female | 0.41 ( 0.37 - 0.45 ) | 0.19 ( 0.16 - 0.22 ) | 0.31 ( 0.27 - 0.35 ) | 0.16 ( 0.11 - 0.2 ) | -24.5 | -3.31 ( -3.44 - -3.18 ) |
| India | Female | 4468.82 ( 4007.53 - 4950.06 ) | 0.66 ( 0.6 - 0.72 ) | 7644.12 ( 6986.95 - 8221.35 ) | 0.62 ( 0.55 - 0.71 ) | 71.05 | -1.52 ( -1.83 - -1.21 ) |
| Indonesia | Female | 398.97 ( 357.49 - 444.81 ) | 0.27 ( 0.24 - 0.3 ) | 491.46 ( 447.2 - 546.2 ) | 0.11 ( 0.1 - 0.13 ) | 23.18 | -1.79 ( -1.83 - -1.75 ) |
| Iran | Female | 168.99 ( 157.17 - 181 ) | 1.81 ( 1.61 - 2.01 ) | 280.52 ( 268.57 - 295.07 ) | 1.33 ( 1.22 - 1.43 ) | 66 | -2.07 ( -2.61 - -1.54 ) |
| Iraq | Female | 66.39 ( 53.4 - 82.21 ) | 0.75 ( 0.67 - 0.84 ) | 68.65 ( 59.8 - 78.86 ) | 0.46 ( 0.42 - 0.51 ) | 3.39 | -3.94 ( -4.56 - -3.31 ) |
| Ireland | Female | 12.39 ( 11.16 - 13.63 ) | 1.35 ( 1.26 - 1.44 ) | 8.67 ( 7.47 - 9.96 ) | 0.84 ( 0.81 - 0.88 ) | -30.01 | -3.64 ( -3.84 - -3.44 ) |
| Israel | Female | 6.34 ( 5.74 - 6.98 ) | 1.58 ( 1.27 - 1.95 ) | 11.21 ( 9.8 - 12.79 ) | 0.6 ( 0.52 - 0.68 ) | 76.87 | -1.71 ( -2.08 - -1.33 ) |
| Italy | Female | 174.15 ( 164.83 - 184.09 ) | 0.54 ( 0.49 - 0.59 ) | 161.55 ( 142.09 - 181.07 ) | 0.23 ( 0.19 - 0.26 ) | -7.24 | -1.73 ( -1.84 - -1.63 ) |
| Ivory Coast | Female | 2.04 ( 1.67 - 2.49 ) | 0.25 ( 0.22 - 0.27 ) | 4.38 ( 3.36 - 5.62 ) | 0.18 ( 0.15 - 0.2 ) | 114.45 | -0.6 ( -0.78 - -0.42 ) |
| Jamaica | Female | 3.01 ( 2.68 - 3.37 ) | 0.34 ( 0.32 - 0.36 ) | 3.43 ( 2.73 - 4.26 ) | 0.2 ( 0.18 - 0.23 ) | 13.93 | -1.19 ( -1.56 - -0.82 ) |
| Japan | Female | 137.19 ( 133.29 - 141.08 ) | 0.11 ( 0.09 - 0.14 ) | 136.28 ( 129.37 - 143.88 ) | 0.1 ( 0.07 - 0.12 ) | -0.66 | -2.88 ( -3.03 - -2.73 ) |
| Jordan | Female | 3.47 ( 2.87 - 4.22 ) | 0.3 ( 0.27 - 0.34 ) | 3.48 ( 2.81 - 4.21 ) | 0.22 ( 0.18 - 0.28 ) | 0.29 | -5.93 ( -6.96 - -4.88 ) |
| Kazakhstan | Female | 54 ( 49.56 - 58.85 ) | 0.14 ( 0.14 - 0.14 ) | 25.14 ( 21.9 - 28.63 ) | 0.07 ( 0.06 - 0.07 ) | -53.43 | -4.23 ( -4.63 - -3.83 ) |
| Kenya | Female | 20.84 ( 17.82 - 24.18 ) | 0.48 ( 0.39 - 0.58 ) | 39.47 ( 34.02 - 45.26 ) | 0.13 ( 0.11 - 0.16 ) | 89.35 | -1.35 ( -1.54 - -1.15 ) |
| Kiribati | Female | 0.17 ( 0.15 - 0.2 ) | 0.67 ( 0.62 - 0.73 ) | 0.28 ( 0.22 - 0.34 ) | 0.25 ( 0.22 - 0.28 ) | 59.07 | -0.57 ( -0.71 - -0.42 ) |
| Kuwait | Female | 0.98 ( 0.86 - 1.1 ) | 0.46 ( 0.39 - 0.53 ) | 1.01 ( 0.88 - 1.16 ) | 0.33 ( 0.29 - 0.38 ) | 3.75 | -4.95 ( -6 - -3.88 ) |
| Kyrgyzstan | Female | 5.82 ( 5.16 - 6.54 ) | 0.83 ( 0.71 - 0.97 ) | 4.25 ( 3.78 - 4.76 ) | 0.72 ( 0.56 - 0.9 ) | -27.04 | -2.85 ( -3.34 - -2.37 ) |
| Laos | Female | 16.32 ( 12.49 - 21.15 ) | 0.38 ( 0.33 - 0.43 ) | 13.26 ( 10.35 - 16.92 ) | 0.1 ( 0.08 - 0.11 ) | -18.74 | -3.33 ( -3.43 - -3.24 ) |
| Latvia | Female | 6.59 ( 5.95 - 7.26 ) | 0.31 ( 0.28 - 0.35 ) | 4.16 ( 3.44 - 4.96 ) | 0.17 ( 0.15 - 0.19 ) | -36.88 | -1.66 ( -1.97 - -1.34 ) |
| Lebanon | Female | 14.54 ( 11.55 - 17.7 ) | 1.44 ( 1.1 - 1.84 ) | 20.02 ( 16.79 - 23.68 ) | 0.62 ( 0.48 - 0.78 ) | 37.67 | -2.73 ( -3 - -2.45 ) |
| Lesotho | Female | 3.47 ( 2.52 - 4.44 ) | 0.28 ( 0.25 - 0.31 ) | 4.53 ( 3 - 6.27 ) | 0.17 ( 0.14 - 0.2 ) | 30.39 | 1.02 ( 0.62 - 1.41 ) |
| Liberia | Female | 1.61 ( 1.26 - 2.01 ) | 1.15 ( 0.91 - 1.39 ) | 2.57 ( 1.95 - 3.32 ) | 0.61 ( 0.51 - 0.72 ) | 59.83 | -0.32 ( -0.46 - -0.18 ) |
| Libya | Female | 5.99 ( 4.76 - 7.37 ) | 0.62 ( 0.45 - 0.79 ) | 14.4 ( 11.16 - 17.95 ) | 0.64 ( 0.42 - 0.88 ) | 140.41 | -0.21 ( -0.34 - -0.08 ) |
| Lithuania | Female | 6.79 ( 6.15 - 7.46 ) | 0.31 ( 0.24 - 0.38 ) | 6.63 ( 5.77 - 7.66 ) | 0.28 ( 0.21 - 0.36 ) | -2.41 | -0.8 ( -1.16 - -0.43 ) |
| Luxembourg | Female | 1.35 ( 1.22 - 1.49 ) | 0.64 ( 0.51 - 0.78 ) | 1.54 ( 1.28 - 1.82 ) | 0.61 ( 0.47 - 0.76 ) | 13.4 | -1.36 ( -1.49 - -1.24 ) |
| Macedonia | Female | 5.43 ( 4.77 - 6.14 ) | 0.24 ( 0.22 - 0.26 ) | 7.08 ( 6 - 8.28 ) | 0.2 ( 0.18 - 0.23 ) | 30.31 | -1.08 ( -1.47 - -0.7 ) |
| Madagascar | Female | 20.99 ( 16.46 - 25.47 ) | 0.43 ( 0.39 - 0.47 ) | 28.8 ( 20.17 - 38.66 ) | 0.3 ( 0.25 - 0.35 ) | 37.17 | -1.91 ( -2.05 - -1.77 ) |
| Malawi | Female | 12.4 ( 8.04 - 15.77 ) | 0.53 ( 0.47 - 0.59 ) | 13.35 ( 10.34 - 16.86 ) | 0.42 ( 0.36 - 0.49 ) | 7.64 | -2.96 ( -3.39 - -2.53 ) |
| Malaysia | Female | 25.84 ( 22.48 - 29.54 ) | 0.75 ( 0.59 - 0.91 ) | 44.16 ( 36.94 - 52.25 ) | 0.48 ( 0.34 - 0.65 ) | 70.94 | -1.85 ( -2.05 - -1.64 ) |
| Maldives | Female | 0.12 ( 0.1 - 0.14 ) | 0.54 ( 0.37 - 0.68 ) | 0.1 ( 0.09 - 0.12 ) | 0.31 ( 0.24 - 0.39 ) | -9.81 | -5.6 ( -5.89 - -5.31 ) |
| Mali | Female | 11.42 ( 9.56 - 13.59 ) | 0.55 ( 0.48 - 0.64 ) | 14.01 ( 10.88 - 17.61 ) | 0.36 ( 0.31 - 0.43 ) | 22.71 | -1.92 ( -2.06 - -1.78 ) |
| Malta | Female | 0.77 ( 0.69 - 0.85 ) | 0.31 ( 0.26 - 0.37 ) | 1.09 ( 0.95 - 1.25 ) | 0.08 ( 0.07 - 0.1 ) | 41.27 | -1.69 ( -1.93 - -1.44 ) |
| Marshall Islands | Female | 0.06 ( 0.05 - 0.08 ) | 0.52 ( 0.44 - 0.62 ) | 0.14 ( 0.11 - 0.19 ) | 0.33 ( 0.26 - 0.42 ) | 124.5 | 0.49 ( 0.21 - 0.78 ) |
| Mauritania | Female | 1.75 ( 1.4 - 2.15 ) | 0.32 ( 0.29 - 0.35 ) | 2.31 ( 1.74 - 3.03 ) | 0.24 ( 0.21 - 0.27 ) | 32.33 | -1 ( -1.13 - -0.88 ) |
| Mauritius | Female | 2.53 ( 2.28 - 2.79 ) | 0.77 ( 0.63 - 0.94 ) | 1.66 ( 1.44 - 1.89 ) | 0.87 ( 0.68 - 1.1 ) | -34.51 | -6.19 ( -6.92 - -5.45 ) |
| Mexico | Female | 136.32 ( 132.39 - 140.37 ) | 0.32 ( 0.26 - 0.4 ) | 146.42 ( 139.87 - 152.9 ) | 0.24 ( 0.18 - 0.31 ) | 7.41 | -4 ( -4.27 - -3.74 ) |
| Micronesia | Female | 0.21 ( 0.17 - 0.26 ) | 0.63 ( 0.57 - 0.69 ) | 0.29 ( 0.23 - 0.37 ) | 0.18 ( 0.16 - 0.21 ) | 35.68 | -0.39 ( -0.45 - -0.32 ) |
| Moldova | Female | 5.75 ( 5.15 - 6.41 ) | 0.62 ( 0.6 - 0.64 ) | 5.37 ( 4.78 - 6.01 ) | 0.25 ( 0.24 - 0.26 ) | -6.53 | -0.5 ( -1.04 - 0.03 ) |
| Mongolia | Female | 2.94 ( 2.47 - 3.5 ) | 0.85 ( 0.67 - 1.03 ) | 3.37 ( 2.77 - 4.06 ) | 0.77 ( 0.61 - 0.97 ) | 14.63 | -2.06 ( -2.55 - -1.57 ) |
| Montenegro | Female | 6.13 ( 5.3 - 7.03 ) | 0.21 ( 0.19 - 0.24 ) | 8.48 ( 7.19 - 9.94 ) | 0.16 ( 0.15 - 0.18 ) | 38.22 | -0.35 ( -0.74 - 0.04 ) |
| Morocco | Female | 39.6 ( 33.56 - 46.81 ) | 0.5 ( 0.42 - 0.6 ) | 55.95 ( 43.77 - 70.67 ) | 0.32 ( 0.27 - 0.4 ) | 41.28 | -1.58 ( -1.62 - -1.54 ) |
| Mozambique | Female | 28.13 ( 20.28 - 36.25 ) | 1.72 ( 1.49 - 1.97 ) | 37.72 ( 27.51 - 49.62 ) | 1.59 ( 1.35 - 1.87 ) | 34.07 | -1.29 ( -1.45 - -1.13 ) |
| Myanmar | Female | 234.99 ( 171.75 - 312.96 ) | 0.51 ( 0.44 - 0.61 ) | 181.99 ( 138.25 - 233.14 ) | 0.34 ( 0.27 - 0.43 ) | -22.55 | -3.63 ( -3.89 - -3.36 ) |
| Namibia | Female | 6.1 ( 4.78 - 7.56 ) | 0.79 ( 0.58 - 1.02 ) | 6.06 ( 4.52 - 8.19 ) | 0.58 ( 0.43 - 0.76 ) | -0.62 | -3.36 ( -4.15 - -2.56 ) |
| Nepal | Female | 142.22 ( 95.01 - 190.27 ) | 1.86 ( 1.36 - 2.45 ) | 187.26 ( 127.14 - 243.28 ) | 0.74 ( 0.56 - 0.95 ) | 31.67 | -2.18 ( -2.52 - -1.83 ) |
| Netherlands | Female | 28.36 ( 26.11 - 30.75 ) | 1.49 ( 1.18 - 1.84 ) | 46 ( 40.13 - 51.63 ) | 0.73 ( 0.55 - 0.99 ) | 62.18 | -0.2 ( -0.6 - 0.2 ) |
| New Zealand | Female | 7.04 ( 6.4 - 7.73 ) | 2.84 ( 1.83 - 3.78 ) | 5.39 ( 4.8 - 6.09 ) | 1.61 ( 1.08 - 2.1 ) | -23.48 | -2.97 ( -3.22 - -2.72 ) |
| Nicaragua | Female | 4.8 ( 4.18 - 5.48 ) | 0.25 ( 0.23 - 0.28 ) | 4.95 ( 4.08 - 5.89 ) | 0.26 ( 0.23 - 0.3 ) | 3.33 | -4.17 ( -4.61 - -3.72 ) |
| Niger | Female | 3.83 ( 3.06 - 4.72 ) | 0.32 ( 0.29 - 0.35 ) | 9.17 ( 6.65 - 12.12 ) | 0.13 ( 0.12 - 0.15 ) | 139.6 | -0.29 ( -0.39 - -0.18 ) |
| Nigeria | Female | 34.65 ( 26.3 - 45.06 ) | 0.58 ( 0.51 - 0.66 ) | 46.05 ( 31.02 - 65.29 ) | 0.21 ( 0.17 - 0.25 ) | 32.9 | -1.76 ( -1.89 - -1.64 ) |
| North Korea | Female | 34.03 ( 25.07 - 43.41 ) | 0.27 ( 0.22 - 0.33 ) | 64.55 ( 49.46 - 81.82 ) | 0.26 ( 0.19 - 0.33 ) | 89.71 | 0.27 ( 0.12 - 0.42 ) |
| Northern Mariana Islands | Female | 0.03 ( 0.02 - 0.03 ) | 0.17 ( 0.13 - 0.21 ) | 0.15 ( 0.13 - 0.18 ) | 0.11 ( 0.08 - 0.15 ) | 478.95 | 2.79 ( 2.1 - 3.48 ) |
| Norway | Female | 8.02 ( 7.76 - 8.3 ) | 0.34 ( 0.25 - 0.44 ) | 6.88 ( 6.53 - 7.29 ) | 0.35 ( 0.27 - 0.44 ) | -14.17 | -1.95 ( -2.22 - -1.68 ) |
| Oman | Female | 0.92 ( 0.7 - 1.19 ) | 0.38 ( 0.31 - 0.46 ) | 1.16 ( 0.91 - 1.44 ) | 0.59 ( 0.5 - 0.71 ) | 26.24 | -2.76 ( -3.07 - -2.45 ) |
| Pakistan | Female | 604.32 ( 517.2 - 698.36 ) | 0.21 ( 0.2 - 0.22 ) | 1092.61 ( 821.47 - 1399.08 ) | 0.14 ( 0.13 - 0.15 ) | 80.8 | -0.99 ( -1.29 - -0.69 ) |
| Palestine | Female | 1.56 ( 1.23 - 1.95 ) | 0.29 ( 0.22 - 0.37 ) | 2.41 ( 2.06 - 2.8 ) | 0.15 ( 0.12 - 0.18 ) | 54.58 | -1.93 ( -2.18 - -1.68 ) |
| Panama | Female | 2.86 ( 2.6 - 3.13 ) | 2.23 ( 1.9 - 2.57 ) | 4.3 ( 3.79 - 4.86 ) | 1.94 ( 1.47 - 2.46 ) | 50.26 | -2.44 ( -2.83 - -2.05 ) |
| Papua New Guinea | Female | 9.09 ( 7.15 - 11.31 ) | 0.31 ( 0.25 - 0.39 ) | 20.78 ( 16.24 - 25.93 ) | 0.19 ( 0.16 - 0.22 ) | 128.56 | 0.29 ( 0.15 - 0.44 ) |
| Paraguay | Female | 2.65 ( 2.31 - 3.02 ) | 0.38 ( 0.35 - 0.42 ) | 5.4 ( 4.18 - 6.8 ) | 0.21 ( 0.18 - 0.23 ) | 103.87 | -0.39 ( -0.88 - 0.1 ) |
| Peru | Female | 38.55 ( 33.35 - 44.44 ) | 0.97 ( 0.78 - 1.2 ) | 40.98 ( 32.67 - 51.1 ) | 0.97 ( 0.77 - 1.2 ) | 6.28 | -3.98 ( -4.4 - -3.55 ) |
| Philippines | Female | 85.21 ( 76.71 - 93.65 ) | 0.23 ( 0.2 - 0.27 ) | 143.62 ( 116.19 - 175.05 ) | 0.2 ( 0.15 - 0.25 ) | 68.55 | -1.46 ( -1.75 - -1.16 ) |
| Poland | Female | 154.88 ( 146.22 - 163.97 ) | 0.61 ( 0.52 - 0.7 ) | 181.17 ( 160.92 - 204.02 ) | 0.26 ( 0.21 - 0.32 ) | 16.98 | -1 ( -1.14 - -0.85 ) |
| Portugal | Female | 33.99 ( 31.33 - 36.94 ) | 0.57 ( 0.52 - 0.63 ) | 20.02 ( 17.41 - 22.8 ) | 0.4 ( 0.33 - 0.49 ) | -41.09 | -4.81 ( -5.36 - -4.25 ) |
| Puerto Rico | Female | 11.57 ( 10.48 - 12.7 ) | 0.6 ( 0.57 - 0.63 ) | 8.43 ( 7.46 - 9.53 ) | 0.48 ( 0.43 - 0.54 ) | -27.1 | -4.05 ( -4.49 - -3.61 ) |
| Qatar | Female | 0.08 ( 0.06 - 0.1 ) | 0.43 ( 0.4 - 0.47 ) | 0.39 ( 0.3 - 0.51 ) | 0.14 ( 0.12 - 0.16 ) | 383.5 | -1.17 ( -1.96 - -0.36 ) |
| Republic of Congo | Female | 4.46 ( 3.53 - 5.5 ) | 0.58 ( 0.52 - 0.63 ) | 6.47 ( 4.71 - 8.36 ) | 0.21 ( 0.18 - 0.23 ) | 45.25 | -1.56 ( -1.77 - -1.35 ) |
| Romania | Female | 61.18 ( 56.52 - 65.97 ) | 0.23 ( 0.18 - 0.28 ) | 58.25 ( 51.59 - 65.69 ) | 0.14 ( 0.11 - 0.18 ) | -4.79 | -1.09 ( -1.27 - -0.91 ) |
| Russia | Female | 338.66 ( 323.12 - 357.34 ) | 0.7 ( 0.56 - 0.86 ) | 242.77 ( 232.96 - 254.29 ) | 0.49 ( 0.36 - 0.62 ) | -28.31 | -2.37 ( -2.68 - -2.06 ) |
| Rwanda | Female | 19.87 ( 16.14 - 24.17 ) | 0.4 ( 0.37 - 0.43 ) | 13.97 ( 10.54 - 18.09 ) | 0.3 ( 0.27 - 0.34 ) | -29.71 | -4.7 ( -5.13 - -4.27 ) |
| Saint Lucia | Female | 0.29 ( 0.26 - 0.32 ) | 0.29 ( 0.27 - 0.3 ) | 0.44 ( 0.39 - 0.5 ) | 0.17 ( 0.17 - 0.18 ) | 55.49 | -1.57 ( -1.84 - -1.3 ) |
| Saint Vincent | Female | 0.19 ( 0.17 - 0.21 ) | 0.57 ( 0.51 - 0.63 ) | 0.23 ( 0.2 - 0.26 ) | 0.41 ( 0.35 - 0.46 ) | 21.7 | -1.19 ( -1.49 - -0.89 ) |
| Samoa | Female | 0.07 ( 0.05 - 0.09 ) | 0.45 ( 0.41 - 0.5 ) | 0.12 ( 0.09 - 0.15 ) | 0.34 ( 0.3 - 0.39 ) | 74.91 | 0.18 ( 0.05 - 0.31 ) |
| Sao Tome and Principe | Female | 0.06 ( 0.05 - 0.07 ) | 0.17 ( 0.13 - 0.21 ) | 0.11 ( 0.08 - 0.14 ) | 0.17 ( 0.13 - 0.22 ) | 74.29 | 0.4 ( 0.18 - 0.62 ) |
| Saudi Arabia | Female | 5.37 ( 4.18 - 6.85 ) | 0.17 ( 0.14 - 0.2 ) | 10.52 ( 8.57 - 13.09 ) | 0.2 ( 0.15 - 0.26 ) | 95.92 | -1.05 ( -1.2 - -0.9 ) |
| Senegal | Female | 4.03 ( 3.28 - 4.91 ) | 0.2 ( 0.15 - 0.25 ) | 9.06 ( 6.88 - 11.54 ) | 0.14 ( 0.12 - 0.17 ) | 124.52 | 0.2 ( 0.08 - 0.33 ) |
| Serbia | Female | 30.42 ( 26.54 - 35.2 ) | 0.25 ( 0.2 - 0.3 ) | 40.69 ( 35.46 - 46.41 ) | 0.25 ( 0.19 - 0.32 ) | 33.75 | 0.11 ( -0.14 - 0.37 ) |
| Seychelles | Female | 0.35 ( 0.31 - 0.4 ) | 0.48 ( 0.42 - 0.55 ) | 0.36 ( 0.31 - 0.41 ) | 0.48 ( 0.42 - 0.54 ) | 1.52 | -1.82 ( -1.95 - -1.69 ) |
| Sierra Leone | Female | 2.81 ( 2.22 - 3.53 ) | 1.07 ( 0.95 - 1.23 ) | 5.95 ( 4.63 - 7.6 ) | 0.63 ( 0.55 - 0.73 ) | 111.91 | 1.22 ( 1 - 1.44 ) |
| Singapore | Female | 3.07 ( 2.79 - 3.36 ) | 0.29 ( 0.23 - 0.37 ) | 2.56 ( 2.26 - 2.91 ) | 0.36 ( 0.28 - 0.45 ) | -16.49 | -4.94 ( -5.31 - -4.57 ) |
| Slovakia | Female | 11.09 ( 9.98 - 12.32 ) | 0.27 ( 0.25 - 0.3 ) | 9.02 ( 7.65 - 10.57 ) | 0.07 ( 0.06 - 0.08 ) | -18.61 | -2.05 ( -2.17 - -1.93 ) |
| Slovenia | Female | 4.68 ( 4.23 - 5.17 ) | 0.32 ( 0.29 - 0.36 ) | 4.9 ( 4.22 - 5.63 ) | 0.18 ( 0.15 - 0.21 ) | 4.66 | -2.01 ( -2.29 - -1.72 ) |
| Solomon Islands | Female | 0.53 ( 0.42 - 0.67 ) | 0.31 ( 0.29 - 0.34 ) | 1.22 ( 0.97 - 1.49 ) | 0.22 ( 0.19 - 0.25 ) | 127.26 | 0.08 ( -0.05 - 0.21 ) |
| Somalia | Female | 12.94 ( 7.8 - 18.72 ) | 0.79 ( 0.63 - 0.97 ) | 21.32 ( 14.32 - 29.77 ) | 0.77 ( 0.62 - 0.93 ) | 64.71 | -1.85 ( -2.05 - -1.65 ) |
| South Africa | Female | 59.86 ( 54.71 - 65.72 ) | 0.89 ( 0.59 - 1.25 ) | 81.58 ( 76.52 - 87.2 ) | 0.61 ( 0.42 - 0.84 ) | 36.27 | -1.72 ( -2.42 - -1 ) |
| South Korea | Female | 120.97 ( 112.45 - 129.87 ) | 0.47 ( 0.43 - 0.51 ) | 44.39 ( 38.76 - 50.82 ) | 0.32 ( 0.3 - 0.34 ) | -63.3 | -9.67 ( -10.55 - -8.78 ) |
| South Sudan | Female | 8.25 ( 5.3 - 11.74 ) | 0.75 ( 0.7 - 0.81 ) | 9.38 ( 6.44 - 13.29 ) | 0.09 ( 0.08 - 0.11 ) | 13.73 | -1.92 ( -2.11 - -1.74 ) |
| Spain | Female | 64.91 ( 60.6 - 69.19 ) | 0.74 ( 0.49 - 1.02 ) | 78.75 ( 69.88 - 88.09 ) | 0.49 ( 0.34 - 0.7 ) | 21.33 | -0.26 ( -0.49 - -0.04 ) |
| Sri Lanka | Female | 10.02 ( 8.73 - 11.45 ) | 0.2 ( 0.19 - 0.22 ) | 36.85 ( 26.81 - 46.95 ) | 0.16 ( 0.14 - 0.18 ) | 267.85 | 2.82 ( 2.37 - 3.27 ) |
| Sudan | Female | 52.49 ( 39.8 - 65.84 ) | 0.19 ( 0.16 - 0.21 ) | 69.82 ( 49.2 - 93.83 ) | 0.28 ( 0.2 - 0.35 ) | 33.03 | -1.22 ( -1.26 - -1.18 ) |
| Suriname | Female | 0.44 ( 0.39 - 0.49 ) | 1.09 ( 0.83 - 1.35 ) | 0.82 ( 0.7 - 0.95 ) | 0.78 ( 0.56 - 1.04 ) | 87.32 | -0.85 ( -1.11 - -0.6 ) |
| Swaziland | Female | 1.12 ( 0.87 - 1.38 ) | 0.33 ( 0.29 - 0.37 ) | 1.52 ( 1.03 - 2.11 ) | 0.26 ( 0.23 - 0.31 ) | 36.29 | -0.53 ( -1.1 - 0.05 ) |
| Sweden | Female | 15.29 ( 14.14 - 16.4 ) | 0.65 ( 0.51 - 0.81 ) | 12.28 ( 11.09 - 13.58 ) | 0.47 ( 0.32 - 0.64 ) | -19.68 | -2.03 ( -2.15 - -1.9 ) |
| Switzerland | Female | 17.31 ( 15.84 - 18.83 ) | 0.19 ( 0.17 - 0.2 ) | 13.86 ( 12.16 - 15.72 ) | 0.12 ( 0.1 - 0.13 ) | -19.95 | -2.52 ( -2.68 - -2.36 ) |
| Syria | Female | 8.54 ( 7.23 - 10.02 ) | 0.29 ( 0.27 - 0.32 ) | 12.31 ( 9.79 - 14.9 ) | 0.16 ( 0.14 - 0.18 ) | 44.16 | -2.24 ( -2.68 - -1.8 ) |
| Tajikistan | Female | 6.15 ( 5.53 - 6.83 ) | 0.2 ( 0.19 - 0.22 ) | 13.01 ( 10.7 - 15.7 ) | 0.08 ( 0.07 - 0.09 ) | 111.61 | -0.04 ( -0.39 - 0.31 ) |
| Tanzania | Female | 46.56 ( 35.23 - 56.96 ) | 0.38 ( 0.34 - 0.42 ) | 53.79 ( 42.77 - 67.14 ) | 0.41 ( 0.34 - 0.49 ) | 15.53 | -2.8 ( -3.1 - -2.49 ) |
| Thailand | Female | 130.84 ( 115.19 - 148.04 ) | 0.77 ( 0.6 - 0.93 ) | 110.75 ( 94.07 - 128.89 ) | 0.41 ( 0.33 - 0.51 ) | -15.35 | -5.13 ( -5.44 - -4.82 ) |
| Timor-Leste | Female | 1.42 ( 1.1 - 1.82 ) | 0.98 ( 0.78 - 1.23 ) | 2.13 ( 1.68 - 2.69 ) | 0.54 ( 0.43 - 0.67 ) | 50.26 | -2.42 ( -2.57 - -2.27 ) |
| Tobago | Female | 1.82 ( 1.64 - 2.01 ) | 0.41 ( 0.37 - 0.45 ) | 1.33 ( 1.01 - 1.72 ) | 0.14 ( 0.11 - 0.18 ) | -26.72 | -5.39 ( -6.11 - -4.67 ) |
| Togo | Female | 1.98 ( 1.6 - 2.42 ) | 0.29 ( 0.24 - 0.35 ) | 4.54 ( 3.41 - 5.98 ) | 0.24 ( 0.19 - 0.31 ) | 129 | -0.59 ( -0.71 - -0.48 ) |
| Tonga | Female | 0.13 ( 0.11 - 0.15 ) | 0.46 ( 0.4 - 0.54 ) | 0.19 ( 0.15 - 0.24 ) | 0.45 ( 0.35 - 0.56 ) | 49.11 | -0.08 ( -0.18 - 0.01 ) |
| Trinidad | Female | 1.82 ( 1.64 - 2.01 ) | 0.41 ( 0.37 - 0.45 ) | 1.33 ( 1.01 - 1.72 ) | 0.14 ( 0.11 - 0.18 ) | -26.72 | -5.39 ( -6.11 - -4.67 ) |
| Tunisia | Female | 9.69 ( 8.41 - 11.16 ) | 0.4 ( 0.35 - 0.46 ) | 17.84 ( 13.24 - 23.53 ) | 0.29 ( 0.21 - 0.38 ) | 84.2 | -1.38 ( -1.52 - -1.25 ) |
| Turkey | Female | 121.53 ( 104.02 - 141.35 ) | 0.63 ( 0.54 - 0.73 ) | 138.76 ( 119.08 - 158.89 ) | 0.29 ( 0.25 - 0.33 ) | 14.18 | -3.16 ( -3.5 - -2.83 ) |
| Turkmenistan | Female | 6.3 ( 5.69 - 6.9 ) | 0.55 ( 0.49 - 0.6 ) | 10.35 ( 8.91 - 12 ) | 0.49 ( 0.42 - 0.56 ) | 64.35 | -1.02 ( -2.8 - 0.79 ) |
| Uganda | Female | 28.67 ( 22.97 - 35.13 ) | 0.82 ( 0.66 - 0.99 ) | 30.09 ( 24 - 37.76 ) | 0.39 ( 0.31 - 0.48 ) | 4.96 | -3.58 ( -3.97 - -3.2 ) |
| UK | Female | 216.81 ( 211.49 - 221.97 ) | 0.41 ( 0.4 - 0.42 ) | 175.55 ( 169.47 - 182.04 ) | 0.26 ( 0.25 - 0.27 ) | -19.03 | -2 ( -2.15 - -1.85 ) |
| Ukraine | Female | 95.38 ( 88.62 - 102.82 ) | 0.21 ( 0.19 - 0.22 ) | 63.57 ( 57.45 - 70.51 ) | 0.15 ( 0.13 - 0.16 ) | -33.35 | -1.56 ( -1.83 - -1.28 ) |
| United Arab Emirates | Female | 1.71 ( 1.2 - 2.5 ) | 1.07 ( 0.77 - 1.54 ) | 8.15 ( 5.89 - 11.18 ) | 0.87 ( 0.66 - 1.16 ) | 377.26 | -0.72 ( -0.98 - -0.47 ) |
| Uruguay | Female | 8.53 ( 7.73 - 9.41 ) | 0.38 ( 0.34 - 0.41 ) | 13.02 ( 10.88 - 15.58 ) | 0.41 ( 0.34 - 0.49 ) | 52.52 | 0.41 ( 0.23 - 0.59 ) |
| USA | Female | 805.81 ( 787.43 - 824.57 ) | 0.45 ( 0.44 - 0.46 ) | 872.65 ( 832.44 - 912.14 ) | 0.3 ( 0.28 - 0.31 ) | 8.29 | -1.96 ( -2.13 - -1.8 ) |
| Uzbekistan | Female | 33.97 ( 30.84 - 37.18 ) | 0.5 ( 0.45 - 0.55 ) | 132.87 ( 109.8 - 157.95 ) | 1.02 ( 0.85 - 1.21 ) | 291.19 | 3.12 ( 1.92 - 4.34 ) |
| Vanuatu | Female | 0.19 ( 0.15 - 0.25 ) | 0.64 ( 0.51 - 0.81 ) | 0.52 ( 0.39 - 0.66 ) | 0.66 ( 0.51 - 0.84 ) | 165.86 | 0.2 ( 0.09 - 0.3 ) |
| Venezuela | Female | 43.84 ( 40.07 - 47.76 ) | 0.88 ( 0.8 - 0.96 ) | 70.75 ( 56.06 - 86.49 ) | 0.48 ( 0.38 - 0.59 ) | 61.41 | -2.56 ( -2.8 - -2.33 ) |
| Vietnam | Female | 84.1 ( 67.62 - 100.2 ) | 0.35 ( 0.28 - 0.42 ) | 121.56 ( 95.23 - 152.64 ) | 0.23 ( 0.18 - 0.29 ) | 44.55 | -1.74 ( -1.87 - -1.62 ) |
| Virgin Islands | Female | 0.2 ( 0.17 - 0.24 ) | 0.45 ( 0.39 - 0.53 ) | 0.35 ( 0.29 - 0.42 ) | 0.34 ( 0.29 - 0.41 ) | 73.56 | -1.19 ( -1.33 - -1.06 ) |
| Yemen | Female | 32.18 ( 20.99 - 43.77 ) | 1.18 ( 0.8 - 1.6 ) | 65.69 ( 47.41 - 87.91 ) | 0.96 ( 0.71 - 1.28 ) | 104.11 | -0.91 ( -1.01 - -0.81 ) |
| Zambia | Female | 18 ( 13.87 - 22.5 ) | 1.17 ( 0.92 - 1.45 ) | 18.46 ( 13.64 - 24.04 ) | 0.53 ( 0.4 - 0.69 ) | 2.52 | -3.97 ( -4.55 - -3.39 ) |
| Zimbabwe | Female | 8.97 ( 7.37 - 10.89 ) | 0.41 ( 0.34 - 0.5 ) | 19.02 ( 14.26 - 24.28 ) | 0.47 ( 0.36 - 0.6 ) | 112.03 | 1.73 ( 0.91 - 2.56 ) |
| Afghanistan | Male | 209.9 ( 103.05 - 304.53 ) | 5.27 ( 2.73 - 7.58 ) | 260.46 ( 189.14 - 349.21 ) | 5.18 ( 3.78 - 7.14 ) | 24.09 | -0.08 ( -0.32 - 0.16 ) |
| Albania | Male | 58.01 ( 53.08 - 63.46 ) | 5.8 ( 5.33 - 6.3 ) | 79.18 ( 60.08 - 101.83 ) | 3.97 ( 3.03 - 5.07 ) | 36.49 | -1.26 ( -1.52 - -1.01 ) |
| Algeria | Male | 229.25 ( 195.91 - 265.4 ) | 3.69 ( 3.17 - 4.25 ) | 383.69 ( 327.52 - 441.87 ) | 2.31 ( 1.96 - 2.66 ) | 67.37 | -1.7 ( -1.76 - -1.65 ) |
| American Samoa | Male | 0.3 ( 0.25 - 0.38 ) | 2.88 ( 2.4 - 3.81 ) | 0.37 ( 0.3 - 0.44 ) | 2.11 ( 1.72 - 2.53 ) | 25.51 | -1.17 ( -1.41 - -0.94 ) |
| Andorra | Male | 0.56 ( 0.41 - 0.79 ) | 1.9 ( 1.4 - 2.66 ) | 0.78 ( 0.62 - 1.01 ) | 1.18 ( 0.93 - 1.52 ) | 39.13 | -1.88 ( -1.93 - -1.82 ) |
| Angola | Male | 84.24 ( 54.1 - 112.02 ) | 4.23 ( 2.9 - 5.46 ) | 156.37 ( 122.94 - 190.84 ) | 3.36 ( 2.64 - 4.16 ) | 85.63 | -1 ( -1.09 - -0.91 ) |
| Antigua | Male | 0.89 ( 0.81 - 1 ) | 3.94 ( 3.58 - 4.37 ) | 1.51 ( 1.34 - 1.72 ) | 3.24 ( 2.87 - 3.68 ) | 69.12 | -0.74 ( -0.89 - -0.59 ) |
| Argentina | Male | 867.16 ( 828.29 - 908.3 ) | 5.78 ( 5.53 - 6.04 ) | 795.55 ( 684.81 - 923.34 ) | 3.41 ( 2.94 - 3.94 ) | -8.26 | -2.32 ( -2.57 - -2.06 ) |
| Armenia | Male | 103.3 ( 95.33 - 112.16 ) | 8.08 ( 7.47 - 8.77 ) | 90.93 ( 82.03 - 100.45 ) | 5 ( 4.53 - 5.51 ) | -11.97 | -2.34 ( -2.54 - -2.15 ) |
| Australia | Male | 217.24 ( 207.08 - 228.14 ) | 2.4 ( 2.29 - 2.52 ) | 221.25 ( 190.72 - 256.33 ) | 1.16 ( 1 - 1.34 ) | 1.85 | -3.15 ( -3.33 - -2.96 ) |
| Austria | Male | 173.6 ( 164.62 - 182.18 ) | 3.75 ( 3.56 - 3.93 ) | 120.05 ( 107.97 - 133.07 ) | 1.59 ( 1.42 - 1.76 ) | -30.85 | -3.5 ( -3.67 - -3.33 ) |
| Azerbaijan | Male | 111.42 ( 101.04 - 122.84 ) | 4.77 ( 4.35 - 5.24 ) | 177.13 ( 146.82 - 214 ) | 4.18 ( 3.43 - 4.99 ) | 58.98 | -1.58 ( -1.99 - -1.16 ) |
| Bahamas | Male | 4.03 ( 3.64 - 4.43 ) | 5.85 ( 5.31 - 6.43 ) | 8.99 ( 7.72 - 10.35 ) | 5.24 ( 4.53 - 5.99 ) | 122.78 | -0.34 ( -0.49 - -0.18 ) |
| Bahrain | Male | 3.3 ( 2.83 - 3.86 ) | 4.38 ( 3.72 - 5.11 ) | 5 ( 4.19 - 5.92 ) | 1.44 ( 1.2 - 1.71 ) | 51.39 | -5.51 ( -6.08 - -4.93 ) |
| Bangladesh | Male | 2008.34 ( 1668.28 - 2405.55 ) | 7.62 ( 6.34 - 9.22 ) | 2280.42 ( 1615.19 - 2862.66 ) | 3.55 ( 2.49 - 4.42 ) | 13.55 | -2.81 ( -2.92 - -2.7 ) |
| Barbados | Male | 3.93 ( 3.58 - 4.29 ) | 3.17 ( 2.9 - 3.45 ) | 6.34 ( 5.56 - 7.27 ) | 2.92 ( 2.56 - 3.34 ) | 61.15 | -0.37 ( -0.56 - -0.18 ) |
| Barbuda | Male | 0.89 ( 0.81 - 1 ) | 3.94 ( 3.58 - 4.37 ) | 1.51 ( 1.34 - 1.72 ) | 3.24 ( 2.87 - 3.68 ) | 69.12 | -0.74 ( -0.89 - -0.59 ) |
| Belarus | Male | 446.79 ( 425.7 - 468.02 ) | 8.41 ( 8.03 - 8.8 ) | 333.32 ( 296.27 - 375.36 ) | 5.26 ( 4.71 - 5.92 ) | -25.4 | -2.63 ( -3.16 - -2.1 ) |
| Belgium | Male | 339.7 ( 323.35 - 357.8 ) | 5.25 ( 5.01 - 5.53 ) | 186.86 ( 169.2 - 207.11 ) | 1.91 ( 1.73 - 2.12 ) | -44.99 | -4.02 ( -4.14 - -3.89 ) |
| Belize | Male | 1.14 ( 1.02 - 1.28 ) | 2.46 ( 2.19 - 2.76 ) | 4.06 ( 3.65 - 4.47 ) | 3.08 ( 2.78 - 3.38 ) | 255.84 | 0.57 ( 0.15 - 0.99 ) |
| Benin | Male | 23.74 ( 19.35 - 28.58 ) | 2.47 ( 2.01 - 2.96 ) | 51.38 ( 39.25 - 67.47 ) | 2.52 ( 1.97 - 3.28 ) | 116.43 | 0.41 ( 0.27 - 0.55 ) |
| Bermuda | Male | 1.58 ( 1.44 - 1.74 ) | 5.85 ( 5.34 - 6.42 ) | 2.27 ( 2.05 - 2.52 ) | 4.2 ( 3.79 - 4.65 ) | 43.69 | -0.98 ( -1.15 - -0.8 ) |
| Bhutan | Male | 8.34 ( 6.25 - 12.34 ) | 6.34 ( 4.74 - 9.36 ) | 11.79 ( 8.35 - 18.6 ) | 3.86 ( 2.75 - 6.04 ) | 41.39 | -1.85 ( -1.94 - -1.75 ) |
| Bolivia | Male | 49.67 ( 40.65 - 59.45 ) | 3.39 ( 2.78 - 4.03 ) | 84.83 ( 63.03 - 113.51 ) | 2.22 ( 1.65 - 2.93 ) | 70.78 | -1.58 ( -1.66 - -1.5 ) |
| Bosnia and Herzegovina | Male | 151.49 ( 140.38 - 163.37 ) | 8.1 ( 7.51 - 8.71 ) | 131.44 ( 115.4 - 148.75 ) | 4.81 ( 4.24 - 5.41 ) | -13.23 | -2.69 ( -3.01 - -2.37 ) |
| Botswana | Male | 11.64 ( 9.48 - 14.5 ) | 4.44 ( 3.64 - 5.43 ) | 14.35 ( 11.26 - 20.26 ) | 2.62 ( 2.08 - 3.57 ) | 23.22 | -2.06 ( -2.26 - -1.86 ) |
| Brazil | Male | 2118.66 ( 2067.35 - 2199.16 ) | 4.91 ( 4.79 - 5.11 ) | 4358.6 ( 4232.98 - 4494.2 ) | 4.19 ( 4.07 - 4.32 ) | 105.72 | -0.7 ( -0.78 - -0.62 ) |
| Brunei | Male | 1.72 ( 1.46 - 2 ) | 3.79 ( 3.25 - 4.41 ) | 1.82 ( 1.57 - 2.11 ) | 1.44 ( 1.23 - 1.67 ) | 5.66 | -4.24 ( -4.56 - -3.91 ) |
| Bulgaria | Male | 299.11 ( 284.39 - 314 ) | 4.91 ( 4.66 - 5.15 ) | 347.86 ( 311.86 - 384.68 ) | 5.66 ( 5.09 - 6.27 ) | 16.3 | 0.64 ( 0.37 - 0.91 ) |
| Burkina Faso | Male | 51.24 ( 37.3 - 67.33 ) | 2.5 ( 1.86 - 3.25 ) | 109.5 ( 76.05 - 137.57 ) | 2.86 ( 2 - 3.54 ) | 113.72 | 0.77 ( 0.63 - 0.9 ) |
| Burundi | Male | 54.9 ( 38.19 - 72.99 ) | 5.38 ( 3.9 - 7 ) | 63.44 ( 44.82 - 84.73 ) | 2.86 ( 2.07 - 3.75 ) | 15.56 | -2.78 ( -2.99 - -2.57 ) |
| Cambodia | Male | 85.85 ( 66.32 - 107.96 ) | 4.48 ( 3.49 - 5.75 ) | 166.87 ( 128.95 - 233.29 ) | 3.75 ( 2.94 - 5.39 ) | 94.38 | -0.58 ( -0.63 - -0.53 ) |
| Cameroon | Male | 59.82 ( 47.81 - 73.87 ) | 2.78 ( 2.24 - 3.4 ) | 164.47 ( 119.83 - 219.94 ) | 3.15 ( 2.35 - 4.27 ) | 174.93 | 0.73 ( 0.54 - 0.92 ) |
| Canada | Male | 380.83 ( 365.71 - 396.04 ) | 2.61 ( 2.5 - 2.71 ) | 403.92 ( 362.82 - 449.3 ) | 1.3 ( 1.17 - 1.45 ) | 6.06 | -2.93 ( -3.12 - -2.73 ) |
| Cape Verde | Male | 2.98 ( 2.49 - 3.48 ) | 3.2 ( 2.65 - 3.72 ) | 3.44 ( 2.97 - 3.96 ) | 1.88 ( 1.62 - 2.15 ) | 15.48 | -2.08 ( -2.24 - -1.91 ) |
| Central African Republic | Male | 26.82 ( 15.63 - 34.75 ) | 4.76 ( 3.02 - 6.02 ) | 40.33 ( 25.55 - 55.18 ) | 3.96 ( 2.82 - 5.19 ) | 50.37 | -0.84 ( -0.95 - -0.73 ) |
| Chad | Male | 28.19 ( 18.69 - 35.72 ) | 2.08 ( 1.39 - 2.61 ) | 73.74 ( 52.77 - 93.96 ) | 2.64 ( 1.92 - 3.33 ) | 161.6 | 1.28 ( 1.07 - 1.49 ) |
| Chile | Male | 115.57 ( 108.33 - 123.68 ) | 2.59 ( 2.43 - 2.77 ) | 145.44 ( 124.3 - 167.21 ) | 1.41 ( 1.21 - 1.62 ) | 25.84 | -2.27 ( -2.45 - -2.09 ) |
| China | Male | 8792.7 ( 8406.42 - 9339.56 ) | 2.18 ( 2.08 - 2.33 ) | 16109.35 ( 15256.29 - 17049.47 ) | 1.72 ( 1.63 - 1.81 ) | 83.21 | -0.98 ( -1.21 - -0.75 ) |
| Colombia | Male | 297.14 ( 283.46 - 314.07 ) | 3.6 ( 3.43 - 3.81 ) | 436.3 ( 375.01 - 505.67 ) | 1.8 ( 1.54 - 2.08 ) | 46.83 | -3.33 ( -3.62 - -3.05 ) |
| Comoros | Male | 3.75 ( 2.81 - 5.04 ) | 3.57 ( 2.73 - 4.74 ) | 5.01 ( 3.59 - 7.55 ) | 2.41 ( 1.75 - 3.7 ) | 33.84 | -1.75 ( -1.97 - -1.54 ) |
| Costa Rica | Male | 28.38 ( 26.38 - 30.41 ) | 3.4 ( 3.17 - 3.65 ) | 56.47 ( 49.04 - 63.02 ) | 2.55 ( 2.21 - 2.84 ) | 99.01 | -1.35 ( -1.61 - -1.08 ) |
| Croatia | Male | 258.23 ( 244.48 - 270.57 ) | 9.24 ( 8.76 - 9.69 ) | 173.41 ( 156.36 - 191.56 ) | 4.67 ( 4.23 - 5.15 ) | -32.85 | -2.72 ( -2.99 - -2.44 ) |
| Cuba | Male | 363.89 ( 347.43 - 382.32 ) | 7.1 ( 6.78 - 7.45 ) | 826.57 ( 703.64 - 955.94 ) | 9.34 ( 7.95 - 10.76 ) | 127.15 | 0.98 ( 0.87 - 1.1 ) |
| Cyprus | Male | 10.84 ( 9.47 - 12.4 ) | 2.77 ( 2.44 - 3.15 ) | 15.18 ( 12.99 - 17.56 ) | 1.72 ( 1.48 - 1.98 ) | 40.13 | -1.83 ( -2.17 - -1.49 ) |
| Czech Republic | Male | 323.47 ( 309.66 - 338.5 ) | 5.52 ( 5.29 - 5.77 ) | 229.46 ( 206.89 - 254.27 ) | 2.55 ( 2.31 - 2.82 ) | -29.06 | -2.83 ( -2.91 - -2.76 ) |
| Democratic Republic of the Congo | Male | 236.11 ( 176.45 - 305.17 ) | 3.25 ( 2.5 - 4.09 ) | 406.32 ( 265.28 - 533.97 ) | 2.77 ( 1.9 - 3.6 ) | 72.09 | -0.69 ( -0.79 - -0.59 ) |
| Denmark | Male | 104.76 ( 99.46 - 110.33 ) | 3 ( 2.86 - 3.16 ) | 78.64 ( 70.19 - 87.31 ) | 1.53 ( 1.38 - 1.69 ) | -24.94 | -2.86 ( -3.02 - -2.7 ) |
| Djibouti | Male | 2.82 ( 1.8 - 4.55 ) | 3.41 ( 2.25 - 5.32 ) | 7.97 ( 5.2 - 15.23 ) | 2.54 ( 1.71 - 4.79 ) | 182.3 | -1.44 ( -1.61 - -1.27 ) |
| Dominica | Male | 1.17 ( 1.05 - 1.29 ) | 3.88 ( 3.52 - 4.24 ) | 1.95 ( 1.73 - 2.2 ) | 4.34 ( 3.87 - 4.88 ) | 67.32 | 0.3 ( 0.14 - 0.45 ) |
| Dominican Republic | Male | 51.12 ( 44.47 - 59.21 ) | 2.77 ( 2.42 - 3.22 ) | 116.97 ( 92.73 - 142.57 ) | 2.69 ( 2.15 - 3.3 ) | 128.83 | 0.31 ( -0.12 - 0.73 ) |
| Ecuador | Male | 47.35 ( 44.26 - 50.9 ) | 1.84 ( 1.72 - 1.98 ) | 84.17 ( 72.67 - 96.75 ) | 1.23 ( 1.06 - 1.41 ) | 77.74 | -1.22 ( -1.57 - -0.87 ) |
| Egypt | Male | 218.93 ( 195.36 - 246.27 ) | 1.48 ( 1.33 - 1.66 ) | 434.17 ( 262.59 - 548.53 ) | 1.4 ( 0.86 - 1.76 ) | 98.31 | -0.11 ( -0.33 - 0.11 ) |
| El Salvador | Male | 25.24 ( 22.59 - 28 ) | 1.84 ( 1.66 - 2.05 ) | 44.98 ( 35.67 - 55.99 ) | 1.87 ( 1.48 - 2.33 ) | 78.23 | 0.3 ( 0.02 - 0.59 ) |
| Equatorial Guinea | Male | 4.35 ( 2.17 - 6.06 ) | 4.8 ( 2.63 - 6.45 ) | 4.72 ( 3.35 - 6.44 ) | 2.57 ( 1.87 - 3.49 ) | 8.6 | -2.82 ( -3.21 - -2.44 ) |
| Eritrea | Male | 27.97 ( 18.85 - 37.7 ) | 6.04 ( 4.35 - 7.82 ) | 39.22 ( 27.64 - 64.71 ) | 3.8 ( 2.77 - 6.14 ) | 40.24 | -2.18 ( -2.47 - -1.89 ) |
| Estonia | Male | 49.97 ( 46.57 - 53.44 ) | 6.28 ( 5.87 - 6.73 ) | 31.38 ( 26.54 - 37.73 ) | 3.23 ( 2.74 - 3.87 ) | -37.21 | -2.95 ( -3.27 - -2.62 ) |
| Ethiopia | Male | 265.61 ( 192.02 - 365.44 ) | 2.52 ( 1.89 - 3.42 ) | 295.73 ( 212.54 - 498.69 ) | 1.46 ( 1.05 - 2.48 ) | 11.34 | -2.28 ( -2.4 - -2.16 ) |
| Fiji | Male | 2.53 ( 2.15 - 3 ) | 1.6 ( 1.36 - 1.9 ) | 6.12 ( 5.11 - 7.45 ) | 2.12 ( 1.78 - 2.55 ) | 141.83 | 1.51 ( 1.27 - 1.75 ) |
| Finland | Male | 45.58 ( 42.66 - 48.59 ) | 1.61 ( 1.51 - 1.71 ) | 38.87 ( 34.83 - 43.92 ) | 0.74 ( 0.66 - 0.83 ) | -14.74 | -2.86 ( -3.01 - -2.7 ) |
| France | Male | 2926.62 ( 2811.2 - 3061.14 ) | 8.48 ( 8.15 - 8.86 ) | 1493.09 ( 1355.35 - 1646.63 ) | 2.68 ( 2.42 - 2.95 ) | -48.98 | -4.55 ( -4.97 - -4.13 ) |
| Gabon | Male | 11.74 ( 9.5 - 14.79 ) | 4.64 ( 3.79 - 5.93 ) | 16.93 ( 13.35 - 25.29 ) | 3.5 ( 2.77 - 5.42 ) | 44.14 | -1.06 ( -1.14 - -0.99 ) |
| Gambia | Male | 2.53 ( 1.9 - 3.27 ) | 4.46 ( 3.95 - 4.98 ) | 5.74 ( 4.48 - 7.58 ) | 2.52 ( 2.16 - 2.94 ) | 127.24 | 0.11 ( 0.01 - 0.2 ) |
| Georgia | Male | 191.58 ( 176.2 - 206.54 ) | 1.4 ( 1.06 - 1.79 ) | 170.22 ( 153.26 - 188.92 ) | 1.36 ( 1.07 - 1.78 ) | -11.15 | 0.24 ( -0.35 - 0.83 ) |
| Germany | Male | 1746.4 ( 1674.85 - 1821.11 ) | 7.15 ( 6.62 - 7.7 ) | 1466.45 ( 1267.8 - 1692.68 ) | 6.93 ( 6.26 - 7.65 ) | -16.03 | -2.64 ( -2.87 - -2.41 ) |
| Ghana | Male | 69.86 ( 48.75 - 89.27 ) | 3.44 ( 3.31 - 3.59 ) | 207.42 ( 129.8 - 260.15 ) | 1.83 ( 1.58 - 2.12 ) | 196.91 | 1.87 ( 1.56 - 2.18 ) |
| Greece | Male | 325.77 ( 309.16 - 343.36 ) | 2.34 ( 1.66 - 2.95 ) | 349.92 ( 316.05 - 385.37 ) | 3.21 ( 2.11 - 3.92 ) | 7.41 | -1.17 ( -1.28 - -1.06 ) |
| Greenland | Male | 0.63 ( 0.56 - 0.71 ) | 4.56 ( 4.33 - 4.8 ) | 1.1 ( 0.97 - 1.24 ) | 3.26 ( 2.94 - 3.61 ) | 74.16 | -0.98 ( -1.24 - -0.72 ) |
| Grenada | Male | 1.08 ( 0.98 - 1.19 ) | 4.24 ( 3.77 - 4.76 ) | 2.59 ( 2.31 - 2.88 ) | 3.21 ( 2.84 - 3.6 ) | 138.78 | 0.03 ( -0.28 - 0.34 ) |
| Grenadines | Male | 1.52 ( 1.37 - 1.67 ) | 5.15 ( 3.84 - 6.38 ) | 3.77 ( 3.4 - 4.2 ) | 2.64 ( 2.07 - 3.34 ) | 147.43 | 0.39 ( 0.22 - 0.56 ) |
| Guam | Male | 0.97 ( 0.83 - 1.27 ) | 3.72 ( 3.39 - 4.1 ) | 1.97 ( 1.72 - 2.26 ) | 3.5 ( 3.12 - 3.88 ) | 103.69 | -1.04 ( -1.24 - -0.84 ) |
| Guatemala | Male | 36.85 ( 34.05 - 39.92 ) | 4.69 ( 4.22 - 5.14 ) | 69.16 ( 59.98 - 79.32 ) | 5.38 ( 4.85 - 5.99 ) | 87.68 | -2.15 ( -2.46 - -1.85 ) |
| Guinea | Male | 34.48 ( 28.12 - 42.74 ) | 3.07 ( 2.65 - 3.85 ) | 77.48 ( 57.6 - 98.08 ) | 2.46 ( 2.14 - 2.81 ) | 124.72 | 1.85 ( 1.65 - 2.04 ) |
| Guinea-Bissau | Male | 7.28 ( 4.99 - 9.3 ) | 2.13 ( 1.96 - 2.31 ) | 10.45 ( 7.87 - 13.22 ) | 1.45 ( 1.26 - 1.66 ) | 43.56 | 0.03 ( -0.11 - 0.17 ) |
| Guyana | Male | 4.01 ( 3.66 - 4.39 ) | 2.08 ( 1.7 - 2.58 ) | 6.31 ( 5.26 - 7.43 ) | 2.95 ( 2.24 - 3.71 ) | 57.37 | 0.48 ( 0.22 - 0.73 ) |
| Haiti | Male | 103 ( 76.22 - 149 ) | 3.69 ( 2.62 - 4.62 ) | 169.84 ( 119.54 - 274.09 ) | 3.41 ( 2.65 - 4.22 ) | 64.9 | -0.35 ( -0.47 - -0.22 ) |
| Honduras | Male | 22.26 ( 18.67 - 26.17 ) | 2.17 ( 1.99 - 2.37 ) | 54.44 ( 41.3 - 68.4 ) | 2.16 ( 1.81 - 2.52 ) | 144.6 | -0.42 ( -0.54 - -0.3 ) |
| Hungary | Male | 572.57 ( 546.99 - 600.59 ) | 6.53 ( 4.83 - 9.65 ) | 432.22 ( 394.25 - 476.65 ) | 5.7 ( 4.05 - 9.26 ) | -24.51 | -1.84 ( -2.13 - -1.55 ) |
| Iceland | Male | 1.87 ( 1.7 - 2.05 ) | 2.1 ( 1.78 - 2.46 ) | 2.27 ( 2.07 - 2.51 ) | 1.93 ( 1.47 - 2.41 ) | 21.77 | -2.31 ( -2.8 - -1.81 ) |
| India | Male | 16980.05 ( 14385.75 - 18955.33 ) | 9 ( 8.61 - 9.43 ) | 27014.57 ( 25194.83 - 28862.7 ) | 5.67 ( 5.18 - 6.23 ) | 59.1 | -1.07 ( -1.29 - -0.84 ) |
| Indonesia | Male | 1157.92 ( 963.53 - 1640.53 ) | 1.41 ( 1.29 - 1.55 ) | 2500.9 ( 1959.13 - 4094.44 ) | 0.91 ( 0.83 - 1 ) | 115.98 | 0.42 ( 0.32 - 0.53 ) |
| Iran | Male | 524.66 ( 472.4 - 627.81 ) | 6.59 ( 5.61 - 7.35 ) | 1013.44 ( 963.93 - 1069.62 ) | 5.02 ( 4.67 - 5.36 ) | 93.16 | -0.57 ( -0.89 - -0.26 ) |
| Iraq | Male | 197.21 ( 149.21 - 243.85 ) | 2.49 ( 2.08 - 3.48 ) | 211.4 ( 189.04 - 235.74 ) | 2.69 ( 2.14 - 4.34 ) | 7.19 | -4.04 ( -4.57 - -3.5 ) |
| Ireland | Male | 50.9 ( 47.37 - 55.03 ) | 4 ( 3.63 - 4.82 ) | 53.63 ( 47.57 - 60.42 ) | 3.02 ( 2.88 - 3.19 ) | 5.37 | -1.99 ( -2.15 - -1.83 ) |
| Israel | Male | 39.48 ( 36.35 - 43.2 ) | 5.09 ( 3.88 - 6.27 ) | 77.78 ( 69.73 - 87.39 ) | 1.87 ( 1.67 - 2.08 ) | 96.99 | -1.27 ( -1.66 - -0.87 ) |
| Italy | Male | 2346.08 ( 2254.36 - 2443.33 ) | 2.72 ( 2.54 - 2.94 ) | 1433.15 ( 1296.34 - 1574.3 ) | 1.58 ( 1.41 - 1.77 ) | -38.91 | -3.59 ( -3.7 - -3.49 ) |
| Ivory Coast | Male | 62.28 ( 50.51 - 76.07 ) | 1.78 ( 1.64 - 1.95 ) | 137.81 ( 104.25 - 183.75 ) | 1.53 ( 1.37 - 1.71 ) | 121.26 | -0.37 ( -0.62 - -0.12 ) |
| Jamaica | Male | 19.36 ( 17.45 - 21.44 ) | 6.01 ( 5.78 - 6.24 ) | 47.31 ( 36.22 - 58.3 ) | 2.29 ( 2.07 - 2.51 ) | 144.4 | 0.82 ( 0.27 - 1.37 ) |
| Japan | Male | 870.89 ( 851.55 - 897.41 ) | 2.67 ( 2.2 - 3.19 ) | 1096.54 ( 1048.5 - 1144.3 ) | 2.51 ( 1.94 - 3.33 ) | 25.91 | -2.58 ( -2.7 - -2.47 ) |
| Jordan | Male | 23.88 ( 18.75 - 29.53 ) | 2.32 ( 2.1 - 2.57 ) | 32.72 ( 27.37 - 39.64 ) | 3.46 ( 2.66 - 4.27 ) | 37.02 | -4.53 ( -4.99 - -4.07 ) |
| Kazakhstan | Male | 377.38 ( 355.56 - 401.03 ) | 1.23 ( 1.21 - 1.27 ) | 219.49 ( 198.94 - 244.56 ) | 0.67 ( 0.64 - 0.69 ) | -41.84 | -3.59 ( -3.89 - -3.29 ) |
| Kenya | Male | 123.96 ( 68.94 - 156.29 ) | 3.27 ( 2.61 - 4.02 ) | 326.69 ( 225.72 - 383.39 ) | 1.17 ( 0.98 - 1.42 ) | 163.54 | 0.37 ( 0.08 - 0.65 ) |
| Kiribati | Male | 0.2 ( 0.17 - 0.23 ) | 6.96 ( 6.57 - 7.34 ) | 0.38 ( 0.31 - 0.46 ) | 3.09 ( 2.81 - 3.41 ) | 90.82 | 0.39 ( 0.1 - 0.69 ) |
| Kuwait | Male | 5.72 ( 5.2 - 6.23 ) | 2.91 ( 1.61 - 3.66 ) | 10.7 ( 9.56 - 12.09 ) | 3.17 ( 2.18 - 3.72 ) | 87.17 | -1.99 ( -2.42 - -1.57 ) |
| Kyrgyzstan | Male | 59.18 ( 53.55 - 65.48 ) | 1.4 ( 1.22 - 1.61 ) | 28.99 ( 25.71 - 32.92 ) | 1.63 ( 1.34 - 1.93 ) | -51.01 | -4.06 ( -4.44 - -3.68 ) |
| Laos | Male | 43.53 ( 32.78 - 56.37 ) | 1.58 ( 1.43 - 1.73 ) | 57.48 ( 40.88 - 99.2 ) | 0.83 ( 0.74 - 0.93 ) | 32.03 | -1.39 ( -1.43 - -1.36 ) |
| Latvia | Male | 97.12 ( 91.73 - 103.02 ) | 4.56 ( 4.15 - 5.02 ) | 74.94 ( 63.75 - 87.29 ) | 1.52 ( 1.36 - 1.7 ) | -22.84 | -1.21 ( -1.57 - -0.85 ) |
| Lebanon | Male | 80.21 ( 63.79 - 103.79 ) | 4.33 ( 3.28 - 5.63 ) | 97.28 ( 85.73 - 111.38 ) | 3.01 ( 2.16 - 5.22 ) | 21.29 | -2.82 ( -3.04 - -2.6 ) |
| Lesotho | Male | 23.82 ( 16.34 - 29.87 ) | 6.9 ( 6.52 - 7.29 ) | 27.69 ( 21.42 - 35.16 ) | 5.19 ( 4.43 - 6.04 ) | 16.24 | 0.62 ( 0.31 - 0.92 ) |
| Liberia | Male | 13.46 ( 10.53 - 17.2 ) | 7.87 ( 6.26 - 10.21 ) | 19.68 ( 14.04 - 26.07 ) | 3.73 ( 3.29 - 4.24 ) | 46.15 | 0.09 ( -0.05 - 0.23 ) |
| Libya | Male | 57.54 ( 45.11 - 73.7 ) | 5.35 ( 3.65 - 6.57 ) | 119.25 ( 80.35 - 151.19 ) | 5.75 ( 4.52 - 7.18 ) | 107.24 | -0.42 ( -0.5 - -0.35 ) |
| Lithuania | Male | 133.7 ( 126.91 - 140.57 ) | 2.2 ( 1.72 - 2.78 ) | 127.44 ( 114.76 - 141.66 ) | 2.16 ( 1.59 - 2.86 ) | -4.68 | -0.85 ( -1.21 - -0.49 ) |
| Luxembourg | Male | 11.4 ( 10.43 - 12.47 ) | 5.64 ( 4.46 - 7.15 ) | 8.42 ( 7.22 - 9.94 ) | 5.13 ( 3.49 - 6.49 ) | -26.08 | -3.76 ( -3.92 - -3.6 ) |
| Macedonia | Male | 57.04 ( 51.21 - 64.57 ) | 7.08 ( 6.72 - 7.43 ) | 96.8 ( 84.06 - 112.7 ) | 6.05 ( 5.45 - 6.7 ) | 69.72 | -0.26 ( -0.51 - -0.01 ) |
| Madagascar | Male | 80.76 ( 65.4 - 98.45 ) | 4.87 ( 4.48 - 5.31 ) | 119.31 ( 88.5 - 154.94 ) | 1.9 ( 1.63 - 2.24 ) | 47.73 | -1.07 ( -1.17 - -0.97 ) |
| Malawi | Male | 25.59 ( 14.55 - 35.72 ) | 6.12 ( 5.51 - 6.92 ) | 42.16 ( 34.38 - 51.59 ) | 6.07 ( 5.3 - 7.05 ) | 64.76 | -0.33 ( -0.64 - -0.02 ) |
| Malaysia | Male | 135.48 ( 118 - 156.39 ) | 2.91 ( 2.37 - 3.52 ) | 260.47 ( 197.98 - 316.84 ) | 2.21 ( 1.68 - 2.79 ) | 92.26 | -1.88 ( -2.14 - -1.63 ) |
| Maldives | Male | 1.54 ( 1.1 - 1.84 ) | 1.3 ( 0.78 - 1.77 ) | 2.04 ( 1.78 - 2.31 ) | 1.29 ( 1.06 - 1.58 ) | 31.93 | -3.24 ( -3.47 - -3 ) |
| Mali | Male | 38.23 ( 31.39 - 45.8 ) | 3.25 ( 2.82 - 3.73 ) | 56.85 ( 43.62 - 73.57 ) | 2.19 ( 1.71 - 2.65 ) | 48.69 | -0.96 ( -1.19 - -0.72 ) |
| Malta | Male | 7.5 ( 6.89 - 8.2 ) | 3.09 ( 2.38 - 3.62 ) | 8.17 ( 7.29 - 9.06 ) | 1.45 ( 1.26 - 1.66 ) | 8.86 | -2.91 ( -3.09 - -2.73 ) |
| Marshall Islands | Male | 0.26 ( 0.16 - 0.32 ) | 1.86 ( 1.53 - 2.21 ) | 0.5 ( 0.37 - 0.63 ) | 1.37 ( 1.06 - 1.74 ) | 91.84 | 0.12 ( -0.02 - 0.27 ) |
| Mauritania | Male | 11 ( 8.82 - 13.56 ) | 3.9 ( 3.59 - 4.24 ) | 19.01 ( 13.48 - 26.94 ) | 2.04 ( 1.82 - 2.25 ) | 72.9 | -0.18 ( -0.42 - 0.07 ) |
| Mauritius | Male | 12.6 ( 11.48 - 13.7 ) | 3.42 ( 2.17 - 4.23 ) | 16.08 ( 14.19 - 18.18 ) | 3.59 ( 2.79 - 4.39 ) | 27.62 | -2.23 ( -2.4 - -2.05 ) |
| Mexico | Male | 646.07 ( 629.51 - 674.62 ) | 2.3 ( 1.86 - 2.83 ) | 951.9 ( 869.05 - 987.7 ) | 2.02 ( 1.45 - 2.84 ) | 47.34 | -2.4 ( -2.52 - -2.27 ) |
| Micronesia | Male | 0.66 ( 0.51 - 0.91 ) | 3.93 ( 3.6 - 4.28 ) | 0.77 ( 0.61 - 0.96 ) | 2.18 ( 1.94 - 2.45 ) | 17.36 | -0.2 ( -0.23 - -0.18 ) |
| Moldova | Male | 136.32 ( 127.84 - 144.71 ) | 3.26 ( 3.18 - 3.41 ) | 151.04 ( 138.05 - 164.97 ) | 1.87 ( 1.71 - 1.94 ) | 10.8 | -0.24 ( -0.6 - 0.13 ) |
| Mongolia | Male | 7 ( 6.14 - 7.91 ) | 3.02 ( 2.35 - 4.31 ) | 20.05 ( 16.88 - 24.53 ) | 2.81 ( 2.3 - 3.47 ) | 186.21 | 3.04 ( 2.45 - 3.63 ) |
| Montenegro | Male | 23.69 ( 20.57 - 28.3 ) | 6.71 ( 6.31 - 7.12 ) | 31.33 ( 26.63 - 36.8 ) | 6.08 ( 5.58 - 6.63 ) | 32.22 | -0.85 ( -1.07 - -0.64 ) |
| Morocco | Male | 370.12 ( 309.53 - 451.87 ) | 1.77 ( 1.55 - 2 ) | 714.32 ( 546.63 - 926.46 ) | 3.19 ( 2.72 - 3.79 ) | 92.99 | -0.5 ( -0.67 - -0.34 ) |
| Mozambique | Male | 122.15 ( 86.31 - 158.26 ) | 8.02 ( 7.01 - 9.52 ) | 185.57 ( 113.39 - 242.28 ) | 6.76 ( 5.78 - 7.89 ) | 51.92 | -0.23 ( -0.32 - -0.13 ) |
| Myanmar | Male | 472.07 ( 356.52 - 610.49 ) | 5.05 ( 4.24 - 6.19 ) | 610.31 ( 486.69 - 905.16 ) | 4.44 ( 3.44 - 5.69 ) | 29.28 | -0.95 ( -1 - -0.9 ) |
| Namibia | Male | 20.56 ( 16.54 - 25.26 ) | 3.96 ( 2.85 - 5.02 ) | 30.11 ( 25.17 - 36.03 ) | 3.59 ( 2.33 - 4.58 ) | 46.47 | -0.85 ( -1.24 - -0.47 ) |
| Nepal | Male | 319.1 ( 243.12 - 412.85 ) | 4.29 ( 3.28 - 5.49 ) | 488.69 ( 373.26 - 642.16 ) | 3.24 ( 2.61 - 4.82 ) | 53.14 | -0.99 ( -1.36 - -0.63 ) |
| Netherlands | Male | 233.32 ( 221.62 - 245.31 ) | 5.96 ( 4.88 - 7.24 ) | 208.24 ( 189 - 228.07 ) | 5.01 ( 4.24 - 5.95 ) | -10.75 | -3.04 ( -3.26 - -2.82 ) |
| New Zealand | Male | 34.31 ( 31.57 - 37.24 ) | 6.22 ( 4.78 - 8.13 ) | 32.51 ( 29.25 - 36.31 ) | 4.72 ( 3.63 - 6.15 ) | -5.27 | -3.11 ( -3.26 - -2.97 ) |
| Nicaragua | Male | 16.32 ( 14.24 - 18.71 ) | 2.73 ( 2.6 - 2.87 ) | 28.73 ( 24.22 - 33.91 ) | 1.36 ( 1.24 - 1.49 ) | 76.05 | -1.93 ( -2.08 - -1.78 ) |
| Niger | Male | 30.68 ( 19.36 - 41.22 ) | 1.95 ( 1.8 - 2.11 ) | 72.18 ( 38.27 - 101.74 ) | 0.92 ( 0.83 - 1.02 ) | 135.27 | 0.22 ( 0.06 - 0.38 ) |
| Nigeria | Male | 683.94 ( 498.07 - 918.44 ) | 2.28 ( 1.98 - 2.6 ) | 900.32 ( 651.21 - 1269.48 ) | 1.42 ( 1.2 - 1.69 ) | 31.64 | -0.97 ( -1.06 - -0.88 ) |
| North Korea | Male | 148.11 ( 110.28 - 191.09 ) | 2.13 ( 1.36 - 2.76 ) | 292.02 ( 226.08 - 363.77 ) | 2.15 ( 1.18 - 2.96 ) | 97.17 | -0.02 ( -0.19 - 0.15 ) |
| Northern Mariana Islands | Male | 0.3 ( 0.24 - 0.43 ) | 2.82 ( 2.11 - 3.72 ) | 0.66 ( 0.56 - 0.79 ) | 2.28 ( 1.67 - 3.18 ) | 117.48 | -0.47 ( -0.64 - -0.3 ) |
| Norway | Male | 42.41 ( 41.24 - 43.7 ) | 2.37 ( 1.83 - 2.98 ) | 31.79 ( 30.22 - 33.82 ) | 2.31 ( 1.81 - 2.84 ) | -25.05 | -2.71 ( -2.82 - -2.6 ) |
| Oman | Male | 7.14 ( 5.4 - 9.32 ) | 3.77 ( 3.01 - 5.16 ) | 11.58 ( 8.74 - 14.94 ) | 3.19 ( 2.7 - 3.76 ) | 62.29 | -2.07 ( -2.24 - -1.9 ) |
| Pakistan | Male | 2496.79 ( 2126.1 - 2911.98 ) | 1.44 ( 1.4 - 1.48 ) | 4819.15 ( 3640.5 - 6131.8 ) | 0.74 ( 0.7 - 0.78 ) | 93.01 | -0.07 ( -0.29 - 0.14 ) |
| Palestine | Male | 9.25 ( 6.54 - 11.72 ) | 1.93 ( 1.46 - 2.47 ) | 15.14 ( 13.07 - 17.71 ) | 1.06 ( 0.81 - 1.32 ) | 63.77 | -2.18 ( -2.38 - -1.98 ) |
| Panama | Male | 29.33 ( 27.15 - 31.79 ) | 7.89 ( 6.76 - 9.15 ) | 40 ( 35.38 - 44.7 ) | 8.14 ( 6.24 - 10.28 ) | 36.38 | -2.68 ( -2.96 - -2.39 ) |
| Papua New Guinea | Male | 28.06 ( 22.04 - 35.35 ) | 2.32 ( 1.65 - 2.92 ) | 68.11 ( 52.99 - 87.8 ) | 1.37 ( 1.18 - 1.62 ) | 142.72 | 0.31 ( 0.23 - 0.39 ) |
| Paraguay | Male | 28.53 ( 24.43 - 33.92 ) | 3.92 ( 3.63 - 4.25 ) | 72.59 ( 56.89 - 91.27 ) | 2.1 ( 1.86 - 2.35 ) | 154.49 | 0.4 ( 0.25 - 0.55 ) |
| Peru | Male | 125.37 ( 108.77 - 149.94 ) | 3.01 ( 2.42 - 3.66 ) | 135.18 ( 110.07 - 163.45 ) | 3.13 ( 2.5 - 3.96 ) | 7.83 | -3.87 ( -4.2 - -3.55 ) |
| Philippines | Male | 257.26 ( 235.83 - 281.55 ) | 2.72 ( 2.34 - 3.19 ) | 600.66 ( 494.43 - 721.79 ) | 2.88 ( 2.27 - 3.6 ) | 133.48 | 0.46 ( 0.35 - 0.58 ) |
| Poland | Male | 1653.02 ( 1588.14 - 1725.29 ) | 2.23 ( 1.93 - 2.66 ) | 1487.53 ( 1354.87 - 1635.85 ) | 0.92 ( 0.75 - 1.11 ) | -10.01 | -2.28 ( -2.46 - -2.09 ) |
| Portugal | Male | 410.81 ( 390.38 - 431.4 ) | 1.73 ( 1.59 - 1.89 ) | 373.23 ( 336.41 - 413.02 ) | 1.9 ( 1.58 - 2.26 ) | -9.15 | -2.11 ( -2.3 - -1.92 ) |
| Puerto Rico | Male | 87.2 ( 81.67 - 93.11 ) | 8.47 ( 8.15 - 8.83 ) | 65.68 ( 59.04 - 73 ) | 5.01 ( 4.57 - 5.49 ) | -24.68 | -3.81 ( -4.04 - -3.58 ) |
| Qatar | Male | 1.12 ( 0.9 - 1.37 ) | 6.7 ( 6.38 - 7.03 ) | 7.21 ( 5.59 - 8.89 ) | 3.98 ( 3.56 - 4.43 ) | 541.11 | -0.69 ( -1.59 - 0.21 ) |
| Republic of Congo | Male | 24.08 ( 18.88 - 29.24 ) | 5.1 ( 4.79 - 5.45 ) | 36.58 ( 27.7 - 54.01 ) | 2.14 ( 1.93 - 2.37 ) | 51.92 | -1.84 ( -2.01 - -1.67 ) |
| Romania | Male | 799.5 ( 763.93 - 841.41 ) | 2.27 ( 1.85 - 2.75 ) | 955.47 ( 875.13 - 1050.42 ) | 1.66 ( 1.29 - 2.06 ) | 19.51 | -0.04 ( -0.26 - 0.18 ) |
| Russia | Male | 5644.4 ( 5457.23 - 5999.06 ) | 4.94 ( 4 - 5.89 ) | 4072.43 ( 3963.25 - 4188.28 ) | 3.24 ( 2.49 - 4.94 ) | -27.85 | -2.87 ( -3.29 - -2.44 ) |
| Rwanda | Male | 70.77 ( 51.28 - 88.86 ) | 5.9 ( 5.64 - 6.2 ) | 64.23 ( 49.59 - 83.39 ) | 6.15 ( 5.62 - 6.76 ) | -9.24 | -3.21 ( -3.55 - -2.88 ) |
| Saint Lucia | Male | 2.03 ( 1.84 - 2.23 ) | 7.99 ( 7.76 - 8.46 ) | 4.17 ( 3.69 - 4.68 ) | 4.45 ( 4.33 - 4.58 ) | 105.64 | -0.92 ( -1.11 - -0.73 ) |
| Saint Vincent | Male | 1.52 ( 1.37 - 1.67 ) | 5.14 ( 4.67 - 5.62 ) | 3.77 ( 3.4 - 4.2 ) | 4.16 ( 3.69 - 4.66 ) | 147.43 | 0.39 ( 0.22 - 0.56 ) |
| Samoa | Male | 0.49 ( 0.4 - 0.59 ) | 4.69 ( 4.22 - 5.14 ) | 0.65 ( 0.49 - 0.77 ) | 5.38 ( 4.85 - 5.99 ) | 33.97 | -0.53 ( -0.61 - -0.45 ) |
| Sao Tome and Principe | Male | 0.48 ( 0.41 - 0.57 ) | 1.31 ( 1.09 - 1.58 ) | 0.85 ( 0.67 - 1.06 ) | 1.16 ( 0.82 - 1.37 ) | 77.85 | 0.86 ( 0.8 - 0.91 ) |
| Saudi Arabia | Male | 63.51 ( 42.45 - 83.89 ) | 1.63 ( 1.4 - 1.92 ) | 112.13 ( 92.17 - 138.27 ) | 2.02 ( 1.59 - 2.48 ) | 76.57 | -0.89 ( -1.22 - -0.57 ) |
| Senegal | Male | 39.92 ( 32.4 - 48.62 ) | 1.82 ( 1.22 - 2.4 ) | 92.67 ( 71.72 - 118.73 ) | 1.3 ( 1.1 - 1.56 ) | 132.12 | 0.83 ( 0.6 - 1.06 ) |
| Serbia | Male | 445.51 ( 390.03 - 532.7 ) | 2.49 ( 2.03 - 3.01 ) | 384.31 ( 340.77 - 431.57 ) | 2.82 ( 2.21 - 3.55 ) | -13.74 | -1.43 ( -1.65 - -1.21 ) |
| Seychelles | Male | 3.05 ( 2.66 - 3.44 ) | 7.84 ( 6.89 - 9.35 ) | 4.57 ( 4.04 - 5.14 ) | 5.27 ( 4.69 - 5.9 ) | 49.71 | -1.3 ( -1.42 - -1.17 ) |
| Sierra Leone | Male | 23.37 ( 16.49 - 29.62 ) | 12.38 ( 10.83 - 13.88 ) | 42.21 ( 33.6 - 53.01 ) | 9.49 ( 8.43 - 10.66 ) | 80.66 | 0.71 ( 0.52 - 0.89 ) |
| Singapore | Male | 25.89 ( 23.82 - 28.09 ) | 2.35 ( 1.68 - 2.95 ) | 23.05 ( 20.31 - 25.79 ) | 2.6 ( 2.09 - 3.2 ) | -10.96 | -4.71 ( -4.95 - -4.46 ) |
| Slovakia | Male | 215.19 ( 199.96 - 228.81 ) | 2.63 ( 2.42 - 2.84 ) | 162.19 ( 141.88 - 188.28 ) | 0.78 ( 0.69 - 0.88 ) | -24.63 | -2.66 ( -2.78 - -2.54 ) |
| Slovenia | Male | 65.61 ( 61.62 - 69.98 ) | 8.17 ( 7.62 - 8.68 ) | 46.1 ( 40.76 - 51.93 ) | 4.05 ( 3.56 - 4.66 ) | -29.74 | -3.69 ( -3.83 - -3.55 ) |
| Solomon Islands | Male | 1.94 ( 1.5 - 2.42 ) | 6.34 ( 5.96 - 6.75 ) | 3.86 ( 3.06 - 4.89 ) | 2.53 ( 2.25 - 2.84 ) | 99.18 | 0.13 ( 0.05 - 0.22 ) |
| Somalia | Male | 49.23 ( 26.64 - 75.5 ) | 2.63 ( 2.08 - 3.21 ) | 92.38 ( 67.61 - 122.85 ) | 2.6 ( 2.06 - 3.36 ) | 87.63 | -1.24 ( -1.47 - -1.01 ) |
| South Africa | Male | 366.87 ( 315.57 - 485.68 ) | 3.62 ( 2.18 - 5.19 ) | 572.41 ( 535.92 - 624.68 ) | 2.95 ( 2.22 - 3.84 ) | 56.03 | -1.21 ( -1.85 - -0.56 ) |
| South Korea | Male | 558.89 ( 531.11 - 590.03 ) | 3.98 ( 3.42 - 5.31 ) | 389.26 ( 346.35 - 436.95 ) | 3.18 ( 2.99 - 3.44 ) | -30.35 | -6.67 ( -7.36 - -5.97 ) |
| South Sudan | Male | 46.07 ( 25.57 - 69.19 ) | 4.62 ( 4.39 - 4.87 ) | 55.31 ( 40.1 - 76.4 ) | 1.13 ( 1 - 1.26 ) | 20.06 | -1.2 ( -1.39 - -1.01 ) |
| Spain | Male | 2036.72 ( 1958.35 - 2126.4 ) | 3.29 ( 1.93 - 4.76 ) | 1399.55 ( 1277.74 - 1537.71 ) | 2.65 ( 1.96 - 3.58 ) | -31.28 | -3.71 ( -3.88 - -3.55 ) |
| Sri Lanka | Male | 78.6 ( 70.6 - 88.08 ) | 8.35 ( 8.03 - 8.7 ) | 157.27 ( 126.26 - 193.4 ) | 3.41 ( 3.12 - 3.73 ) | 100.1 | 0.82 ( 0.41 - 1.24 ) |
| Sudan | Male | 168.33 ( 121.27 - 226.21 ) | 1.46 ( 1.32 - 1.63 ) | 279.67 ( 201.72 - 389.99 ) | 1.45 ( 1.17 - 1.76 ) | 66.15 | -0.47 ( -0.51 - -0.43 ) |
| Suriname | Male | 1.98 ( 1.76 - 2.21 ) | 3.48 ( 2.54 - 4.66 ) | 5.21 ( 4.41 - 6.14 ) | 3.01 ( 2.18 - 4.2 ) | 163.33 | 0.72 ( 0.5 - 0.94 ) |
| Swaziland | Male | 7.52 ( 5.85 - 9.27 ) | 1.62 ( 1.45 - 1.81 ) | 11.49 ( 8.1 - 14.51 ) | 1.95 ( 1.66 - 2.27 ) | 52.85 | -0.17 ( -0.66 - 0.31 ) |
| Sweden | Male | 70.13 ( 66.04 - 74.49 ) | 5.63 ( 4.45 - 6.89 ) | 56.05 ( 51.07 - 61.4 ) | 4.89 ( 3.5 - 6.11 ) | -20.07 | -2.16 ( -2.23 - -2.08 ) |
| Switzerland | Male | 113.58 ( 107.28 - 120.02 ) | 1.04 ( 0.99 - 1.11 ) | 78.48 ( 69.47 - 89.02 ) | 0.58 ( 0.53 - 0.63 ) | -30.91 | -3.09 ( -3.24 - -2.94 ) |
| Syria | Male | 44.34 ( 37.72 - 52.03 ) | 2.57 ( 2.43 - 2.71 ) | 83.28 ( 65.5 - 103.97 ) | 1.03 ( 0.92 - 1.17 ) | 87.83 | -1.1 ( -1.43 - -0.77 ) |
| Tajikistan | Male | 27.59 ( 24.89 - 30.63 ) | 2.1 ( 2.01 - 2.2 ) | 24.26 ( 20.48 - 28.47 ) | 1.49 ( 1.35 - 1.64 ) | -12.05 | -2.8 ( -3.11 - -2.5 ) |
| Tanzania | Male | 174.44 ( 118.06 - 247.54 ) | 2.1 ( 1.9 - 2.33 ) | 271.91 ( 206.89 - 396.88 ) | 1.03 ( 0.87 - 1.21 ) | 55.88 | -1.39 ( -1.57 - -1.21 ) |
| Thailand | Male | 721.82 ( 638.78 - 805.25 ) | 3.1 ( 2.18 - 4.34 ) | 1119.46 ( 955.68 - 1315.91 ) | 2.33 ( 1.78 - 3.35 ) | 55.09 | -2.61 ( -2.84 - -2.37 ) |
| Timor-Leste | Male | 3.58 ( 2.7 - 5.42 ) | 2.58 ( 1.99 - 3.82 ) | 10.42 ( 7.41 - 17.37 ) | 2.73 ( 1.98 - 4.51 ) | 190.83 | 0.37 ( 0.18 - 0.55 ) |
| Tobago | Male | 12.04 ( 11.03 - 13.08 ) | 3.06 ( 2.81 - 3.32 ) | 18.97 ( 14.9 - 23.89 ) | 2.3 ( 1.8 - 2.89 ) | 57.64 | -1.32 ( -1.58 - -1.07 ) |
| Togo | Male | 13.67 ( 10.93 - 16.12 ) | 2.37 ( 1.92 - 2.78 ) | 39.19 ( 29.15 - 49.98 ) | 2.79 ( 2.14 - 3.47 ) | 186.76 | 0.96 ( 0.79 - 1.13 ) |
| Tonga | Male | 0.48 ( 0.4 - 0.64 ) | 1.99 ( 1.65 - 2.64 ) | 0.74 ( 0.59 - 0.97 ) | 2.11 ( 1.69 - 2.77 ) | 53.12 | 0.33 ( 0.27 - 0.39 ) |
| Trinidad | Male | 12.04 ( 11.03 - 13.08 ) | 3.06 ( 2.81 - 3.32 ) | 18.97 ( 14.9 - 23.89 ) | 2.3 ( 1.8 - 2.89 ) | 57.64 | -1.32 ( -1.58 - -1.07 ) |
| Tunisia | Male | 135.01 ( 115.38 - 163.69 ) | 5.17 ( 4.46 - 6.27 ) | 252.8 ( 189.59 - 323.28 ) | 4.31 ( 3.26 - 5.47 ) | 87.25 | -0.95 ( -1.09 - -0.8 ) |
| Turkey | Male | 1124.65 ( 963.56 - 1365.06 ) | 6.57 ( 5.63 - 8.04 ) | 1343.76 ( 1157.35 - 1539.74 ) | 3.48 ( 3 - 3.98 ) | 19.48 | -2.65 ( -2.89 - -2.41 ) |
| Turkmenistan | Male | 39.72 ( 36.67 - 42.99 ) | 4.56 ( 4.22 - 4.94 ) | 26.12 ( 22.11 - 29.58 ) | 1.53 ( 1.31 - 1.73 ) | -34.23 | -4.74 ( -5.1 - -4.37 ) |
| Uganda | Male | 76.98 ( 57.73 - 99.61 ) | 2.29 ( 1.74 - 2.94 ) | 147.19 ( 112.61 - 205.26 ) | 2.4 ( 1.85 - 3.3 ) | 91.21 | -0.32 ( -0.71 - 0.07 ) |
| UK | Male | 766.09 ( 749.96 - 782.79 ) | 1.99 ( 1.95 - 2.03 ) | 714.15 ( 694.13 - 735.32 ) | 1.25 ( 1.21 - 1.28 ) | -6.78 | -2.05 ( -2.18 - -1.92 ) |
| Ukraine | Male | 2392.45 ( 2273.28 - 2508.07 ) | 8.27 ( 7.89 - 8.64 ) | 1751.34 ( 1603.17 - 1898.25 ) | 5.84 ( 5.35 - 6.32 ) | -26.8 | -2.24 ( -2.65 - -1.83 ) |
| United Arab Emirates | Male | 12.44 ( 8.92 - 17.79 ) | 3.91 ( 2.94 - 5.06 ) | 114.78 ( 76.26 - 159.62 ) | 3.82 ( 2.23 - 5.34 ) | 822.76 | 0.05 ( -0.07 - 0.17 ) |
| Uruguay | Male | 159.27 ( 150.26 - 168.65 ) | 9.02 ( 8.52 - 9.53 ) | 109.05 ( 93.17 - 126.56 ) | 4.89 ( 4.15 - 5.66 ) | -31.53 | -2.28 ( -2.42 - -2.14 ) |
| USA | Male | 3700.49 ( 3640.66 - 3802.71 ) | 2.7 ( 2.66 - 2.78 ) | 4300.61 ( 4147.46 - 4446.67 ) | 1.73 ( 1.67 - 1.79 ) | 16.22 | -2.05 ( -2.19 - -1.92 ) |
| Uzbekistan | Male | 167.07 ( 157.77 - 176.64 ) | 3.21 ( 3.03 - 3.4 ) | 229.48 ( 195.44 - 268.25 ) | 2.47 ( 2.12 - 2.85 ) | 37.36 | -0.64 ( -1.02 - -0.26 ) |
| Vanuatu | Male | 1.1 ( 0.8 - 1.87 ) | 3.2 ( 2.37 - 5.34 ) | 2.88 ( 1.94 - 6.04 ) | 3.59 ( 2.47 - 7.34 ) | 161.42 | 0.53 ( 0.46 - 0.61 ) |
| Venezuela | Male | 192.86 ( 181.86 - 205.48 ) | 4.33 ( 4.08 - 4.62 ) | 486.58 ( 396.6 - 588.17 ) | 3.82 ( 3.13 - 4.63 ) | 152.3 | -0.67 ( -0.8 - -0.54 ) |
| Vietnam | Male | 552.15 ( 454.84 - 653.57 ) | 3.21 ( 2.66 - 3.79 ) | 1301.34 ( 1052.34 - 1617.98 ) | 3.23 ( 2.61 - 3.97 ) | 135.69 | 0.05 ( -0.1 - 0.2 ) |
| Virgin Islands | Male | 1.74 ( 1.55 - 1.96 ) | 4.45 ( 3.99 - 4.98 ) | 4.2 ( 3.27 - 5.02 ) | 4.94 ( 3.85 - 5.85 ) | 141.81 | 0.61 ( 0.5 - 0.73 ) |
| Yemen | Male | 98.71 ( 54.91 - 138.67 ) | 3.95 ( 2.3 - 5.59 ) | 230.71 ( 169.81 - 296.25 ) | 3.84 ( 2.87 - 4.92 ) | 133.73 | -0.1 ( -0.15 - -0.05 ) |
| Zambia | Male | 63.36 ( 48.25 - 88.68 ) | 4.03 ( 3.16 - 5.59 ) | 91.65 ( 70.39 - 145.04 ) | 2.92 ( 2.25 - 4.65 ) | 44.65 | -1.79 ( -2.09 - -1.48 ) |
| Zimbabwe | Male | 86.04 ( 71.77 - 101.53 ) | 4.11 ( 3.45 - 4.83 ) | 139.24 ( 107.69 - 172.48 ) | 4.59 ( 3.61 - 5.64 ) | 61.83 | 0.82 ( 0.15 - 1.5 ) |

ASDR, age standardized death rate; CI, confidence interval; EAPC, estimated annual percentage change; UI, uncertainty interval.
