# Supplementary material for: Global burden of larynx cancer, 1990-2017: estimates from the global burden of disease 2017 study
Source: Aging (Albany NY). 2020 Feb 8;12(3):2545–83. doi: 10.18632/aging.102762 (PMC7041735; doi:10.18632/aging.102762)
Supplement: Supplementary Table 5 [file aging-12-102762-s004..docx]

**Supplementary Table 5. The DALYs of larynx cancer in 1990 and 2017 among 195vcountries and territories, and its temporal trends from 1990 to 2017.**

| **Nation** | **Sex** | **1990** | | **2017** | | **1990-2017** | |
| --- | --- | --- | --- | --- | --- | --- | --- |
|  |  | **DALY No. (95% UI)** | **Age standardized DALY rate (per 100,000) No. (95% UI)** | **DALY No. (95% UI)** | **Age standardized DALY rate (per 100,000) No. (95% UI)** | **Change in**  **absolute number**  **of DALYs**  **No. (%)** | **EAPC No. (95% CI)** |
| Afghanistan | Both | 8041.03 ( 4230.15 - 10977.39 ) | 104.62 ( 55.87 - 143.75 ) | 12648.83 ( 9916.54 - 15880.6 ) | 95.28 ( 75.38 - 119.39 ) | 57.3 | -0.39 ( -0.65 - -0.14 ) |
| Albania | Both | 1748.09 ( 1613.98 - 1904.58 ) | 75.24 ( 69.34 - 81.63 ) | 2034.2 ( 1587.3 - 2613.45 ) | 49.34 ( 38.56 - 63.12 ) | 16.37 | -1.41 ( -1.66 - -1.15 ) |
| Algeria | Both | 7150.84 ( 6167.39 - 8140.04 ) | 51.52 ( 44.45 - 58.58 ) | 11520.26 ( 9991.39 - 13086.41 ) | 32.6 ( 28.31 - 37.02 ) | 61.1 | -1.61 ( -1.67 - -1.55 ) |
| American Samoa | Both | 8.32 ( 7.18 - 10.23 ) | 32.32 ( 27.69 - 40.66 ) | 12.09 ( 10.37 - 14.02 ) | 25.91 ( 22.03 - 29.9 ) | 45.29 | -0.69 ( -0.93 - -0.44 ) |
| Andorra | Both | 15.44 ( 11.5 - 21.04 ) | 25.61 ( 19.04 - 34.94 ) | 20.81 ( 16.61 - 26.48 ) | 15.87 ( 12.68 - 20.22 ) | 34.76 | -1.97 ( -2.04 - -1.91 ) |
| Angola | Both | 2833.55 ( 1836.04 - 3760.42 ) | 60.68 ( 41.11 - 79.32 ) | 5099.9 ( 4096.88 - 6167.47 ) | 41.27 ( 33.16 - 49.68 ) | 79.98 | -1.61 ( -1.72 - -1.51 ) |
| Antigua | Both | 21.47 ( 19.5 - 23.82 ) | 43.07 ( 38.98 - 47.95 ) | 37.73 ( 33.13 - 42.94 ) | 36.08 ( 31.77 - 41.01 ) | 75.76 | -0.72 ( -0.82 - -0.61 ) |
| Argentina | Both | 25224.56 ( 24014.95 - 26425.91 ) | 75.67 ( 72.13 - 79.34 ) | 21501.52 ( 18755.87 - 24751.32 ) | 41.76 ( 36.43 - 48.17 ) | -14.76 | -2.63 ( -2.9 - -2.37 ) |
| Armenia | Both | 3191.78 ( 2945.71 - 3471.99 ) | 101.43 ( 93.71 - 109.86 ) | 2530.76 ( 2300.51 - 2783.55 ) | 59.82 ( 54.58 - 65.61 ) | -20.71 | -2.46 ( -2.7 - -2.22 ) |
| Australia | Both | 6049.58 ( 5771.57 - 6342.93 ) | 30.87 ( 29.46 - 32.43 ) | 5468.21 ( 4780.12 - 6292.96 ) | 14.46 ( 12.62 - 16.65 ) | -9.61 | -3.21 ( -3.41 - -3.01 ) |
| Austria | Both | 4876.12 ( 4617.8 - 5134.39 ) | 46.28 ( 43.82 - 48.89 ) | 3247.31 ( 2926.98 - 3600.89 ) | 21.28 ( 19.11 - 23.58 ) | -33.4 | -3.22 ( -3.36 - -3.08 ) |
| Azerbaijan | Both | 4094.95 ( 3769.51 - 4459.83 ) | 70.22 ( 64.66 - 76.26 ) | 6248.07 ( 5329.36 - 7331.02 ) | 57.74 ( 49.43 - 67.41 ) | 52.58 | -1.69 ( -2.08 - -1.29 ) |
| Bahamas | Both | 121.93 ( 110.23 - 133.55 ) | 72.2 ( 65.25 - 79.16 ) | 263.22 ( 227.37 - 301.31 ) | 64.13 ( 55.55 - 73.12 ) | 115.89 | -0.34 ( -0.49 - -0.18 ) |
| Bahrain | Both | 80.51 ( 69.42 - 92.36 ) | 46.53 ( 40.04 - 53.66 ) | 133.91 ( 113.22 - 156.46 ) | 14.88 ( 12.59 - 17.43 ) | 66.32 | -5.72 ( -6.32 - -5.12 ) |
| Bangladesh | Both | 72649.02 ( 61579.04 - 85799.45 ) | 136.09 ( 116.4 - 160.18 ) | 81172.66 ( 64739.64 - 98432.5 ) | 61.85 ( 48.63 - 75.12 ) | 11.73 | -2.75 ( -2.86 - -2.65 ) |
| Barbados | Both | 98.14 ( 90.87 - 105.98 ) | 36.13 ( 33.39 - 39.07 ) | 161.58 ( 141.58 - 184.55 ) | 34.24 ( 30.01 - 39.02 ) | 64.64 | -0.25 ( -0.38 - -0.12 ) |
| Barbuda | Both | 21.47 ( 19.5 - 23.82 ) | 43.07 ( 38.98 - 47.95 ) | 37.73 ( 33.13 - 42.94 ) | 36.08 ( 31.77 - 41.01 ) | 75.76 | -0.72 ( -0.82 - -0.61 ) |
| Belarus | Both | 13689.31 ( 13036.87 - 14354.44 ) | 103.83 ( 98.8 - 108.83 ) | 9421.07 ( 8383.73 - 10636.47 ) | 60.85 ( 54.11 - 68.83 ) | -31.18 | -2.97 ( -3.53 - -2.41 ) |
| Belgium | Both | 9559.74 ( 9085.69 - 10047 ) | 68.12 ( 64.65 - 71.83 ) | 5112.95 ( 4652.08 - 5654.84 ) | 26.71 ( 24.25 - 29.7 ) | -46.52 | -3.83 ( -3.98 - -3.69 ) |
| Belize | Both | 31.87 ( 28.8 - 35.24 ) | 33.07 ( 29.83 - 36.58 ) | 120.16 ( 108.48 - 131.85 ) | 42.35 ( 38.23 - 46.36 ) | 277.01 | 0.76 ( 0.34 - 1.18 ) |
| Benin | Both | 667.17 ( 539.16 - 799.35 ) | 31.39 ( 25.57 - 37.51 ) | 1511.11 ( 1154.14 - 1967.4 ) | 30.24 ( 23.36 - 39.2 ) | 126.49 | 0.12 ( 0.02 - 0.23 ) |
| Bermuda | Both | 42.56 ( 38.95 - 46.34 ) | 65.28 ( 59.79 - 71.04 ) | 52.18 ( 47.05 - 57.68 ) | 43.07 ( 38.71 - 47.68 ) | 22.6 | -1.32 ( -1.47 - -1.16 ) |
| Bhutan | Both | 332.87 ( 263.98 - 449.55 ) | 112.25 ( 88.83 - 153.06 ) | 392.65 ( 293 - 566.72 ) | 59.61 ( 44.39 - 87.03 ) | 17.96 | -2.46 ( -2.57 - -2.36 ) |
| Bolivia | Both | 1811.87 ( 1526.21 - 2105.82 ) | 50.52 ( 42.66 - 58.54 ) | 2532.67 ( 1959.01 - 3266.86 ) | 28.39 ( 22.07 - 36.45 ) | 39.78 | -2.28 ( -2.38 - -2.17 ) |
| Bosnia and Herzegovina | Both | 4665.23 ( 4334.11 - 5022.58 ) | 99.21 ( 92.42 - 106.28 ) | 3419.45 ( 3024.77 - 3857.75 ) | 56.42 ( 49.95 - 63.48 ) | -26.7 | -2.95 ( -3.31 - -2.58 ) |
| Botswana | Both | 365.9 ( 294.36 - 451.54 ) | 56.12 ( 45.77 - 69.12 ) | 455.14 ( 365.11 - 618.68 ) | 30.83 ( 24.97 - 41.68 ) | 24.39 | -2.24 ( -2.36 - -2.12 ) |
| Brazil | Both | 67867.28 ( 66246.63 - 70030.73 ) | 67.76 ( 66.14 - 69.9 ) | 130280.65 ( 126595.65 - 134485.79 ) | 54.94 ( 53.4 - 56.67 ) | 91.96 | -0.91 ( -1.01 - -0.8 ) |
| Brunei | Both | 58.18 ( 52.27 - 65.21 ) | 55.01 ( 48.9 - 61.94 ) | 58.52 ( 51.67 - 66.27 ) | 18.08 ( 15.91 - 20.52 ) | 0.59 | -4.88 ( -5.26 - -4.49 ) |
| Bulgaria | Both | 9068.63 ( 8604.2 - 9562.99 ) | 71.71 ( 67.96 - 75.81 ) | 9697.95 ( 8743.95 - 10716.52 ) | 78.07 ( 70.11 - 86.59 ) | 6.94 | 0.26 ( 0 - 0.52 ) |
| Burkina Faso | Both | 1594.59 ( 1197.5 - 2032.92 ) | 33.18 ( 25.09 - 41.96 ) | 3350.42 ( 2374.42 - 4133.58 ) | 34.93 ( 24.94 - 43.05 ) | 110.11 | 0.37 ( 0.26 - 0.47 ) |
| Burundi | Both | 2214.17 ( 1656.14 - 2797.2 ) | 84.95 ( 63.91 - 106.74 ) | 2354.59 ( 1733.56 - 3078.93 ) | 46.51 ( 34.47 - 60.1 ) | 6.34 | -2.75 ( -2.99 - -2.51 ) |
| Cambodia | Both | 3391.06 ( 2790.08 - 4056.11 ) | 65.68 ( 54.25 - 78.67 ) | 5480.27 ( 4399.81 - 7062.68 ) | 44.49 ( 36.07 - 56.97 ) | 61.61 | -1.48 ( -1.53 - -1.42 ) |
| Cameroon | Both | 1856.95 ( 1491.5 - 2254.46 ) | 36.85 ( 29.81 - 44.7 ) | 4874.09 ( 3616.41 - 6436.4 ) | 38.86 ( 28.99 - 51.01 ) | 162.48 | 0.42 ( 0.22 - 0.63 ) |
| Canada | Both | 11029.3 ( 10597.94 - 11493.32 ) | 34.2 ( 32.88 - 35.65 ) | 10675.79 ( 9622.68 - 11755.38 ) | 16.85 ( 15.19 - 18.52 ) | -3.21 | -3.01 ( -3.25 - -2.77 ) |
| Cape Verde | Both | 71.2 ( 59.86 - 83.21 ) | 31.12 ( 26.1 - 36.51 ) | 91.05 ( 78.82 - 103.87 ) | 20.45 ( 17.65 - 23.32 ) | 27.89 | -1.65 ( -1.79 - -1.51 ) |
| Central African Republic | Both | 951.43 ( 591.8 - 1223.45 ) | 68.9 ( 44.28 - 87.15 ) | 1430.34 ( 935.27 - 1920.99 ) | 55.9 ( 38.05 - 74.41 ) | 50.34 | -0.94 ( -1.04 - -0.84 ) |
| Chad | Both | 805.83 ( 562.56 - 1001.13 ) | 26.95 ( 18.82 - 33.42 ) | 2124.3 ( 1546.81 - 2679.09 ) | 36.64 ( 26.87 - 46.13 ) | 163.62 | 1.53 ( 1.32 - 1.75 ) |
| Chile | Both | 3170.18 ( 2979.15 - 3373.11 ) | 30.23 ( 28.41 - 32.13 ) | 3548.44 ( 3067.26 - 4055.6 ) | 15.19 ( 13.13 - 17.35 ) | 11.93 | -2.48 ( -2.68 - -2.28 ) |
| China | Both | 308695.84 ( 296000.75 - 325055.62 ) | 32.39 ( 31.08 - 34.14 ) | 465228.27 ( 442883.16 - 489143 ) | 22.89 ( 21.8 - 24.03 ) | 50.71 | -1.46 ( -1.74 - -1.17 ) |
| Colombia | Both | 9799.48 ( 9439.73 - 10250.37 ) | 51.36 ( 49.4 - 53.78 ) | 11185.77 ( 9709.43 - 12740.56 ) | 20.73 ( 18.02 - 23.64 ) | 14.15 | -4.15 ( -4.42 - -3.87 ) |
| Comoros | Both | 141.35 ( 108.65 - 182.04 ) | 59.61 ( 46.24 - 76.8 ) | 176.28 ( 131.79 - 240.31 ) | 35.11 ( 26.37 - 48.24 ) | 24.72 | -2.26 ( -2.47 - -2.06 ) |
| Costa Rica | Both | 761.73 ( 713.84 - 813.29 ) | 41.48 ( 38.82 - 44.28 ) | 1333.47 ( 1166.09 - 1481.01 ) | 26.85 ( 23.59 - 29.8 ) | 75.06 | -1.96 ( -2.21 - -1.7 ) |
| Croatia | Both | 7483.3 ( 7083.5 - 7860.69 ) | 111.05 ( 105.37 - 116.66 ) | 4316.22 ( 3871.66 - 4766 ) | 54.82 ( 49.18 - 60.93 ) | -42.32 | -2.87 ( -3.18 - -2.56 ) |
| Cuba | Both | 9774 ( 9357.62 - 10212.03 ) | 93.34 ( 89.36 - 97.62 ) | 21903.13 ( 18867.67 - 25090.74 ) | 119.06 ( 102.44 - 136 ) | 124.1 | 0.98 ( 0.88 - 1.07 ) |
| Cyprus | Both | 279.51 ( 247.8 - 317.88 ) | 33.22 ( 29.5 - 37.69 ) | 359.05 ( 307.29 - 414.4 ) | 19.38 ( 16.58 - 22.29 ) | 28.45 | -2.17 ( -2.46 - -1.88 ) |
| Czech Republic | Both | 9673.6 ( 9240.09 - 10111.68 ) | 73.12 ( 69.63 - 76.41 ) | 6255.12 ( 5670.63 - 6892.94 ) | 34.05 ( 30.78 - 37.62 ) | -35.34 | -2.81 ( -2.88 - -2.74 ) |
| Democratic Republic of the Congo | Both | 7749.44 ( 5923.83 - 9910.3 ) | 42.89 ( 33.41 - 54.11 ) | 13793.67 ( 9234.85 - 17964.63 ) | 35.34 ( 24.45 - 45.16 ) | 78 | -0.8 ( -0.91 - -0.7 ) |
| Denmark | Both | 3167.69 ( 3013.45 - 3335.22 ) | 43.5 ( 41.33 - 45.93 ) | 2169.64 ( 1962.42 - 2385.02 ) | 21.39 ( 19.39 - 23.58 ) | -31.51 | -2.98 ( -3.15 - -2.81 ) |
| Djibouti | Both | 111.73 ( 72.04 - 173.01 ) | 56.48 ( 37.61 - 85.5 ) | 278.95 ( 182.43 - 495.19 ) | 40.63 ( 27.16 - 71.74 ) | 149.66 | -1.6 ( -1.8 - -1.4 ) |
| Dominica | Both | 31.88 ( 29.38 - 34.6 ) | 46.48 ( 42.87 - 50.34 ) | 51.13 ( 45.61 - 57.15 ) | 57.31 ( 51.1 - 63.78 ) | 60.42 | 0.73 ( 0.61 - 0.85 ) |
| Dominican Republic | Both | 1684.79 ( 1504.53 - 1870.36 ) | 40.21 ( 35.9 - 44.85 ) | 3450.78 ( 2835.32 - 4100.48 ) | 36.58 ( 30.01 - 43.5 ) | 104.82 | -0.05 ( -0.48 - 0.37 ) |
| Ecuador | Both | 1545.32 ( 1462.64 - 1638.4 ) | 26.29 ( 24.82 - 27.89 ) | 2420.55 ( 2148.05 - 2739.75 ) | 16.11 ( 14.31 - 18.25 ) | 56.64 | -1.64 ( -1.94 - -1.33 ) |
| Egypt | Both | 8415.69 ( 7595.85 - 9390.77 ) | 24.08 ( 21.85 - 26.83 ) | 15689.39 ( 10535.56 - 19250.61 ) | 21.83 ( 14.41 - 26.67 ) | 86.43 | -0.16 ( -0.37 - 0.04 ) |
| El Salvador | Both | 704.54 ( 637.12 - 780.16 ) | 21.92 ( 19.78 - 24.32 ) | 1172.11 ( 946.14 - 1443.75 ) | 20.63 ( 16.65 - 25.43 ) | 66.37 | -0.07 ( -0.31 - 0.18 ) |
| Equatorial Guinea | Both | 149.16 ( 82.77 - 206.43 ) | 65.69 ( 37.78 - 89.39 ) | 150.2 ( 102.5 - 209.97 ) | 29.29 ( 20.26 - 40.29 ) | 0.7 | -3.52 ( -3.97 - -3.07 ) |
| Eritrea | Both | 1158.77 ( 796.96 - 1534.99 ) | 91.51 ( 66.34 - 119.16 ) | 1624.28 ( 1210.64 - 2501.52 ) | 53.96 ( 41.2 - 82.63 ) | 40.17 | -2.48 ( -2.73 - -2.22 ) |
| Estonia | Both | 1481.39 ( 1380.94 - 1588.49 ) | 71.79 ( 66.97 - 76.98 ) | 822.39 ( 698.38 - 986.88 ) | 36.99 ( 31.32 - 44.51 ) | -44.49 | -3.16 ( -3.52 - -2.8 ) |
| Ethiopia | Both | 10474.06 ( 7937.59 - 13609.71 ) | 43.12 ( 32.93 - 55.93 ) | 10007.65 ( 7717.03 - 15482.2 ) | 22.26 ( 17.04 - 34.98 ) | -4.45 | -2.8 ( -2.92 - -2.67 ) |
| Fiji | Both | 94.34 ( 81.54 - 108.62 ) | 22.31 ( 19.36 - 25.64 ) | 208.53 ( 178.25 - 244.61 ) | 26.3 ( 22.64 - 30.66 ) | 121.03 | 1.07 ( 0.85 - 1.28 ) |
| Finland | Both | 1296.01 ( 1216.99 - 1374.7 ) | 18.83 ( 17.7 - 20.01 ) | 994.49 ( 893.07 - 1115.28 ) | 9.58 ( 8.59 - 10.81 ) | -23.27 | -2.45 ( -2.57 - -2.33 ) |
| France | Both | 82155.02 ( 78792.3 - 86092.36 ) | 111.92 ( 107.17 - 117.41 ) | 40188.32 ( 36570.72 - 44388.9 ) | 36.76 ( 33.31 - 40.5 ) | -51.08 | -4.4 ( -4.8 - -4.01 ) |
| Gabon | Both | 358.34 ( 293.08 - 439.89 ) | 58.31 ( 47.94 - 71.23 ) | 505.57 ( 401.8 - 724.51 ) | 44.08 ( 35.37 - 63.63 ) | 41.09 | -1.02 ( -1.12 - -0.91 ) |
| Gambia | Both | 79.13 ( 61.27 - 100.73 ) | 57.09 ( 51.47 - 63.01 ) | 171.81 ( 136.83 - 221.75 ) | 30.83 ( 26.43 - 35.99 ) | 117.12 | -0.33 ( -0.4 - -0.26 ) |
| Georgia | Both | 6008.39 ( 5565.99 - 6453.15 ) | 19.77 ( 15.41 - 25.11 ) | 4832.79 ( 4363.9 - 5330.43 ) | 17.34 ( 13.87 - 22.47 ) | -19.57 | 0.05 ( -0.48 - 0.58 ) |
| Germany | Both | 52394.15 ( 50084.74 - 54739.93 ) | 91.16 ( 84.71 - 98.17 ) | 38986.59 ( 33916.91 - 45101.28 ) | 87.02 ( 78.48 - 95.95 ) | -25.59 | -2.59 ( -2.79 - -2.4 ) |
| Ghana | Both | 2174.55 ( 1600.88 - 2734.4 ) | 45.53 ( 43.49 - 47.69 ) | 6086.05 ( 3876.17 - 7601.88 ) | 25.05 ( 21.73 - 28.98 ) | 179.88 | 1.22 ( 0.98 - 1.46 ) |
| Greece | Both | 7545.71 ( 7175.82 - 7970.15 ) | 30.37 ( 22.47 - 37.96 ) | 7591.83 ( 6878.2 - 8381.36 ) | 36.19 ( 23.45 - 44.83 ) | 0.61 | -0.82 ( -0.94 - -0.7 ) |
| Greenland | Both | 18.42 ( 16.33 - 20.76 ) | 48.87 ( 46.49 - 51.65 ) | 28.2 ( 24.89 - 31.96 ) | 38.16 ( 34.4 - 42.2 ) | 53.09 | -0.61 ( -0.85 - -0.38 ) |
| Grenada | Both | 31.1 ( 28.53 - 33.95 ) | 46.75 ( 41.79 - 52.27 ) | 66.47 ( 59.4 - 73.31 ) | 38.41 ( 34.01 - 43.24 ) | 113.72 | 0.49 ( 0.16 - 0.83 ) |
| Grenadines | Both | 40.47 ( 36.78 - 44.1 ) | 79.36 ( 63.35 - 96.1 ) | 98.36 ( 88.01 - 109.78 ) | 34.16 ( 27.8 - 42.63 ) | 143.05 | 0.87 ( 0.7 - 1.04 ) |
| Guam | Both | 27.96 ( 23.99 - 36.26 ) | 46.16 ( 42.33 - 50.14 ) | 60.26 ( 53.1 - 68.18 ) | 48.53 ( 43.27 - 53.48 ) | 115.55 | 0.09 ( -0.07 - 0.25 ) |
| Guatemala | Both | 1256.88 ( 1179.8 - 1338.73 ) | 55.47 ( 50.23 - 60.64 ) | 2099.93 ( 1859.59 - 2360.73 ) | 71.65 ( 64.14 - 79.92 ) | 67.07 | -2.52 ( -2.88 - -2.16 ) |
| Guinea | Both | 988.34 ( 822.26 - 1184.85 ) | 31.73 ( 27.47 - 40.77 ) | 2168.91 ( 1629.28 - 2753.39 ) | 32.6 ( 28.86 - 36.82 ) | 119.45 | 1.74 ( 1.54 - 1.94 ) |
| Guinea-Bissau | Both | 224.88 ( 157.16 - 282.54 ) | 29.82 ( 28.03 - 31.73 ) | 334.34 ( 253.51 - 420.57 ) | 17.98 ( 15.94 - 20.16 ) | 48.68 | -0.31 ( -0.44 - -0.17 ) |
| Guyana | Both | 124.73 ( 114.78 - 136.39 ) | 27.89 ( 23.21 - 33.53 ) | 196.93 ( 166.36 - 229.68 ) | 38.38 ( 29.04 - 48.44 ) | 57.89 | 0.4 ( 0.13 - 0.67 ) |
| Haiti | Both | 3371.51 ( 2566.22 - 4523.65 ) | 49.96 ( 35.52 - 62.4 ) | 5185.9 ( 3816.1 - 7690 ) | 42.46 ( 33 - 52.7 ) | 53.82 | -0.87 ( -0.98 - -0.76 ) |
| Honduras | Both | 688.96 ( 577.61 - 808.1 ) | 29.62 ( 27.23 - 32.39 ) | 1455.93 ( 1115.87 - 1855 ) | 29.04 ( 24.65 - 33.65 ) | 111.32 | -0.85 ( -0.96 - -0.74 ) |
| Hungary | Both | 17959.05 ( 17157.85 - 18814.71 ) | 92.89 ( 70.98 - 126.14 ) | 13082.49 ( 12014.64 - 14313.23 ) | 71.26 ( 52.65 - 106.97 ) | -27.15 | -1.91 ( -2.24 - -1.58 ) |
| Iceland | Both | 55.53 ( 51.31 - 60.56 ) | 28 ( 23.64 - 32.76 ) | 59.81 ( 54.23 - 65.86 ) | 22.96 ( 17.74 - 28.89 ) | 7.71 | -2.55 ( -2.97 - -2.14 ) |
| India | Both | 637603.97 ( 560723.15 - 697886.78 ) | 126.74 ( 120.99 - 133.23 ) | 963096.98 ( 913023.7 - 1016639.14 ) | 79.71 ( 72.83 - 87.42 ) | 51.05 | -1.38 ( -1.63 - -1.14 ) |
| Indonesia | Both | 44397.54 ( 38307.28 - 58717.83 ) | 20.5 ( 18.88 - 22.37 ) | 77043.57 ( 62576.83 - 118987.32 ) | 12.12 ( 11.01 - 13.35 ) | 73.53 | -0.46 ( -0.55 - -0.38 ) |
| Iran | Both | 19468.27 ( 18031.67 - 22182.65 ) | 112.62 ( 98.96 - 123.32 ) | 31922.85 ( 30618.04 - 33541.02 ) | 80.79 ( 76.46 - 85.34 ) | 63.97 | -1.12 ( -1.38 - -0.86 ) |
| Iraq | Both | 7571.53 ( 6134.15 - 9091.85 ) | 38.86 ( 33.65 - 51.31 ) | 7611.16 ( 6894.31 - 8391.93 ) | 33.6 ( 27.48 - 51.61 ) | 0.52 | -4.38 ( -5.02 - -3.75 ) |
| Ireland | Both | 1457.01 ( 1363.53 - 1564.26 ) | 63.47 ( 58.91 - 72.48 ) | 1431.18 ( 1276.9 - 1600.82 ) | 42.98 ( 41.26 - 45.1 ) | -1.77 | -2.14 ( -2.28 - -2 ) |
| Israel | Both | 987.81 ( 918.61 - 1073.1 ) | 86.94 ( 70.21 - 103.67 ) | 1849.15 ( 1667.65 - 2052.64 ) | 29.42 ( 26.8 - 32.36 ) | 87.2 | -1.25 ( -1.63 - -0.87 ) |
| Italy | Both | 60331.65 ( 58002.65 - 62866.55 ) | 36.52 ( 34.18 - 39.13 ) | 31864.47 ( 29087.2 - 34957.2 ) | 20.62 ( 18.34 - 23.06 ) | -47.18 | -3.64 ( -3.76 - -3.52 ) |
| Ivory Coast | Both | 1941.07 ( 1578.34 - 2379.11 ) | 20.53 ( 19.09 - 22.34 ) | 4228.08 ( 3181.35 - 5656.3 ) | 17.54 ( 15.77 - 19.45 ) | 117.82 | -0.51 ( -0.74 - -0.28 ) |
| Jamaica | Both | 492.52 ( 450.49 - 541.53 ) | 70.06 ( 67.32 - 73.02 ) | 1247.34 ( 960.02 - 1538.73 ) | 26.17 ( 23.81 - 28.79 ) | 153.26 | 1.01 ( 0.45 - 1.57 ) |
| Japan | Both | 22223.56 ( 21449.87 - 23107.31 ) | 38.61 ( 31.61 - 46.66 ) | 21987.82 ( 20793.56 - 23263.2 ) | 35.03 ( 26.66 - 46.56 ) | -1.06 | -2.33 ( -2.41 - -2.25 ) |
| Jordan | Both | 809.42 ( 659.33 - 972.88 ) | 27.99 ( 25.62 - 30.75 ) | 964.98 ( 827.96 - 1152.33 ) | 43.34 ( 33.46 - 53.43 ) | 19.22 | -5.28 ( -5.86 - -4.69 ) |
| Kazakhstan | Both | 12671.28 ( 11971.11 - 13399.71 ) | 12.61 ( 12.18 - 13.11 ) | 6726.76 ( 6121.55 - 7468.67 ) | 7.14 ( 6.75 - 7.56 ) | -46.91 | -3.98 ( -4.31 - -3.65 ) |
| Kenya | Both | 4196.84 ( 2647.33 - 5172.63 ) | 48.32 ( 39.32 - 58.43 ) | 10954.67 ( 7953.17 - 12707.14 ) | 14.62 ( 12.48 - 17.5 ) | 161.02 | 0.05 ( -0.24 - 0.34 ) |
| Kiribati | Both | 10.35 ( 9.21 - 11.51 ) | 87.57 ( 82.98 - 92.37 ) | 18.88 ( 15.94 - 22.01 ) | 36.32 ( 33.17 - 40.11 ) | 82.4 | -0.17 ( -0.41 - 0.06 ) |
| Kuwait | Both | 198.33 ( 182.14 - 215.45 ) | 43.89 ( 27.25 - 54.04 ) | 289.64 ( 259.39 - 325.73 ) | 43.97 ( 31.85 - 51.03 ) | 46.04 | -2.68 ( -3.06 - -2.3 ) |
| Kyrgyzstan | Both | 1887.7 ( 1721.71 - 2071.43 ) | 24.67 ( 22.27 - 27.26 ) | 934.82 ( 838.61 - 1054.05 ) | 24.46 ( 20.85 - 28.17 ) | -50.48 | -4.21 ( -4.54 - -3.89 ) |
| Laos | Both | 1698.06 ( 1370.85 - 2096.12 ) | 25.09 ( 23.02 - 27.25 ) | 1897.18 ( 1413.35 - 2948.88 ) | 10.91 ( 9.77 - 12.23 ) | 11.73 | -2.15 ( -2.19 - -2.1 ) |
| Latvia | Both | 2893.92 ( 2730.01 - 3077.13 ) | 57.5 ( 52.3 - 63.15 ) | 1973.63 ( 1683.06 - 2315.78 ) | 19.13 ( 17.27 - 21.47 ) | -31.8 | -1.46 ( -1.86 - -1.05 ) |
| Lebanon | Both | 2616.42 ( 2146.03 - 3262.42 ) | 71.29 ( 57.32 - 87.97 ) | 3164.98 ( 2795.51 - 3580.84 ) | 41.06 ( 30.58 - 65.02 ) | 20.97 | -2.95 ( -3.22 - -2.67 ) |
| Lesotho | Both | 769.43 ( 554.08 - 944.31 ) | 79.36 ( 74.76 - 84.39 ) | 929.33 ( 727.95 - 1164.06 ) | 58.26 ( 49.59 - 68.54 ) | 20.78 | 0.46 ( 0.1 - 0.82 ) |
| Liberia | Both | 378.1 ( 302.17 - 471.96 ) | 105.55 ( 86.62 - 132.34 ) | 590.38 ( 438.47 - 762.09 ) | 49.81 ( 44.09 - 56.24 ) | 56.14 | -0.35 ( -0.51 - -0.19 ) |
| Libya | Both | 1775.56 ( 1400.05 - 2250.42 ) | 70.59 ( 50.67 - 86.43 ) | 3879.07 ( 2759.52 - 4877.65 ) | 71.08 ( 56.07 - 88.96 ) | 118.47 | -0.61 ( -0.7 - -0.53 ) |
| Lithuania | Both | 4037.19 ( 3821.7 - 4246.84 ) | 31.66 ( 25.34 - 39.39 ) | 3467.92 ( 3130.44 - 3858.18 ) | 28.25 ( 21.04 - 36.67 ) | -14.1 | -1.15 ( -1.55 - -0.74 ) |
| Luxembourg | Both | 329.17 ( 302.18 - 360.8 ) | 82.88 ( 65.95 - 104.55 ) | 244.09 ( 210.92 - 283.16 ) | 72.55 ( 51.31 - 90.56 ) | -25.85 | -3.33 ( -3.48 - -3.19 ) |
| Macedonia | Both | 1725.47 ( 1553.12 - 1942.36 ) | 87.64 ( 82.83 - 92.3 ) | 2676.06 ( 2329.53 - 3106.8 ) | 71.4 ( 64.13 - 79.35 ) | 55.09 | -0.41 ( -0.66 - -0.15 ) |
| Madagascar | Both | 3103.78 ( 2600.72 - 3702.03 ) | 62.27 ( 57.13 - 68.22 ) | 4659.04 ( 3592.21 - 5905.82 ) | 27.1 ( 23.44 - 31.46 ) | 50.11 | -1.48 ( -1.59 - -1.37 ) |
| Malawi | Both | 1079.81 ( 585.02 - 1442.72 ) | 83.72 ( 75.61 - 94.06 ) | 1598.12 ( 1331.75 - 1917.78 ) | 80.44 ( 70.08 - 93.43 ) | 48 | -1.2 ( -1.59 - -0.81 ) |
| Malaysia | Both | 4134.04 ( 3679.28 - 4703.62 ) | 51.37 ( 43.19 - 61.08 ) | 7458.42 ( 5935.27 - 8850.61 ) | 35.68 ( 27.94 - 44.76 ) | 80.41 | -1.8 ( -2.11 - -1.49 ) |
| Maldives | Both | 44.55 ( 31.48 - 53.24 ) | 23.2 ( 13.36 - 30.63 ) | 51.16 ( 44.89 - 58.23 ) | 19.49 ( 16.35 - 23.35 ) | 14.83 | -4.02 ( -4.22 - -3.82 ) |
| Mali | Both | 1438.28 ( 1239.71 - 1672.22 ) | 41.34 ( 36.79 - 46.97 ) | 1973.46 ( 1572.11 - 2462.94 ) | 27.75 ( 22.11 - 32.84 ) | 37.21 | -1.29 ( -1.51 - -1.07 ) |
| Malta | Both | 201.68 ( 186.09 - 219.33 ) | 44.32 ( 32.82 - 52.11 ) | 211.26 ( 190.14 - 234.05 ) | 17.28 ( 15.04 - 19.75 ) | 4.75 | -2.48 ( -2.65 - -2.31 ) |
| Marshall Islands | Both | 9.08 ( 6.58 - 10.84 ) | 30.87 ( 26.49 - 35.86 ) | 19.1 ( 15.48 - 23.27 ) | 21.29 ( 17.05 - 26.45 ) | 110.34 | 0.21 ( 0 - 0.41 ) |
| Mauritania | Both | 331.13 ( 268 - 399.14 ) | 46.16 ( 42.61 - 50.16 ) | 512.82 ( 370.97 - 712.65 ) | 26.74 ( 24.02 - 29.7 ) | 54.87 | -0.55 ( -0.75 - -0.35 ) |
| Mauritius | Both | 394.31 ( 364.02 - 423.75 ) | 47.65 ( 34.46 - 56.8 ) | 443.76 ( 392.48 - 498.91 ) | 50.99 ( 41.79 - 61.56 ) | 12.54 | -2.76 ( -2.92 - -2.59 ) |
| Mexico | Both | 18076.81 ( 17660.07 - 18734.34 ) | 30.57 ( 24.84 - 36.91 ) | 23878.42 ( 22063.82 - 24725.99 ) | 24.68 ( 18 - 34.18 ) | 32.09 | -2.79 ( -2.94 - -2.64 ) |
| Micronesia | Both | 23.31 ( 19.27 - 28.96 ) | 48.93 ( 45.38 - 52.48 ) | 29.98 ( 23.39 - 36.69 ) | 25.27 ( 22.53 - 28.28 ) | 28.59 | -0.52 ( -0.57 - -0.48 ) |
| Moldova | Both | 4371.8 ( 4096.32 - 4649.52 ) | 39.4 ( 38.48 - 40.89 ) | 4539.88 ( 4151.08 - 4966.01 ) | 20.6 ( 19.03 - 21.32 ) | 3.84 | -0.41 ( -0.78 - -0.03 ) |
| Mongolia | Both | 217.7 ( 196.34 - 241.71 ) | 43.44 ( 35.93 - 54.93 ) | 539.04 ( 460.31 - 648.51 ) | 37.74 ( 30.41 - 45.61 ) | 147.61 | 1.29 ( 0.82 - 1.76 ) |
| Montenegro | Both | 832.46 ( 730.54 - 960.43 ) | 91.33 ( 85.66 - 97.22 ) | 1015.13 ( 885.77 - 1176.53 ) | 79.74 ( 72.96 - 87.45 ) | 21.94 | -0.8 ( -1.09 - -0.51 ) |
| Morocco | Both | 11249.13 ( 9559.5 - 13290.4 ) | 20.4 ( 18.43 - 22.57 ) | 20720.45 ( 16105.3 - 26462.26 ) | 26.01 ( 22.25 - 30.74 ) | 84.2 | -0.71 ( -0.81 - -0.61 ) |
| Mozambique | Both | 4505.65 ( 3263.96 - 5754.65 ) | 123.09 ( 108.38 - 141.27 ) | 6974.26 ( 4591.13 - 8918.18 ) | 104.22 ( 91.02 - 120.82 ) | 54.79 | -0.47 ( -0.58 - -0.37 ) |
| Myanmar | Both | 19960.42 ( 15792.53 - 24439.75 ) | 71.97 ( 61.1 - 85.6 ) | 20479.06 ( 16714.54 - 27895.3 ) | 60.39 ( 47.09 - 77.05 ) | 2.6 | -2.18 ( -2.26 - -2.09 ) |
| Namibia | Both | 752.1 ( 612.35 - 911.76 ) | 61.21 ( 44.45 - 77.43 ) | 1010.21 ( 838.31 - 1214.69 ) | 52.28 ( 34.83 - 66.27 ) | 34.32 | -1.68 ( -2.19 - -1.15 ) |
| Nepal | Both | 13761.32 ( 11006.05 - 16856.67 ) | 75.98 ( 60.38 - 92.71 ) | 17908.29 ( 14393.78 - 21738.22 ) | 42.56 ( 35.06 - 57.97 ) | 30.13 | -1.72 ( -2.08 - -1.37 ) |
| Netherlands | Both | 6193.3 ( 5879.77 - 6501 ) | 95.01 ( 77.95 - 114.55 ) | 5624.56 ( 5142.18 - 6144.07 ) | 66.41 ( 55.37 - 79.33 ) | -9.18 | -2.56 ( -2.8 - -2.32 ) |
| New Zealand | Both | 946.04 ( 880.84 - 1014.66 ) | 122.15 ( 97.6 - 149.98 ) | 828.48 ( 750.49 - 916.03 ) | 76.95 ( 61.77 - 92.92 ) | -12.43 | -3.02 ( -3.18 - -2.86 ) |
| Nicaragua | Both | 518.99 ( 462.27 - 580.86 ) | 32.25 ( 30.55 - 33.92 ) | 780.63 ( 658.12 - 918.69 ) | 18.19 ( 16.66 - 19.86 ) | 50.42 | -2.37 ( -2.56 - -2.18 ) |
| Niger | Both | 961 ( 631.58 - 1257.49 ) | 24.1 ( 22.39 - 25.82 ) | 2193.56 ( 1232.84 - 3042.84 ) | 11.66 ( 10.58 - 12.85 ) | 128.26 | -0.26 ( -0.41 - -0.1 ) |
| Nigeria | Both | 19795.97 ( 14175.74 - 26917.49 ) | 30.22 ( 27.03 - 33.94 ) | 25899.32 ( 18345.77 - 37116.11 ) | 16.84 ( 14.24 - 19.81 ) | 30.83 | -1.61 ( -1.73 - -1.49 ) |
| North Korea | Both | 5292.34 ( 4101.42 - 6661.72 ) | 29.62 ( 19.52 - 38.67 ) | 9634.72 ( 7645.78 - 12047.96 ) | 27.05 ( 15.44 - 37.05 ) | 82.05 | 0.27 ( 0.16 - 0.38 ) |
| Northern Mariana Islands | Both | 9.97 ( 7.89 - 13.88 ) | 39.95 ( 28.76 - 54.34 ) | 20.73 ( 17.7 - 23.81 ) | 27.47 ( 19.69 - 38.85 ) | 108.03 | -0.36 ( -0.56 - -0.16 ) |
| Norway | Both | 1124.87 ( 1087.37 - 1162.76 ) | 28.01 ( 22.07 - 34.77 ) | 851.7 ( 806.65 - 904.6 ) | 29.4 ( 23.49 - 36.53 ) | -24.29 | -2.54 ( -2.66 - -2.42 ) |
| Oman | Both | 234.58 ( 181.99 - 303.38 ) | 45.74 ( 37.07 - 62.26 ) | 385.3 ( 296.03 - 492.18 ) | 37.77 ( 32.64 - 43.35 ) | 64.25 | -2.27 ( -2.4 - -2.14 ) |
| Pakistan | Both | 87793.36 ( 76636.9 - 99420.89 ) | 18.5 ( 17.88 - 19.13 ) | 174319.52 ( 136681.33 - 216341.64 ) | 9.94 ( 9.44 - 10.54 ) | 98.56 | -0.45 ( -0.71 - -0.19 ) |
| Palestine | Both | 265.39 ( 195.84 - 330.5 ) | 29.21 ( 22.6 - 37.18 ) | 463.36 ( 408.3 - 530.55 ) | 15.96 ( 12.27 - 20.15 ) | 74.59 | -2.05 ( -2.23 - -1.87 ) |
| Panama | Both | 795.17 ( 737.93 - 857.86 ) | 141.42 ( 123.62 - 160.86 ) | 986.49 ( 880.24 - 1095.97 ) | 134.13 ( 106.1 - 164.78 ) | 24.06 | -3 ( -3.32 - -2.68 ) |
| Papua New Guinea | Both | 1218.06 ( 988.83 - 1489.45 ) | 28.59 ( 21.11 - 35.57 ) | 2887.62 ( 2337.13 - 3589.94 ) | 17.34 ( 15.26 - 19.95 ) | 137.07 | 0.18 ( 0.07 - 0.29 ) |
| Paraguay | Both | 799.19 ( 685.27 - 956.04 ) | 50.23 ( 46.64 - 54.18 ) | 1986.25 ( 1569.53 - 2480.73 ) | 24.87 ( 22.19 - 27.68 ) | 148.53 | 0.33 ( 0.17 - 0.49 ) |
| Peru | Both | 4111.46 ( 3658.65 - 4712.55 ) | 53.48 ( 44.7 - 64.17 ) | 3770.24 ( 3176.16 - 4375.26 ) | 53.14 ( 44.25 - 64.46 ) | -8.3 | -4.05 ( -4.4 - -3.7 ) |
| Philippines | Both | 10072.35 ( 9323.96 - 10883.35 ) | 33.63 ( 28.91 - 40.04 ) | 19722.54 ( 16774.06 - 23198.87 ) | 35.67 ( 28.32 - 44.26 ) | 95.81 | -0.43 ( -0.57 - -0.29 ) |
| Poland | Both | 50994.78 ( 49016.56 - 53156.68 ) | 31 ( 27.67 - 35.7 ) | 41706.99 ( 37985.59 - 45730.13 ) | 12.28 ( 10.35 - 14.27 ) | -18.21 | -2.48 ( -2.66 - -2.3 ) |
| Portugal | Both | 11312.99 ( 10786.16 - 11887.45 ) | 28.26 ( 26.24 - 30.44 ) | 9554.79 ( 8513.19 - 10681.75 ) | 25.12 ( 21.45 - 29.37 ) | -15.54 | -1.98 ( -2.23 - -1.72 ) |
| Puerto Rico | Both | 2219.18 ( 2083.59 - 2370.59 ) | 113.83 ( 109.35 - 118.71 ) | 1552.35 ( 1403.94 - 1716.95 ) | 64.37 ( 58.77 - 70.71 ) | -30.05 | -3.83 ( -4.06 - -3.6 ) |
| Qatar | Both | 35.54 ( 28.4 - 43.03 ) | 84.02 ( 79.98 - 88.51 ) | 231.89 ( 181.86 - 281.63 ) | 50.98 ( 45.15 - 57.08 ) | 552.42 | -0.23 ( -1.13 - 0.68 ) |
| Republic of Congo | Both | 818.6 ( 640.58 - 991.55 ) | 60.1 ( 56.44 - 64.29 ) | 1230.98 ( 953.13 - 1695.7 ) | 24.84 ( 22.5 - 27.53 ) | 50.38 | -1.86 ( -2.05 - -1.67 ) |
| Romania | Both | 25325.19 ( 24153.22 - 26683.24 ) | 27.88 ( 22.59 - 33.79 ) | 26999.91 ( 24690.65 - 29636.02 ) | 22.75 ( 17.89 - 28.2 ) | 6.61 | -0.38 ( -0.62 - -0.14 ) |
| Russia | Both | 174387.54 ( 167726.19 - 185861.27 ) | 66.24 ( 52.99 - 79.39 ) | 116573.37 ( 113187.62 - 119850.22 ) | 43.51 ( 34.2 - 60.55 ) | -33.15 | -3 ( -3.46 - -2.54 ) |
| Rwanda | Both | 2698.36 ( 2150.84 - 3281.87 ) | 86.93 ( 82.99 - 91.65 ) | 2276.66 ( 1847.37 - 2865.96 ) | 84.39 ( 76.85 - 92.55 ) | -15.63 | -4 ( -4.39 - -3.61 ) |
| Saint Lucia | Both | 54.9 ( 50.36 - 59.77 ) | 91.84 ( 88.38 - 98.37 ) | 113.07 ( 100.01 - 126.71 ) | 51.27 ( 49.76 - 52.76 ) | 105.95 | -0.65 ( -0.82 - -0.49 ) |
| Saint Vincent | Both | 40.47 ( 36.78 - 44.1 ) | 60.56 ( 55.46 - 65.97 ) | 98.36 ( 88.01 - 109.78 ) | 52.91 ( 46.93 - 59.1 ) | 143.05 | 0.87 ( 0.7 - 1.04 ) |
| Samoa | Both | 14.03 ( 11.69 - 16.76 ) | 55.47 ( 50.23 - 60.64 ) | 17.91 ( 14.61 - 21.05 ) | 71.65 ( 64.14 - 79.92 ) | 27.61 | -0.86 ( -0.97 - -0.76 ) |
| Sao Tome and Principe | Both | 12.59 ( 10.82 - 14.89 ) | 15.88 ( 13.34 - 19.03 ) | 23.54 ( 18.7 - 29.22 ) | 12.8 ( 10.29 - 14.87 ) | 86.93 | 0.79 ( 0.71 - 0.86 ) |
| Saudi Arabia | Both | 1871.6 ( 1290.34 - 2458.23 ) | 17.9 ( 15.42 - 21.15 ) | 3721.17 ( 3024.76 - 4627.86 ) | 22.05 ( 17.42 - 27.13 ) | 98.82 | -1.2 ( -1.54 - -0.85 ) |
| Senegal | Both | 1137.72 ( 937.78 - 1373.56 ) | 26.75 ( 18.52 - 34.91 ) | 2557.98 ( 2004.58 - 3284.73 ) | 18.09 ( 15.15 - 21.9 ) | 124.84 | 0.45 ( 0.24 - 0.66 ) |
| Serbia | Both | 13143.01 ( 11493.2 - 15572.59 ) | 32.36 ( 26.7 - 39 ) | 10408.83 ( 9309.17 - 11741.8 ) | 33.43 ( 26.37 - 42.73 ) | -20.8 | -1.45 ( -1.7 - -1.19 ) |
| Seychelles | Both | 85.44 ( 73.98 - 96.08 ) | 103.58 ( 91.22 - 122.56 ) | 130.99 ( 115.66 - 146.95 ) | 70.13 ( 62.29 - 79.44 ) | 53.32 | -1.27 ( -1.37 - -1.17 ) |
| Sierra Leone | Both | 661.44 ( 482.38 - 830.87 ) | 149.29 ( 128.13 - 168.1 ) | 1264.92 ( 1012.33 - 1564.45 ) | 114.52 ( 101.5 - 128.04 ) | 91.24 | 0.54 ( 0.39 - 0.7 ) |
| Singapore | Both | 701.77 ( 650.43 - 756.51 ) | 32.26 ( 23.56 - 40.33 ) | 586.25 ( 522.7 - 654.18 ) | 34.47 ( 27.75 - 42.7 ) | -16.46 | -4.72 ( -4.89 - -4.55 ) |
| Slovakia | Both | 6490.82 ( 6039.5 - 6925.17 ) | 28.98 ( 26.88 - 31.24 ) | 4539.48 ( 3959.76 - 5245.12 ) | 8.43 ( 7.52 - 9.36 ) | -30.06 | -2.91 ( -3.04 - -2.79 ) |
| Slovenia | Both | 1916.94 ( 1795.83 - 2064.63 ) | 110.48 ( 102.46 - 118.23 ) | 1189.5 ( 1061.49 - 1330.19 ) | 51.66 ( 45 - 59.62 ) | -37.95 | -3.61 ( -3.77 - -3.46 ) |
| Solomon Islands | Both | 73.8 ( 59.54 - 88.99 ) | 75.76 ( 70.89 - 81.42 ) | 146.44 ( 121.38 - 175.32 ) | 31.68 ( 28.24 - 35.46 ) | 98.42 | -0.25 ( -0.34 - -0.16 ) |
| Somalia | Both | 1962.77 ( 1006.14 - 3029.31 ) | 44.79 ( 36.73 - 53.81 ) | 3455.8 ( 2489.95 - 4570.69 ) | 39.93 ( 33.37 - 48.05 ) | 76.07 | -1.51 ( -1.76 - -1.26 ) |
| South Africa | Both | 12505 ( 10987.41 - 15543.47 ) | 59.75 ( 34.03 - 88.11 ) | 17487.14 ( 16394.41 - 19011.16 ) | 46.07 ( 33.59 - 59.64 ) | 39.84 | -1.78 ( -2.46 - -1.1 ) |
| South Korea | Both | 18106.61 ( 17312.57 - 19067.81 ) | 53.47 ( 46.82 - 67.4 ) | 9244.75 ( 8242.09 - 10384.99 ) | 37.05 ( 34.79 - 40.21 ) | -48.94 | -7.39 ( -8 - -6.77 ) |
| South Sudan | Both | 1575.56 ( 870.29 - 2431.58 ) | 53.97 ( 51.65 - 56.76 ) | 1975.72 ( 1406.15 - 2764.97 ) | 10.75 ( 9.61 - 12.05 ) | 25.4 | -1.47 ( -1.68 - -1.26 ) |
| Spain | Both | 54340.27 ( 52146 - 56759.38 ) | 57.18 ( 32.58 - 85.52 ) | 32803.64 ( 29979.03 - 36038.21 ) | 43.55 ( 31.62 - 59.82 ) | -39.63 | -3.96 ( -4.16 - -3.76 ) |
| Sri Lanka | Both | 2370.44 ( 2129.08 - 2636.49 ) | 104.66 ( 100.37 - 109.52 ) | 4842.42 ( 3963.32 - 5831 ) | 40.47 ( 36.91 - 44.52 ) | 104.28 | 0.75 ( 0.38 - 1.13 ) |
| Sudan | Both | 6060.51 ( 4651 - 7757.7 ) | 19.74 ( 17.82 - 21.92 ) | 9309.95 ( 7051.64 - 12096.89 ) | 18.72 ( 15.39 - 22.52 ) | 53.62 | -0.77 ( -0.83 - -0.7 ) |
| Suriname | Both | 61.41 ( 55.12 - 68.28 ) | 57.95 ( 44.74 - 73.81 ) | 152.41 ( 130.93 - 177.54 ) | 46.63 ( 35.08 - 61.47 ) | 148.2 | 0.42 ( 0.19 - 0.64 ) |
| Swaziland | Both | 252.65 ( 202.17 - 310.82 ) | 22.21 ( 19.94 - 24.68 ) | 383.09 ( 280.25 - 483.89 ) | 24.8 ( 21.36 - 28.82 ) | 51.63 | -0.32 ( -0.88 - 0.23 ) |
| Sweden | Both | 1835.46 ( 1740.31 - 1940.03 ) | 74.11 ( 59.16 - 90.62 ) | 1403.03 ( 1289.51 - 1526.18 ) | 60.18 ( 44.38 - 75.52 ) | -23.56 | -2.1 ( -2.15 - -2.04 ) |
| Switzerland | Both | 3170.4 ( 2991.34 - 3354.7 ) | 13.47 ( 12.76 - 14.26 ) | 2033.32 ( 1811.76 - 2287.43 ) | 7.73 ( 7.11 - 8.41 ) | -35.87 | -3.17 ( -3.3 - -3.05 ) |
| Syria | Both | 1456.86 ( 1261.26 - 1677.81 ) | 33.05 ( 31.14 - 35 ) | 2473.21 ( 1990.42 - 3057.53 ) | 13.54 ( 12.04 - 15.29 ) | 69.76 | -1.6 ( -1.97 - -1.23 ) |
| Tajikistan | Both | 993.91 ( 909.77 - 1095.21 ) | 29.11 ( 27.78 - 30.46 ) | 1145.06 ( 989.42 - 1296.91 ) | 18.17 ( 16.45 - 20.01 ) | 15.21 | -2.27 ( -2.5 - -2.03 ) |
| Tanzania | Both | 6335.43 ( 4373.71 - 8711.68 ) | 31.63 ( 28.88 - 34.84 ) | 9369.34 ( 7406.46 - 13091.46 ) | 18.23 ( 15.87 - 20.61 ) | 47.89 | -1.76 ( -1.98 - -1.55 ) |
| Thailand | Both | 23119.89 ( 20848.06 - 25545.62 ) | 50.31 ( 35.42 - 68.35 ) | 31060.64 ( 26507.31 - 36280.55 ) | 34.63 ( 27.52 - 48.56 ) | 34.35 | -2.8 ( -3.05 - -2.56 ) |
| Timor-Leste | Both | 152.57 ( 122.38 - 208.55 ) | 43.48 ( 35.21 - 59.86 ) | 301.66 ( 218.93 - 470.57 ) | 35.98 ( 26.5 - 55.95 ) | 97.71 | -0.69 ( -0.87 - -0.52 ) |
| Tobago | Both | 334.95 ( 309.78 - 361.12 ) | 37.64 ( 34.79 - 40.58 ) | 486.13 ( 381.68 - 616.39 ) | 26.75 ( 20.95 - 33.9 ) | 45.13 | -1.61 ( -1.86 - -1.36 ) |
| Togo | Both | 431.75 ( 352.78 - 504.51 ) | 30.73 ( 25.18 - 35.89 ) | 1230.66 ( 923.66 - 1580.86 ) | 31.39 ( 23.85 - 39.38 ) | 185.04 | 0.31 ( 0.2 - 0.43 ) |
| Tonga | Both | 14.59 ( 12.55 - 18.43 ) | 24.87 ( 21.52 - 31.54 ) | 21.4 ( 17.65 - 27.08 ) | 25.96 ( 21.4 - 32.73 ) | 46.68 | 0.31 ( 0.22 - 0.39 ) |
| Trinidad | Both | 334.95 ( 309.78 - 361.12 ) | 37.64 ( 34.79 - 40.58 ) | 486.13 ( 381.68 - 616.39 ) | 26.75 ( 20.95 - 33.9 ) | 45.13 | -1.61 ( -1.86 - -1.36 ) |
| Tunisia | Both | 3591.35 ( 3084.61 - 4295.78 ) | 65.44 ( 56.5 - 78.01 ) | 6541.83 ( 4902.52 - 8361.75 ) | 51.46 ( 38.75 - 65.42 ) | 82.16 | -1.18 ( -1.34 - -1.02 ) |
| Turkey | Both | 33864.61 ( 29372.05 - 40045.44 ) | 86.2 ( 75.11 - 102.06 ) | 35132.04 ( 30591.28 - 39891.99 ) | 39.56 ( 34.48 - 44.91 ) | 3.74 | -3.26 ( -3.56 - -2.95 ) |
| Turkmenistan | Both | 1363.38 ( 1263.32 - 1463.52 ) | 61.81 ( 57.4 - 66.33 ) | 1055.47 ( 931.03 - 1169.18 ) | 24.41 ( 21.67 - 26.91 ) | -22.58 | -4.07 ( -4.63 - -3.51 ) |
| Uganda | Both | 3006.6 ( 2392.12 - 3737.89 ) | 40.27 ( 32.11 - 49.89 ) | 5253.53 ( 4218.53 - 6920.76 ) | 33.29 ( 26.73 - 43.95 ) | 74.73 | -1.32 ( -1.72 - -0.92 ) |
| UK | Both | 21933.36 ( 21432.57 - 22474.38 ) | 25.68 ( 25.08 - 26.33 ) | 18759.05 ( 18070.55 - 19460.62 ) | 16.71 ( 16.1 - 17.34 ) | -14.47 | -1.91 ( -2.02 - -1.79 ) |
| Ukraine | Both | 75447.19 ( 71526.37 - 79175.04 ) | 106.55 ( 101.05 - 111.99 ) | 51019.62 ( 46757.48 - 55549.73 ) | 71.5 ( 65.42 - 77.76 ) | -32.38 | -2.6 ( -3.08 - -2.12 ) |
| United Arab Emirates | Both | 490.68 ( 358.01 - 729.07 ) | 69.58 ( 53.55 - 90.45 ) | 4485.49 ( 3047.46 - 6224.71 ) | 71.42 ( 45.85 - 97.15 ) | 814.13 | 0.1 ( 0.02 - 0.19 ) |
| Uruguay | Both | 4201.67 ( 3956.75 - 4455.46 ) | 109.62 ( 102.97 - 116.18 ) | 2773.76 ( 2357.99 - 3212.71 ) | 57.65 ( 48.71 - 66.76 ) | -33.98 | -2.42 ( -2.57 - -2.27 ) |
| USA | Both | 108949.53 ( 106532.15 - 111951.15 ) | 36.19 ( 35.4 - 37.19 ) | 121169.89 ( 116098.74 - 126130.43 ) | 23.34 ( 22.34 - 24.32 ) | 11.22 | -2.06 ( -2.19 - -1.92 ) |
| Uzbekistan | Both | 6100.32 ( 5793.04 - 6415.23 ) | 47.63 ( 45.18 - 50.05 ) | 10836.36 ( 9543.32 - 12301.25 ) | 41.65 ( 36.72 - 47.08 ) | 77.64 | -0.08 ( -0.64 - 0.47 ) |
| Vanuatu | Both | 36.29 ( 26.54 - 58.49 ) | 48.03 ( 35.8 - 76.48 ) | 89.79 ( 62.17 - 173.35 ) | 49.56 ( 34.58 - 96.1 ) | 147.46 | 0.2 ( 0.14 - 0.26 ) |
| Venezuela | Both | 5903.27 ( 5567.96 - 6230.28 ) | 56.75 ( 53.69 - 59.92 ) | 13591.11 ( 11326.46 - 16260.11 ) | 46.39 ( 38.7 - 55.5 ) | 130.23 | -1 ( -1.12 - -0.88 ) |
| Vietnam | Both | 17250.18 ( 14505.59 - 20068.41 ) | 40.19 ( 33.75 - 46.67 ) | 40063.42 ( 32908.06 - 49592.44 ) | 39.88 ( 33.01 - 48.81 ) | 132.25 | 0.01 ( -0.1 - 0.13 ) |
| Virgin Islands | Both | 50.06 ( 44.64 - 56.04 ) | 53.31 ( 47.76 - 59.87 ) | 107.24 ( 84.54 - 127.53 ) | 57.2 ( 45.47 - 67.92 ) | 114.24 | 0.49 ( 0.37 - 0.6 ) |
| Yemen | Both | 3851.59 ( 2270.6 - 5378.36 ) | 65.07 ( 39.78 - 89.87 ) | 8213.95 ( 6111.44 - 10649.67 ) | 56.83 ( 42.37 - 73.41 ) | 113.26 | -0.58 ( -0.67 - -0.49 ) |
| Zambia | Both | 2412.21 ( 1856.46 - 3154.7 ) | 70.36 ( 55.13 - 91.64 ) | 3324.22 ( 2652.14 - 4922.3 ) | 43.82 ( 35.02 - 64.62 ) | 37.81 | -2.52 ( -2.86 - -2.18 ) |
| Zimbabwe | Both | 2657.91 ( 2235.71 - 3130.5 ) | 56.62 ( 47.87 - 66.45 ) | 4790.93 ( 3770.62 - 5910.09 ) | 59.99 ( 47.47 - 73.31 ) | 80.25 | 0.71 ( -0.04 - 1.47 ) |
| Afghanistan | Female | 2552.97 ( 1420.24 - 3446.37 ) | 70.81 ( 40.38 - 94.87 ) | 4925.06 ( 3541.12 - 6860 ) | 63.32 ( 47.18 - 84.03 ) | 92.91 | -0.5 ( -0.76 - -0.25 ) |
| Albania | Female | 247.26 ( 221.2 - 278.63 ) | 19.97 ( 17.85 - 22.37 ) | 200.87 ( 147.17 - 269.73 ) | 10.07 ( 7.4 - 13.4 ) | -18.76 | -3.05 ( -3.57 - -2.53 ) |
| Algeria | Female | 945.59 ( 799.38 - 1106.02 ) | 12.8 ( 10.8 - 14.99 ) | 1520.06 ( 1300.04 - 1769.14 ) | 8.37 ( 7.19 - 9.74 ) | 60.75 | -1.32 ( -1.42 - -1.21 ) |
| American Samoa | Female | 0.63 ( 0.52 - 0.74 ) | 4.7 ( 3.97 - 5.47 ) | 2.55 ( 2.1 - 3.04 ) | 9.85 ( 8.33 - 11.55 ) | 307.38 | 3.94 ( 3.3 - 4.59 ) |
| Andorra | Female | 1.62 ( 1.17 - 2.21 ) | 5.62 ( 4.08 - 7.69 ) | 2.59 ( 1.98 - 3.28 ) | 4.12 ( 3.16 - 5.22 ) | 60.53 | -1.46 ( -1.85 - -1.07 ) |
| Angola | Female | 367.11 ( 260.09 - 482.34 ) | 14.77 ( 10.73 - 19.4 ) | 590.9 ( 444.8 - 782.55 ) | 8.4 ( 6.38 - 11.07 ) | 60.96 | -2.34 ( -2.56 - -2.11 ) |
| Antigua | Female | 0.78 ( 0.7 - 0.87 ) | 2.8 ( 2.51 - 3.14 ) | 1.24 ( 1.09 - 1.4 ) | 2.3 ( 2.03 - 2.59 ) | 58.84 | -0.88 ( -1.05 - -0.71 ) |
| Argentina | Female | 1732.03 ( 1600.03 - 1876.05 ) | 9.68 ( 8.93 - 10.49 ) | 2673.84 ( 2264.73 - 3162.62 ) | 9.59 ( 8.11 - 11.35 ) | 54.38 | -0.16 ( -0.44 - 0.12 ) |
| Armenia | Female | 258.48 ( 231.36 - 289.17 ) | 15.59 ( 14.04 - 17.33 ) | 246.79 ( 221.28 - 274.58 ) | 10.74 ( 9.67 - 11.9 ) | -4.52 | -0.89 ( -1.69 - -0.08 ) |
| Australia | Female | 689.14 ( 629.39 - 747.98 ) | 6.58 ( 6.02 - 7.15 ) | 728.92 ( 600.75 - 863.93 ) | 3.8 ( 3.13 - 4.5 ) | 5.77 | -2.33 ( -2.49 - -2.17 ) |
| Austria | Female | 368.81 ( 337.2 - 402.72 ) | 6.08 ( 5.57 - 6.62 ) | 387.99 ( 336.98 - 444.11 ) | 4.98 ( 4.32 - 5.74 ) | 5.2 | -0.99 ( -1.22 - -0.76 ) |
| Azerbaijan | Female | 692.6 ( 610.82 - 773.18 ) | 21.66 ( 19.28 - 24.17 ) | 1050.11 ( 880.35 - 1244.41 ) | 18.49 ( 15.62 - 21.86 ) | 51.62 | -1.34 ( -1.9 - -0.78 ) |
| Bahamas | Female | 11.63 ( 10.46 - 12.95 ) | 12.56 ( 11.26 - 13.96 ) | 21.83 ( 18.3 - 26.01 ) | 10.19 ( 8.58 - 12.07 ) | 87.63 | -0.81 ( -0.99 - -0.63 ) |
| Bahrain | Female | 7.84 ( 6.73 - 9.21 ) | 8.31 ( 7.07 - 9.72 ) | 10.21 ( 8.53 - 12.1 ) | 2.41 ( 2.03 - 2.86 ) | 30.19 | -6.08 ( -7.24 - -4.9 ) |
| Bangladesh | Female | 16831.84 ( 13594.34 - 21021.75 ) | 63.34 ( 51.28 - 79.19 ) | 20707.82 ( 15942.63 - 25977.53 ) | 31.59 ( 24.14 - 39.64 ) | 23.03 | -2.04 ( -2.25 - -1.83 ) |
| Barbados | Female | 13.91 ( 12.48 - 15.48 ) | 8.84 ( 7.95 - 9.85 ) | 18.2 ( 15.5 - 21.15 ) | 7.16 ( 6.11 - 8.29 ) | 30.85 | -0.91 ( -1.11 - -0.71 ) |
| Barbuda | Female | 0.78 ( 0.7 - 0.87 ) | 2.8 ( 2.51 - 3.14 ) | 1.24 ( 1.09 - 1.4 ) | 2.3 ( 2.03 - 2.59 ) | 58.84 | -0.88 ( -1.05 - -0.71 ) |
| Belarus | Female | 365.86 ( 334.09 - 401.69 ) | 4.86 ( 4.44 - 5.32 ) | 222.78 ( 192.85 - 253.47 ) | 2.69 ( 2.32 - 3.06 ) | -39.11 | -2.7 ( -3.04 - -2.35 ) |
| Belgium | Female | 954.45 ( 878.02 - 1036.55 ) | 12.69 ( 11.63 - 13.81 ) | 718.89 ( 628.24 - 819.15 ) | 7.46 ( 6.47 - 8.5 ) | -24.68 | -1.98 ( -2.17 - -1.79 ) |
| Belize | Female | 4.64 ( 4.05 - 5.22 ) | 9.63 ( 8.36 - 10.87 ) | 11.53 ( 10.15 - 12.99 ) | 8.25 ( 7.27 - 9.26 ) | 148.62 | -0.56 ( -0.98 - -0.14 ) |
| Benin | Female | 78.07 ( 63.43 - 95.52 ) | 6.75 ( 5.44 - 8.25 ) | 167.48 ( 120.54 - 224.54 ) | 6.06 ( 4.42 - 8.15 ) | 114.52 | -0.31 ( -0.39 - -0.22 ) |
| Bermuda | Female | 3.94 ( 3.52 - 4.4 ) | 10.98 ( 9.8 - 12.26 ) | 2.87 ( 2.43 - 3.36 ) | 4.3 ( 3.63 - 5.04 ) | -27.22 | -3.93 ( -4.24 - -3.62 ) |
| Bhutan | Female | 94.55 ( 73.67 - 123.85 ) | 61.43 ( 47.7 - 80.49 ) | 82.26 ( 59.59 - 107.4 ) | 24.8 ( 18.36 - 32.3 ) | -13 | -3.67 ( -3.85 - -3.5 ) |
| Bolivia | Female | 540.72 ( 444.18 - 668.5 ) | 27.42 ( 22.38 - 33.79 ) | 628.14 ( 468.03 - 824.93 ) | 13.24 ( 9.89 - 17.34 ) | 16.17 | -3.05 ( -3.27 - -2.83 ) |
| Bosnia and Herzegovina | Female | 497.04 ( 442.63 - 558.46 ) | 20.04 ( 17.86 - 22.39 ) | 379.27 ( 324.14 - 445.41 ) | 12.08 ( 10.36 - 14.12 ) | -23.69 | -2.83 ( -3.44 - -2.21 ) |
| Botswana | Female | 43.68 ( 30.66 - 57.9 ) | 12.02 ( 8.5 - 15.78 ) | 87.57 ( 70.32 - 107.56 ) | 10.17 ( 8.18 - 12.5 ) | 100.49 | 0.85 ( -0.05 - 1.75 ) |
| Brazil | Female | 8431.49 ( 8114.6 - 8772.36 ) | 16.13 ( 15.52 - 16.76 ) | 15519.12 ( 14848.23 - 16231.1 ) | 12.29 ( 11.76 - 12.85 ) | 84.06 | -1.1 ( -1.2 - -1.01 ) |
| Brunei | Female | 17.31 ( 14.86 - 20.11 ) | 32.67 ( 27.87 - 37.95 ) | 11.95 ( 10.18 - 13.83 ) | 7.03 ( 6.01 - 8.08 ) | -30.93 | -6.94 ( -7.52 - -6.35 ) |
| Bulgaria | Female | 486.49 ( 441.93 - 535.09 ) | 7.49 ( 6.81 - 8.22 ) | 514.3 ( 447.67 - 581.22 ) | 8.04 ( 6.97 - 9.2 ) | 5.72 | 0.22 ( 0.05 - 0.39 ) |
| Burkina Faso | Female | 266.24 ( 214.76 - 326.47 ) | 10.11 ( 8.19 - 12.34 ) | 417.19 ( 332.24 - 523.45 ) | 7.87 ( 6.23 - 9.77 ) | 56.7 | -0.97 ( -1.13 - -0.81 ) |
| Burundi | Female | 527.96 ( 404.94 - 700.44 ) | 35.3 ( 26.89 - 46.69 ) | 427.72 ( 305.32 - 564.64 ) | 16.81 ( 11.93 - 22.11 ) | -18.99 | -3.37 ( -3.67 - -3.07 ) |
| Cambodia | Female | 1043.97 ( 847.92 - 1267.33 ) | 34.22 ( 27.99 - 41.43 ) | 1094.16 ( 852.9 - 1386.71 ) | 15.46 ( 12.08 - 19.51 ) | 4.81 | -3.19 ( -3.3 - -3.08 ) |
| Cameroon | Female | 233.5 ( 190.43 - 290.99 ) | 8.65 ( 7.1 - 10.74 ) | 452.38 ( 325.25 - 621 ) | 6.8 ( 4.92 - 9.32 ) | 93.74 | -0.77 ( -0.93 - -0.61 ) |
| Canada | Female | 1712.42 ( 1587.58 - 1840.61 ) | 9.93 ( 9.17 - 10.68 ) | 1697.79 ( 1487.41 - 1938.37 ) | 5.24 ( 4.6 - 5.99 ) | -0.85 | -2.72 ( -2.88 - -2.56 ) |
| Cape Verde | Female | 3.26 ( 2.79 - 3.78 ) | 2.46 ( 2.11 - 2.88 ) | 12.95 ( 11.08 - 15 ) | 5.31 ( 4.5 - 6.15 ) | 297.92 | 2.75 ( 2.1 - 3.41 ) |
| Central African Republic | Female | 156.31 ( 114.84 - 198.74 ) | 20.67 ( 15.63 - 26 ) | 194.32 ( 128.89 - 267.56 ) | 14.69 ( 9.91 - 19.86 ) | 24.32 | -1.32 ( -1.45 - -1.19 ) |
| Chad | Female | 111.98 ( 90.37 - 137.19 ) | 6.95 ( 5.62 - 8.48 ) | 211.86 ( 159.4 - 278.44 ) | 7.45 ( 5.61 - 9.76 ) | 89.19 | 0.46 ( 0.38 - 0.55 ) |
| Chile | Female | 448.72 ( 408.2 - 489.65 ) | 7.84 ( 7.13 - 8.56 ) | 484.55 ( 404.47 - 574.7 ) | 3.89 ( 3.25 - 4.61 ) | 7.98 | -2.45 ( -2.76 - -2.14 ) |
| China | Female | 66304.91 ( 62705.49 - 71223.76 ) | 13.88 ( 13.14 - 14.92 ) | 71781.99 ( 67389.05 - 76308.35 ) | 7.17 ( 6.74 - 7.63 ) | 8.26 | -2.41 ( -2.72 - -2.1 ) |
| Colombia | Female | 2444.48 ( 2291.1 - 2609.63 ) | 24.91 ( 23.36 - 26.63 ) | 1994.2 ( 1674.71 - 2349.36 ) | 6.85 ( 5.74 - 8.07 ) | -18.42 | -5.65 ( -5.96 - -5.33 ) |
| Comoros | Female | 30.98 ( 21.12 - 41.02 ) | 24.63 ( 16.87 - 32.4 ) | 36.25 ( 24.79 - 48.83 ) | 13.31 ( 9.1 - 18.02 ) | 17.01 | -2.56 ( -2.7 - -2.41 ) |
| Costa Rica | Female | 94.03 ( 86.02 - 102.62 ) | 9.9 ( 9.04 - 10.81 ) | 104.5 ( 90.95 - 119.54 ) | 3.95 ( 3.45 - 4.52 ) | 11.14 | -3.79 ( -4.17 - -3.41 ) |
| Croatia | Female | 359.75 ( 327.01 - 395.14 ) | 9.87 ( 9.03 - 10.81 ) | 243.77 ( 212.7 - 279.98 ) | 5.8 ( 5.03 - 6.63 ) | -32.24 | -2.07 ( -2.57 - -1.56 ) |
| Cuba | Female | 1628.14 ( 1501.15 - 1755.9 ) | 30.72 ( 28.28 - 33.18 ) | 2474.33 ( 2084.38 - 2933.43 ) | 26.05 ( 21.91 - 30.9 ) | 51.97 | -0.73 ( -0.92 - -0.55 ) |
| Cyprus | Female | 27.65 ( 23.98 - 31.8 ) | 6.27 ( 5.45 - 7.2 ) | 23.95 ( 20.32 - 28.61 ) | 2.64 ( 2.25 - 3.15 ) | -13.38 | -3.64 ( -3.94 - -3.34 ) |
| Czech Republic | Female | 606.88 ( 558.61 - 662.7 ) | 8.16 ( 7.5 - 8.88 ) | 539.35 ( 471.2 - 612.34 ) | 5.71 ( 4.96 - 6.48 ) | -11.13 | -1.04 ( -1.24 - -0.84 ) |
| Democratic Republic of the Congo | Female | 1267.19 ( 939.02 - 1631.87 ) | 12.24 ( 9.16 - 15.56 ) | 2156.08 ( 1560.8 - 2935.8 ) | 10.12 ( 7.27 - 13.64 ) | 70.15 | -0.78 ( -0.87 - -0.69 ) |
| Denmark | Female | 646.95 ( 591.17 - 708.11 ) | 16.89 ( 15.32 - 18.5 ) | 382.21 ( 332.86 - 434.82 ) | 7.37 ( 6.43 - 8.42 ) | -40.92 | -3.43 ( -3.61 - -3.25 ) |
| Djibouti | Female | 21.31 ( 14.47 - 29.37 ) | 21.17 ( 14.62 - 29.14 ) | 39.88 ( 25.69 - 58.85 ) | 11.99 ( 7.9 - 17.62 ) | 87.14 | -2.52 ( -2.74 - -2.3 ) |
| Dominica | Female | 5.82 ( 5.22 - 6.52 ) | 14.7 ( 13.19 - 16.38 ) | 5.82 ( 4.98 - 6.7 ) | 13.11 ( 11.23 - 15.11 ) | -0.06 | -0.54 ( -0.7 - -0.39 ) |
| Dominican Republic | Female | 393.41 ( 338.48 - 450.79 ) | 17.9 ( 15.5 - 20.51 ) | 613.92 ( 491.49 - 766.69 ) | 12.56 ( 10.04 - 15.6 ) | 56.05 | -1.28 ( -1.69 - -0.88 ) |
| Ecuador | Female | 389.47 ( 357.88 - 421.04 ) | 12.54 ( 11.54 - 13.56 ) | 610.62 ( 519.4 - 712.14 ) | 7.82 ( 6.64 - 9.12 ) | 56.78 | -1.95 ( -2.36 - -1.54 ) |
| Egypt | Female | 1847.11 ( 1546.08 - 2217.64 ) | 9.18 ( 7.89 - 10.7 ) | 2653.7 ( 2090.18 - 3336.7 ) | 6.74 ( 5.4 - 8.33 ) | 43.67 | -0.99 ( -1.13 - -0.85 ) |
| El Salvador | Female | 73.56 ( 65.31 - 82.65 ) | 4.24 ( 3.75 - 4.79 ) | 148.58 ( 115.84 - 187.25 ) | 4.53 ( 3.53 - 5.72 ) | 101.99 | 0.45 ( 0.17 - 0.73 ) |
| Equatorial Guinea | Female | 23.84 ( 17.24 - 31.26 ) | 18.73 ( 13.66 - 24.48 ) | 21.81 ( 13.09 - 34.22 ) | 6.96 ( 4.24 - 10.84 ) | -8.52 | -4.13 ( -4.49 - -3.77 ) |
| Eritrea | Female | 210.38 ( 153.49 - 277.25 ) | 30 ( 22.27 - 38.95 ) | 296.05 ( 207.61 - 397.07 ) | 17.8 ( 12.6 - 23.4 ) | 40.72 | -2.05 ( -2.16 - -1.94 ) |
| Estonia | Female | 79.22 ( 71.55 - 87.16 ) | 6.49 ( 5.88 - 7.13 ) | 50.87 ( 41.17 - 62.67 ) | 4.02 ( 3.24 - 4.93 ) | -35.79 | -2.39 ( -2.75 - -2.04 ) |
| Ethiopia | Female | 2433.03 ( 1897.17 - 3019.24 ) | 19.07 ( 14.95 - 23.65 ) | 1727.28 ( 1478.63 - 2005.42 ) | 7.17 ( 6.12 - 8.34 ) | -29.01 | -4.15 ( -4.39 - -3.91 ) |
| Fiji | Female | 24.52 ( 19.4 - 30.46 ) | 10.78 ( 8.71 - 13.26 ) | 48 ( 37.78 - 59.43 ) | 11.3 ( 8.9 - 13.94 ) | 95.8 | 0.5 ( 0.31 - 0.69 ) |
| Finland | Female | 164.85 ( 150.61 - 180.44 ) | 4.31 ( 3.94 - 4.71 ) | 129.47 ( 112.77 - 147.68 ) | 2.52 ( 2.21 - 2.89 ) | -21.46 | -1.89 ( -2.02 - -1.76 ) |
| France | Female | 4237.61 ( 3999.21 - 4476.95 ) | 10.57 ( 9.94 - 11.21 ) | 4350.91 ( 3832.92 - 4959.61 ) | 7.79 ( 6.82 - 8.96 ) | 2.67 | -1.12 ( -1.3 - -0.94 ) |
| Gabon | Female | 42.15 ( 32.66 - 53.98 ) | 12.63 ( 9.86 - 16.16 ) | 41.76 ( 30.03 - 55.66 ) | 7.01 ( 5.05 - 9.37 ) | -0.92 | -2.23 ( -2.56 - -1.91 ) |
| Gambia | Female | 10.1 ( 7.68 - 12.93 ) | 16.17 ( 14.28 - 18.3 ) | 26.99 ( 20.94 - 34.61 ) | 4.75 ( 3.99 - 5.6 ) | 167.31 | 0.13 ( 0.04 - 0.21 ) |
| Georgia | Female | 511.99 ( 461.77 - 569.2 ) | 5.03 ( 3.83 - 6.49 ) | 335.91 ( 296.27 - 375.98 ) | 4.98 ( 3.83 - 6.36 ) | -34.39 | -1.23 ( -1.87 - -0.59 ) |
| Germany | Female | 4328.56 ( 4077.1 - 4601.02 ) | 14.31 ( 12.87 - 15.89 ) | 4940.63 ( 4103.55 - 5847.98 ) | 11.11 ( 9.82 - 12.52 ) | 14.14 | -0.52 ( -0.68 - -0.36 ) |
| Ghana | Female | 276.25 ( 211.85 - 356.76 ) | 6.65 ( 6.24 - 7.09 ) | 565.36 ( 439.59 - 726.37 ) | 6.25 ( 5.16 - 7.39 ) | 104.65 | -0.54 ( -0.61 - -0.48 ) |
| Greece | Female | 534.32 ( 486.72 - 586.88 ) | 7.05 ( 5.39 - 9.17 ) | 582.69 ( 507.8 - 662.62 ) | 5.88 ( 4.61 - 7.52 ) | 9.05 | -0.49 ( -0.74 - -0.25 ) |
| Greenland | Female | 1.04 ( 0.9 - 1.2 ) | 6.7 ( 6.1 - 7.34 ) | 1.59 ( 1.31 - 1.89 ) | 5.63 ( 4.87 - 6.4 ) | 52.91 | 0.04 ( -0.5 - 0.58 ) |
| Grenada | Female | 5.8 ( 5.16 - 6.45 ) | 5.37 ( 4.64 - 6.19 ) | 5.46 ( 4.83 - 6.18 ) | 4.68 ( 3.93 - 5.58 ) | -5.91 | -1.87 ( -2.17 - -1.58 ) |
| Grenadines | Female | 4.18 ( 3.75 - 4.65 ) | 32.45 ( 26.32 - 39.58 ) | 5.37 ( 4.71 - 6.12 ) | 10.73 ( 8.1 - 13.9 ) | 28.51 | -1.15 ( -1.46 - -0.85 ) |
| Guam | Female | 1.7 ( 1.44 - 1.99 ) | 14.91 ( 13.23 - 16.52 ) | 11.06 ( 9.3 - 13.04 ) | 7.81 ( 6.87 - 8.91 ) | 552.36 | 5.49 ( 4.77 - 6.22 ) |
| Guatemala | Female | 283.85 ( 259.1 - 311.43 ) | 10.32 ( 9.24 - 11.51 ) | 438.29 ( 365.38 - 515.36 ) | 8.18 ( 7.17 - 9.34 ) | 54.41 | -2.5 ( -3.18 - -1.8 ) |
| Guinea | Female | 139.54 ( 112.05 - 169.16 ) | 3.89 ( 3.31 - 4.52 ) | 227.82 ( 176.63 - 291.63 ) | 11.66 ( 9.83 - 13.8 ) | 63.26 | 0.32 ( 0.18 - 0.46 ) |
| Guinea-Bissau | Female | 26.52 ( 19.87 - 34.45 ) | 12.89 ( 11.75 - 14.11 ) | 37.44 ( 27.83 - 48.36 ) | 6.88 ( 5.74 - 8.12 ) | 41.16 | -0.7 ( -0.82 - -0.59 ) |
| Guyana | Female | 16.57 ( 14.85 - 18.4 ) | 7.56 ( 6.1 - 9.15 ) | 24.19 ( 19.98 - 29.2 ) | 7.82 ( 6.08 - 10.03 ) | 45.94 | -0.35 ( -0.73 - 0.03 ) |
| Haiti | Female | 592.39 ( 467.07 - 736.84 ) | 10.74 ( 8.09 - 13.83 ) | 773.68 ( 553.16 - 1028.02 ) | 8.62 ( 6.48 - 11.08 ) | 30.6 | -1.9 ( -1.99 - -1.8 ) |
| Honduras | Female | 47.52 ( 39.53 - 57.15 ) | 7.49 ( 6.7 - 8.33 ) | 116.19 ( 85.09 - 151.12 ) | 6.94 ( 5.72 - 8.34 ) | 144.5 | -0.73 ( -0.88 - -0.59 ) |
| Hungary | Female | 1518.85 ( 1376.44 - 1655.11 ) | 31.49 ( 25.01 - 38.69 ) | 1605.09 ( 1420.39 - 1829.24 ) | 19.33 ( 13.99 - 25.52 ) | 5.68 | -0.14 ( -0.54 - 0.27 ) |
| Iceland | Female | 9.82 ( 8.9 - 10.88 ) | 4.06 ( 3.37 - 4.89 ) | 7.03 ( 6.18 - 8.02 ) | 3.48 ( 2.53 - 4.49 ) | -28.41 | -3.36 ( -3.49 - -3.23 ) |
| India | Female | 139711.46 ( 125437.18 - 154004.05 ) | 19.97 ( 18.07 - 21.86 ) | 218208.58 ( 200965.37 - 234630.64 ) | 18.13 ( 16.04 - 20.68 ) | 56.19 | -1.63 ( -1.95 - -1.3 ) |
| Indonesia | Female | 12086.17 ( 10825.68 - 13538.34 ) | 7 ( 6.33 - 7.77 ) | 12821.66 ( 11611.04 - 14253.25 ) | 2.87 ( 2.51 - 3.26 ) | 6.09 | -2.25 ( -2.32 - -2.19 ) |
| Iran | Female | 5021.01 ( 4638.41 - 5412.46 ) | 49.7 ( 44.54 - 54.87 ) | 6990.91 ( 6676.19 - 7386.01 ) | 35.88 ( 33 - 38.59 ) | 39.23 | -2.41 ( -2.91 - -1.91 ) |
| Iraq | Female | 2112.67 ( 1681.68 - 2653.01 ) | 19.7 ( 17.67 - 21.99 ) | 2071.3 ( 1788.88 - 2417.62 ) | 10.66 ( 9.67 - 11.82 ) | -1.96 | -4.54 ( -5.24 - -3.85 ) |
| Ireland | Female | 265.62 ( 240.01 - 292.42 ) | 32.96 ( 30.61 - 35.32 ) | 190.87 ( 163.46 - 221.86 ) | 18.71 ( 17.87 - 19.73 ) | -28.14 | -3.54 ( -3.74 - -3.34 ) |
| Israel | Female | 134.92 ( 122.26 - 148.53 ) | 45.96 ( 36.7 - 57.76 ) | 212.68 ( 185.9 - 242.4 ) | 15.18 ( 13.22 - 17.61 ) | 57.63 | -1.64 ( -1.99 - -1.3 ) |
| Italy | Female | 3750.94 ( 3520.76 - 3970.12 ) | 12.51 ( 11.31 - 13.79 ) | 3044.84 ( 2662.71 - 3430.73 ) | 5.41 ( 4.62 - 6.31 ) | -18.82 | -1.71 ( -1.81 - -1.6 ) |
| Ivory Coast | Female | 61.23 ( 50 - 74.76 ) | 5.29 ( 4.8 - 5.82 ) | 126.86 ( 97.17 - 161.43 ) | 3.79 ( 3.29 - 4.32 ) | 107.19 | -0.65 ( -0.83 - -0.47 ) |
| Jamaica | Female | 66.47 ( 59.2 - 74.34 ) | 8.04 ( 7.52 - 8.51 ) | 78.41 ( 61.18 - 98.66 ) | 4.85 ( 4.22 - 5.48 ) | 17.97 | -1.14 ( -1.54 - -0.73 ) |
| Japan | Female | 2973.05 ( 2852.11 - 3094.55 ) | 2.62 ( 2.15 - 3.21 ) | 2430.53 ( 2268.18 - 2604.75 ) | 2.21 ( 1.68 - 2.85 ) | -18.25 | -2.29 ( -2.38 - -2.19 ) |
| Jordan | Female | 108.82 ( 90.11 - 132.04 ) | 7.08 ( 6.29 - 7.95 ) | 96.98 ( 78.43 - 117.06 ) | 5.26 ( 4.1 - 6.64 ) | -10.88 | -6.68 ( -7.76 - -5.58 ) |
| Kazakhstan | Female | 1492.77 ( 1358.58 - 1637.46 ) | 3.16 ( 3.03 - 3.29 ) | 701.51 ( 608.66 - 804.26 ) | 1.73 ( 1.62 - 1.85 ) | -53.01 | -4.28 ( -4.67 - -3.89 ) |
| Kenya | Female | 645.81 ( 546.36 - 749.26 ) | 12.74 ( 10.55 - 15.51 ) | 1235.9 ( 1071.67 - 1415.7 ) | 3 ( 2.42 - 3.64 ) | 91.37 | -1.35 ( -1.58 - -1.12 ) |
| Kiribati | Female | 5.15 ( 4.29 - 6.05 ) | 18.25 ( 16.59 - 19.94 ) | 8.14 ( 6.21 - 10.34 ) | 6.79 ( 5.92 - 7.74 ) | 58.16 | -0.68 ( -0.83 - -0.53 ) |
| Kuwait | Female | 31.62 ( 27.67 - 35.63 ) | 12.55 ( 10.62 - 14.62 ) | 33.17 ( 28.67 - 38.48 ) | 9.13 ( 7.87 - 10.47 ) | 4.91 | -4.94 ( -5.95 - -3.92 ) |
| Kyrgyzstan | Female | 170.53 ( 151.11 - 191.72 ) | 21.64 ( 18.2 - 25.25 ) | 125.48 ( 111.36 - 140.36 ) | 18.29 ( 14.17 - 22.74 ) | -26.42 | -3.07 ( -3.54 - -2.6 ) |
| Laos | Female | 485.76 ( 367.96 - 632.95 ) | 9.67 ( 8.38 - 10.9 ) | 372.49 ( 285.27 - 480.72 ) | 2.44 ( 2.1 - 2.79 ) | -23.32 | -3.67 ( -3.78 - -3.56 ) |
| Latvia | Female | 156.55 ( 141.67 - 173.24 ) | 9.05 ( 8.02 - 10.15 ) | 88.38 ( 72.49 - 106.55 ) | 4.59 ( 4.08 - 5.13 ) | -43.55 | -1.72 ( -2.06 - -1.37 ) |
| Lebanon | Female | 438.1 ( 348 - 546.33 ) | 38.27 ( 29.07 - 49.96 ) | 544.61 ( 450.37 - 649.68 ) | 15.12 ( 11.65 - 19.45 ) | 24.31 | -2.99 ( -3.25 - -2.72 ) |
| Lesotho | Female | 97.15 ( 69.06 - 127.03 ) | 7.14 ( 6.47 - 7.87 ) | 125.61 ( 82.93 - 178.46 ) | 4.36 ( 3.58 - 5.27 ) | 29.29 | 0.96 ( 0.55 - 1.38 ) |
| Liberia | Female | 44.17 ( 34.5 - 55.2 ) | 31.78 ( 25.3 - 39.31 ) | 72.01 ( 53.38 - 94.66 ) | 15.81 ( 13.08 - 18.92 ) | 63.02 | -0.73 ( -0.89 - -0.57 ) |
| Libya | Female | 175.5 ( 138.75 - 217.49 ) | 16.6 ( 11.84 - 21.76 ) | 446.61 ( 341.75 - 569.06 ) | 16.64 ( 11.04 - 23.58 ) | 154.48 | -0.23 ( -0.34 - -0.13 ) |
| Lithuania | Female | 174.78 ( 157.7 - 192.49 ) | 7.9 ( 6.17 - 9.87 ) | 157.65 ( 134.67 - 182.53 ) | 6.63 ( 4.94 - 8.62 ) | -9.8 | -0.72 ( -1.12 - -0.31 ) |
| Luxembourg | Female | 31.56 ( 28.3 - 35.01 ) | 17.2 ( 13.61 - 21.2 ) | 34.29 ( 28.37 - 40.7 ) | 16.37 ( 12.72 - 20.56 ) | 8.66 | -1.42 ( -1.54 - -1.3 ) |
| Macedonia | Female | 163.19 ( 143.88 - 185.48 ) | 6.55 ( 5.94 - 7.2 ) | 200.56 ( 168.42 - 234.81 ) | 5.66 ( 4.86 - 6.57 ) | 22.9 | -0.91 ( -1.28 - -0.54 ) |
| Madagascar | Female | 688.27 ( 536.05 - 837.34 ) | 11.1 ( 9.95 - 12.38 ) | 945.01 ( 657.43 - 1279.77 ) | 7.55 ( 6.26 - 8.95 ) | 37.3 | -2.03 ( -2.18 - -1.88 ) |
| Malawi | Female | 379.14 ( 221.13 - 492.63 ) | 15.26 ( 13.46 - 17.3 ) | 388.56 ( 296.59 - 496.19 ) | 12.79 ( 10.76 - 15.01 ) | 2.48 | -3.09 ( -3.56 - -2.63 ) |
| Malaysia | Female | 691.92 ( 604.05 - 791.04 ) | 21.66 ( 16.94 - 26.36 ) | 1099.72 ( 924.01 - 1303.27 ) | 13.57 ( 9.45 - 18.25 ) | 58.94 | -2.01 ( -2.28 - -1.73 ) |
| Maldives | Female | 3.52 ( 2.89 - 4.33 ) | 14.75 ( 9.21 - 18.88 ) | 2.65 ( 2.26 - 3.1 ) | 8.34 ( 6.39 - 10.76 ) | -24.84 | -5.89 ( -6.16 - -5.62 ) |
| Mali | Female | 361.56 ( 297.68 - 430.76 ) | 13 ( 11.32 - 14.86 ) | 435.6 ( 337.63 - 554.01 ) | 8.14 ( 6.82 - 9.64 ) | 20.48 | -2.12 ( -2.28 - -1.96 ) |
| Malta | Female | 18.4 ( 16.47 - 20.39 ) | 7.51 ( 6.22 - 9.19 ) | 23.42 ( 20.44 - 27.12 ) | 1.84 ( 1.57 - 2.18 ) | 27.23 | -1.6 ( -1.85 - -1.35 ) |
| Marshall Islands | Female | 1.91 ( 1.55 - 2.35 ) | 14.66 ( 12.18 - 17.37 ) | 4.6 ( 3.46 - 6 ) | 8.92 ( 6.9 - 11.36 ) | 140.69 | 0.54 ( 0.22 - 0.87 ) |
| Mauritania | Female | 47.19 ( 37.33 - 58.02 ) | 7.82 ( 7.01 - 8.67 ) | 60.64 ( 45.16 - 79.48 ) | 5.86 ( 5.11 - 6.72 ) | 28.51 | -1.47 ( -1.6 - -1.34 ) |
| Mauritius | Female | 64.68 ( 58.23 - 71.54 ) | 19.86 ( 16.22 - 24.61 ) | 40.12 ( 34.88 - 46.59 ) | 22.86 ( 17.61 - 29.55 ) | -37.97 | -6.18 ( -6.91 - -5.43 ) |
| Mexico | Female | 3128.28 ( 3032.32 - 3222.55 ) | 8.29 ( 6.53 - 10.2 ) | 3122.11 ( 2978.7 - 3270.36 ) | 5.53 ( 4.12 - 7.26 ) | -0.2 | -4.06 ( -4.33 - -3.78 ) |
| Micronesia | Female | 6.01 ( 4.66 - 7.43 ) | 14.93 ( 13.49 - 16.48 ) | 8.47 ( 6.45 - 11.15 ) | 4.41 ( 3.85 - 5.09 ) | 41.01 | -0.47 ( -0.55 - -0.39 ) |
| Moldova | Female | 164.56 ( 146.87 - 184.9 ) | 12.71 ( 12.32 - 13.1 ) | 145.02 ( 128 - 162.81 ) | 5.05 ( 4.81 - 5.28 ) | -11.88 | -0.48 ( -1.04 - 0.09 ) |
| Mongolia | Female | 64.81 ( 54.69 - 75.95 ) | 22.03 ( 17.39 - 26.85 ) | 79.12 ( 65.49 - 95.36 ) | 19.47 ( 15.2 - 25.32 ) | 22.08 | -2.16 ( -2.65 - -1.67 ) |
| Montenegro | Female | 166.07 ( 141.19 - 192.55 ) | 6.14 ( 5.48 - 6.87 ) | 208.41 ( 173.18 - 247.11 ) | 4.77 ( 4.23 - 5.35 ) | 25.5 | -0.46 ( -0.92 - 0 ) |
| Morocco | Female | 1209.97 ( 1027.66 - 1430.89 ) | 10.74 ( 9.06 - 12.56 ) | 1631.27 ( 1253.03 - 2087.06 ) | 6.87 ( 5.59 - 8.35 ) | 34.82 | -1.69 ( -1.74 - -1.64 ) |
| Mozambique | Female | 916.44 ( 657.38 - 1190.28 ) | 46.67 ( 39.86 - 54.06 ) | 1207.2 ( 871.01 - 1602.3 ) | 41.94 ( 34.78 - 49.59 ) | 31.73 | -1.38 ( -1.55 - -1.2 ) |
| Myanmar | Female | 6771.33 ( 4922.31 - 9132.07 ) | 14.52 ( 12.3 - 17.22 ) | 4614.93 ( 3460.24 - 5917.08 ) | 9.34 ( 7.22 - 11.94 ) | -31.85 | -4.05 ( -4.32 - -3.77 ) |
| Namibia | Female | 178.16 ( 139.09 - 221.41 ) | 22.3 ( 16 - 28.86 ) | 168.48 ( 122.5 - 228.35 ) | 16.03 ( 11.61 - 21.18 ) | -5.43 | -3.66 ( -4.5 - -2.81 ) |
| Nepal | Female | 4453.42 ( 3138.75 - 5969.74 ) | 48.48 ( 35.2 - 65.26 ) | 5338.77 ( 3716.81 - 6974.43 ) | 17.32 ( 13.05 - 22.16 ) | 19.88 | -2.32 ( -2.69 - -1.96 ) |
| Netherlands | Female | 662.77 ( 606.55 - 723.53 ) | 41.72 ( 32.63 - 51.89 ) | 1020.61 ( 873.78 - 1169.46 ) | 19.4 ( 14.14 - 26.28 ) | 53.99 | -0.33 ( -0.73 - 0.07 ) |
| New Zealand | Female | 161.83 ( 147.48 - 177.49 ) | 78.55 ( 53.96 - 104.58 ) | 124.79 ( 110.3 - 141.18 ) | 42.88 ( 29.58 - 56.12 ) | -22.89 | -2.89 ( -3.12 - -2.66 ) |
| Nicaragua | Female | 125.49 ( 108.69 - 143.98 ) | 6.58 ( 5.99 - 7.17 ) | 115.91 ( 95.23 - 139.38 ) | 6.6 ( 5.65 - 7.55 ) | -7.63 | -4.35 ( -4.77 - -3.92 ) |
| Niger | Female | 116.62 ( 92.37 - 145.84 ) | 7.83 ( 7.12 - 8.56 ) | 259.68 ( 185.14 - 343.74 ) | 3.48 ( 3.09 - 3.93 ) | 122.67 | -0.76 ( -0.88 - -0.65 ) |
| Nigeria | Female | 824.48 ( 613.83 - 1077.92 ) | 13.21 ( 11.45 - 15.14 ) | 1299.86 ( 854.48 - 1903.93 ) | 4.54 ( 3.74 - 5.46 ) | 57.66 | -1.72 ( -1.84 - -1.6 ) |
| North Korea | Female | 944.03 ( 706.2 - 1202.45 ) | 7.05 ( 5.57 - 8.7 ) | 1568.81 ( 1219.28 - 1997.69 ) | 5.99 ( 4.32 - 7.95 ) | 66.18 | 0.16 ( 0.01 - 0.3 ) |
| Northern Mariana Islands | Female | 0.8 ( 0.62 - 1 ) | 3.68 ( 2.74 - 4.81 ) | 4.21 ( 3.43 - 5.15 ) | 2.5 ( 1.66 - 3.58 ) | 428.23 | 2.92 ( 2.24 - 3.61 ) |
| Norway | Female | 165.3 ( 159.1 - 171.71 ) | 8.79 ( 6.59 - 11.16 ) | 143.16 ( 134.9 - 152.68 ) | 8.79 ( 6.84 - 11.2 ) | -13.4 | -1.96 ( -2.21 - -1.7 ) |
| Oman | Female | 27.8 ( 20.69 - 36.73 ) | 8.81 ( 7.09 - 10.77 ) | 35.25 ( 27.48 - 45.06 ) | 13.97 ( 11.7 - 16.87 ) | 26.79 | -2.92 ( -3.21 - -2.62 ) |
| Pakistan | Female | 18335.61 ( 15625.03 - 21324.11 ) | 5.13 ( 4.93 - 5.33 ) | 34596.26 ( 25696.88 - 44699.34 ) | 3.34 ( 3.14 - 3.55 ) | 88.68 | -1.13 ( -1.47 - -0.8 ) |
| Palestine | Female | 47.43 ( 36.33 - 60.96 ) | 7.68 ( 5.75 - 10.11 ) | 71.03 ( 59.99 - 83.81 ) | 3.75 ( 2.9 - 4.77 ) | 49.76 | -2.31 ( -2.53 - -2.09 ) |
| Panama | Female | 72.19 ( 65.61 - 78.78 ) | 61.07 ( 52.2 - 71.12 ) | 91.75 ( 80.87 - 104.71 ) | 51.84 ( 38.99 - 66.52 ) | 27.1 | -2.75 ( -3.12 - -2.37 ) |
| Papua New Guinea | Female | 302.63 ( 232.67 - 381.49 ) | 8.56 ( 6.66 - 10.88 ) | 699.48 ( 533.2 - 888.06 ) | 4.81 ( 4.08 - 5.63 ) | 131.13 | 0.19 ( 0.04 - 0.34 ) |
| Paraguay | Female | 60.89 ( 53.24 - 69.78 ) | 8.99 ( 8.16 - 9.83 ) | 121.27 ( 92.94 - 154.56 ) | 4.54 ( 3.99 - 5.18 ) | 99.17 | -0.41 ( -0.81 - -0.01 ) |
| Peru | Female | 1058.64 ( 903.23 - 1224.27 ) | 26.97 ( 21.24 - 33.56 ) | 958.6 ( 759.07 - 1205.23 ) | 26.33 ( 20.73 - 32.83 ) | -9.45 | -4.13 ( -4.55 - -3.71 ) |
| Philippines | Female | 2394.68 ( 2142.33 - 2649.16 ) | 4.95 ( 4.31 - 5.68 ) | 3584.67 ( 2865.29 - 4410.19 ) | 4.28 ( 3.29 - 5.44 ) | 49.69 | -1.69 ( -1.97 - -1.41 ) |
| Poland | Female | 4070.39 ( 3836.91 - 4314.38 ) | 14.88 ( 12.76 - 17.16 ) | 4456.41 ( 3916.61 - 5030.68 ) | 6.04 ( 4.77 - 7.59 ) | 9.48 | -1.07 ( -1.25 - -0.88 ) |
| Portugal | Female | 694.1 ( 637.74 - 754.8 ) | 13.21 ( 11.85 - 14.56 ) | 348.97 ( 305.97 - 394.31 ) | 8.79 ( 7.09 - 10.72 ) | -49.72 | -4.64 ( -5.17 - -4.12 ) |
| Puerto Rico | Female | 239.2 ( 217.78 - 262.62 ) | 16.43 ( 15.45 - 17.44 ) | 158.5 ( 139.01 - 180.17 ) | 12.95 ( 11.38 - 14.57 ) | -33.74 | -3.83 ( -4.25 - -3.41 ) |
| Qatar | Female | 2.59 ( 2.04 - 3.24 ) | 9.38 ( 8.62 - 10.18 ) | 14.29 ( 10.83 - 18.63 ) | 3.19 ( 2.78 - 3.63 ) | 451.58 | -0.54 ( -1.45 - 0.37 ) |
| Republic of Congo | Female | 133.17 ( 104.2 - 164.94 ) | 12.04 ( 10.97 - 13.25 ) | 198.71 ( 143.28 - 260.06 ) | 4.6 ( 4.03 - 5.23 ) | 49.22 | -1.7 ( -1.95 - -1.46 ) |
| Romania | Female | 1653.28 ( 1517.58 - 1792.36 ) | 5.33 ( 4.29 - 6.55 ) | 1403.28 ( 1242.63 - 1586.51 ) | 3.72 ( 2.85 - 4.7 ) | -15.12 | -1.06 ( -1.26 - -0.86 ) |
| Russia | Female | 8162.3 ( 7761.73 - 8626.24 ) | 19.09 ( 15.02 - 23.59 ) | 6115.17 ( 5844.95 - 6410.13 ) | 12.94 ( 9.47 - 16.73 ) | -25.08 | -1.98 ( -2.33 - -1.63 ) |
| Rwanda | Female | 639.33 ( 518.61 - 781.32 ) | 10.93 ( 10.04 - 11.82 ) | 416.98 ( 311 - 537.34 ) | 8.38 ( 7.36 - 9.55 ) | -34.78 | -5.1 ( -5.58 - -4.63 ) |
| Saint Lucia | Female | 6.6 ( 5.92 - 7.35 ) | 7.27 ( 6.9 - 7.71 ) | 9.97 ( 8.59 - 11.5 ) | 4.88 ( 4.66 - 5.12 ) | 51.05 | -1.71 ( -2.01 - -1.41 ) |
| Saint Vincent | Female | 4.18 ( 3.75 - 4.65 ) | 13.22 ( 11.82 - 14.75 ) | 5.37 ( 4.71 - 6.12 ) | 9.21 ( 7.94 - 10.61 ) | 28.51 | -1.15 ( -1.46 - -0.85 ) |
| Samoa | Female | 1.92 ( 1.5 - 2.45 ) | 10.32 ( 9.24 - 11.51 ) | 3 ( 2.33 - 3.72 ) | 8.18 ( 7.17 - 9.34 ) | 56.43 | -0.01 ( -0.14 - 0.11 ) |
| Sao Tome and Principe | Female | 1.62 ( 1.38 - 1.91 ) | 4.08 ( 3.2 - 5.19 ) | 2.96 ( 2.26 - 3.94 ) | 4.04 ( 3.14 - 5 ) | 82.85 | 0.32 ( 0.09 - 0.55 ) |
| Saudi Arabia | Female | 160.44 ( 122.15 - 208.9 ) | 4.23 ( 3.59 - 5.03 ) | 366.19 ( 288.41 - 468.06 ) | 4.88 ( 3.7 - 6.57 ) | 128.24 | -0.98 ( -1.07 - -0.88 ) |
| Senegal | Female | 113.22 ( 91.07 - 137.89 ) | 5.1 ( 3.92 - 6.56 ) | 241.24 ( 181.93 - 312.39 ) | 3.73 ( 3.05 - 4.55 ) | 113.07 | -0.12 ( -0.21 - -0.03 ) |
| Serbia | Female | 811.23 ( 699.19 - 944.38 ) | 6.18 ( 4.94 - 7.53 ) | 915.43 ( 792.68 - 1051.74 ) | 5.83 ( 4.39 - 7.53 ) | 12.84 | -0.18 ( -0.49 - 0.13 ) |
| Seychelles | Female | 7.97 ( 7.04 - 9.08 ) | 12.67 ( 11 - 14.69 ) | 7.88 ( 6.88 - 9.01 ) | 11.82 ( 10.28 - 13.53 ) | -1.16 | -2.06 ( -2.21 - -1.9 ) |
| Sierra Leone | Female | 77.88 ( 60.97 - 99.29 ) | 25.42 ( 22.47 - 28.98 ) | 163.29 ( 124.96 - 208.94 ) | 13.93 ( 12.12 - 15.86 ) | 109.66 | 0.93 ( 0.72 - 1.14 ) |
| Singapore | Female | 71.51 ( 64.99 - 77.81 ) | 7.5 ( 5.88 - 9.56 ) | 62.31 ( 54.74 - 71.71 ) | 8.62 ( 6.63 - 11.13 ) | -12.87 | -4.19 ( -4.54 - -3.85 ) |
| Slovakia | Female | 280.66 ( 251.26 - 314.38 ) | 5.62 ( 5.1 - 6.14 ) | 221.38 ( 186.07 - 263.11 ) | 1.72 ( 1.52 - 1.97 ) | -21.12 | -1.96 ( -2.09 - -1.84 ) |
| Slovenia | Female | 113.57 ( 102.39 - 125.65 ) | 8.53 ( 7.64 - 9.5 ) | 107.12 ( 91.73 - 124.91 ) | 4.92 ( 4.12 - 5.84 ) | -5.68 | -1.99 ( -2.28 - -1.69 ) |
| Solomon Islands | Female | 17.79 ( 13.64 - 23.04 ) | 7.98 ( 7.25 - 8.81 ) | 37.89 ( 29.61 - 47.56 ) | 5.56 ( 4.77 - 6.51 ) | 112.96 | -0.05 ( -0.18 - 0.09 ) |
| Somalia | Female | 430.53 ( 244.38 - 639.07 ) | 21.42 ( 16.8 - 26.82 ) | 667.64 ( 448.22 - 931.74 ) | 20.04 ( 15.84 - 24.7 ) | 55.07 | -1.97 ( -2.2 - -1.74 ) |
| South Africa | Female | 1933.68 ( 1776.88 - 2112.13 ) | 24.86 ( 15.16 - 35.91 ) | 2216.62 ( 2077.86 - 2378.9 ) | 16.74 ( 11.31 - 23.11 ) | 14.63 | -2.37 ( -3.15 - -1.59 ) |
| South Korea | Female | 2862.31 ( 2665.16 - 3068.65 ) | 14.14 ( 12.97 - 15.46 ) | 906.2 ( 793.64 - 1041.79 ) | 8.26 ( 7.74 - 8.86 ) | -68.34 | -8.99 ( -9.69 - -8.29 ) |
| South Sudan | Female | 256.53 ( 155.85 - 377.54 ) | 15.61 ( 14.59 - 16.72 ) | 308.89 ( 211.25 - 437.84 ) | 2.07 ( 1.81 - 2.36 ) | 20.41 | -1.94 ( -2.14 - -1.74 ) |
| Spain | Female | 1375.09 ( 1280.72 - 1470.43 ) | 20.15 ( 12.82 - 29.09 ) | 1837.76 ( 1603.91 - 2083.24 ) | 13.45 ( 9.22 - 19.06 ) | 33.65 | 0.39 ( 0.18 - 0.6 ) |
| Sri Lanka | Female | 273.63 ( 237.87 - 312.68 ) | 4.84 ( 4.48 - 5.2 ) | 867.42 ( 626.51 - 1138.14 ) | 4.62 ( 4.02 - 5.26 ) | 217 | 2.51 ( 2.12 - 2.9 ) |
| Sudan | Female | 1642.21 ( 1215.9 - 2099.22 ) | 4.53 ( 3.95 - 5.16 ) | 2182.1 ( 1508.57 - 2966.2 ) | 6.28 ( 4.58 - 8.17 ) | 32.88 | -1.42 ( -1.46 - -1.39 ) |
| Suriname | Female | 10.8 ( 9.49 - 12.09 ) | 30.17 ( 22.8 - 38.2 ) | 19.41 ( 16.52 - 22.76 ) | 20.7 ( 14.54 - 28.08 ) | 79.75 | -0.95 ( -1.23 - -0.66 ) |
| Swaziland | Female | 33.81 ( 26.3 - 42.24 ) | 7.66 ( 6.72 - 8.61 ) | 43.96 ( 29.28 - 61.33 ) | 6.11 ( 5.2 - 7.13 ) | 30.03 | -0.59 ( -1.2 - 0.04 ) |
| Sweden | Female | 324.14 ( 298.75 - 347.98 ) | 17.55 ( 13.57 - 21.82 ) | 251.61 ( 225.33 - 278.8 ) | 12.1 ( 8.03 - 16.8 ) | -22.38 | -2.06 ( -2.18 - -1.94 ) |
| Switzerland | Female | 390.25 ( 355.32 - 426.06 ) | 4.74 ( 4.37 - 5.08 ) | 295.73 ( 256.24 - 337.71 ) | 2.9 ( 2.61 - 3.21 ) | -24.22 | -2.57 ( -2.72 - -2.41 ) |
| Syria | Female | 256.83 ( 210.56 - 311.24 ) | 7.57 ( 6.88 - 8.3 ) | 346.59 ( 268.22 - 433.98 ) | 3.96 ( 3.43 - 4.53 ) | 34.95 | -2.4 ( -2.79 - -2 ) |
| Tajikistan | Female | 199.26 ( 176.67 - 223.48 ) | 4.78 ( 4.41 - 5.15 ) | 469.11 ( 380.39 - 574.66 ) | 1.83 ( 1.62 - 2.06 ) | 135.42 | 0.05 ( -0.32 - 0.43 ) |
| Tanzania | Female | 1442.61 ( 1048.34 - 1803.05 ) | 11.76 ( 10.45 - 13.15 ) | 1617.47 ( 1251.38 - 2043.88 ) | 13.1 ( 10.77 - 15.91 ) | 12.12 | -2.99 ( -3.32 - -2.65 ) |
| Thailand | Female | 3462.04 ( 3054.28 - 3916.78 ) | 21.23 ( 15.91 - 26.16 ) | 2502.76 ( 2107.79 - 2947.6 ) | 11.01 ( 8.61 - 13.77 ) | -27.71 | -5.28 ( -5.64 - -4.91 ) |
| Timor-Leste | Female | 45.48 ( 35.19 - 58.85 ) | 25.05 ( 19.53 - 32.09 ) | 53.63 ( 40.92 - 68.65 ) | 12.63 ( 9.81 - 16.11 ) | 17.92 | -2.76 ( -2.93 - -2.59 ) |
| Tobago | Female | 41.22 ( 37.13 - 45.8 ) | 8.98 ( 8.08 - 10 ) | 29.72 ( 21.95 - 38.99 ) | 3.19 ( 2.36 - 4.18 ) | -27.92 | -5.22 ( -5.91 - -4.53 ) |
| Togo | Female | 60.26 ( 48.17 - 74.12 ) | 7.52 ( 6.02 - 9.25 ) | 126.59 ( 94.1 - 167.82 ) | 5.72 ( 4.26 - 7.59 ) | 110.08 | -1.02 ( -1.14 - -0.9 ) |
| Tonga | Female | 3.41 ( 2.86 - 4.06 ) | 10.78 ( 9.12 - 12.74 ) | 4.48 ( 3.5 - 5.57 ) | 10.2 ( 7.98 - 12.64 ) | 31.64 | -0.19 ( -0.28 - -0.11 ) |
| Trinidad | Female | 41.22 ( 37.13 - 45.8 ) | 8.98 ( 8.08 - 10 ) | 29.72 ( 21.95 - 38.99 ) | 3.19 ( 2.36 - 4.18 ) | -27.92 | -5.22 ( -5.91 - -4.53 ) |
| Tunisia | Female | 261.05 ( 224.07 - 301.47 ) | 9.52 ( 8.24 - 10.95 ) | 444.15 ( 325.31 - 590.72 ) | 6.9 ( 5.08 - 9.14 ) | 70.14 | -1.44 ( -1.58 - -1.29 ) |
| Turkey | Female | 3421.71 ( 2901.36 - 4037.28 ) | 16.46 ( 13.94 - 19.27 ) | 3463 ( 2926.91 - 4017.54 ) | 7.44 ( 6.29 - 8.61 ) | 1.21 | -3.24 ( -3.58 - -2.89 ) |
| Turkmenistan | Female | 175.37 ( 158.86 - 192.12 ) | 14.3 ( 12.97 - 15.69 ) | 297.85 ( 254.35 - 347.2 ) | 12.79 ( 10.96 - 14.83 ) | 69.84 | -1.02 ( -2.76 - 0.76 ) |
| Uganda | Female | 899.5 ( 710.56 - 1109.04 ) | 22.47 ( 17.94 - 27.62 ) | 914.62 ( 719.26 - 1152.22 ) | 10.13 ( 8 - 12.84 ) | 1.68 | -3.82 ( -4.23 - -3.41 ) |
| UK | Female | 4491.92 ( 4360.44 - 4614.61 ) | 9.61 ( 9.33 - 9.87 ) | 3532.19 ( 3389.62 - 3684.77 ) | 6.15 ( 5.91 - 6.42 ) | -21.37 | -1.96 ( -2.11 - -1.82 ) |
| Ukraine | Female | 2391.47 ( 2216.28 - 2583.43 ) | 5.71 ( 5.29 - 6.16 ) | 1685.01 ( 1503.34 - 1879.39 ) | 4.57 ( 4.06 - 5.1 ) | -29.54 | -1.28 ( -1.61 - -0.95 ) |
| United Arab Emirates | Female | 55.63 ( 38.97 - 83.22 ) | 27.34 ( 19.26 - 40.2 ) | 287.05 ( 205.72 - 396.2 ) | 21.7 ( 16.02 - 29.41 ) | 416 | -0.9 ( -1.11 - -0.68 ) |
| Uruguay | Female | 182.13 ( 164.57 - 201.03 ) | 8.64 ( 7.8 - 9.51 ) | 258.11 ( 211.76 - 309.96 ) | 9.54 ( 7.8 - 11.57 ) | 41.72 | 0.48 ( 0.31 - 0.65 ) |
| USA | Female | 19087.47 ( 18580.16 - 19657.65 ) | 11.64 ( 11.32 - 11.99 ) | 20268.44 ( 19134.99 - 21358.94 ) | 7.5 ( 7.07 - 7.9 ) | 6.19 | -2.06 ( -2.2 - -1.92 ) |
| Uzbekistan | Female | 1046.94 ( 946.97 - 1144.7 ) | 14.86 ( 13.38 - 16.32 ) | 4317.38 ( 3556.66 - 5172.31 ) | 29.44 ( 24.44 - 35.16 ) | 312.38 | 3.01 ( 1.87 - 4.17 ) |
| Vanuatu | Female | 6.05 ( 4.52 - 8.08 ) | 16.54 ( 12.74 - 21.63 ) | 15.27 ( 11.09 - 20.21 ) | 16.67 ( 12.61 - 21.49 ) | 152.35 | 0.11 ( 0.01 - 0.21 ) |
| Venezuela | Female | 1020.9 ( 933.21 - 1113.91 ) | 18.7 ( 17.04 - 20.46 ) | 1560.69 ( 1219.07 - 1918.41 ) | 10.23 ( 8.02 - 12.59 ) | 52.87 | -2.53 ( -2.72 - -2.33 ) |
| Vietnam | Female | 2324.79 ( 1841.45 - 2818.4 ) | 9.48 ( 7.5 - 11.49 ) | 3178.78 ( 2420.22 - 4043.41 ) | 5.94 ( 4.55 - 7.53 ) | 36.73 | -1.99 ( -2.11 - -1.87 ) |
| Virgin Islands | Female | 4.88 ( 4.18 - 5.7 ) | 9.95 ( 8.53 - 11.63 ) | 7.5 ( 6.23 - 8.92 ) | 7.5 ( 6.26 - 9.01 ) | 53.58 | -1.18 ( -1.32 - -1.05 ) |
| Yemen | Female | 1006.98 ( 615.7 - 1396.46 ) | 32.64 ( 20.81 - 44.81 ) | 2046.2 ( 1452.83 - 2794.9 ) | 25.67 ( 18.44 - 34.71 ) | 103.2 | -1.07 ( -1.18 - -0.96 ) |
| Zambia | Female | 593.18 ( 450.28 - 747.47 ) | 32.6 ( 24.97 - 40.82 ) | 569.89 ( 421.02 - 745.72 ) | 14.11 ( 10.41 - 18.5 ) | -3.93 | -4.22 ( -4.84 - -3.61 ) |
| Zimbabwe | Female | 259.59 ( 211.53 - 317.39 ) | 10.59 ( 8.62 - 12.98 ) | 574.56 ( 431.33 - 742.93 ) | 12.62 ( 9.41 - 16.13 ) | 121.34 | 2.07 ( 1.1 - 3.05 ) |
| Afghanistan | Male | 5488.06 ( 2485.12 - 8066.75 ) | 130.49 ( 59.89 - 192.34 ) | 7723.77 ( 5395.44 - 10331.1 ) | 129.83 ( 94.09 - 174.51 ) | 40.74 | -0.02 ( -0.29 - 0.25 ) |
| Albania | Male | 1500.82 ( 1370.49 - 1653.64 ) | 132.75 ( 121.41 - 145.28 ) | 1833.33 ( 1384.43 - 2382.62 ) | 91.36 ( 69.53 - 117.99 ) | 22.16 | -1.15 ( -1.42 - -0.88 ) |
| Algeria | Male | 6205.25 ( 5282.07 - 7192.57 ) | 91.91 ( 77.88 - 106.5 ) | 10000.21 ( 8515.96 - 11580.98 ) | 56.15 ( 48.06 - 64.78 ) | 61.16 | -1.78 ( -1.84 - -1.73 ) |
| American Samoa | Male | 7.7 ( 6.57 - 9.62 ) | 59.22 ( 50.05 - 75.93 ) | 9.54 ( 7.88 - 11.31 ) | 43.22 ( 35.5 - 51.28 ) | 23.99 | -1.19 ( -1.41 - -0.98 ) |
| Andorra | Male | 13.83 ( 9.91 - 19.53 ) | 44.12 ( 31.85 - 62.24 ) | 18.22 ( 14.1 - 24.05 ) | 27.15 ( 21.09 - 35.63 ) | 31.75 | -1.95 ( -2 - -1.9 ) |
| Angola | Male | 2466.44 ( 1493.74 - 3364.81 ) | 105.32 ( 67.73 - 140.13 ) | 4509 ( 3521.26 - 5551.47 ) | 80.51 ( 63.2 - 98.13 ) | 82.81 | -1.17 ( -1.28 - -1.07 ) |
| Antigua | Male | 20.69 ( 18.69 - 23.03 ) | 91.15 ( 82.36 - 101.46 ) | 36.5 ( 31.92 - 41.65 ) | 73.82 ( 64.87 - 84.3 ) | 76.4 | -0.82 ( -0.96 - -0.69 ) |
| Argentina | Male | 23492.53 ( 22308.54 - 24676.45 ) | 151.55 ( 144.06 - 159 ) | 18827.68 ( 16135.37 - 21966.79 ) | 79.73 ( 68.31 - 92.99 ) | -19.86 | -2.84 ( -3.13 - -2.55 ) |
| Armenia | Male | 2933.3 ( 2696.83 - 3203.41 ) | 209.46 ( 192.96 - 227.56 ) | 2283.98 ( 2055.14 - 2535.81 ) | 122.42 ( 110.59 - 134.85 ) | -22.14 | -2.57 ( -2.79 - -2.35 ) |
| Australia | Male | 5360.44 ( 5101.58 - 5647.51 ) | 57.63 ( 54.82 - 60.74 ) | 4739.3 ( 4026 - 5537.46 ) | 26.01 ( 22.12 - 30.38 ) | -11.59 | -3.37 ( -3.59 - -3.16 ) |
| Austria | Male | 4507.31 ( 4256.05 - 4765.85 ) | 96.73 ( 91.39 - 102.33 ) | 2859.33 ( 2545.6 - 3206.97 ) | 39.24 ( 34.79 - 44.04 ) | -36.56 | -3.67 ( -3.83 - -3.52 ) |
| Azerbaijan | Male | 3402.35 ( 3078.69 - 3753.93 ) | 131.98 ( 120.02 - 144.92 ) | 5197.95 ( 4280.28 - 6250.33 ) | 105.69 ( 88.32 - 126.31 ) | 52.78 | -1.83 ( -2.23 - -1.43 ) |
| Bahamas | Male | 110.29 ( 98.64 - 121.84 ) | 144.23 ( 129.63 - 158.65 ) | 241.4 ( 205 - 280.81 ) | 126.93 ( 108.88 - 146.22 ) | 118.87 | -0.37 ( -0.54 - -0.2 ) |
| Bahrain | Male | 72.67 ( 61.82 - 84.59 ) | 78.55 ( 66.84 - 91.99 ) | 123.7 ( 103.1 - 146.94 ) | 24.89 ( 20.76 - 29.52 ) | 70.22 | -5.67 ( -6.25 - -5.09 ) |
| Bangladesh | Male | 55817.19 ( 46002.62 - 67360.44 ) | 195.53 ( 161.48 - 235.6 ) | 60464.84 ( 45265.05 - 77237.05 ) | 89.99 ( 66.63 - 114.89 ) | 8.33 | -2.81 ( -2.9 - -2.71 ) |
| Barbados | Male | 84.23 ( 77.19 - 91.96 ) | 71.62 ( 65.4 - 78.31 ) | 143.38 ( 123.41 - 165.09 ) | 66.15 ( 57.07 - 76.07 ) | 70.22 | -0.33 ( -0.49 - -0.18 ) |
| Barbuda | Male | 20.69 ( 18.69 - 23.03 ) | 91.15 ( 82.36 - 101.46 ) | 36.5 ( 31.92 - 41.65 ) | 73.82 ( 64.87 - 84.3 ) | 76.4 | -0.82 ( -0.96 - -0.69 ) |
| Belarus | Male | 13323.45 ( 12675.22 - 13998.27 ) | 240.84 ( 229.63 - 252.39 ) | 9198.29 ( 8161.47 - 10402.85 ) | 140.39 ( 124.92 - 158.88 ) | -30.96 | -2.93 ( -3.47 - -2.39 ) |
| Belgium | Male | 8605.29 ( 8126.54 - 9110.65 ) | 132.26 ( 124.93 - 140.27 ) | 4394.07 ( 3950.07 - 4924.65 ) | 47.47 ( 42.76 - 53.32 ) | -48.94 | -4.18 ( -4.32 - -4.04 ) |
| Belize | Male | 27.24 ( 24.24 - 30.61 ) | 55.69 ( 49.49 - 62.65 ) | 108.63 ( 97.13 - 120.17 ) | 75.17 ( 67.1 - 82.91 ) | 298.87 | 0.91 ( 0.5 - 1.33 ) |
| Benin | Male | 589.1 ( 471.82 - 715.63 ) | 57.9 ( 46.21 - 70.51 ) | 1343.63 ( 991.04 - 1799.58 ) | 57.13 ( 43.1 - 75.25 ) | 128.08 | 0.24 ( 0.11 - 0.37 ) |
| Bermuda | Male | 38.63 ( 35.11 - 42.45 ) | 132.05 ( 120.02 - 144.93 ) | 49.32 ( 44.22 - 54.95 ) | 89.96 ( 80.78 - 99.9 ) | 27.68 | -1.16 ( -1.34 - -0.98 ) |
| Bhutan | Male | 238.32 ( 174.95 - 350.93 ) | 160.77 ( 119.94 - 237.79 ) | 310.39 ( 215.88 - 486.08 ) | 91.72 ( 64.87 - 144.05 ) | 30.24 | -2.11 ( -2.21 - -2.01 ) |
| Bolivia | Male | 1271.15 ( 1025.61 - 1543.89 ) | 76.58 ( 62.4 - 92.1 ) | 1904.53 ( 1383.62 - 2604.79 ) | 45.34 ( 33.18 - 61.75 ) | 49.83 | -1.99 ( -2.07 - -1.91 ) |
| Bosnia and Herzegovina | Male | 4168.19 ( 3837.16 - 4530.22 ) | 198.49 ( 183.63 - 214.19 ) | 3040.17 ( 2641.32 - 3483.59 ) | 108.5 ( 94.72 - 123.73 ) | -27.06 | -3.05 ( -3.38 - -2.72 ) |
| Botswana | Male | 322.22 ( 256.23 - 410.28 ) | 109.61 ( 87.99 - 138.09 ) | 367.57 ( 280.84 - 532.25 ) | 57.4 ( 44.4 - 82.08 ) | 14.07 | -2.57 ( -2.8 - -2.34 ) |
| Brazil | Male | 59435.79 ( 57855.27 - 61610.21 ) | 124.8 ( 121.64 - 129.52 ) | 114761.53 ( 111163.15 - 118831.83 ) | 104.48 ( 101.28 - 108.1 ) | 93.08 | -0.8 ( -0.91 - -0.68 ) |
| Brunei | Male | 40.87 ( 35.01 - 47.39 ) | 75.74 ( 64.4 - 88.42 ) | 46.56 ( 40.09 - 54.58 ) | 30.08 ( 25.87 - 34.93 ) | 13.93 | -4.01 ( -4.32 - -3.71 ) |
| Bulgaria | Male | 8582.14 ( 8111.12 - 9064.15 ) | 141.32 ( 133.28 - 149.7 ) | 9183.65 ( 8234.79 - 10233.21 ) | 156.6 ( 140.11 - 174.28 ) | 7.01 | 0.35 ( 0.05 - 0.64 ) |
| Burkina Faso | Male | 1328.35 ( 941.9 - 1748.42 ) | 58.5 ( 42.01 - 76.91 ) | 2933.23 ( 1986.59 - 3696.1 ) | 66.99 ( 45.94 - 84.34 ) | 120.82 | 0.72 ( 0.6 - 0.85 ) |
| Burundi | Male | 1686.21 ( 1167.35 - 2255.35 ) | 146.37 ( 101.7 - 194.59 ) | 1926.88 ( 1339.13 - 2632.93 ) | 74.1 ( 52.61 - 99.1 ) | 14.27 | -3.02 ( -3.26 - -2.77 ) |
| Cambodia | Male | 2347.08 ( 1784.64 - 2953.01 ) | 107.78 ( 83.06 - 135.76 ) | 4386.11 ( 3338.5 - 5946.61 ) | 85.36 ( 66.09 - 118.02 ) | 86.87 | -0.8 ( -0.85 - -0.76 ) |
| Cameroon | Male | 1623.46 ( 1270.41 - 2021.29 ) | 66.13 ( 52.33 - 81.83 ) | 4421.71 ( 3177 - 5945.17 ) | 72.32 ( 52.49 - 96.23 ) | 172.36 | 0.55 ( 0.34 - 0.75 ) |
| Canada | Male | 9316.88 ( 8912.81 - 9742.77 ) | 62.18 ( 59.48 - 65.06 ) | 8978 ( 7997.48 - 10030.84 ) | 29.59 ( 26.39 - 32.96 ) | -3.64 | -3.15 ( -3.4 - -2.89 ) |
| Cape Verde | Male | 67.94 ( 56.57 - 79.92 ) | 71.63 ( 59.3 - 84.32 ) | 78.1 ( 66.3 - 91.07 ) | 39.62 ( 33.6 - 46.02 ) | 14.95 | -2.31 ( -2.49 - -2.13 ) |
| Central African Republic | Male | 795.12 ( 440.3 - 1045.87 ) | 122.33 ( 71.72 - 158.19 ) | 1236.02 ( 732.85 - 1722.19 ) | 100.41 ( 65.58 - 136.42 ) | 55.45 | -0.93 ( -1.05 - -0.8 ) |
| Chad | Male | 693.85 ( 452.74 - 889.73 ) | 48.69 ( 31.9 - 62.07 ) | 1912.44 ( 1344.37 - 2492.67 ) | 61.17 ( 43.32 - 78.72 ) | 175.63 | 1.21 ( 0.99 - 1.44 ) |
| Chile | Male | 2721.46 ( 2544.23 - 2919.46 ) | 57.12 ( 53.41 - 61.21 ) | 3063.89 ( 2596.21 - 3553.96 ) | 28.68 ( 24.31 - 33.2 ) | 12.58 | -2.51 ( -2.71 - -2.31 ) |
| China | Male | 242390.93 ( 230780.93 - 257332.91 ) | 51.25 ( 48.93 - 54.5 ) | 393446.28 ( 370636.22 - 417568.96 ) | 39.06 ( 36.86 - 41.38 ) | 62.32 | -1.22 ( -1.5 - -0.93 ) |
| Colombia | Male | 7355.01 ( 6993.66 - 7757.73 ) | 79.25 ( 75.42 - 83.73 ) | 9191.57 ( 7798.41 - 10721.6 ) | 37.11 ( 31.49 - 43.28 ) | 24.97 | -3.57 ( -3.85 - -3.3 ) |
| Comoros | Male | 110.37 ( 81.49 - 151.2 ) | 95.57 ( 70.81 - 129.7 ) | 140.03 ( 98.03 - 210.64 ) | 60.51 ( 42.97 - 90.77 ) | 26.88 | -2 ( -2.22 - -1.79 ) |
| Costa Rica | Male | 667.71 ( 620.81 - 717.32 ) | 74.78 ( 69.51 - 80.38 ) | 1228.97 ( 1064.21 - 1378.47 ) | 53.2 ( 46.3 - 59.57 ) | 84.06 | -1.6 ( -1.86 - -1.34 ) |
| Croatia | Male | 7123.55 ( 6726.94 - 7500.75 ) | 238.39 ( 225.8 - 250.56 ) | 4072.44 ( 3638.15 - 4522.07 ) | 111.92 ( 99.99 - 124.57 ) | -42.83 | -3.05 ( -3.34 - -2.75 ) |
| Cuba | Male | 8145.86 ( 7766.37 - 8554.61 ) | 157.65 ( 150.19 - 165.51 ) | 19428.8 ( 16336.48 - 22672.42 ) | 220.06 ( 185.58 - 256.2 ) | 138.51 | 1.33 ( 1.23 - 1.43 ) |
| Cyprus | Male | 251.86 ( 221.07 - 288.32 ) | 63.61 ( 55.97 - 72.72 ) | 335.1 ( 283.34 - 389.24 ) | 37.59 ( 31.93 - 43.54 ) | 33.05 | -2.08 ( -2.39 - -1.78 ) |
| Czech Republic | Male | 9066.72 ( 8634.89 - 9493.73 ) | 151.03 ( 143.69 - 158.17 ) | 5715.77 ( 5140.53 - 6357.45 ) | 65.29 ( 58.59 - 72.51 ) | -36.96 | -3.1 ( -3.17 - -3.03 ) |
| Democratic Republic of the Congo | Male | 6482.25 ( 4735.6 - 8548 ) | 77.66 ( 58.03 - 100.26 ) | 11637.58 ( 7332.31 - 15671.07 ) | 65.8 ( 43.13 - 86.11 ) | 79.53 | -0.73 ( -0.83 - -0.63 ) |
| Denmark | Male | 2520.75 ( 2385.5 - 2666.86 ) | 74.09 ( 70.07 - 78.41 ) | 1787.43 ( 1585.12 - 1991.55 ) | 36.33 ( 32.32 - 40.46 ) | -29.09 | -3 ( -3.17 - -2.82 ) |
| Djibouti | Male | 90.42 ( 55.67 - 148.22 ) | 89.38 ( 57.15 - 143.62 ) | 239.07 ( 152.28 - 456.98 ) | 64.23 ( 41.97 - 121.92 ) | 164.39 | -1.6 ( -1.79 - -1.41 ) |
| Dominica | Male | 26.05 ( 23.51 - 28.74 ) | 89.77 ( 81.06 - 98.63 ) | 45.31 ( 39.79 - 51.1 ) | 100.19 ( 88.19 - 112.69 ) | 73.94 | 0.3 ( 0.12 - 0.48 ) |
| Dominican Republic | Male | 1291.38 ( 1116.19 - 1485.58 ) | 63.06 ( 54.41 - 72.54 ) | 2836.86 ( 2224.58 - 3472.48 ) | 61.82 ( 48.71 - 75.86 ) | 119.68 | 0.25 ( -0.19 - 0.7 ) |
| Ecuador | Male | 1155.84 ( 1078.65 - 1246.05 ) | 40.56 ( 37.86 - 43.71 ) | 1809.94 ( 1554.83 - 2098.63 ) | 25.02 ( 21.5 - 28.97 ) | 56.59 | -1.49 ( -1.84 - -1.14 ) |
| Egypt | Male | 6568.58 ( 5807.36 - 7457.19 ) | 38.24 ( 34.09 - 43.26 ) | 13035.69 ( 7894.52 - 16439.36 ) | 35.63 ( 21.78 - 44.94 ) | 98.46 | -0.07 ( -0.3 - 0.16 ) |
| El Salvador | Male | 630.98 ( 564.38 - 706.38 ) | 42.08 ( 37.59 - 47.17 ) | 1023.53 ( 799 - 1290.1 ) | 42.16 ( 32.88 - 53.08 ) | 62.21 | 0.16 ( -0.1 - 0.42 ) |
| Equatorial Guinea | Male | 125.32 ( 58.44 - 178.53 ) | 121.74 ( 60.09 - 170.16 ) | 128.39 ( 88.83 - 178.17 ) | 59.53 ( 41.54 - 81.72 ) | 2.45 | -3.21 ( -3.65 - -2.76 ) |
| Eritrea | Male | 948.39 ( 615.46 - 1300.87 ) | 172.35 ( 118.34 - 230.72 ) | 1328.23 ( 920.08 - 2200.62 ) | 105.14 ( 74.97 - 173.49 ) | 40.05 | -2.39 ( -2.73 - -2.04 ) |
| Estonia | Male | 1402.17 ( 1304.1 - 1506.75 ) | 166.79 ( 155.2 - 178.76 ) | 771.52 ( 649.06 - 932.27 ) | 80.7 ( 68.09 - 97.97 ) | -44.98 | -3.31 ( -3.66 - -2.95 ) |
| Ethiopia | Male | 8041.03 ( 5743.42 - 11062.41 ) | 66.29 ( 47.98 - 90.54 ) | 8280.37 ( 5962.53 - 13892.07 ) | 36.6 ( 26.3 - 61.74 ) | 2.98 | -2.47 ( -2.59 - -2.35 ) |
| Fiji | Male | 69.83 ( 58.13 - 82.79 ) | 34.21 ( 28.91 - 40.51 ) | 160.52 ( 132.1 - 195.18 ) | 43.67 ( 36.49 - 52.81 ) | 129.89 | 1.38 ( 1.13 - 1.64 ) |
| Finland | Male | 1131.16 ( 1053.02 - 1208.68 ) | 38.03 ( 35.47 - 40.6 ) | 865.02 ( 767.47 - 989.14 ) | 17.45 ( 15.47 - 19.99 ) | -23.53 | -2.84 ( -2.99 - -2.7 ) |
| France | Male | 77917.41 ( 74486.55 - 81747.33 ) | 227.24 ( 217.36 - 238.52 ) | 35837.41 ( 32196.46 - 39933.93 ) | 68.87 ( 61.68 - 76.76 ) | -54.01 | -4.72 ( -5.13 - -4.31 ) |
| Gabon | Male | 316.19 ( 250.59 - 396.22 ) | 113.57 ( 90.93 - 142.55 ) | 463.81 ( 361.58 - 683.48 ) | 83.05 ( 65.35 - 123.57 ) | 46.69 | -1.18 ( -1.27 - -1.1 ) |
| Gambia | Male | 69.03 ( 51.35 - 90.22 ) | 103.61 ( 91.58 - 115.66 ) | 144.81 ( 109.26 - 192.03 ) | 60.7 ( 51.59 - 71.5 ) | 109.78 | -0.04 ( -0.14 - 0.05 ) |
| Georgia | Male | 5496.4 ( 5051.17 - 5931.32 ) | 32.46 ( 24.3 - 42.05 ) | 4496.88 ( 4025.04 - 4990.52 ) | 30.24 ( 23.3 - 40.16 ) | -18.19 | 0.11 ( -0.49 - 0.72 ) |
| Germany | Male | 48065.59 ( 45807.82 - 50357.26 ) | 193.72 ( 178.49 - 208.98 ) | 34045.96 ( 29191.59 - 39834.89 ) | 183.5 ( 164.74 - 203.07 ) | -29.17 | -3 ( -3.22 - -2.78 ) |
| Ghana | Male | 1898.3 ( 1312.75 - 2463.79 ) | 92.25 ( 87.91 - 96.59 ) | 5520.69 ( 3334.85 - 7041.53 ) | 45.51 ( 38.85 - 53.28 ) | 190.82 | 1.74 ( 1.45 - 2.02 ) |
| Greece | Male | 7011.4 ( 6635.45 - 7424.77 ) | 54.79 ( 38.16 - 70.48 ) | 7009.14 ( 6294.65 - 7796.14 ) | 73.5 ( 45.75 - 92.4 ) | -0.03 | -0.91 ( -1.02 - -0.8 ) |
| Greenland | Male | 17.38 ( 15.35 - 19.81 ) | 97.45 ( 92.39 - 103.13 ) | 26.61 ( 23.31 - 30.32 ) | 74.29 ( 66.41 - 82.63 ) | 53.1 | -1.05 ( -1.31 - -0.79 ) |
| Grenada | Male | 25.3 ( 22.86 - 27.88 ) | 90.8 ( 80.73 - 101.79 ) | 61.01 ( 54.07 - 67.7 ) | 67.74 ( 59.87 - 76.41 ) | 141.13 | 0.28 ( -0.05 - 0.61 ) |
| Grenadines | Male | 36.29 ( 32.64 - 39.98 ) | 136.26 ( 97.09 - 172.68 ) | 92.99 ( 82.48 - 104.7 ) | 65.84 ( 50.81 - 85.44 ) | 156.24 | 0.56 ( 0.39 - 0.73 ) |
| Guam | Male | 26.26 ( 22.3 - 34.65 ) | 86.43 ( 78.07 - 95 ) | 49.19 ( 42.49 - 56.75 ) | 87.24 ( 77.07 - 97.22 ) | 87.34 | -0.57 ( -0.75 - -0.38 ) |
| Guatemala | Male | 973.04 ( 900.37 - 1048.92 ) | 109.34 ( 98.45 - 120.51 ) | 1661.63 ( 1431.68 - 1918.15 ) | 130.84 ( 116.29 - 146.93 ) | 70.77 | -2.28 ( -2.58 - -1.97 ) |
| Guinea | Male | 848.8 ( 683.04 - 1038.88 ) | 60.87 ( 52.35 - 78.6 ) | 1941.09 ( 1413.01 - 2504.63 ) | 54.3 ( 47.29 - 62.52 ) | 128.69 | 1.81 ( 1.63 - 1.99 ) |
| Guinea-Bissau | Male | 198.35 ( 129.92 - 257.05 ) | 47.15 ( 43.63 - 50.77 ) | 296.9 ( 218.16 - 381.58 ) | 31.36 ( 27.07 - 36.18 ) | 49.68 | -0.11 ( -0.23 - 0.01 ) |
| Guyana | Male | 108.15 ( 98.52 - 119.31 ) | 47.99 ( 38.73 - 59.02 ) | 172.75 ( 142.88 - 205.22 ) | 67.76 ( 49.92 - 86.87 ) | 59.72 | 0.56 ( 0.28 - 0.83 ) |
| Haiti | Male | 2779.12 ( 2047.85 - 3908.38 ) | 90.91 ( 60.67 - 116.91 ) | 4412.22 ( 3064.24 - 6998.07 ) | 81.44 ( 61.09 - 103.47 ) | 58.76 | -0.5 ( -0.63 - -0.36 ) |
| Honduras | Male | 641.44 ( 528.99 - 762.23 ) | 53.07 ( 48.12 - 58.35 ) | 1339.75 ( 1003.47 - 1734.27 ) | 53.2 ( 44.3 - 62.84 ) | 108.87 | -0.76 ( -0.87 - -0.66 ) |
| Hungary | Male | 16440.2 ( 15641.71 - 17315.22 ) | 155.18 ( 115.01 - 221.92 ) | 11477.4 ( 10391.06 - 12697.37 ) | 130.12 ( 91.05 - 207.86 ) | -30.19 | -2.12 ( -2.46 - -1.78 ) |
| Iceland | Male | 45.72 ( 41.58 - 50.31 ) | 52.79 ( 43.97 - 62.42 ) | 52.79 ( 47.42 - 58.59 ) | 44.27 ( 33.3 - 56.82 ) | 15.46 | -2.47 ( -2.97 - -1.97 ) |
| India | Male | 497892.51 ( 420660.8 - 557652.51 ) | 256.92 ( 244.41 - 270.29 ) | 744888.4 ( 696456.46 - 794265.93 ) | 153.74 ( 139.36 - 169.8 ) | 49.61 | -1.16 ( -1.39 - -0.93 ) |
| Indonesia | Male | 32311.37 ( 26741.51 - 45972.46 ) | 35.07 ( 31.84 - 38.69 ) | 64221.9 ( 49787.91 - 106334.16 ) | 21.7 ( 19.51 - 24.05 ) | 98.76 | 0.07 ( -0.04 - 0.17 ) |
| Iran | Male | 14447.25 ( 13014.84 - 17104.23 ) | 170.61 ( 144.44 - 190.76 ) | 24931.93 ( 23701.1 - 26384.98 ) | 127.2 ( 118.49 - 135.6 ) | 72.57 | -0.6 ( -0.91 - -0.28 ) |
| Iraq | Male | 5458.86 ( 4056.82 - 6867.54 ) | 59.55 ( 49.43 - 84.21 ) | 5539.86 ( 4912.07 - 6237.03 ) | 58.99 ( 46.13 - 96.54 ) | 1.48 | -4.39 ( -5 - -3.78 ) |
| Ireland | Male | 1191.38 ( 1100.83 - 1290.53 ) | 91.95 ( 83.01 - 109.28 ) | 1240.31 ( 1085.24 - 1406.05 ) | 67.49 ( 64.1 - 71.34 ) | 4.11 | -1.99 ( -2.15 - -1.82 ) |
| Israel | Male | 852.88 ( 785.34 - 937.64 ) | 128.56 ( 96.21 - 158.79 ) | 1636.47 ( 1461.65 - 1831.85 ) | 43.16 ( 38.58 - 48.24 ) | 91.87 | -1.28 ( -1.7 - -0.85 ) |
| Italy | Male | 56580.71 ( 54209.04 - 59128.96 ) | 63.26 ( 58.45 - 68.45 ) | 28819.63 ( 25959.43 - 31915.86 ) | 36.73 ( 32.17 - 41.51 ) | -49.06 | -3.91 ( -4.03 - -3.79 ) |
| Ivory Coast | Male | 1879.83 ( 1519.97 - 2316.65 ) | 38.4 ( 35.37 - 42.29 ) | 4101.22 ( 3048.81 - 5520.14 ) | 33.13 ( 29.54 - 37.09 ) | 118.17 | -0.45 ( -0.71 - -0.19 ) |
| Jamaica | Male | 426.05 ( 385.44 - 475.43 ) | 144.63 ( 138.72 - 150.98 ) | 1168.93 ( 873.19 - 1463.74 ) | 50.56 ( 45.53 - 56.09 ) | 174.36 | 1.1 ( 0.48 - 1.72 ) |
| Japan | Male | 19250.51 ( 18579.49 - 20088.81 ) | 69.18 ( 56.21 - 84.04 ) | 19557.29 ( 18430.32 - 20753.59 ) | 64.11 ( 48.38 - 85.77 ) | 1.59 | -2.54 ( -2.63 - -2.45 ) |
| Jordan | Male | 700.6 ( 548.1 - 860.75 ) | 51.37 ( 46.43 - 57.23 ) | 868 ( 729.46 - 1053.93 ) | 83.38 ( 62.38 - 104.17 ) | 23.89 | -5.12 ( -5.64 - -4.6 ) |
| Kazakhstan | Male | 11178.52 ( 10521.83 - 11917.93 ) | 24.9 ( 24.04 - 25.99 ) | 6025.24 ( 5413.87 - 6739.09 ) | 13.45 ( 12.65 - 14.29 ) | -46.1 | -3.92 ( -4.25 - -3.59 ) |
| Kenya | Male | 3551.03 ( 2009.09 - 4513.12 ) | 81.86 ( 64.23 - 101.02 ) | 9718.77 ( 6766 - 11422.17 ) | 25.23 ( 21.15 - 30.59 ) | 173.69 | 0.3 ( -0.02 - 0.63 ) |
| Kiribati | Male | 5.2 ( 4.5 - 5.98 ) | 185.75 ( 175.35 - 196.74 ) | 10.74 ( 8.52 - 13.3 ) | 76.81 ( 69.6 - 85.26 ) | 106.38 | 0.36 ( 0.01 - 0.72 ) |
| Kuwait | Male | 166.71 ( 151.01 - 183.37 ) | 76.07 ( 42.86 - 96.24 ) | 256.47 ( 226.01 - 294.12 ) | 81.36 ( 56.4 - 95.36 ) | 53.84 | -2.29 ( -2.69 - -1.89 ) |
| Kyrgyzstan | Male | 1717.17 ( 1548.9 - 1901.89 ) | 29.5 ( 25.48 - 33.66 ) | 809.33 ( 716.12 - 926.56 ) | 34.23 ( 27.42 - 41.34 ) | -52.87 | -4.38 ( -4.74 - -4.02 ) |
| Laos | Male | 1212.3 ( 907.61 - 1565.94 ) | 34.5 ( 31.27 - 37.79 ) | 1524.69 ( 1063.32 - 2567.5 ) | 16.39 ( 14.57 - 18.59 ) | 25.77 | -1.71 ( -1.75 - -1.67 ) |
| Latvia | Male | 2737.36 ( 2571.49 - 2918.91 ) | 122.21 ( 110.43 - 134.99 ) | 1885.25 ( 1593.84 - 2223.41 ) | 37.66 ( 33.57 - 42.82 ) | -31.13 | -1.49 ( -1.89 - -1.09 ) |
| Lebanon | Male | 2178.32 ( 1727.94 - 2793.97 ) | 107.5 ( 81.01 - 138.23 ) | 2620.36 ( 2277.57 - 3017.64 ) | 68.91 ( 47.95 - 119.18 ) | 20.29 | -2.88 ( -3.16 - -2.61 ) |
| Lesotho | Male | 672.28 ( 469.98 - 848.75 ) | 184.71 ( 174.02 - 196.64 ) | 803.72 ( 613.54 - 1035.67 ) | 132.73 ( 112.48 - 156.63 ) | 19.55 | 0.76 ( 0.4 - 1.11 ) |
| Liberia | Male | 333.93 ( 259.84 - 428.49 ) | 191.3 ( 152.4 - 247.05 ) | 518.37 ( 363.72 - 689.11 ) | 90.3 ( 78.7 - 104.03 ) | 55.23 | -0.09 ( -0.23 - 0.06 ) |
| Libya | Male | 1600.06 ( 1232.91 - 2066.52 ) | 135.14 ( 93.41 - 170.46 ) | 3432.46 ( 2263.26 - 4437.51 ) | 148.77 ( 114.46 - 190.42 ) | 114.52 | -0.45 ( -0.53 - -0.36 ) |
| Lithuania | Male | 3862.41 ( 3644.87 - 4069.99 ) | 51.31 ( 39.77 - 65.67 ) | 3310.27 ( 2976.16 - 3686.79 ) | 48.77 ( 34.45 - 65.2 ) | -14.3 | -1.15 ( -1.55 - -0.76 ) |
| Luxembourg | Male | 297.61 ( 271.01 - 328.75 ) | 139.31 ( 108.24 - 180.02 ) | 209.8 ( 177.92 - 247.51 ) | 126.95 ( 84.39 - 161.75 ) | -29.5 | -3.84 ( -4 - -3.68 ) |
| Macedonia | Male | 1562.28 ( 1395.43 - 1780.05 ) | 198.08 ( 187.42 - 208.43 ) | 2475.5 ( 2130.69 - 2907.1 ) | 159.97 ( 143.52 - 177.92 ) | 58.45 | -0.41 ( -0.67 - -0.15 ) |
| Madagascar | Male | 2415.51 ( 1951.26 - 3014.35 ) | 123.06 ( 112.08 - 136.08 ) | 3714.03 ( 2716.25 - 4893.46 ) | 47.15 ( 39.95 - 55.64 ) | 53.76 | -1.24 ( -1.34 - -1.14 ) |
| Malawi | Male | 700.66 ( 356.64 - 988.9 ) | 157.51 ( 141.23 - 178.88 ) | 1209.56 ( 966.01 - 1520.91 ) | 151.79 ( 131.03 - 178.62 ) | 72.63 | -0.29 ( -0.66 - 0.08 ) |
| Malaysia | Male | 3442.12 ( 2996.17 - 3991.74 ) | 80.59 ( 65.02 - 99.82 ) | 6358.7 ( 4785.59 - 7702.46 ) | 58.94 ( 43.97 - 76.17 ) | 84.73 | -1.88 ( -2.2 - -1.56 ) |
| Maldives | Male | 41.03 ( 27.45 - 49.78 ) | 32.43 ( 17.24 - 45.74 ) | 48.51 ( 42.28 - 55.5 ) | 33.14 ( 26.88 - 41 ) | 18.24 | -3.53 ( -3.73 - -3.34 ) |
| Mali | Male | 1076.72 ( 884.03 - 1286.09 ) | 71.53 ( 62.55 - 82.76 ) | 1537.85 ( 1159.75 - 2008.93 ) | 47.53 ( 36.14 - 57.43 ) | 42.83 | -1.13 ( -1.39 - -0.86 ) |
| Malta | Male | 183.28 ( 168.06 - 200.73 ) | 69.92 ( 49.14 - 83.48 ) | 187.84 ( 167.24 - 210.11 ) | 30.61 ( 26.48 - 35.26 ) | 2.49 | -2.81 ( -3 - -2.63 ) |
| Marshall Islands | Male | 7.17 ( 4.44 - 8.82 ) | 46.78 ( 38.28 - 55.86 ) | 14.51 ( 10.58 - 18.66 ) | 32.76 ( 25.01 - 42.37 ) | 102.26 | 0.06 ( -0.09 - 0.21 ) |
| Mauritania | Male | 283.94 ( 224.09 - 351.56 ) | 92.21 ( 84.52 - 100.87 ) | 452.17 ( 312.98 - 662.52 ) | 48.96 ( 43.57 - 54.87 ) | 59.25 | -0.56 ( -0.79 - -0.32 ) |
| Mauritius | Male | 329.63 ( 299.79 - 358.56 ) | 76.37 ( 47.83 - 94.54 ) | 403.64 ( 353.09 - 460.03 ) | 79.28 ( 59.46 - 99.2 ) | 22.45 | -2.2 ( -2.37 - -2.03 ) |
| Mexico | Male | 14948.53 ( 14556.45 - 15582.28 ) | 54.63 ( 43.26 - 67.68 ) | 20756.31 ( 18971.98 - 21578.11 ) | 43.73 ( 30.42 - 63.4 ) | 38.85 | -2.5 ( -2.63 - -2.36 ) |
| Micronesia | Male | 17.31 ( 13.29 - 23.26 ) | 89.66 ( 82.01 - 96.93 ) | 21.51 ( 16.03 - 27.76 ) | 49.5 ( 43.67 - 56.04 ) | 24.29 | -0.36 ( -0.38 - -0.33 ) |
| Moldova | Male | 4207.24 ( 3932.59 - 4481.62 ) | 68.49 ( 66.73 - 71.41 ) | 4394.86 ( 4009.64 - 4809.32 ) | 38.38 ( 35.09 - 39.88 ) | 4.46 | -0.41 ( -0.79 - -0.03 ) |
| Mongolia | Male | 152.89 ( 133.68 - 173.39 ) | 66.41 ( 51.04 - 90.47 ) | 459.92 ( 385.12 - 566.55 ) | 59.65 ( 46.97 - 74.66 ) | 200.82 | 2.56 ( 2 - 3.13 ) |
| Montenegro | Male | 666.39 ( 568.57 - 790.87 ) | 201.05 ( 188.18 - 214.06 ) | 806.72 ( 681.7 - 961 ) | 175.22 ( 160.11 - 192.11 ) | 21.06 | -0.97 ( -1.22 - -0.73 ) |
| Morocco | Male | 10039.16 ( 8313.97 - 12040.06 ) | 32.75 ( 28.8 - 36.92 ) | 19089.18 ( 14491.89 - 24842.09 ) | 54.02 ( 45.65 - 65.05 ) | 90.15 | -0.64 ( -0.78 - -0.5 ) |
| Mozambique | Male | 3589.21 ( 2504.69 - 4711.79 ) | 212.96 ( 182.73 - 254.52 ) | 5767.06 ( 3429.66 - 7696.61 ) | 173.45 ( 146.35 - 206.18 ) | 60.68 | -0.12 ( -0.23 - -0.01 ) |
| Myanmar | Male | 13189.1 ( 9821.16 - 17262.76 ) | 130.49 ( 108.82 - 158.55 ) | 15864.12 ( 12447.87 - 23141.36 ) | 111.3 ( 84.66 - 144.85 ) | 20.28 | -1.24 ( -1.28 - -1.19 ) |
| Namibia | Male | 573.94 ( 450.24 - 714.81 ) | 102.99 ( 72.49 - 134.36 ) | 841.73 ( 683.17 - 1036.82 ) | 95.67 ( 58.22 - 125.44 ) | 46.66 | -0.96 ( -1.4 - -0.52 ) |
| Nepal | Male | 9307.9 ( 7001.15 - 12217.29 ) | 106.73 ( 80.31 - 139.14 ) | 12569.52 ( 9415.08 - 16618.73 ) | 74.8 ( 59.21 - 110.22 ) | 35.04 | -1.33 ( -1.7 - -0.96 ) |
| Netherlands | Male | 5530.53 ( 5235 - 5829.43 ) | 155.92 ( 123.3 - 193.16 ) | 4603.95 ( 4151.13 - 5054.24 ) | 127.49 ( 104.81 - 154.76 ) | -16.75 | -3.13 ( -3.37 - -2.9 ) |
| New Zealand | Male | 784.2 ( 720.65 - 853.81 ) | 162.87 ( 124.57 - 212.47 ) | 703.69 ( 630.98 - 786.39 ) | 113.77 ( 85.66 - 149.85 ) | -10.27 | -3.12 ( -3.28 - -2.95 ) |
| Nicaragua | Male | 393.5 ( 341 - 450.86 ) | 63.1 ( 59.71 - 66.49 ) | 664.73 ( 550.12 - 797.16 ) | 30.7 ( 27.62 - 33.81 ) | 68.93 | -1.82 ( -1.99 - -1.66 ) |
| Niger | Male | 844.38 ( 526.44 - 1139.56 ) | 42.98 ( 39.5 - 46.72 ) | 1933.88 ( 1010.83 - 2758.98 ) | 20.75 ( 18.6 - 23.15 ) | 129.03 | 0.07 ( -0.08 - 0.22 ) |
| Nigeria | Male | 18971.49 ( 13517.21 - 25887.31 ) | 48.72 ( 42.28 - 55.75 ) | 24599.46 ( 17409.82 - 35428.38 ) | 31.13 ( 25.81 - 37.22 ) | 29.67 | -1.08 ( -1.18 - -0.98 ) |
| North Korea | Male | 4348.31 ( 3124.89 - 5719.42 ) | 49.61 ( 31.27 - 66.26 ) | 8065.91 ( 6097.93 - 10362.65 ) | 48.88 ( 25.95 - 68.95 ) | 85.5 | 0.01 ( -0.12 - 0.13 ) |
| Northern Mariana Islands | Male | 9.17 ( 7.09 - 13.11 ) | 69.46 ( 50.16 - 94.13 ) | 16.52 ( 13.69 - 19.61 ) | 54.99 ( 39.36 - 78.31 ) | 80.2 | -0.46 ( -0.63 - -0.29 ) |
| Norway | Male | 959.57 ( 923.41 - 996.99 ) | 57.3 ( 42.62 - 73.41 ) | 708.54 ( 667.55 - 760.1 ) | 56.26 ( 43.35 - 70.85 ) | -26.16 | -2.82 ( -2.93 - -2.7 ) |
| Oman | Male | 206.78 ( 154.43 - 273.63 ) | 74.49 ( 59.47 - 104.32 ) | 350.05 ( 258.34 - 451.4 ) | 63.35 ( 53.34 - 73.7 ) | 69.29 | -2.29 ( -2.42 - -2.16 ) |
| Pakistan | Male | 69457.76 ( 58542.98 - 81059.24 ) | 33.96 ( 32.71 - 35.25 ) | 139723.26 ( 103852.5 - 181471.06 ) | 16.87 ( 15.9 - 18.08 ) | 101.16 | -0.2 ( -0.45 - 0.06 ) |
| Palestine | Male | 217.96 ( 147.95 - 281.6 ) | 46.4 ( 35.04 - 60.44 ) | 392.33 ( 338.43 - 462.18 ) | 24.47 ( 18.42 - 31.32 ) | 80 | -2.21 ( -2.42 - -1.99 ) |
| Panama | Male | 722.98 ( 666.42 - 783.56 ) | 209.65 ( 177.51 - 244.96 ) | 894.74 ( 786.72 - 1004.06 ) | 209.56 ( 157.46 - 268.43 ) | 23.76 | -2.92 ( -3.24 - -2.6 ) |
| Papua New Guinea | Male | 915.43 ( 700.98 - 1179.19 ) | 52.18 ( 35.6 - 67.65 ) | 2188.14 ( 1671.22 - 2869.39 ) | 30.5 ( 26.39 - 35.9 ) | 139.03 | 0.14 ( 0.05 - 0.23 ) |
| Paraguay | Male | 738.3 ( 623.21 - 893.43 ) | 90.26 ( 83.23 - 97.8 ) | 1864.97 ( 1449.63 - 2355.1 ) | 45.91 ( 40.47 - 51.57 ) | 152.6 | 0.36 ( 0.19 - 0.54 ) |
| Peru | Male | 3052.82 ( 2650.91 - 3659.21 ) | 77.88 ( 61.08 - 97.5 ) | 2811.64 ( 2250.78 - 3424.93 ) | 77.41 ( 60.61 - 99.2 ) | -7.9 | -4.04 ( -4.37 - -3.71 ) |
| Philippines | Male | 7677.67 ( 7005.08 - 8456.67 ) | 64.13 ( 54.37 - 77.19 ) | 16137.87 ( 13210.51 - 19362.74 ) | 68.49 ( 53.44 - 86.26 ) | 110.19 | 0.07 ( -0.04 - 0.17 ) |
| Poland | Male | 46924.4 ( 44909.66 - 49094.56 ) | 48 ( 41.63 - 57.6 ) | 37250.58 ( 33647.52 - 41175.91 ) | 18.83 ( 15.04 - 22.89 ) | -20.62 | -2.67 ( -2.86 - -2.48 ) |
| Portugal | Male | 10618.89 ( 10085.92 - 11190.28 ) | 44.19 ( 40.46 - 48.5 ) | 9205.82 ( 8191.95 - 10323.98 ) | 43.99 ( 36.2 - 52.68 ) | -13.31 | -1.94 ( -2.21 - -1.68 ) |
| Puerto Rico | Male | 1979.98 ( 1850.14 - 2134.03 ) | 232.55 ( 222.86 - 243.14 ) | 1393.85 ( 1250.71 - 1554.61 ) | 125.25 ( 113.29 - 138.51 ) | -29.6 | -3.77 ( -4 - -3.54 ) |
| Qatar | Male | 32.95 ( 25.9 - 40.39 ) | 172.82 ( 164.14 - 182.21 ) | 217.61 ( 168.22 - 267.58 ) | 105.77 ( 93.46 - 118.87 ) | 560.34 | -0.59 ( -1.46 - 0.3 ) |
| Republic of Congo | Male | 685.43 ( 515.1 - 849.22 ) | 115.54 ( 108.05 - 124.54 ) | 1032.26 ( 771.4 - 1503.89 ) | 49.09 ( 43.93 - 54.95 ) | 50.6 | -2.17 ( -2.36 - -1.97 ) |
| Romania | Male | 23671.9 ( 22522.43 - 25053.74 ) | 42.07 ( 33.77 - 51.12 ) | 25596.63 ( 23294.79 - 28281.34 ) | 30.81 ( 24.07 - 38.48 ) | 8.13 | -0.31 ( -0.55 - -0.07 ) |
| Russia | Male | 166225.24 ( 159845.05 - 177834.92 ) | 124.82 ( 96.44 - 152.06 ) | 110458.21 ( 107231.13 - 113730.91 ) | 76.08 ( 57.54 - 111.94 ) | -33.55 | -3.08 ( -3.52 - -2.63 ) |
| Rwanda | Male | 2059.03 ( 1449.11 - 2631.09 ) | 172 ( 163.85 - 181.93 ) | 1859.68 ( 1427.66 - 2445.64 ) | 169.71 ( 154.09 - 187.02 ) | -9.68 | -3.54 ( -3.92 - -3.15 ) |
| Saint Lucia | Male | 48.3 ( 43.83 - 53.22 ) | 217.79 ( 210.14 - 233.19 ) | 103.1 ( 90.72 - 116.81 ) | 116.3 ( 112.83 - 119.76 ) | 113.45 | -0.79 ( -0.96 - -0.62 ) |
| Saint Vincent | Male | 36.29 ( 32.64 - 39.98 ) | 117.95 ( 107.18 - 129.98 ) | 92.99 ( 82.48 - 104.7 ) | 98.75 ( 86.78 - 111.63 ) | 156.24 | 0.56 ( 0.39 - 0.73 ) |
| Samoa | Male | 12.12 ( 9.92 - 14.92 ) | 109.34 ( 98.45 - 120.51 ) | 14.91 ( 11.79 - 18.01 ) | 130.84 ( 116.29 - 146.93 ) | 23.06 | -0.85 ( -0.94 - -0.76 ) |
| Sao Tome and Principe | Male | 10.97 ( 9.18 - 13.22 ) | 27.87 ( 23 - 34.09 ) | 20.58 ( 15.63 - 26.06 ) | 22.7 ( 17.34 - 26.88 ) | 87.53 | 0.86 ( 0.8 - 0.92 ) |
| Saudi Arabia | Male | 1711.16 ( 1116.78 - 2294.23 ) | 33.5 ( 28.32 - 40.29 ) | 3354.97 ( 2661.2 - 4237.11 ) | 41.49 ( 32.07 - 51.96 ) | 96.06 | -1.24 ( -1.57 - -0.9 ) |
| Senegal | Male | 1024.49 ( 831.52 - 1251.23 ) | 41.67 ( 27.86 - 55.35 ) | 2316.74 ( 1776.87 - 3041.19 ) | 27.86 ( 23.14 - 34 ) | 126.14 | 0.64 ( 0.43 - 0.86 ) |
| Serbia | Male | 12331.78 ( 10729.87 - 14772.4 ) | 57.95 ( 47.05 - 70.62 ) | 9493.41 ( 8366.25 - 10748.05 ) | 63.03 ( 48.67 - 81.93 ) | -23.02 | -1.57 ( -1.83 - -1.32 ) |
| Seychelles | Male | 77.46 ( 66.21 - 87.81 ) | 204.78 ( 178.85 - 245.09 ) | 123.11 ( 108.27 - 139.17 ) | 134.18 ( 118.49 - 152.4 ) | 58.92 | -1.43 ( -1.56 - -1.3 ) |
| Sierra Leone | Male | 583.56 ( 404.97 - 746.82 ) | 295.71 ( 252.72 - 335.18 ) | 1101.62 ( 856.04 - 1412.5 ) | 221.06 ( 194.8 - 248.26 ) | 88.78 | 0.56 ( 0.37 - 0.74 ) |
| Singapore | Male | 630.26 ( 582.18 - 685.2 ) | 55.69 ( 38.47 - 71.11 ) | 523.94 ( 462.35 - 591.32 ) | 59.69 ( 47 - 76.13 ) | -16.87 | -4.73 ( -4.92 - -4.53 ) |
| Slovakia | Male | 6210.16 ( 5756.59 - 6637.58 ) | 55.13 ( 50.91 - 59.91 ) | 4318.1 ( 3740.33 - 5018.96 ) | 16.2 ( 14.23 - 18.27 ) | -30.47 | -3.01 ( -3.15 - -2.87 ) |
| Slovenia | Male | 1803.37 ( 1684.85 - 1952.6 ) | 232.58 ( 215.07 - 248.76 ) | 1082.38 ( 956.28 - 1219.99 ) | 105.99 ( 92.37 - 122.9 ) | -39.98 | -4.05 ( -4.2 - -3.9 ) |
| Solomon Islands | Male | 56.01 ( 42.76 - 70.47 ) | 163.43 ( 152.87 - 176.08 ) | 108.55 ( 85.04 - 136.59 ) | 60.24 ( 53.15 - 67.89 ) | 93.8 | -0.07 ( -0.15 - 0.02 ) |
| Somalia | Male | 1532.24 ( 751.6 - 2461.78 ) | 63.06 ( 48.79 - 78.6 ) | 2788.16 ( 2006.68 - 3752.65 ) | 59.21 ( 46.91 - 74.34 ) | 81.97 | -1.31 ( -1.57 - -1.05 ) |
| South Africa | Male | 10571.33 ( 9125.22 - 13673.7 ) | 94.65 ( 52.36 - 143.79 ) | 15270.52 ( 14225.62 - 16825.89 ) | 77.08 ( 56.4 - 102.02 ) | 44.45 | -1.59 ( -2.28 - -0.9 ) |
| South Korea | Male | 15244.3 ( 14446.19 - 16138.5 ) | 103.37 ( 88.82 - 136.14 ) | 8338.55 ( 7366.1 - 9462.12 ) | 75.52 ( 70.54 - 82.83 ) | -45.3 | -7.25 ( -7.87 - -6.63 ) |
| South Sudan | Male | 1319.03 ( 695.85 - 2090.2 ) | 105.69 ( 100.56 - 111.85 ) | 1666.83 ( 1181.81 - 2373.11 ) | 21.6 ( 19.15 - 24.39 ) | 26.37 | -1.17 ( -1.38 - -0.96 ) |
| Spain | Male | 52965.18 ( 50740.14 - 55387.32 ) | 84.67 ( 45.99 - 130.04 ) | 30965.88 ( 28159.3 - 34067.33 ) | 69.52 ( 50.22 - 96.95 ) | -41.54 | -4.17 ( -4.38 - -3.96 ) |
| Sri Lanka | Male | 2096.81 ( 1867.87 - 2355.3 ) | 218.92 ( 209.81 - 228.88 ) | 3975.01 ( 3129.05 - 4949.92 ) | 80.37 ( 72.92 - 88.71 ) | 89.57 | 0.81 ( 0.36 - 1.26 ) |
| Sudan | Male | 4418.3 ( 3050.95 - 5942.37 ) | 34.68 ( 31.03 - 38.9 ) | 7127.85 ( 5198.46 - 9813.91 ) | 33.72 ( 26.76 - 41.65 ) | 61.33 | -0.68 ( -0.73 - -0.62 ) |
| Suriname | Male | 50.61 ( 44.6 - 57.35 ) | 83.08 ( 58.66 - 111.4 ) | 133 ( 111.55 - 157.87 ) | 68.39 ( 49.4 - 93.98 ) | 162.8 | 0.76 ( 0.54 - 0.99 ) |
| Swaziland | Male | 218.84 ( 169.42 - 273.17 ) | 37.55 ( 33.23 - 42.21 ) | 339.14 ( 240.93 - 432.54 ) | 45.79 ( 38.61 - 54.23 ) | 54.97 | -0.03 ( -0.6 - 0.54 ) |
| Sweden | Male | 1511.33 ( 1419.66 - 1614 ) | 141.2 ( 110.03 - 174.17 ) | 1151.42 ( 1044.92 - 1271.89 ) | 125.09 ( 89.29 - 157.75 ) | -23.81 | -2.25 ( -2.32 - -2.18 ) |
| Switzerland | Male | 2780.15 ( 2614.1 - 2963.96 ) | 23.64 ( 22.21 - 25.22 ) | 1737.59 ( 1524.26 - 1989.51 ) | 12.87 ( 11.65 - 14.21 ) | -37.5 | -3.41 ( -3.54 - -3.28 ) |
| Syria | Male | 1200.03 ( 1012.85 - 1429.5 ) | 63.13 ( 59.29 - 67.45 ) | 2126.62 ( 1659.25 - 2726.76 ) | 23.86 ( 20.91 - 27.28 ) | 77.21 | -1.44 ( -1.81 - -1.08 ) |
| Tajikistan | Male | 794.65 ( 711.55 - 893.31 ) | 50.8 ( 48.35 - 53.34 ) | 675.95 ( 571.02 - 797.89 ) | 36.05 ( 32.45 - 39.98 ) | -14.94 | -3.25 ( -3.58 - -2.92 ) |
| Tanzania | Male | 4892.82 ( 3204.37 - 7138.47 ) | 54.84 ( 49.29 - 61.22 ) | 7751.87 ( 5804.39 - 11565.24 ) | 23.98 ( 20.21 - 28.2 ) | 58.43 | -1.46 ( -1.65 - -1.27 ) |
| Thailand | Male | 19657.84 ( 17353.35 - 22137.91 ) | 80.29 ( 53.13 - 115.81 ) | 28557.88 ( 24092.44 - 33771.56 ) | 59.72 ( 45.24 - 88.2 ) | 45.27 | -2.47 ( -2.72 - -2.22 ) |
| Timor-Leste | Male | 107.09 ( 79.18 - 163.27 ) | 61.04 ( 46.18 - 92.2 ) | 248.03 ( 170.1 - 421.27 ) | 59.56 ( 41.42 - 100.49 ) | 131.6 | 0.03 ( -0.16 - 0.22 ) |
| Tobago | Male | 293.72 ( 268.6 - 319.2 ) | 68.77 ( 62.87 - 74.79 ) | 456.41 ( 352.88 - 587.89 ) | 51.84 ( 40.05 - 66.31 ) | 55.39 | -1.28 ( -1.53 - -1.04 ) |
| Togo | Male | 371.5 ( 293.46 - 441.99 ) | 55.87 ( 44.32 - 65.94 ) | 1104.07 ( 805.05 - 1437.19 ) | 64.53 ( 48.21 - 81.99 ) | 197.2 | 0.84 ( 0.67 - 1.01 ) |
| Tonga | Male | 11.18 ( 9.25 - 15 ) | 39.69 ( 33.02 - 52.99 ) | 16.91 ( 13.25 - 22.52 ) | 44.06 ( 34.82 - 58.51 ) | 51.26 | 0.57 ( 0.49 - 0.66 ) |
| Trinidad | Male | 293.72 ( 268.6 - 319.2 ) | 68.77 ( 62.87 - 74.79 ) | 456.41 ( 352.88 - 587.89 ) | 51.84 ( 40.05 - 66.31 ) | 55.39 | -1.28 ( -1.53 - -1.04 ) |
| Tunisia | Male | 3330.3 ( 2813.23 - 4039.36 ) | 117.87 ( 100.29 - 142.05 ) | 6097.68 ( 4484.35 - 7924.24 ) | 97.66 ( 72.45 - 126.82 ) | 83.1 | -1.01 ( -1.18 - -0.83 ) |
| Turkey | Male | 30442.9 ( 25948.68 - 36583.92 ) | 161.15 ( 138.09 - 193.72 ) | 31669.04 ( 27088.33 - 36347.53 ) | 76.18 ( 65.38 - 87.37 ) | 4.03 | -3.16 ( -3.52 - -2.8 ) |
| Turkmenistan | Male | 1188.01 ( 1094.77 - 1287.13 ) | 122.26 ( 113.03 - 132.22 ) | 757.62 ( 633.27 - 865.59 ) | 38.48 ( 32.64 - 43.54 ) | -36.23 | -5.01 ( -5.41 - -4.62 ) |
| Uganda | Male | 2107.1 ( 1557.76 - 2768.61 ) | 57.68 ( 42.77 - 75.57 ) | 4338.92 ( 3325.56 - 6065.39 ) | 61.34 ( 46.82 - 85.68 ) | 105.92 | -0.33 ( -0.77 - 0.11 ) |
| UK | Male | 17441.43 ( 16995.79 - 17946.16 ) | 45.2 ( 44.06 - 46.5 ) | 15226.86 ( 14610.39 - 15861.41 ) | 28.23 ( 27.08 - 29.41 ) | -12.7 | -2.06 ( -2.18 - -1.94 ) |
| Ukraine | Male | 73055.72 ( 69165.26 - 76790.74 ) | 244.54 ( 232.21 - 256.85 ) | 49334.62 ( 45068.17 - 53850.48 ) | 163.61 ( 149.45 - 178.37 ) | -32.47 | -2.56 ( -3.02 - -2.09 ) |
| United Arab Emirates | Male | 435.05 ( 307.25 - 660.3 ) | 93.12 ( 69.41 - 121.56 ) | 4198.44 ( 2772.54 - 5899.46 ) | 91.32 ( 55.46 - 126.85 ) | 865.04 | 0.03 ( -0.07 - 0.12 ) |
| Uruguay | Male | 4019.54 ( 3774.12 - 4274.42 ) | 227.72 ( 213.97 - 242 ) | 2515.65 ( 2106.79 - 2945.92 ) | 116.1 ( 97.06 - 136.28 ) | -37.41 | -2.54 ( -2.68 - -2.4 ) |
| USA | Male | 89862.06 ( 87639.69 - 92676.43 ) | 65.95 ( 64.31 - 68.03 ) | 100901.45 ( 96261.13 - 105519.45 ) | 41.08 ( 39.18 - 43 ) | 12.28 | -2.18 ( -2.32 - -2.04 ) |
| Uzbekistan | Male | 5053.38 ( 4759.37 - 5353.41 ) | 88.34 ( 83.23 - 93.49 ) | 6518.97 ( 5521.19 - 7706.71 ) | 57.74 ( 49.22 - 67.35 ) | 29 | -1.3 ( -1.66 - -0.93 ) |
| Vanuatu | Male | 30.23 ( 21.23 - 52.51 ) | 73.83 ( 53 - 126.64 ) | 74.52 ( 49.1 - 159.16 ) | 79.79 ( 53.24 - 169.32 ) | 146.48 | 0.4 ( 0.32 - 0.47 ) |
| Venezuela | Male | 4882.37 ( 4576.1 - 5203.4 ) | 98.51 ( 92.43 - 104.94 ) | 12030.42 ( 9754.89 - 14623.33 ) | 86.87 ( 69.84 - 105.32 ) | 146.41 | -0.72 ( -0.85 - -0.58 ) |
| Vietnam | Male | 14925.39 ( 12250.01 - 17853.21 ) | 80.44 ( 65.83 - 96.13 ) | 36884.64 ( 29682.01 - 45975.07 ) | 81.58 ( 66.19 - 101.01 ) | 147.13 | 0.11 ( -0.03 - 0.26 ) |
| Virgin Islands | Male | 45.17 ( 39.9 - 51.22 ) | 102.94 ( 91.56 - 116.61 ) | 99.74 ( 77.07 - 120.07 ) | 115.75 ( 90.3 - 138.58 ) | 120.8 | 0.67 ( 0.55 - 0.79 ) |
| Yemen | Male | 2844.61 ( 1504.55 - 4071.58 ) | 97.68 ( 54.12 - 138.06 ) | 6167.75 ( 4501.26 - 8059.02 ) | 88.76 ( 64.86 - 115.78 ) | 116.82 | -0.39 ( -0.45 - -0.32 ) |
| Zambia | Male | 1819.03 ( 1329.62 - 2577.42 ) | 105.17 ( 78.74 - 148.75 ) | 2754.33 ( 2079.36 - 4370.1 ) | 75.07 ( 57.65 - 119.03 ) | 51.42 | -1.91 ( -2.25 - -1.57 ) |
| Zimbabwe | Male | 2398.32 ( 1986.29 - 2861.64 ) | 103.22 ( 85.96 - 122.34 ) | 4216.37 ( 3209.3 - 5280.66 ) | 122.33 ( 94.15 - 151.83 ) | 75.81 | 1.09 ( 0.31 - 1.87 ) |

DALY: disability adjusted life-year; EAPC: estimated annual percentage change; CI: confidence interval; UI: uncertainty interval; SDI: socio-demographic index
